# Discovery of a simple iron catalyst reveals the intimate steps of C–H amination to form C–N bonds†

**DOI:** 10.1039/d2sc04170g

**Published:** 2022-12-28

**Authors:** Wowa Stroek, Martin Albrecht

**Affiliations:** a Department of Chemistry, Biochemistry, and Pharmaceutical Sciences, University of Bern CH-3012 Bern Switzerland Martin.albrecht@unibe.ch

## Abstract

Formation of ubiquitous C–N bonds traditionally uses prefunctionalized carbon precursors. Recently, metal-catalyzed amination of unfunctionalized C–H bonds with azides has become an attractive and atom-economic strategy for C–N bond formation, though all catalysts contain sophisticated ligands. Here, we report Fe(HMDS)_2_ (HMDS = N(SiMe_3_)_2_^−^) as an easy-to-prepare catalyst for intramolecular C–H amination. The catalyst shows unprecedented turnover frequencies (110 h^−1^*vs.* 70 h^−1^ reported to date) and requires no additives. Amination is successful for benzylic and aliphatic C–H bonds (>80% yield) and occurs even at room temperature. The simplicity of the catalyst enabled for the first time comprehensive mechanistic investigations. Kinetic, stoichiometric, and computational studies unveiled the intimate steps of the C–H amination process, including the resting state of the catalyst and turnover-limiting N_2_ loss of the coordinated azide. The high reactivity of the iron imido intermediate is rationalized by its complex spin system revealing imidyl and nitrene character.

## Abstract 2

Fe(HMDS)_2_ is an efficient catalyst for the intramolecular C–H amination using azides as nitrene precursors; its simplicity offers crucial insights into the mechanism of this reaction.

## Graphical abstract

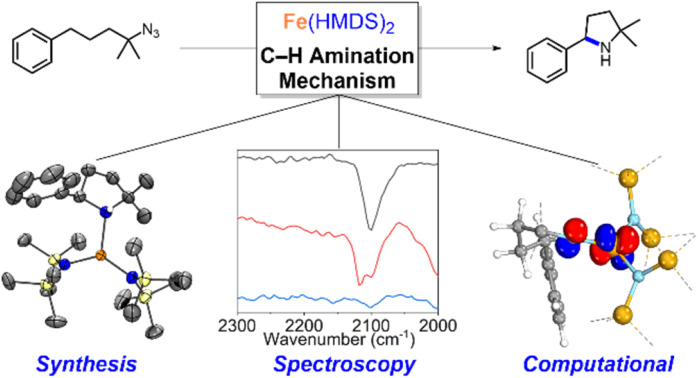

## 1 Introduction

Despite their ubiquitous presence, formation of C–N bonds has been challenging from a synthetic point of view.^1–3^ Typical methodologies for C–N bond formation include reductive amination, nucleophilic substitutions, and related processes that all require pre-installed directing and functional groups.^4–8^ These transiently installed groups generally compromise the atom-economy of the reaction and lead to considerable amounts of waste. A much more attractive approach towards C–N bond formation is the direct amination of unactivated C–H bonds, a method that relies on catalysts capable of transferring and inserting nitrenes.^9^ Many different nitrene sources have been utilized including iminoiodanes,^10^ chloroamines,^11^ hydroxylamines,^12–14^ anthranils,^15^ nitrosoarenes^16^ and others.^17–20^ Despite their high reactivity, these nitrene sources are difficult to prepare and show poor atom-economy as they produce considerable amounts of waste products in the process of generating the reactive nitrene. Using organic azides as the nitrene precursor mitigates these issues, as only dinitrogen is formed as a benign side product. However, organic azides are by far the least reactive of these nitrene precursors, which adds to the challenge of catalyst development.^21^

In pioneering work, Betley *et al.* described the use of an iron dipyrrin complex for the intramolecular C–H amination with organic azides to form 5-membered N-heterocycles,^22^ a class of compounds relevant to the pharmaceutical industry.^23,24^ Based on this breakthrough, several other catalysts were developed by using sophisticated ligand design and involving iron,^25–34^ cobalt,^35–39^ nickel,^40,41^ ruthenium^42,43^ and palladium.^44,45^ Currently, iron is the most attractive metal for this transformation with high turnover numbers (TONs) up to 7600,^31^ and with additional benefits such as low costs, low toxicity and high Earth-abundance.^46^ Despite the popularity of iron for this transformation, remarkably little is known about the mechanism of C–H amination with azides.^29^ In part, mechanistic studies have been complicated by the fact that a stoichiometric amount of Boc_2_O is needed in the catalytic reaction to prevent product inhibition.^25^ Only a few cobalt^37,39^ and nickel^40,41^ complexes, which do not require Boc_2_O as additive, were studied mechanistically by both experimental and computational methods.

Herein, we report the simple iron(ii) complex Fe(HMDS)_2_ (HMDS = N(SiMe_3_)_2_^−^) as a new catalyst for the intramolecular C–H amination using organic azides. The complex is conveniently synthesized in one single step using commercially available starting materials only,^47^ making it particularly interesting for utilization, as no ligand synthesis is required. This complex is catalytically active without the use of Boc_2_O or any other additive. The simplicity of the catalytic system provides a unique opportunity to investigate the catalytic mechanism in order to understand and rationally improve the catalytic process. We have therefore used both experimental and computational methods to elucidate this iron-catalyzed C–H amination reaction and also to identify critical decomposition pathways.

## 2 Results and discussion

### 2.1 Catalytic optimization

Fe(HMDS)_2_ was prepared from commercially available FeBr_2_ and LiHMDS and purified by distillation, according to established procedures.^47^ Reacting (4-azido-4-methylpentyl)-benzene (1a) with 1 mol% Fe(HMDS)_2_ in C_6_D_6_ at 100 °C resulted in the formation of 2,2-dimethyl-5-phenylpyrrolidine (1b) as the aminated product in 48% yield after 4 h (Table 1, entry 1). The conversion was slightly higher (56%) due to the formation of some cyclic imine as the most prominent side product together with small quantities of several other unidentified species. Performing the reaction in toluene-d_8_ resulted in comparable yields and conversions after 4 h (entry 2). Notably, THF-d_8_ significantly inhibited the catalytic activity and even after 24 h, yields did not exceed 38% (entry 3). We attribute the slow conversion to active site inhibition due to the coordinating ability of THF.^48^ Similar effects were observed with other under-ligated complexes.^22^ Increasing the temperature to 120 °C resulted in an increase of conversion (70%) and yield (58% after 4 h). To achieve full conversion, 24 h was required, as the reaction slows down in the presence of product (*vide infra*). Accordingly, an increase of catalyst loading to 2% further enhanced both conversion (82%) and yield (74%) within 4 h. The same trend was observed when utilizing 10 mol% catalyst loading, reaching full conversion in only 1 h. However, under these conditions the selectivity is significantly reduced to 62%. Lowering the catalyst loading to 0.1% resulted in a maximum yield of 18% after 4 d (entry 6). This performance corresponds to 180 turnovers, making Fe(HMDS)_2_ despite the simple ligand design one of the more robust iron catalysts known to date.^25–33^ Moreover, no Boc_2_O or other protecting groups were used during the catalysis, which is usually required for other iron systems.^25–33^ In fact, addition of Boc_2_O is deleterious and completely inhibits catalytic turnover.^49^ Notably, the reaction also proceeded at room temperature; at 1 mol% catalyst loading in toluene-d_8_ 43% yield and 52% conversion were accomplished after 14 d (entry 7). Even though the reaction is slow, this is the first iron complex that shows activity in this C–H amination process without heating.^25–33^ In the absence of Fe(HMDS)_2_1a is not converted at all within 7 d.

**Table 1:** Catalyst optimization for the intramolecular C–H amination by Fe(HMDS)_2_^a^

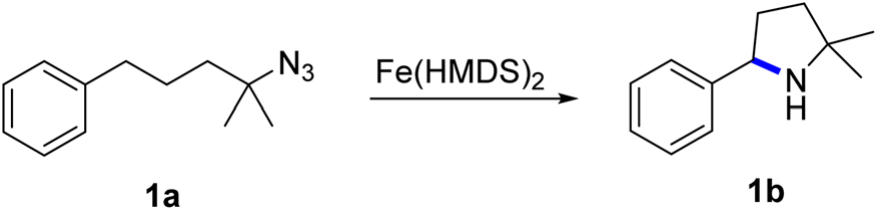						
Entry	Cat. loading (mol%)	Solvent	Temp. (°C)	Time	Yield^b^ (%)	Conversion^b^ (%)
1	1	C_6_D_6_	100	4 h	48	56
2	1	Toluene-d_8_	100	4 h	46	54
3	1	THF-d_8_	100	24 h	38	44
4	1	Toluene-d_8_	120	4 h/24 h	58/83	70/100
5	2	Toluene-d_8_	120	4 h/24 h	74/77	82/100
6	10	Toluene-d_8_	120	1 h	62	100
7	0.1	Toluene-d_8_	120	4 d	18	21
8	1	Toluene-d_8_	25	14 d	43	52
9	0	Toluene-d_8_	120	14 d	0	0

a Catalysis was performed on a 0.25 mmol scale in J Young NMR tubes; see the ESI for exact experimental details.

b Yields and conversions were determined by ^1^H NMR spectroscopy using 1,3,5-trimethoxybenzene as internal standard.

### 2.2 Kinetic aspects

**Fig. 1:**
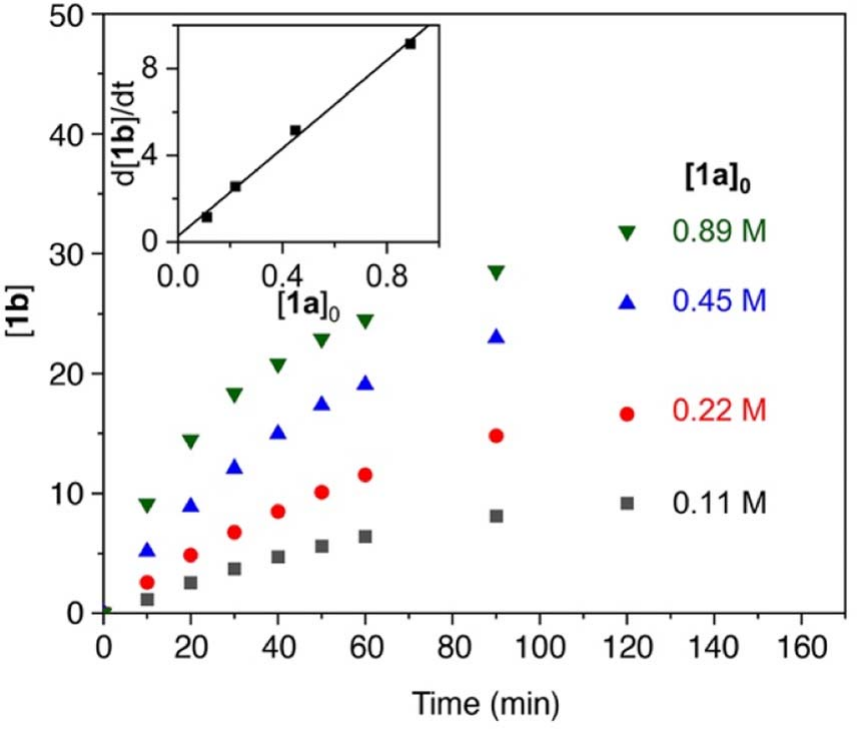
Time–conversion profiles for the C–H amination to yield 1b using different initial concentrations of substrate 1a. Inset displays the linear dependence of initial rates on the initial substrate concentration. See the ESI† for experimental details.

To gain more insights into the mechanism, kinetic experiments were performed. Firstly, the initial concentration of substrate 1a was varied under otherwise identical conditions.^50^ Time-dependent monitoring of the yields does not show any observable initiation period (Fig. 1). The pertinent time–conversion profiles clearly indicate a first order rate dependence on the initial substrate concentration for the first 10 minutes. Moreover, a gradual decrease of the reaction rate at longer reaction times was noted. The higher the initial substrate concentration, the faster this rate decrease occurs. This behavior might point towards product inhibition, as more amine product will be formed at higher initial substrate concentrations. To test this hypothesis, catalytic runs were carried out that were spiked with 1 equiv. of product 1b at different stages of conversion (0% and 25%). Product addition did not reveal any significant effect on the rate, clearly indicating that product inhibition is not a turnover-limiting factor in the catalytic reaction (Fig. S9†). Instead, the gradual loss of activity was attributed to catalyst decomposition. Such a process is supported by the observation of the protonated ligand, H–N(SiMe_3_)_2_, as a singlet at 0.08 ppm in the ^1^H NMR spectrum. To further support this model, two further experiments were carried out. Firstly, a new batch of substrate 1a was added to a catalytic run after reaching 70% conversion. This new batch was converted markedly slower (11% conversion in 3 h *vs.* 66% conversion in this time span with the initial substrate batch, Fig. S10†). Secondly, the catalyst was pre-heated to the reaction temperature, 120 °C, in the presence of a 20-fold excess of amine product 1b (0.2 equiv.) prior to substrate addition (1 equiv.). This run resulted in similarly poor conversion (9% after 3 h), in full agreement with catalyst deactivation in the presence of the amine product.

The first order dependence in substrate concentration is in line with what was observed for the previously reported cobalt porphyrin system by de Bruin *et al.*^35^ and our iron mesoionic carbene catalyst, which show substrate coordination or loss of dinitrogen to be rate limiting.^31^ Contrastingly, all other reported systems display a zero-order dependence on substrate concentration, suggesting hydrogen atom abstraction by the nitrene as the rate limiting step.^22,25,38,40,44^ The turnover frequency (TOF) of Fe(HMDS)_2_ at the highest substrate concentration reached 110 h^−1^, which is even faster than for the previously reported mesoionic carbene iron complex as the most active system so far (*cf.* TOF_max_ = 70 h^−1^ under identical conditions).^31^

**Fig. 2:**
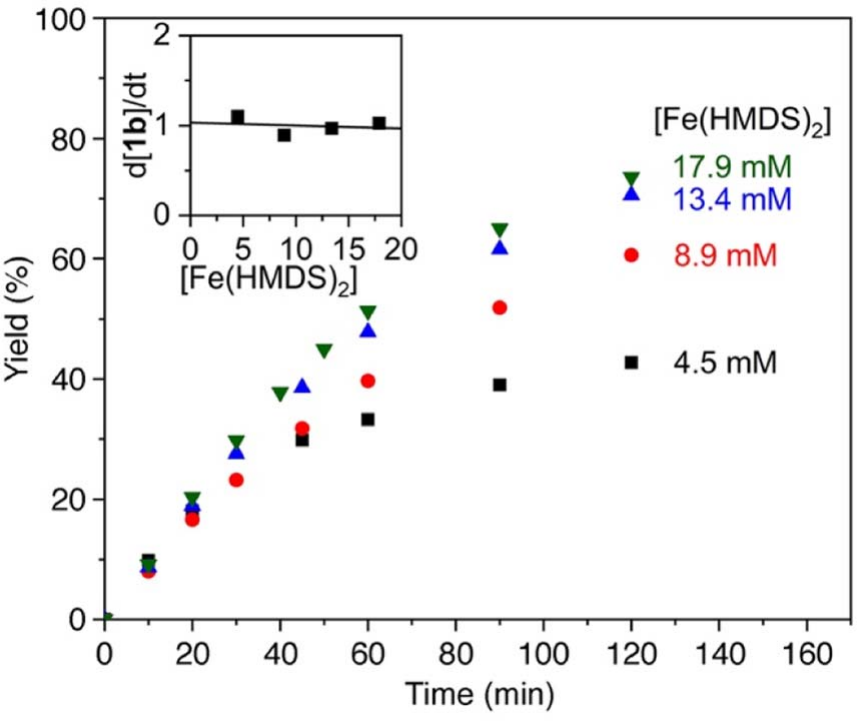
Time–conversion profiles for the formation of 1b using different catalyst concentrations (4.5 mM–17.9 mM) and 0.11 M 1a. Inset displays the independence of catalyst concentration on the initial reaction rates. See the ESI† for experimental details.

The reaction order in catalyst was investigated by varying the catalyst concentration under otherwise identical conditions. Analysis of the time-dependent yields of 1b showed no observable dependence of the catalyst concentration on the initial rate of the reaction (Fig. 2), suggestion an apparent zero-order dependence in catalyst. While these data might suggest that the catalyst is not involved in the turnover-limiting step, there are other scenarios that can lead to such an observation. In particular, reversible product coordination of the catalyst at the beginning of the reaction has been suggested to afford a negative dependence, which results in a net cancelling of the catalyst concentration in the rate law.^51^ Such a scenario is supported by the observation that at advanced stages of the reaction and increasing product concentration, the order in catalyst is not zero anymore.

### 2.3 Kinetic isotope effect

**Fig. 3:**
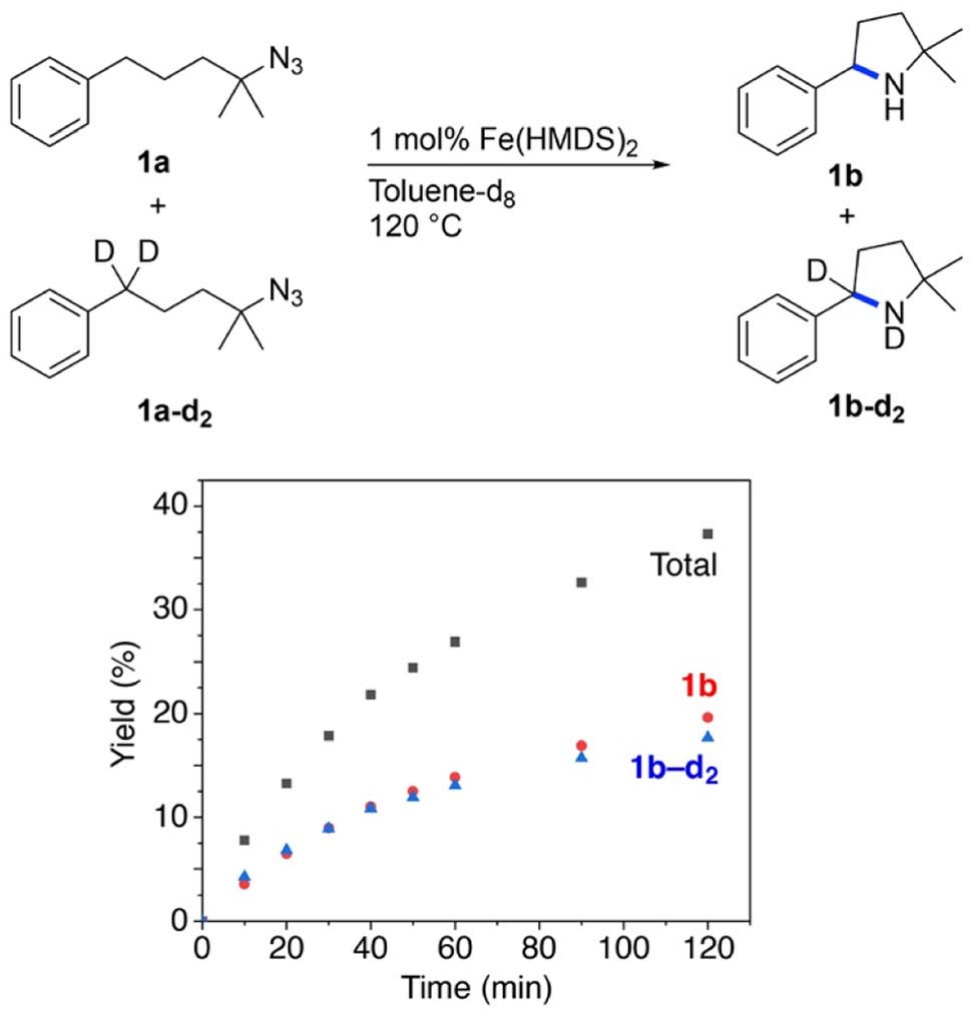
Time-dependent yield of 1b and 1b–d_2_ from intermolecular KIE competition experiments.

**Fig. 4:**
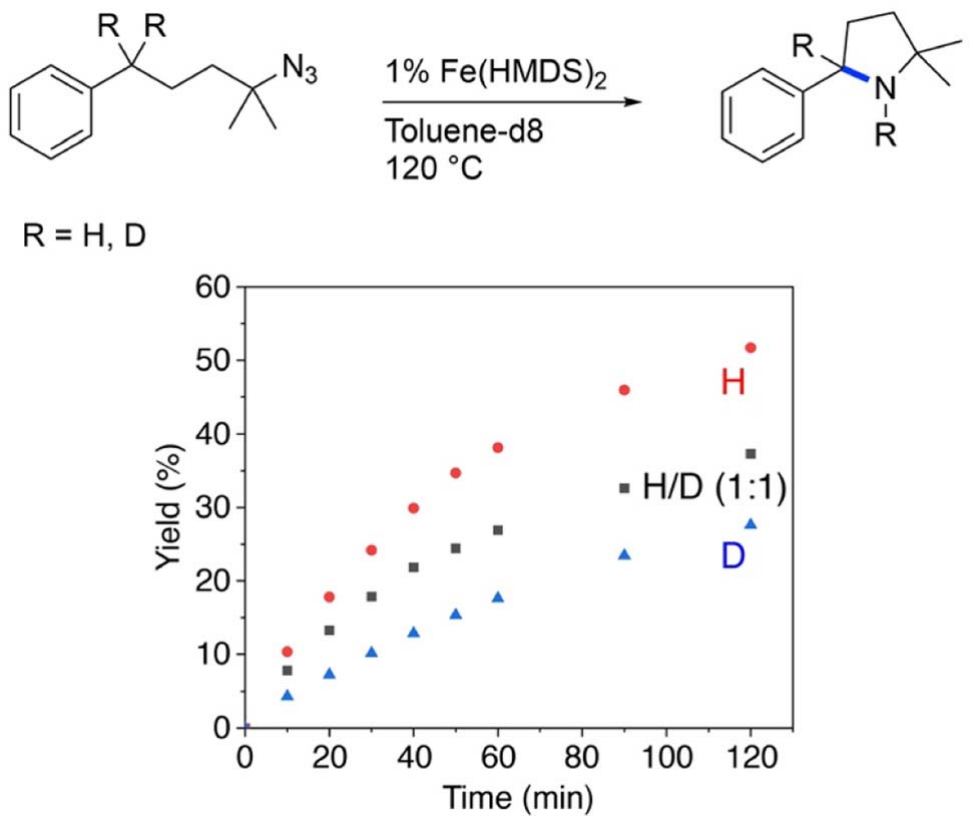
Evolution of C–H amination product 1b from azide 1a (red trace), 1a–d_2_ (blue trace), and a 1 : 1 mixture of the two substrates (black trace).

Further insights into the mechanism of the Fe(HMDS)_2_-catalyzed C–H amination were obtained from kinetic isotope experiments. Firstly, intermolecular competition experiments were performed, by reacting a 1 : 1 mixture of 1a and 1a–d_2_ (Fig. 3). The observation of equal ratios of 1b and 1b–d_2_ throughout the reaction indicates the absence of a KIE, commensurate with C–H/D bond breaking not being involved in the rate-determining step. This conclusion is in line with the kinetic data. Notably, the total rate of the intermolecular KIE competition experiment is significantly lower than the rate when exclusively using 1a (Fig. 4). The rate drops even further when performing the amination exclusively with deuterated substrate 1a–d_2_. Interestingly, the rate of the competition experiment using a 1 : 1 ratio of 1a and 1a–d_2_ is exactly the average of the rates from runs using 1a and 1a–d_2_ individually. Obviously, this dependence does not result from a regular KIE, as even in the competition experiment, a 1 : 1 ratio of the two isotopologue products is maintained throughout the whole reaction. Instead, secondary effects may rationalize the observed rate dependence. For example, stronger coordination of the deuterated pyrrolidine 1b–d_2_ compared to 1b increases the rate of catalyst decomposition, which lowers catalytic activity. Slightly stronger coordination of 1b–d_2_ is also predicted by density functional theory (DFT) calculations at the B3LYP^52,53^/def2-TZVP^54,55^ level,^56,57^ as the exchange of coordinated 1b with 1b–d_2_ at Fe(HMDS)_2_ is computed to be exergonic by 0.2 kcal mol^−1^ (Tables S3–S6†).

### 2.4 Stoichiometric experiments

**Fig. 5:**
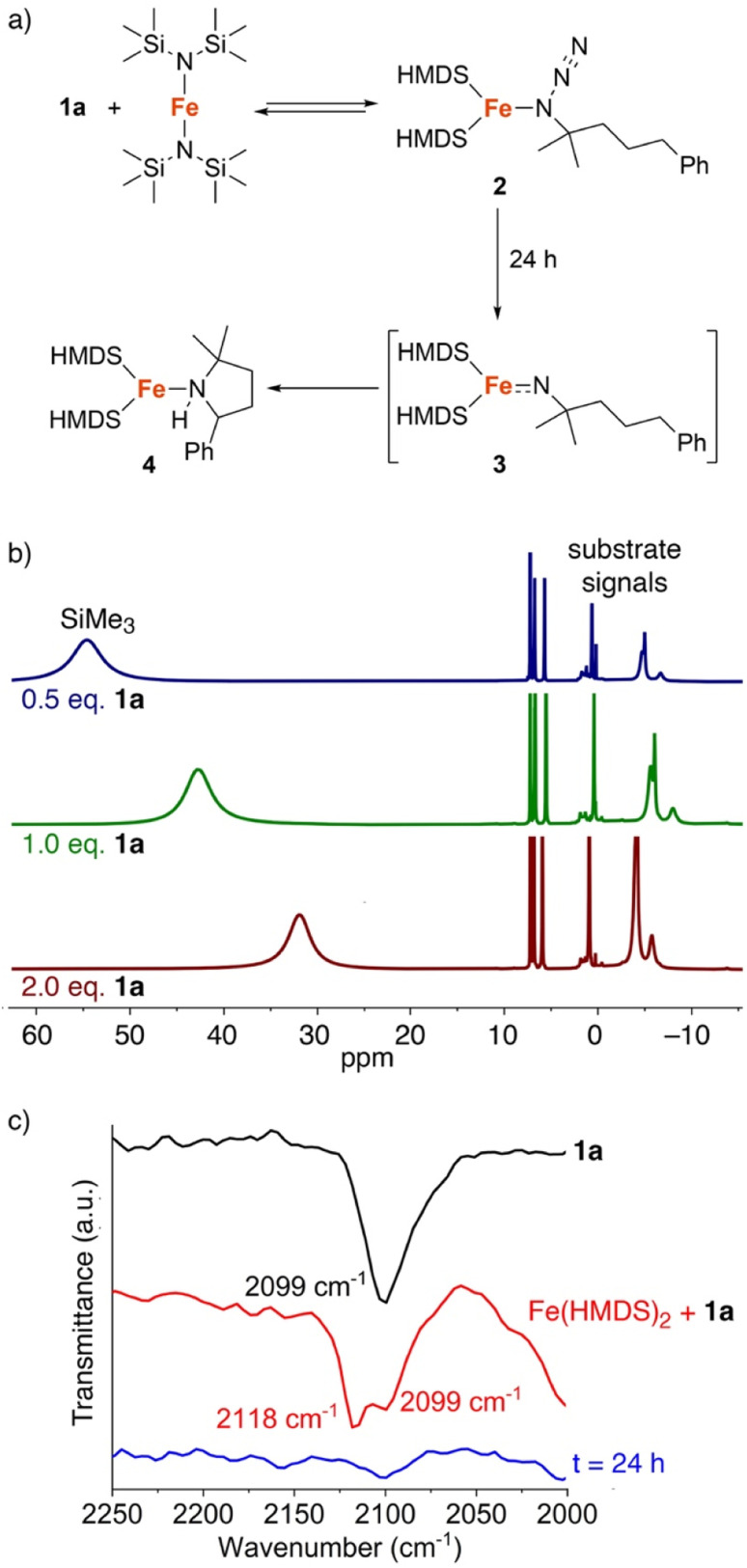
(a) Stoichiometric reaction of substrate 1a with Fe(HMDS)_2_; (b) ^1^H NMR spectra of the reaction with Fe(HMDS)_2_ with different equivalents of substrate 1a at *t* = 0 in C_6_D_6_; (c) FTIR-spectra of substrate 1a (black) and a mixture (1 : 1) of 1a and Fe(HMDS)_2_ at *t* = 0 (red) and after 24 h (blue).

The fact that C–H amination with Fe(HMDS)_2_ proceeds even at room temperature offers opportunities to monitor the reaction in detail. Therefore, a set of (sub)stoichiometric experiments were performed and analyzed by spectroscopic techniques. Upon addition of half an equivalent of model substrate 1a to Fe(HMDS)_2_ in C_6_D_6_ at ambient temperature, an immediate color change was observed from pale green to pale yellow, attributed to coordination of the azide substrate and formation of complex 2 (Fig. 5a). In ^1^H NMR spectroscopy, the signals for 1a experience paramagnetic perturbation and the singlet for Fe(HMDS)_2_ shifts upfield from 63 ppm to 55 ppm (Fig. 5b).

**Fig. 6:**
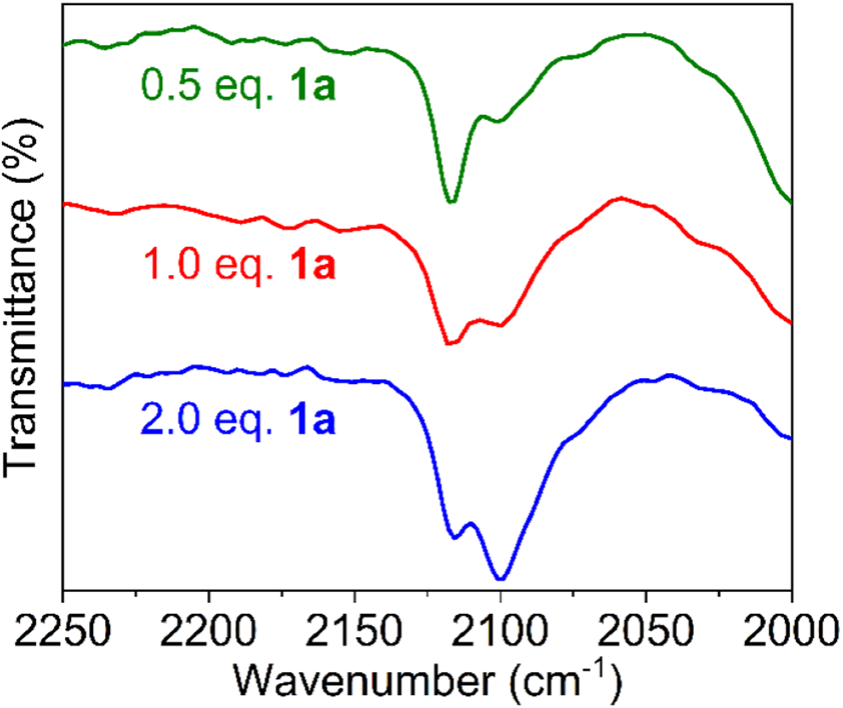
FTIR spectra of a mixture of substrate 1a and Fe(HMDS)_2_ at *t* = 0 using 0.5, 1.0 and 2.0 equivalents of 1a; free azide at 2099 cm^−1^, azide of 2 at 2118 cm^−1^.

When using one or two equivalents of 1a, comparable yet slightly shifted paramagnetic signals evolved in the ^1^H NMR spectrum, though no diamagnetic signals for the substrate were observed (Fig. 5b). The most significant change pertains to the SiMe_3_ signal of iron-bound HMDS, which shifted further upfield to 43 and 32 ppm, respectively. Notably, when using 10 equiv. of substrate, broadening of the substate signals is observed in the diamagnetic region, indicating interaction with the paramagnetic iron center (Fig. S14†). We attribute this observation to an equilibrium being reached between Fe(HMDS)_2_-coordinated azide 2 and non-coordinated substrate 1a, which is fast on the NMR time scale and gradually shifts to the product side as more substrate equivalents are used (Fig. 5b). This assignment was further supported by IR spectroscopy. Thus, a mixture of Fe(HMDS)_2_ with one equivalent of 1a resulted in two azide stretching bands at 2099 cm^−1^, corresponding to free 1a, and at 2118 cm^−1^, attributed to adduct 2 (Fig. 5c). The 19 cm^−1^ blue-shift upon coordination indicates no π-backbonding of the coordinated azide, in line with the cobalt system of Betley *et al.*^39^ It is worth noting that α-nitrogen coordination of organic azides to first-row transition metals has been detected only very rarely and only with cobalt.^39,58^ The observation of signals for free and coordinated azide supports the presence of an equilibrium. Further evidence of an equilibrium was obtained by changing the number of equivalents of 1a used for the IR measurement (Fig. 6). The use of only half an equivalent of azide resulted in a higher ratio of coordinated *vs.* free azide, while two equivalents of azide decreased this ratio. This strongly suggests single coordination of 1a to Fe(HMDS)_2_.

**Fig. 7:**
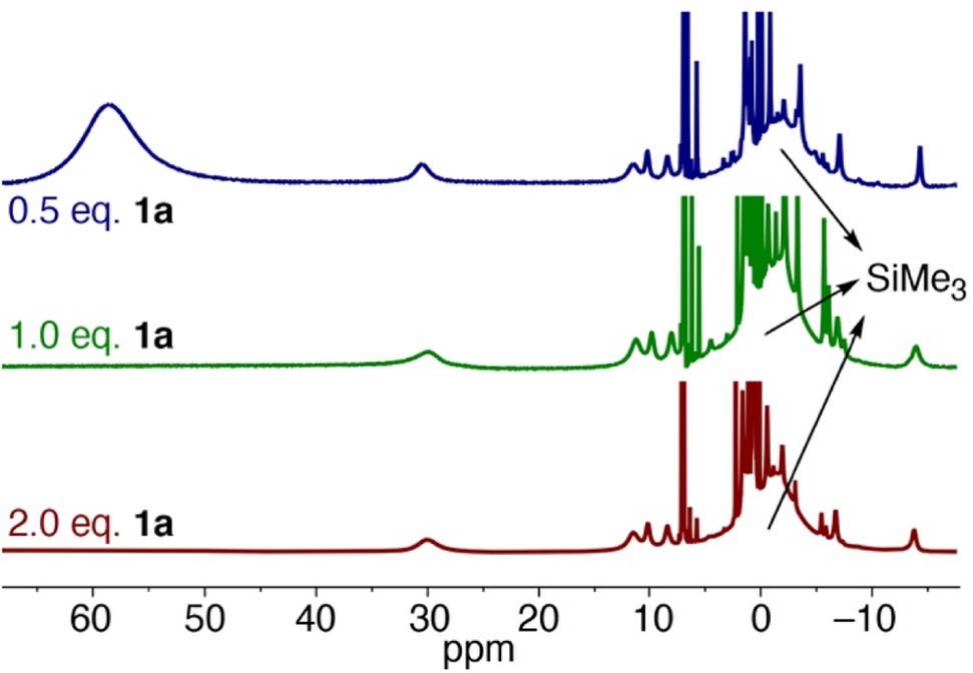
^1^H NMR spectra of the reaction of Fe(HMDS)_2_ with different equivalents of substrate 1a at *t* = 24 h in C_6_D_6_.

Upon leaving the mixture of 1a with one equivalent of Fe(HMDS)_2_ for 24 h, full conversion to a new species was observed, as determined by the formation of new paramagnetic signals in the ^1^H NMR spectrum (Fig. 7). Specifically, the signal for the HMDS group shifted to −1 ppm. Upon opening the reaction container, pressure release was noted, which was attributed to the release of dinitrogen indicating transient formation of a putative nitrene 3, and eventually the cyclized amine in the iron coordination sphere, *i.e.* complex 4 (*cf.*Fig. 5a). Using 2 equivalents of 1a resulted in the same signals in the ^1^H NMR spectrum. No unbound 1b was detected, indicating a fast exchange between coordinated and uncoordinated 1b. Interestingly when using only 0.5 equiv. of azide 1a with Fe(HMDS)_2_ for 24 h, the same new paramagnetic signals are observed in the ^1^H NMR spectrum at −1 ppm, and in addition there is also a broad signal at 59 ppm in an approximately 1 : 1 relative ratio (Fig. 6), which was attributed to Fe(HMDS)_2_. This 1 : 1 stoichiometry of the Fe-bound product and Fe(HMDS)_2_ suggests that migration of 1b from one Fe(HMDS)_2_ unit to another is slow on the NMR time scale. We note that this behavior is incompatible with a dissociative mechanism for product release from the metal center.

**Fig. 8:**
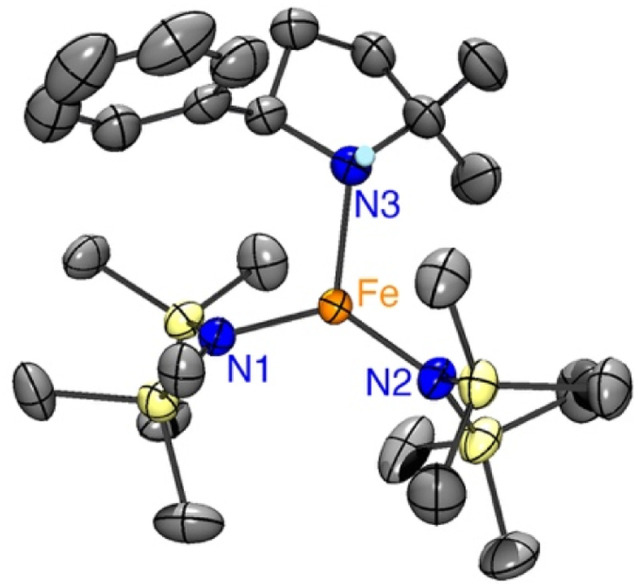
ORTEP representation of 4 (50% probability ellipsoids, all carbon-bound H atoms omitted for clarity).

To elucidate the structure of 4, single crystals suitable for X-ray diffraction were grown by slow evaporation of pentane at −30 °C from a 1 : 1 reaction mixture of Fe(HMDS)_2_ and 1a. The structure of the colorless crystals displays a trigonal coordination geometry around the iron, comprising two HMDS moieties and one cyclized amine product (Fig. 8). The N–H hydrogen of the cyclized amine was located from the residual electron density on the Fourier difference map and refined using a riding model.

### 2.5 Computed mechanism

**Scheme 1:**
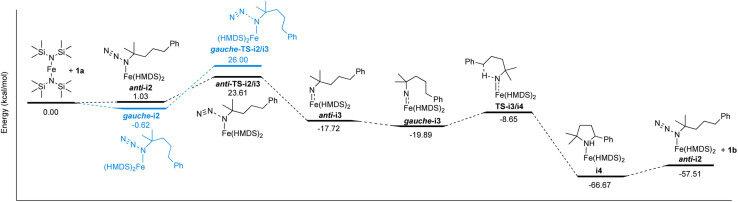
Reaction coordinate profile of the proposed mechanism for the intramolecular C–H amination catalyzed by Fe(HMDS)_2_. Energies calculated by DFT are giving in kcal mol^−1^ (see Fig. S27–S40† for structures of optimized intermediates and transition states).

The well-behaved kinetics, the establishing of a pre-equilibrium and the isolation of molecularly defined intermediates in stoichiometric experiments strongly indicate that Fe(HMDS)_2_ is a homogeneous catalyst in the intramolecular C–H amination reaction. Therefore, this fairly simple catalytic system is amenable to computational investigations to further elucidate the mechanism, to identify critical intermediates, and to better characterize the key C–N bond formation step. To this end, DFT calculations were performed at the B3LYP^52,53^/def2-TZVP^54,55^ level.^56,57^ Coordination of azide 1a on Fe(HMDS)_2_ forming *anti*-i2 was calculated to be 1.0 kcal mol^−1^ uphill (Scheme 1). Notably, conformational isomerization of *anti*-i2 to *gauche*-i2 led to stabilization of the complex by 1.7 kcal mol^−1^, making azide coordination 0.6 kcal mol^−1^ downhill compared to Fe(HMDS)_2_. This small energy difference is in line with the observed equilibrium of azide coordination in ^1^H NMR and FTIR spectroscopies (*vide supra*). Loss of dinitrogen to form the corresponding nitrene complex *gauche*-i3 has an energy barrier of 26.6 kcal mol^−1^ (*gauche-*TS-i2/i3), yet it is only 22.6 kcal mol^−1^ for the *anti*-conformer (*anti-*TS-i2/i3). Therefore, loss of dinitrogen and formation of the corresponding nitrene complex is predicted to occur through *anti*-i2/i3, as the activation energy is 2.4 kcal mol^−1^ lower. Formation of *anti*-i3 was calculated to be 18.8 kcal mol^−1^ downhill from the azide complex i2, with further stabilization of 2.2 kcal mol^−1^ after isomerization to the *gauche* conformation of the nitrene complex (*gauche*-i3). The pentet spin state (*S* = 2) was determined to be the most stable configuration in comparison to the triplet (*S* = 1, Δ*E* = +3.6 kcal mol^−1^), septet (*S* = 3, Δ*E* = +6.7 kcal mol^−1^), or the pentet broken symmetry solution with two anti-ferromagnetically coupled unpaired electrons (Δ*E* = +3.1 kcal mol^−1^; Table S4†). Interestingly, the pentet spin state resulted in a computed expectation value 〈*S*^2^〉 of 6.48, significantly higher than the ideal 6.00 for a pentet spin-system. On top of that, the spin density plot displayed both α- and β-spin on the nitrene nitrogen (Fig. S22†). This indicates multireference character, which cannot be computed by DFT, and more sophisticated methods such as complete active space self-consistent field (CASSCF) calculations are required to elucidate the exact spin-state (*vide infra*).

**Fig. 9:**
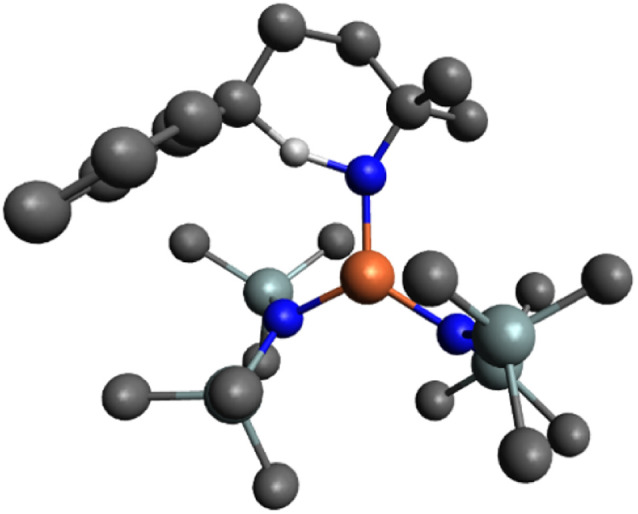
Ball and stick representation of the optimized transition state TS-i3/i4 by DFT (B3LYP/def2-TZVP) in the pentet spin state. H atoms on all carbons omitted for clarity (Fe orange, N blue, Si mint, C grey, H off-white).

The next step in the mechanism, based on literature examples, would be a hydrogen atom abstraction (HAA) to form a carbon-centered radical.^40^ In order to find the transition state of this transformation a relaxed surface scan was performed by decreasing the distance of the benzylic hydrogen and the nitrene. Throughout the whole scan, the carbon-centered radical was not formed and during the HAA, the cyclized product was directly formed with just one energy barrier. Accordingly, the HAA and the formation of the C–N bond proceed in one single concerted step with an energy barrier of 11.2 kcal mol^−1^ yielding the coordinated cyclized product (i4), which is 46.8 kcal mol^−1^ downhill from the nitrene complex. The corresponding transition state (TS-i3/i4) consists of the hydrogen atom bonded to both the carbon and the nitrogen center with a distance of 1.273 Å and 1.318 Å respectively, and a C⋯N internuclear distance of 2.537 Å (Fig. 9).

To close the catalytic cycle, either 1b has to dissociate from i4 to regenerate Fe(HMDS)_2_, or alternatively, the coordinated amine is substituted by the azide substrate through an associative or an interchange mechanism. The dissociative mechanism to form Fe(HMDS)_2_ was computed to be 8.1 kcal mol^−1^ uphill for the dissociation of the amine, followed by 1.0 kcal mol^−1^ uphill for the azide coordination to form i2. Performing a geometry optimization for the associative mechanism, resulted in decoordination of the azide, making the associative mechanism unlikely. We were unable to compute any transition state for the three possible mechanisms, despite multiple attempts using relaxed surface scan and nudged elastic band (NEB) calculations. Finding transition states with computational methods of reactions where entropy is a major contributor such as associations and dissociations is a major challenge, as only electronic energies are scanned on the energy surface.^59,60^ Due to this, we cannot distinguish the energy pathway of the dissociative and interchange mechanisms using DFT, as they both lead to the same product without going through higher-energy computationally characterized intermediates. However, several experimental facts are incompatible with a dissociative mechanism and hence strongly support an interchange process: (i) crystallization of the iron-coordinated product 4 (Fig. 8) identifies this complex as the resting state, (ii) the reaction is first-order in azide while in a dissociative substitution, it should be zero-order in incoming ligand unless Fe(HMDS)_2_ were the resting state, and (iii) stoichiometric experiments using Fe(HMDS)_2_ and half an equivalent of 1a did not show any exchange of pyrrolidine between 4 and Fe(HMDS)_2_. Notably, the overall calculated reaction profile is in excellent agreement also with the pre-equilibrium between 4 and 2. Furthermore, nitrene formation through loss of dinitrogen as a rate-limiting process features a computed ∼24 kcal mol^−1^ energy barrier, which is just about accessible even at room temperature, rationalizing the slow kinetics.

The rate of the reaction significantly decreases over time, combined with formation of H–N(SiMe_3_)_2_ (*vide supra*). We hypothesized a catalyst decomposition pathway, in which a coordinated HMDS group deprotonates the coordinated amine in the resting state 4, leading to decoordination of H–N(SiMe_3_)_2_ and pyrrolidinyl bonding to iron. According to DFT calculations this transformation is 9.7 kcal mol^−1^ uphill, only slightly higher than for the substitution with another substrate molecule and therefore potentially competitive as a decomposition pathway (Schemes S1 and S2†). Attempts to isolate and characterize any degradation product have not been successful so far, though the decoordinated H–N(SiMe_3_)_2_ was unambiguously identified by ^1^H NMR spectroscopy.

### 2.6 NEVPT2-CASSCF analysis of the nitrene intermediate

**Fig. 10:**
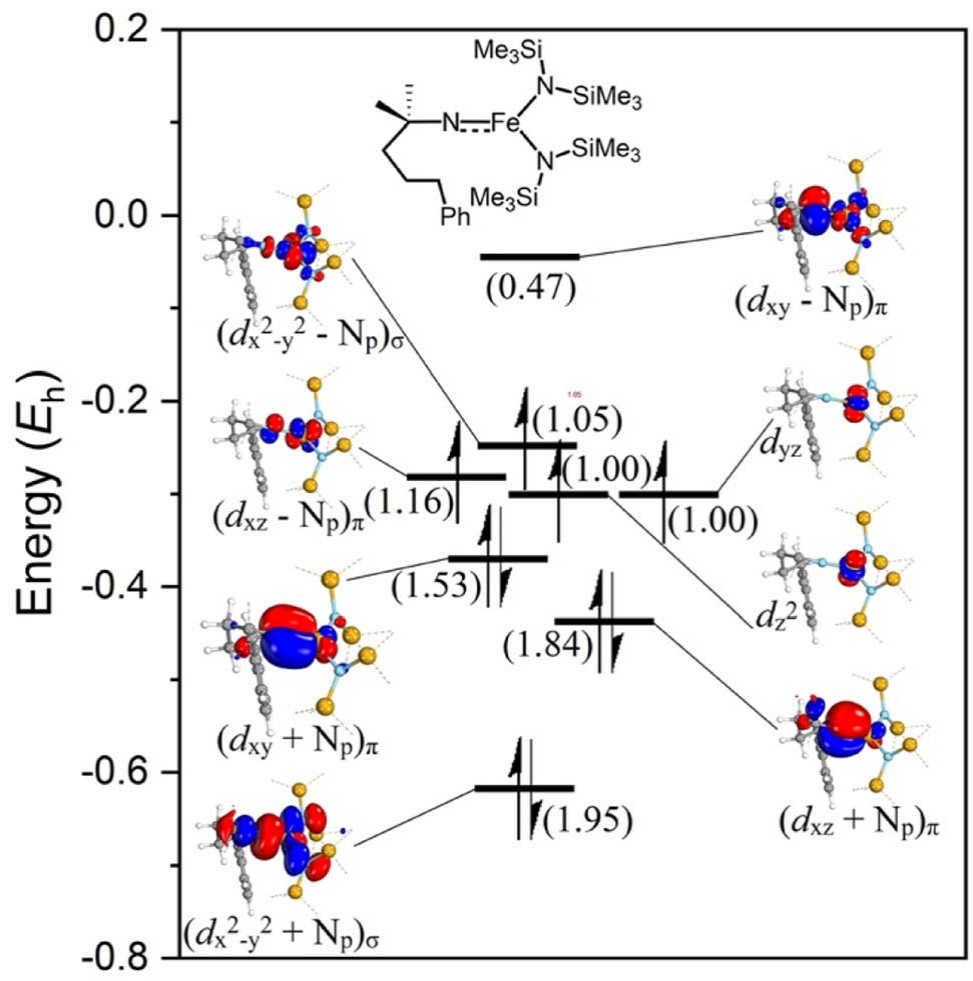
Active space orbitals from a NEVPT2-CASSCF(10,8) calculation on *gauche*-i3. Orbital filling of the main contribution (55%) is illustrated. Partial occupation due to multi-reference character described in brackets per orbital. Isosurface is set at 90. All Me groups omitted for clarity.

The electronic configuration of the M–NR moiety in intermediate 3 plays a crucial role in the reactivity with C–H bonds, and in particular, imidyl/nitrene character strongly benefits reactivity.^61^ Better insight into the electronic configuration was obtained by performing *n*-electron valence state second-order perturbation theory (NEVPT2) corrected CASSCF calculations on the DFT-optimized M–NR structure.^62–64^ A NEVPT2-CASSCF(10,8) calculation on *gauche*-i3 including all the metal d-orbitals, nitrene orbitals, and p-orbitals of the HMDS-ligands in the active space showed significant multireference character for this intermediate. The lead electronic state, which contributes 55% towards the ground state, features three doubly occupied molecular orbitals (MOs), which represent a bonding combination between the iron and the nitrene nitrogen (Fig. 10; see Fig. S23† for all contributions). Two of these MOs represent a π-interaction and one MO a σ-interaction. Furthermore, two singly occupied molecular orbitals (SOMOs) predominantly display the two non-bonding iron *d*_*yz*_ and *d*_*z*_^2^ orbitals. The other two SOMOs consist of antibonding interactions between the Fe–N bond, one σ*- and one π*-interaction. Finally, the lowest unoccupied molecular orbital (LUMO) represents the second antibonding π*-interaction in the Fe–N bond. This brings the total bond order of the Fe–N bond in the predominant configuration to 2.0. However, due to contributions of higher spin states, significant electron density is transferred from the bonding π-orbitals of the Fe–N bond (occupancy 1.84 and 1.53) to the anti-bonding combination of those orbitals (occupancy 1.16 and 0.47). This shift results in a decreased bond order of 1.32 for the Fe–N interaction, giving rise to Fe–N bond elongation and significant imidyl and nitrene character. Indeed, the DFT computed Fe–N bond length of 1.737 Å exceeds the range of 1.612(2)–1.6723(18) Å for all known Fe(iv) imido complexes.^65–71^ Notably, two iron imido complexes reported by Deng *et al.*^69^ and Betley *et al.*^72^ show a similar Fe–N bond elongation to 1.708(2) Å and 1.768(4) Å, respectively. However, both these complexes display a significant amount of imidyl radical character. Both the bond length obtained from the DFT optimized structure and the results from the NEVPT2-CASSCF calculations indicate imidyl and nitrene character, thus rationalizing the high reactivity of the complex.

### 2.7 Substrate modifications

Since all mechanistic and kinetic data lend strong support to a molecularly well-defined and homogeneous catalyst, the activity of Fe(HMDS)_2_ towards various benchmark substrates was explored. A limited number of functional groups in the *para*-position of model substrate 1a such as –Me, –OMe, or –Br groups (5a–7a) did not give any significant change in yield of the aminated product nor any correlation of the reaction rates with Hammett *σ*_p_ parameters (Table 2, entries 2–4). Substituting the phenyl group with an electron-rich and weakly coordinating thiophene group in 8a was also tolerated (entry 5). Primary benzylic C–H bonds were also activated, but the yield after 3 and 24 h was significantly lower (9a, entry 6).

**Table 2:** Catalyst optimization for the intramolecular C–H amination by Fe(HMDS)_2_^a^

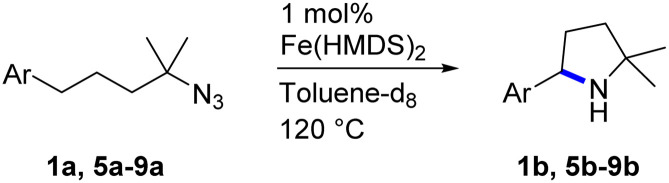				
Entry	Substrate	Product	Time^b^ (h)	Yield^b^ (%)
1	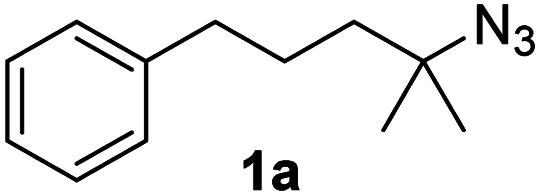	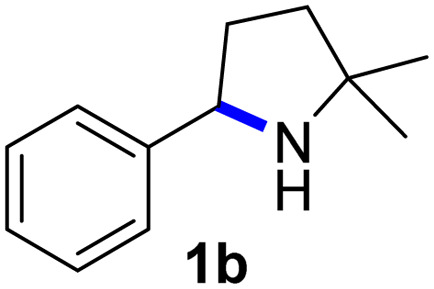	3/24	52/83
2	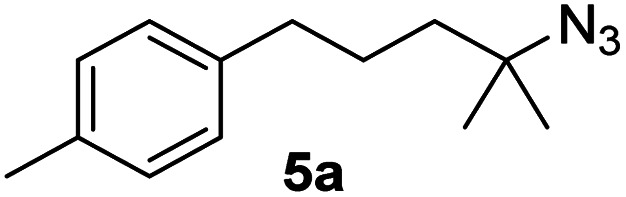	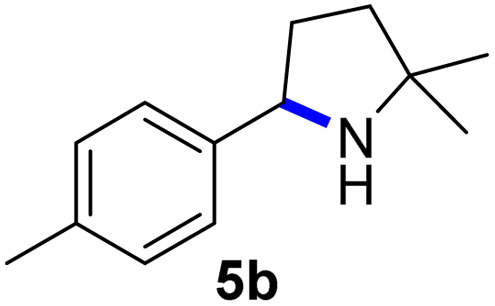	3/24	53/82
3	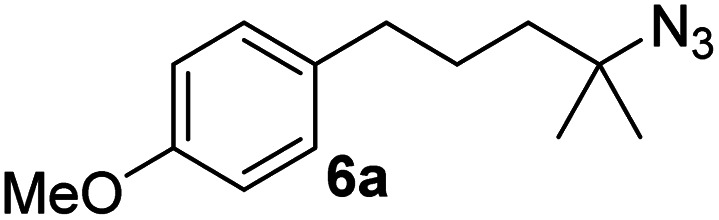	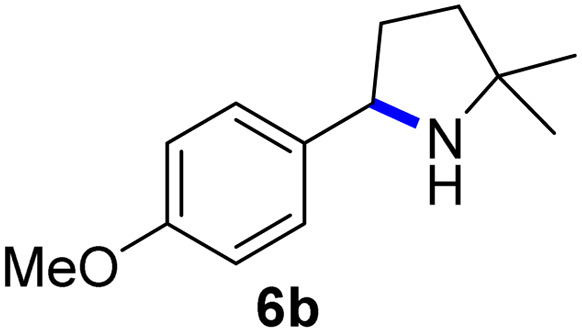	3/24	62/83
4	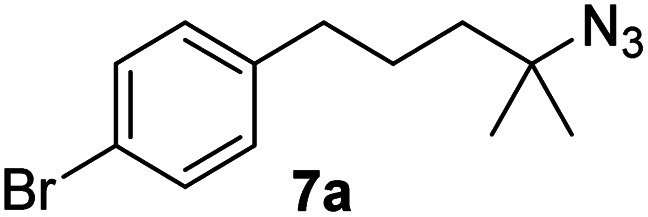	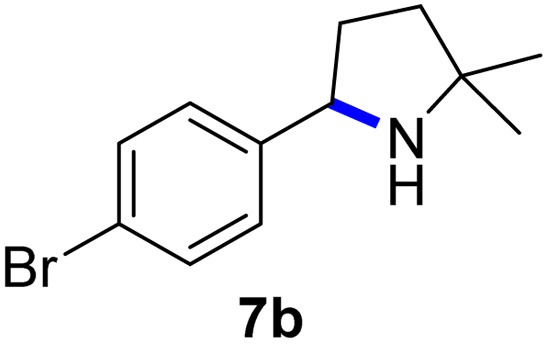	3	91
5	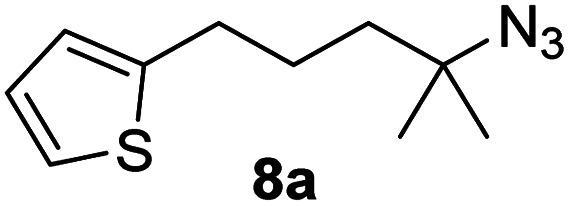	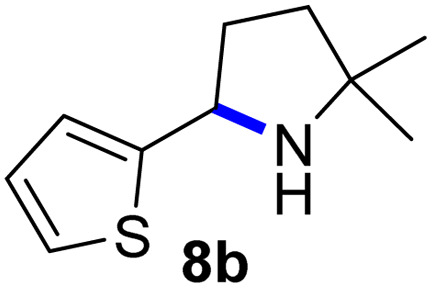	3/24	62/87
6	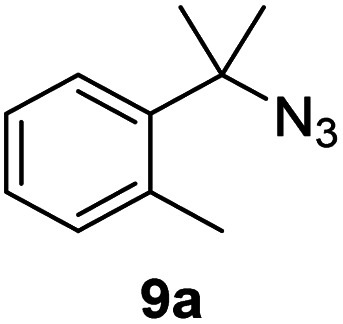	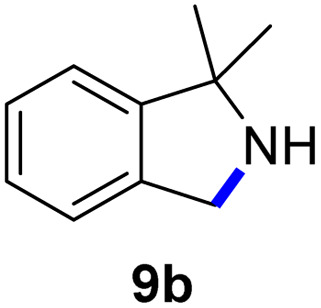	3/24	3/18

a Catalysis was performed on a 0.25 mmol scale in J Young NMR tubes; see the ESI for exact experimental details.

b Yields and conversions were determined by ^1^H NMR spectroscopy using 1,3,5-trimethoxybenzene as internal standard.

Moreover, Fe(HMDS)_2_ catalyzed the amination of stronger aliphatic C–H bonds. Reacting (5-azido-5-methylhexyl)benzene 10a resulted in selective formation of the benzyl-substituted 5-membered N-heterocycle 10b (Table 3, entry 1). No 6-membered product was observed, even though formation of the latter would be energetically more favorable due to the weaker bond dissociation energy (BDE) of the benzylic C–H bond in 10a,^73^ suggesting a kinetic reaction control. Simple alkyl azides also underwent intramolecular C–H amination. A clear trend of declining yields after 3 and 24 h was observed from tertiary to secondary and primary C–H bonds (11a, 12a, and 13a, respectively), which mirrors the increased C–H BDE (entries 2–4).^73^ This trend suggests that either C–H bond activation becomes rate-limiting for those substrates, or that product coordination is more effective due to the stronger coordination ability of these aliphatic pyrrolidines. Formation of bridged N-heterocycle 14b and spirocyclic pyrrolidine 15b was also achieved by Fe(HMDS)_2_ (entries 5 and 6). Incorporation of an alkyl bromide as in 16a suppressed C–H amination. This lack of activity is attributed to a radical transfer from the nitrene in the presence of the alkyl bromide, which causes catalyst decomposition.^40^

**Table 3:** Scope of the intramolecular amination reaction of aliphatic C–H bonds^a^

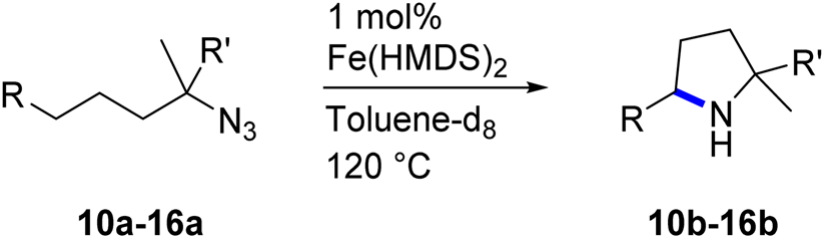				
Entry	Substrate	Product	Time^b^ (h)	Yield^b^ (%)
1	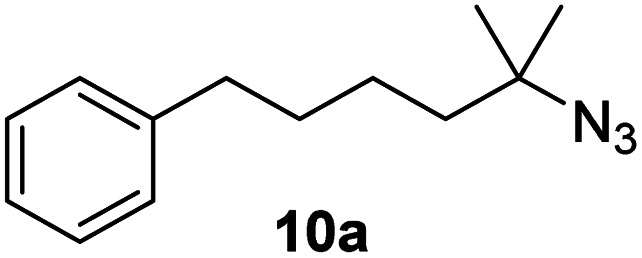	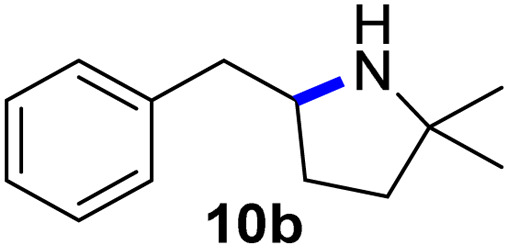	3/24	18/48
2	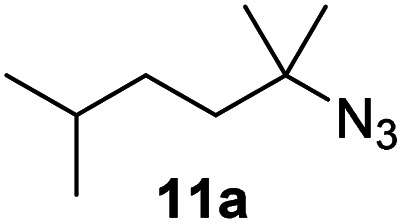	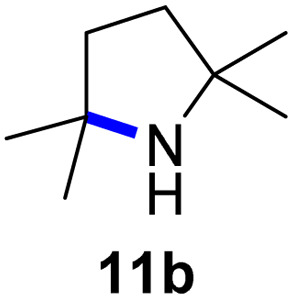	3/24	38/82
3	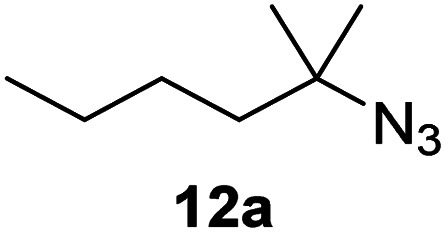	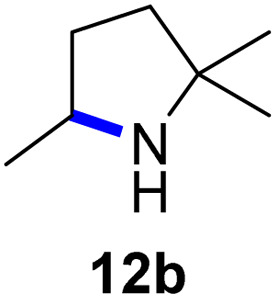	3/24	19/67
4	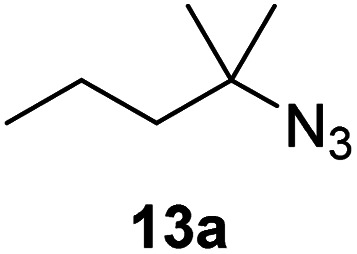	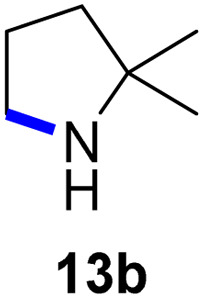	3/24	5/18
5	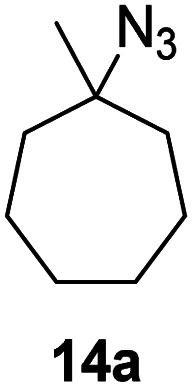	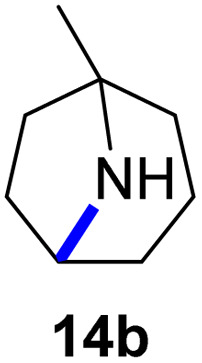	3/24	29/51
6	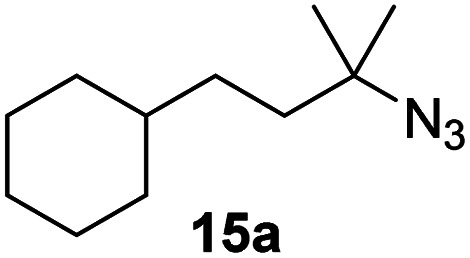	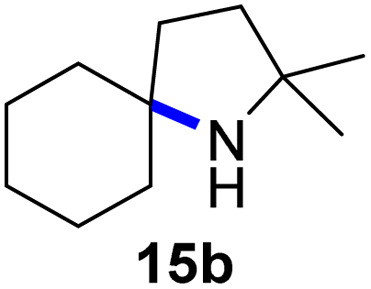	3/24	45/92
7	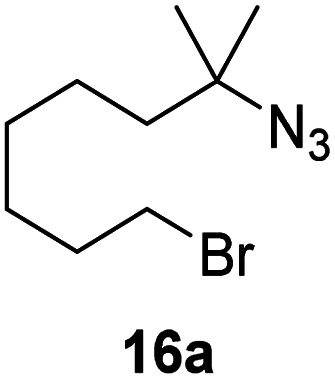		24	0

a Catalysis was performed on a 0.25 mmol scale in J Young NMR tubes; see the ESI for exact experimental details.

b Yields and conversions were determined by ^1^H NMR spectroscopy using 1,3,5-trimethoxybenzene as internal standard.

## 3 Conclusions

Herein we have reported the intramolecular C–H amination of organic azides, catalyzed by Fe(HMDS)_2_ as a simple and easily accessible catalyst precursor. At the initial stages, the catalytic rate is unprecedently high and reaches TOFs up to 110 h^−1^. No Boc_2_O or any other additive is required for catalytic conversion, though product-induced catalyst deactivation at later reaction stages has been found to reduce catalytic activity. Nevertheless, appreciable TONs up to 180 are accomplished, which make this process synthetically simple and useful. A wide variety of C–H bonds were activated for amination, including benzylic and strong aliphatic C–H bonds, even in the presence of a variety of functional groups. The simplicity of the catalytic system allowed, for the first time, in-depth investigation of the mechanism of iron-catalyzed C–H bond amination. Experimental and computational data confirm an equilibrium between product and substrate coordination at iron, and rate-limiting N_2_ dissociation en route to nitrene formation. Subsequent cyclization occurs according to calculations through a concerted pathway featuring hydrogen atom transfer and simultaneous C–N bond formation, without involving a carbon centered radical as previously postulated for other iron complexes. Such concerted hydrogen transfer and C–N bond formation may be at play also with other catalysts. The proposed mechanism is supported by the characterization of two intermediates, by stoichiometric experiments, kinetic data, and computational studies. Furthermore, our data strongly point to a catalyst deactivation that proceeds through a well-defined decomposition pathway. Based on these insights, improved catalytic activity may ensue from suppressing catalyst decomposition, for example by using sterically more demanding amide ligands. In addition, the barrier for N_2_ loss may be lowered when using more electron-donating amide ligands, thus facilitating better activation of the azide substrate. These approaches are currently under investigation in our laboratory.

## Data availability

Experimental details, synthetic procedures, NMR spectra, crystallographic data, detailed catalytic procedures, and computational details are available in the ESI.†

## Author contributions

W. S and M. A conceived the project and contributed intellectually to its development. W. S performed experimental and computational experiments and analyzed the data. M. A supervised the project. W. S and M. A wrote the manuscript.

## Conflicts of interest

The authors declare no competing financial interest.

## Supplementary Material

SC-014-D2SC04170G-s001

SC-014-D2SC04170G-s002

## References

[cit1] Hili R., Yudin A. K. (2006). Nat. Chem. Biol..

[cit2] Kürti L. (2015). Science.

[cit3] Ricci A. , Amino Group Chemistry: From Synthesis to the Life Sciences, Wiley-VCH: Weinheim, 2008

[cit4] Bariwal J., Van Der Eycken E. (2013). Chem. Soc. Rev..

[cit5] Candeias N. R., Branco L. C., Gois P. M. P., Afonso C. A. M., Trindade A. F. (2009). Chem. Rev..

[cit6] Deiters A., Martin S. F. (2004). Chem. Rev..

[cit7] Cho S. H., Kim J. Y., Kwak J., Chang S. (2011). Chem. Soc. Rev..

[cit8] Kattamuri P. V., Yin J., Siriwongsup S., Kwon D. H., Ess D. H., Li Q., Li G., Yousufuddin M., Richardson P. F., Sutton S. C., Kürti L. (2017). J. Am. Chem. Soc..

[cit9] Park Y., Kim Y., Chang S. (2017). Chem. Rev..

[cit10] Dick A. R., Remy M. S., Kampf J. W., Sanford M. S. (2007). Organometallics.

[cit11] Kawano T., Hirano K., Satoh T., Miura M. (2010). J. Am. Chem. Soc..

[cit12] Ng K. H., Chan A. S. C., Yu W. Y. (2010). J. Am. Chem. Soc..

[cit13] Jarrige L., Zhou Z., Hemming M., Meggers E. (2021). Angew. Chem., Int. Ed..

[cit14] Zhong D., Wu L. Y., Wang X. Z., Liu W. B. (2021). Chin. J. Chem..

[cit15] Yu S., Tang G., Li Y., Zhou X., Lan Y., Li X. (2016). Angew. Chem., Int. Ed..

[cit16] Hu X. H., Yang X. F., Loh T. P. (2016). ACS Catal..

[cit17] Kim J. Y., Park S. H., Ryu J., Cho S. H., Kim S. H., Chang S. (2012). J. Am. Chem. Soc..

[cit18] Park S. H., Kwak J., Shin K., Ryu J., Park Y., Chang S. (2014). J. Am. Chem. Soc..

[cit19] Sun K., Li Y., Xiong T., Zhang J., Zhang Q. (2011). J. Am. Chem. Soc..

[cit20] Van Vliet K. M., Polak L. H., Siegler M. A., Van Der Vlugt J. I., Guerra C. F., De Bruin B. (2019). J. Am. Chem. Soc..

[cit21] Goswami M., Lyaskovskyy V., Domingos S. R., Buma W. J., Woutersen S., Troeppner O., Ivanović-Burmazović I., Lu H., Cui X., Zhang X. P., Reijerse E. J., Debeer S., Van Schooneveld M. M., Pfaff F. F., Ray K., De Bruin B. (2015). J. Am. Chem. Soc..

[cit22] Hennessy E. T., Betley T. A. (2013). Science.

[cit23] Taylor R. D., Maccoss M., Lawson A. D. G. (2014). J. Med. Chem..

[cit24] Vitaku E., Smith D. T., Njardarson J. T. (2014). J. Med. Chem..

[cit25] Bagh B., Broere D. L. J., Sinha V., Kuijpers P. F., Van Leest N. P., De Bruin B., Demeshko S., Siegler M. A., Van Der Vlugt J. I. (2017). J. Am. Chem. Soc..

[cit26] Shing K. P., Liu Y., Cao B., Chang X. Y., You T., Che C. M. (2018). Angew. Chem., Int. Ed..

[cit27] Du Y. D., Xu Z. J., Zhou C. Y., Che C. M. (2019). Org. Lett..

[cit28] Du Y. D., Zhou C. Y., To W. P., Wang H. X., Che C. M. (2020). Chem. Sci..

[cit29] Liang S., Zhao X., Yang T., Yu W. (2020). Org. Lett..

[cit30] You T., Zeng S. H., Fan J., Wu L., Kang F., Liu Y., Che C. M. (2021). Chem. Commun..

[cit31] Stroek W., Keilwerth M., Pividori D. M., Meyer K., Albrecht M. (2021). J. Am. Chem. Soc..

[cit32] Thacker N. C., Lin Z., Zhang T., Gilhula J. C., Abney C. W., Lin W. (2016). J. Am. Chem. Soc..

[cit33] Iovan D. A., Wilding M. J. T., Baek Y., Hennessy E. T., Betley T. A. (2017). Angew. Chem., Int. Ed..

[cit34] Wu L., Zhong D., Liu W. (2021). Chin. J. Org. Chem..

[cit35] Kuijpers P. F., Tiekink M. J., Breukelaar W. B., Broere D. L. J., van Leest N. P., van der Vlugt J. I., Reek J. N. H., de Bruin B. (2017). Chem. - Eur. J..

[cit36] Goswami M., Geuijen P., Reek J. N. H., de Bruin B. (2018). Eur. J. Inorg. Chem..

[cit37] Baek Y., Hennessy E. T., Betley T. A. (2019). J. Am. Chem. Soc..

[cit38] Baek Y., Betley T. A. (2019). J. Am. Chem. Soc..

[cit39] Baek Y., Das A., Zheng S. L., Reibenspies J. H., Powers D. C., Betley T. A. (2020). J. Am. Chem. Soc..

[cit40] Dong Y., Clarke R. M., Porter G. J., Betley T. A. (2020). J. Am. Chem. Soc..

[cit41] Dong Y., Lund C. J., Porter G. J., Clarke R. M., Zheng S. L., Cundari T. R., Betley T. A. (2021). J. Am. Chem. Soc..

[cit42] Qin J., Zhou Z., Cui T., Hemming M., Meggers E. (2019). Chem. Sci..

[cit43] Zhou Z., Chen S., Qin J., Nie X., Zheng X., Harms K., Riedel R., Houk K. N., Meggers E. (2019). Angew. Chem., Int. Ed..

[cit44] Broere D. L. J., De Bruin B., Reek J. N. H., Lutz M., Dechert S., Van Der Vlugt J. I. (2014). J. Am. Chem. Soc..

[cit45] Broere D. L. J., Van Leest N. P., De Bruin B., Siegler M. A., Van Der Vlugt J. I. (2016). Inorg. Chem..

[cit46] Keijer T., Bakker V., Slootweg J. C. (2019). Nat. Chem..

[cit47] Andersen R. A., Faegri K., Green J. C., Haaland A., Lappert M. F., Leung W., Rypdal K. (1988). Inorg. Chem..

[cit48] Broere D. L. J., Čorić I., Brosnahan A., Holland P. L. (2017). Inorg. Chem..

[cit49] Addition of Boc_2_O to a solution of Fe(HMDS)_2_ results in an immediate color change to brown and formation of a black precipitate, indicating decomposition to a catalytically inactive species

[cit50] Ben-Tal Y., Boaler P. J., Dale H. J. A., Dooley R. E., Fohn N. A., Gao Y., García-Domínguez A., Grant K. M., Hall A. M. R., Hayes H. L. D., Kucharski M. M., Wei R., Lloyd-Jones G. C. (2022). Prog. Nucl. Magn. Reson. Spectrosc..

[cit51] Walsh D. J., Lau S. H., Hyatt M. G., Guironnet D. (2017). J. Am. Chem. Soc..

[cit52] Lee C., Yang W., Parr R. G. (1988). Phys. Rev. B: Condens. Matter Mater. Phys..

[cit53] Becke A. D. (1993). J. Chem. Phys..

[cit54] Weigend F., Häser M., Patzelt H., Ahlrichs R. (1998). Chem. Phys. Lett..

[cit55] Weigend F., Ahlrichs R. (2005). Phys. Chem. Chem. Phys..

[cit56] Neese F. (2012). Wiley Interdiscip. Rev.: Comput. Mol. Sci..

[cit57] Neese F. (2018). Wiley Interdiscip. Rev.: Comput. Mol. Sci..

[cit58] Grant L. N., Carroll M. E., Carroll P. J., Mindiola D. J. (2016). Inorg. Chem..

[cit59] Baik M. H., Friesner R. A., Lippard S. J. (2002). J. Am. Chem. Soc..

[cit60] Ryu H., Park J., Kim H. K., Park J. Y., Kim S. T., Baik M. H. (2018). Organometallics.

[cit61] Wilding M. J. T., Iovan D. A., Wrobel A. T., Lukens J. T., Macmillan S. N., Lancaster K. M., Betley T. A. (2017). J. Am. Chem. Soc..

[cit62] Angeli C., Cimiraglia R., Evangelisti S., Leininger T., Malrieu J.-P. (2001). J. Chem. Phys..

[cit63] Angeli C., Cimiraglia R., Malrieu J.-P. (2001). Chem. Phys. Lett..

[cit64] Angeli C., Cimiraglia R., Malrieu J.-P. (2002). J. Chem. Phys..

[cit65] Anneser M. R., Elpitiya G. R., Townsend J., Johnson E. J., Powers X. B., DeJesus J. F., Vogiatzis K. D., Jenkins D. M. (2019). Angew. Chem., Int. Ed..

[cit66] Thomas C. M., Mankad N. P., Peters J. C. (2006). J. Am. Chem. Soc..

[cit67] Searles K., Fortier S., Khusniyarov M. M., Carroll P. J., Sutter J., Meyer K., Mindiola D. J., Caulton K. G. (2014). Angew. Chem., Int. Ed..

[cit68] Zhang H., Ouyang Z., Liu Y., Zhang Q., Wang L., Deng L. (2014). Angew. Chem., Int. Ed..

[cit69] Wang L., Hu L., Zhang H., Chen H., Deng L. (2015). J. Am. Chem. Soc..

[cit70] Nieto I., Ding F., Bontchev R. P., Wang H., Smith J. M. (2008). J. Am. Chem. Soc..

[cit71] Jacobs B. P., Wolczanski P. T., Jiang Q., Cundari T. R., MacMillan S. N. (2017). J. Am. Chem. Soc..

[cit72] Iovan D. A., Betley T. A. (2016). J. Am. Chem. Soc..

[cit73] Luo Y.-R. , Comprehensive Handbook of Chemical Bond Energies, CRC Press, Taylor & Francis Group, Boca Raton, 2007

