# Supplementary material for: Discovery of a simple iron catalyst reveals the intimate steps of C–H amination to form C–N bonds
Source: Chem Sci. 2022 Dec 28;14(11):2849–59. doi: 10.1039/d2sc04170g (PMC10016609; doi:10.1039/d2sc04170g)
Supplement: SC-014-D2SC04170G-s001 [file SC-014-D2SC04170G-s001.pdf]

Electronic Supplementary Material (ESI) for Chemical Science.
This journal is © The Royal Society of Chemistry 2022

Electronic Supporting Information

Discovery of a Simple Iron Catalyst Reveals the Intimate Steps of C–H Amination to Form C–N Bonds

Wowa Stroek[1] and Martin Albrecht[1,*]

[1]Department of Chemistry, Biochemistry and Pharmaceutical Sciences, University of Bern, CH-3012 Bern, Switzerland

Table of Contents

General consideration S3

$Fe(HMDS)_2$ synthesis S6

Substrate synthesis S6

Catalysis S21

General procedure S21

Characterization of substrates S22

Kinetics S26

General procedure S26

Variation of substrate concentration S26

Variation of catalyst concentration S28

Product inhibition studies S30

Catalyst decomposition studies S31

Intermolecular competition Kinetic isotope effect S32

Intermolecular Kinetic isotope effect S33

Stoichiometric experiments S35

NMR spectroscopy S35

FTIR-spectroscopy S39

Computational details S41

Density functional theory S41

NEVPT2-CASSCF S44

DFT calculated structures S47

DFT calculated coordinates S52

NMR spectra S76

$Fe(HMDS)_2$ S76

Substrate synthesis S76

Catalysis S109

Crystallographic and refinement data S116

References S117

General considerations

Chemicals, solvents and synthesis

All manipulations involving transition metal complexes were performed inside an argon filled MBraun glovebox with <0.1 O_2 and H_2O levels using dry and degassed solvents, unless stated otherwise. Benzene, hexane and diethylether were taken from a MBraun SPS system, degassed by three freeze-pump-thaw cycles and dried over 4 Å molecular sieves prior to use. THF-d_8, C_6D_6 and toluene-d_8 were distilled over NaK, degassed by three freeze-pump-thaw cycles and dried over 4 Å molecular sieves. Molecular sieves were pre-dried in a 1000W microwave for 10 min, in 30 s intervals. After which they were dried under vacuum at 220 °C for 7 days.

All organic synthesis was performed under aerobic conditions with commercially available solvents, unless stated otherwise. All other chemicals were used as received from commercial sources.

NMR-spectroscopy

All ^{1}H and ^{13}C NMR spectra were recorded on a Bruker AVANCE III HD 300. The chemical shifts are reported relative to $SiMe_4$ using the chemical shift of residual solvent peaks as reference.[S1]

FT-IR spectroscopy

FTIR-spectroscopy was measured in solution using the ReactIR 15 by Mettler Toledo using a fiber probe inserted into a Schlenk under argon atmosphere. The spectra were obtained with a 1 cm^{-1} resolution.

Density functional theory

All calculations were performed using the Orca 5.0.1 software package[S2,3] on the full atomic models (no substitutions of bulky groups to reduce computational time). All geometry optimizations (!Opt keyword) and transition state searched (!OptTS keyword) were performed on the B3LYP[S4,5] / def2-TZVP[S6,7] level of theory together with the RIJCOSX[S8] approximation in conjunction with the def2/J[S9] fitting the basis set to reduce computational time. Empirical dispersion corrections were including in all calculations with Grimme's DFT-D3 method[S10] (!D3BJ keyword). Numerical precision for the SCF convergence was set at an energy change of $1.0e^{-08}$ au (!TightSCF keyword). Convergence to an energy minima for was confirmed by performing a frequency analysis (!Freq keyword) and no imaginary frequencies were found.[S11] Convergence to an energy saddle point was confirmed by one imaginary frequency after the frequency calculation. The negative frequency was visualized using the Chemcraft software package[S12] to confirm the frequency represents the reaction coordinate of the transition state. Prior to transition state search calculations, the geometry was estimated by performing a Relaxed Surface Scan on the BP86[S5,13,14] / def2-SVP[S6,7] level of theory together with the RI-J[S15] approximation in conjunction with the def2/J[S9] fitting the basis set to reduce computational time. The geometry of the highest energy point was used for the prior described transition state search.

NEVPT2-CASSCF

All calculations were performed using the Orca 5.0.1 software package.[S2,3] Single point calculations on the DFT optimized structures were performed to generate quasi-restricted orbitals (QRO's) as initial guess orbitals for the CASSCF calculations (!UNO keyword). The B3LYP[S4,5] functional with the def2-TZVP[S6,7] basis set was used together with the RIJCOSX[S8] approximation in conjunction with the def2-TZVP/C[S16] fitting the basis set to reduce computational cost. The obtained QRO's were inspected and selected for the active space using the Avogadro software package.[S17] If necessary orbitals were rotated into the active space (%scf rotate{orbital 1,orbital 2,90} end end) and inspected again. Next a single root CASSCF calculation was performed using the guess orbitals (!MOREAD keyword) using the def2-TZVP[S6,7] basis set together with the RIJCOSX[S8] approximation in conjunction with the def2-TZVP/C[S16] fitting the basis set to reduce computational time. After convergence the active space orbitals were inspected to be the orbitals of interest. If necessary, orbitals of interest outside the active space were rotated in the active space and the CASSCF calculation was repeated until all orbitals of interest were converged into the active space. Lastly, a NEVPT2[S18–20] correction was performed (!SOMF(1X) RI-NEVPT2 keyword) on the correctly converged CASSCF orbitals (!MOREAD keyword). Orbital images were generated using the IboView software package.[S21]

Single crystal X-ray diffraction

All crystals were measured on an Oxford Diffraction SuperNova area-detector diffractometer[S22] using mirror optics monochromated Mo *K*α radiation (λ = 0.71073 Å) and Al filtered.[S23]

Data reduction was performed using the *CrysAlisPro*[S22] program. The intensities were corrected for Lorentz and polarization effects, and an absorption correction based on the multi-scan method using SCALE3 ABSPACK in *CrysAlisPro*[S22] was applied.

The structures were solved by direct methods using *SHELXT*,[S24] which revealed the positions of all non-hydrogen atoms of the title compounds. All non-hydrogen atoms were refined anisotropically. H-atoms were assigned in geometrically calculated positions and refined using a riding model where each H-atom was assigned a fixed isotropic displacement parameter with a value equal to 1.2Ueq of its parent atom (1.5 Ueq for methyl groups), except for those attached to N atoms, where the H atoms were located from the map but refined within the riding model as described above.

Refinement of the structures was carried out on F^2 using full-matrix least-squares procedures, which minimized the function $\Sigma w(F_o^2 - F_c^2)^2$. The weighting scheme was based on counting statistics and included a factor to downweight the intense reflections. All calculations were performed using the *SHELXL-2014/7*[S24] program in OLEX2.[S25] Further crystallographic details are compiled in table S6 and Figure S118. Crystallographic data for all structures have been deposited with the Cambridge Crystallographic Data entre (CCDC) as supplementary publication number 2171951 (**4**).

Disorder model was used for parts of the structure where the occupancies of each disordered part was refined through the use of a free variable. A solvent mask was used which would include the contribution of electron densities from void areas into the calculated structure factors, however the total electron count found in the voids are zero. The void mask was kept only to suppress checkcif alerts.

Fe(HMDS)$_2$ synthesis

Synthesized according to a literature procedure.[S26] An oven dried Schlenk was charged with $FeBr_2$ (10.78 g; 50.00 mmol; 1.00 eq) and LiHMDS (16.73 g; 100.00 mmol; 2.00 eq) under an argon atmosphere. Next Et_2O (200 mL) was added at 0 °C, the white suspension was stirred at room temperature for 2 days. The obtained brown suspension was concentrated to dryness. The solid was extracted with hexane (1x 200 mL) and filtered over Celite, the filter was washed with hexane (20 mL). The filtrate was concentrated to dryness to obtain a green slurry which was distilled under active vacuum (approximately 0.01 mbar) at 80 °C to obtain a green oil as the final product (17.00 g; 45.00 mmol; 90%). The product was stored as a solid in the freezer at -30 °C in an argon filled glovebox.

Spectral data were consistent with previously reported characterization of the product.[S26,27]
^{1}H NMR (300 MHz, C_6D_6) δ 63.07 (s, 36H).

Substrate synthesis

General procedure

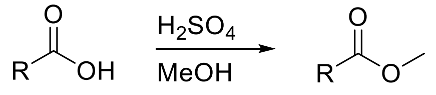

Synthesized according to a literature procedure.[S28] Corresponding carboxylic acid was dissolved in MeOH and 10 drops of concentrated sulphuric acid were added. The solution was stirred for 16 h and concentrated under reduced pressure. Water was added and the emulsion was extracted with Et_2O, washed with brine, dried over Na_2SO_4, filtered and concentrated to obtain the corresponding ester as the product.

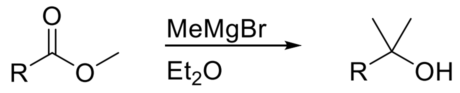

Synthesized according to a literature procedure.[S28] In an oven dried Schlenk under an argon atmosphere corresponding ester (1.0 eq) was dissolved in anhydrous Et_2O and cooled to 0 °C. A solution of 3.0 M MeMgBr (3.0 eq) in Et_2O was added dropwise and the obtained white suspension was stirred for 16 h. The mixture was quenched with concentrated aqueous NH_4Cl solution and extracted with Et_2O, washed with brine, dried over Na_2SO_4, filtered and concentrated to obtain the corresponding alcohol as the product.

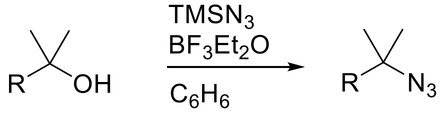

Synthesized according to a literature procedure.[S28] In an oven dried Schlenk under an argon atmosphere corresponding alcohol (1.0 eq) and $TMSN_3$ (1.2 eq) was dissolved in anhydrous

C_6H_6. BF_3Et_2O (1.2 eq) was added dropwise and the solution was stirred for 16 h. The obtained mixture was quenched with water, extracted with Et_2O, washed with brine, dried over Na_2SO_4, filtered and concentrated. The crude product was purified by column chromatography over SiO_2 using hexane as eluent.

All azide products were transferred into a J Young Schlenk, degassed by four freeze-pump-thaw cycles and dried over 4 Å molecular sieves for at least one week before use in catalysis.

Substrate **1a**

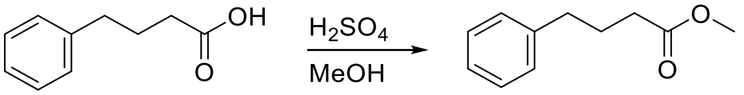

Synthesized according to a literature procedure.[S29] 4-phenylbutanoic acid (50.0 g; 304.5 mmol; 1.0 eq) was dissolved in MeOH (500 mL) and 10 drops of concentrated sulphuric acid were added. The solution was stirred for 16 h and concentrated under reduced pressure. Water (100 mL) was added and the emulsion was extracted with Et_2O (3x 250 mL), washed with brine (100 mL), dried over Na_2SO_4, filtered and concentrated. The product was obtained as a colorless oil (52.11 g; 292.4 mmol; 96%).

Spectral data were consistent with previously reported characterization of the product.[S29] ^1H NMR (300 MHz, CD_2Cl_2) δ 7.32 – 7.23 (m, 2H), 7.23 – 7.05 (m, 3H), 3.64 (s, 3H), 2.64 (dd, *J* = 8.5, 6.8 Hz, 2H), 2.32 (t, *J* = 7.5 Hz, 2H), 1.93 (p, *J* = 7.5 Hz, 2H).

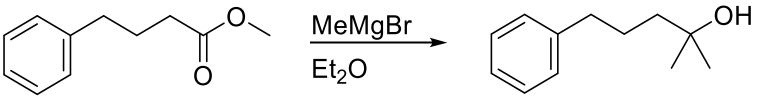

Synthesized according to a literature procedure.[S29] In an oven dried Schlenk under an argon atmosphere methyl 4-phenylbutanoate (52.0 g; 291.8 mmol; 1.0 eq) was dissolved in anhydrous Et_2O (300 mL) and cooled to 0 °C. A solution of 3.0 M MeMgBr (292 mL; 875.3 mmol; 3.0 eq) in Et_2O was added dropwise and the obtained white suspension was stirred for 16 h. The mixture was quenched with concentrated aqueous NH_4Cl (200 mL) solution and extracted with Et_2O (5x 250 mL), washed with brine (100 mL), dried over Na_2SO_4, filtered and concentrated. The product was obtained as a colorless oil (38.91 g; 218.3 mmol; 75%).

Spectral data were consistent with previously reported characterization of the product.[S29] ^1H NMR (300 MHz, $CDCl_3$) δ 7.26 – 7.15 (m, 2H), 7.15 – 7.07 (m, 3H), 2.54 (t, *J* = 7.6 Hz, 2H), 1.70 – 1.54 (m, 2H), 1.49 – 1.37 (m, 2H), 1.12 (s, 6H).

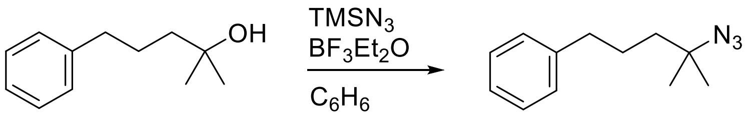

Synthesized according to a literature procedure.[S29] In an oven dried Schlenk under an argon atmosphere 2-methyl-5-phenylpentan-2-ol (30.0 g; 168.3 mmol; 1.0 eq) and $TMSN_3$ (26.8 mL; 201.9 mmol; 1.2 eq) was dissolved in anhydrous C_6H_6 (500 mL). BF_3Et_2O (24.9 mL; 201.9 mmol; 1.2 eq) was added dropwise and the solution was stirred for 16 h. The obtained mixture was quenched with water (200 mL), extracted with Et_2O (3x 250 mL), washed with brine (100 mL), dried over Na_2SO_4, filtered and concentrated. The crude product was purified by column chromatography over SiO_2 using hexane as eluent. The product was obtained as a colorless oil (13.55 g; 66.7 mmol; 40%).

Spectral data were consistent with previously reported characterization of the product.[S29] ^{1}H NMR (300 MHz, $CDCl_3$) δ 7.42 – 7.31 (m, 2H), 7.31 – 7.20 (m, 3H), 2.70 (t, *J* = 7.5 Hz, 2H), 1.89 – 1.67 (m, 2H), 1.67 – 1.42 (m, 2H), 1.33 (s, 6H). ^{13}C NMR (75 MHz, $CDCl_3$) δ 142.17, 128.50, 126.01, 61.71, 41.19, 36.18, 26.24, 26.13.

<u>Substrate **9a**</u>

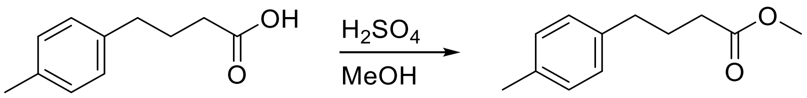

Synthesized according to a literature procedure.[S30] 4-(p-tolyl)butanoic acid (8.00 g; 44.89 mmol; 1.0 eq) was dissolved in MeOH (100 mL) and 10 drops of concentrated sulphuric acid were added. The solution was stirred for 16 h and concentrated under reduced pressure. Water (100 mL) was added and the emulsion was extracted with Et_2O (3x 100 mL), washed with brine (100 mL), dried over Na_2SO_4, filtered and concentrated. The product was obtained as a colorless oil (7.80 g; 40.6 mmol; 90%).

Spectral data were consistent with previously reported characterization of the product.[S30] ^{1}H NMR (300 MHz, $CDCl_3$) δ 7.05 – 6.92 (m, 4H), 3.57 (s, 3H), 2.52 (t, *J* = 7.6 Hz, 2H), 2.23 (d, *J* = 2.7 Hz, 5H), 1.84 (p, *J* = 7.6 Hz, 2H). ^{13}C NMR (75 MHz, $CDCl_3$) δ 174.13, 138.41, 135.55, 129.19, 128.49, 51.62, 34.81, 33.53, 26.73, 21.12.

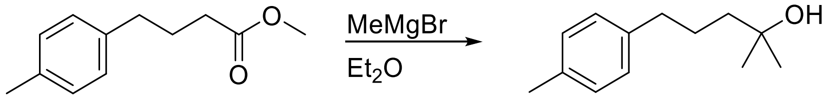

Synthesized according to a literature procedure.[S29] In an oven dried Schlenk under an argon atmosphere methyl 4-(p-tolyl)butanoate (7.79 g; 40.52 mmol; 1.0 eq) was dissolved in anhydrous Et_2O (200 mL) and cooled to 0 °C. A solution of 3.0 M MeMgBr (40.5 mL; 121.6 mmol; 3.0 eq) in Et_2O was added dropwise and the obtained white suspension was stirred for 16 h. The mixture was quenched with concentrated aqueous NH_4Cl (25 mL) solution and extracted with Et_2O (3x 100 mL), washed with brine (100 mL), dried over Na_2SO_4, filtered and concentrated. The product was obtained as a colorless oil (7.00 g; 36.4 mmol; 90%).

Spectral data were consistent with previously reported characterization of the product.[S29] ^{1}H NMR (300 MHz, $CDCl_3$) δ 7.10 (s, 4H), 2.59 (t, *J* = 7.5 Hz, 2H), 2.33 (s, 3H), 1.77 – 1.60 (m, 2H),

1.57 – 1.45 (m, 2H), 1.31 (s, 1H), 1.21 (s, 6H). ^{13}C NMR (75 MHz, $CDCl_3$) δ 139.47, 135.28, 129.12, 128.40, 71.08, 43.64, 36.01, 29.36, 26.52, 21.12.

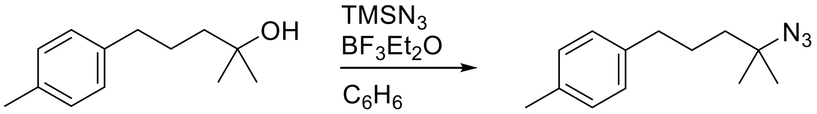

Synthesized according to a literature procedure.[S29] In an oven dried Schlenk under an argon atmosphere 2-methyl-5-(p-tolyl)pentan-2-ol (6.44 g; 33.5 mmol; 1.0 eq) and $TMSN_3$ (5.3 mL; 40.2 mmol; 1.2 eq) was dissolved in anhydrous C_6H_6 (200 mL). BF_3Et_2O (4.96 mL; 40.2 mmol; 1.2 eq) was added dropwise and the solution was stirred for 16 h. The obtained mixture was quenched with water (100 mL), extracted with Et_2O (3x 100 mL), washed with brine (100 mL), dried over Na_2SO_4, filtered and concentrated. The crude product was purified by column chromatography over SiO_2 using hexane as eluent. The product was obtained as a colorless oil (1.77 g; 8.1 mmol; 24%).

Spectral data were consistent with previously reported characterization of the product.[S29] ^{1}H NMR (300 MHz, $CDCl_3$) δ 7.15 – 7.03 (m, 4H), 2.59 (t, *J* = 7.5 Hz, 2H), 2.33 (s, 3H), 1.76 – 1.56 (m, 2H), 1.56 – 1.41 (m, 2H), 1.25 (s, 6H). ^{13}C NMR (75 MHz, $CDCl_3$) δ 139.09, 135.44, 129.19, 128.37, 61.73, 41.20, 35.73, 26.36, 26.12, 21.14.

<u>Substrate **10a**</u>

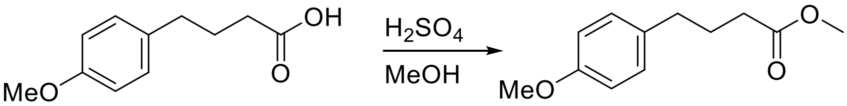

Synthesized according to a literature procedure.[S29] 4-(4-methoxyphenyl)butanoic acid (8.00 g; 41.2 mmol; 1.0 eq) was dissolved in MeOH (100 mL) and 10 drops of concentrated sulphuric acid were added. The solution was stirred for 16 h and concentrated under reduced pressure. Water (100 mL) was added and the emulsion was extracted with Et_2O (3x 100 mL), washed with brine (100 mL), dried over Na_2SO_4, filtered and concentrated. The product was obtained as a colorless oil (8.12 g; 39.0 mmol; 95%).

Spectral data were consistent with previously reported characterization of the product.[S29] ^{1}H NMR (300 MHz, $CDCl_3$) δ 7.14 – 7.05 (m, 2H), 6.87 – 6.78 (m, 2H), 3.79 (s, 3H), 3.66 (s, 3H), 2.59 (t, *J* = 7.6 Hz, 2H), 2.32 (t, *J* = 7.5 Hz, 2H), 2.00 – 1.87 (m, 2H). ^{13}C NMR (75 MHz, $CDCl_3$) δ 174.15, 158.03, 133.57, 129.51, 113.93, 55.39, 51.63, 34.34, 33.48, 26.86.

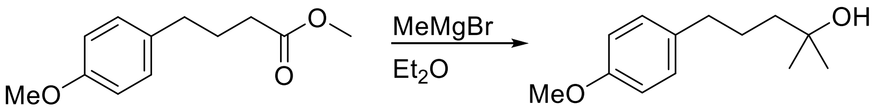

Synthesized according to a literature procedure.[S29] In an oven dried Schlenk under an argon atmosphere methyl 4-(4-methoxyphenyl)butanoate (8.07 g; 38.8 mmol; 1.0 eq) was dissolved in anhydrous Et_2O (200 mL) and cooled to 0 °C. A solution of 3.0 M MeMgBr (38.8 mL; 116.3

mmol; 3.0 eq) in Et_2O was added dropwise and the obtained white suspension was stirred for 16 h. The mixture was quenched with concentrated aqueous NH_4Cl (50 mL) solution and extracted with Et_2O (3x 100 mL), washed with brine (100 mL), dried over Na_2SO_4, filtered and concentrated. The product was obtained as a colorless oil (7.34 g; 35.2 mmol; 91%).

Spectral data were consistent with previously reported characterization of the product.[S29] ^1H NMR (300 MHz, $CDCl_3$) δ 7.15 – 7.06 (m, 2H), 6.87 – 6.78 (m, 2H), 3.79 (s, 3H), 2.57 (t, J = 7.5 Hz, 2H), 1.75 – 1.55 (m, 2H), 1.54 – 1.44 (m, 2H), 1.38 (d, J = 14.1 Hz, 1H), 1.20 (s, 6H). ^{13}C NMR (75 MHz, $CDCl_3$) δ 157.85, 134.67, 129.39, 113.86, 71.09, 55.39, 43.58, 35.54, 29.38, 26.63.

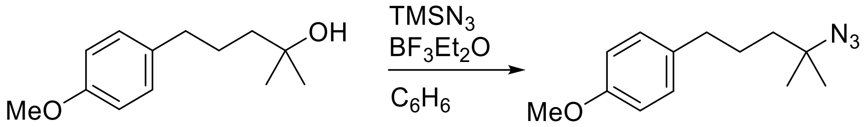

Synthesized according to a literature procedure.[S29] In an oven dried Schlenk under an argon atmosphere 5-(4-methoxyphenyl)-2-methylpentan-2-ol (6.88 g; 33.0 mmol; 1.0 eq) and $TMSN_3$ (5.3 mL; 39.6 mmol; 1.2 eq) was dissolved in anhydrous C_6H_6 (200 mL). BF_3Et_2O (4.9 mL; 39.6 mmol; 1.2 eq) was added dropwise and the solution was stirred at 60 °C for 40 h. The obtained mixture was quenched with water (100 mL), extracted with Et_2O (3x 100 mL), washed with brine (100 mL), dried over Na_2SO_4, filtered and concentrated. The crude product was purified by column chromatography over SiO_2 using hexane as eluent. The product was obtained as a colorless oil (0.93 g; 4.0 mmol; 12%).

Spectral data were consistent with previously reported characterization of the product.[S29] ^1H NMR (300 MHz, $CDCl_3$) δ 7.14 – 7.05 (m, 2H), 6.88 – 6.79 (m, 2H), 3.79 (s, 3H), 2.56 (t, J = 7.4 Hz, 2H), 1.72 – 1.57 (m, 2H), 1.56 – 1.46 (m, 2H), 1.24 (s, 6H). ^{13}C NMR (75 MHz, $CDCl_3$) δ 157.94, 134.26, 129.37, 113.92, 61.73, 55.40, 41.13, 35.25, 26.47, 26.13.

Substrate **11a**

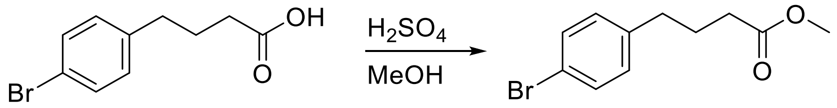

Synthesized according to a literature procedure.[S30] 4-(4-bromophenyl)butanoic acid (20.00 g; 82.3 mmol; 1.0 eq) was dissolved in MeOH (300 mL) and 10 drops of concentrated sulphuric acid were added. The solution was stirred for 16 h and concentrated under reduced pressure. Water (200 mL) was added and the emulsion was extracted with Et_2O (3x 200 mL), washed with brine (100 mL), dried over Na_2SO_4, filtered and concentrated. The product was obtained as a colorless oil (19.54 g; 76.0 mmol; 92%).

Spectral data were consistent with previously reported characterization of the product.[S30] ^1H NMR (300 MHz, $CDCl_3$) δ 7.44 – 7.35 (m, 2H), 7.09 – 7.00 (m, 2H), 3.66 (d, J = 1.2 Hz, 3H), 2.60 (t, J = 7.6 Hz, 2H), 2.31 (t, J = 7.4 Hz, 2H), 2.01 – 1.87 (m, 2H). ^{13}C NMR (75 MHz, CDCl3) δ 173.86, 140.43, 131.56, 130.36, 119.87, 51.68, 34.61, 33.33, 26.41.

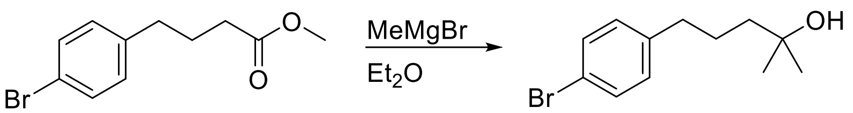

Synthesized according to a literature procedure.[S31] In an oven dried Schlenk under an argon atmosphere methyl 4-(4-bromophenyl)butanoate (19.54 g; 72.06 mmol; 1.0 eq) was dissolved in anhydrous Et_2O (400 mL) and cooled to 0 °C. A solution of 3.0 M MeMgBr (72.1 mL; 216.2 mmol; 3.0 eq) in Et_2O was added dropwise and the obtained white suspension was stirred for 16 h. The mixture was quenched with concentrated aqueous NH_4Cl (100 mL) solution and extracted with Et_2O (3x 200 mL), washed with brine (100 mL), dried over Na_2SO_4, filtered and concentrated. The product was obtained as a colorless oil (16.77 g; 61.8 mmol; 86%).

Spectral data were consistent with previously reported characterization of the product.[S31] ^{1}H NMR (300 MHz, $CDCl_3$) δ 7.44 – 7.35 (m, 2H), 7.11 – 7.01 (m, 2H), 2.58 (td, *J* = 7.6, 2.0 Hz, 2H), 1.76 – 1.59 (m, 2H), 1.54 – 1.43 (m, 2H), 1.28 (d, *J* = 3.2 Hz, 1H), 1.20 (d, *J* = 2.0 Hz, 6H). ^{13}C NMR (75 MHz, $CDCl_3$) δ 141.48, 131.47, 130.31, 119.58, 70.99, 43.40, 35.82, 29.43, 26.17.

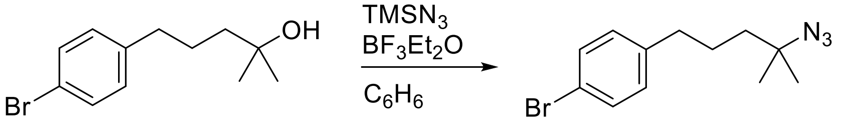

Synthesized according to a literature procedure.[S32] In an oven dried Schlenk under an argon atmosphere 5-(4-bromophenyl)-2-methylpentan-2-ol (4.27 g; 16.6 mmol; 1.0 eq) and $TMSN_3$ (2.6 mL; 19.9 mmol; 1.2 eq) was dissolved in anhydrous C_6H_6 (200 mL). BF_3Et_2O (2.5 mL; 19.9 mmol; 1.2 eq) was added dropwise and the solution was stirred for 16 h. The obtained mixture was quenched with water (100 mL), extracted with Et_2O (3x 100 mL), washed with brine (100 mL), dried over Na_2SO_4, filtered and concentrated. The crude product was purified by column chromatography over SiO_2 using hexane as eluent. The product was obtained as a colorless oil (2.55 g; 9.0 mmol; 54%).

Spectral data were consistent with previously reported characterization of the product.[S32] ^{1}H NMR (300 MHz, $CDCl_3$) δ 7.44 – 7.35 (m, 2H), 7.11 – 7.01 (m, 2H), 2.57 (t, *J* = 7.5 Hz, 2H), 1.74 – 1.59 (m, 2H), 1.53 – 1.45 (m, 2H), 1.24 (s, 6H). ^{13}C NMR (75 MHz, $CDCl_3$) δ 141.08, 131.56, 130.27, 119.74, 61.60, 41.05, 35.52, 26.13, 26.02.

<u>Substrate **12a**</u>

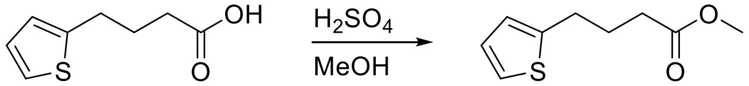

Synthesized according to a literature procedure.[S33] 4-(thiophen-2-yl)butanoic acid (8.05 g; 47.3 mmol; 1.0 eq) was dissolved in MeOH (100 mL) and 10 drops of concentrated sulphuric acid were added. The solution was stirred for 16 h and concentrated under reduced pressure. Water (100 mL) was added and the emulsion was extracted with Et_2O (3x 100 mL), washed with brine (100 mL), dried over Na_2SO_4, filtered and concentrated. The product was obtained as a brown oil (8.00 g; 43.4 mmol; 92%).

Spectral data were consistent with previously reported characterization of the product.[S33] ^{1}H NMR (300 MHz, $CDCl_3$) δ 7.12 (dd, *J* = 5.1, 1.2 Hz, 1H), 6.92 (dd, *J* = 5.1, 3.4 Hz, 1H), 6.80 (dq, *J* = 3.3, 1.0 Hz, 1H), 3.68 (s, 3H), 2.97 – 2.82 (m, 2H), 2.38 (t, *J* = 7.4 Hz, 2H), 2.01 (p, *J* = 7.5 Hz, 2H).

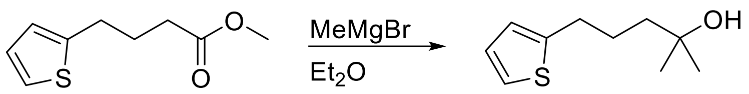

Synthesized according to a literature procedure.[S33] In an oven dried Schlenk under an argon atmosphere methyl 4-(thiophen-2-yl)butanoate (8.00 g; 43.4 mmol; 1.0 eq) was dissolved in anhydrous Et_2O (200 mL) and cooled to 0 °C. A solution of 3.0 M MeMgBr (43.4 mL; 130.3 mmol; 3.0 eq) in Et_2O was added dropwise and the obtained white suspension was stirred for 16 h. The mixture was quenched with concentrated aqueous NH_4Cl (50 mL) solution and extracted with Et_2O (3x 100 mL), washed with brine (100 mL), dried over Na_2SO_4, filtered and concentrated. The product was obtained as a brown oil (6.78 g; 36.8 mmol; 85%).

Spectral data were consistent with previously reported characterization of the product.[S33] ^{1}H NMR (300 MHz, $CDCl_3$) δ 7.11 (dd, *J* = 5.1, 1.2 Hz, 1H), 6.92 (dd, *J* = 5.1, 3.4 Hz, 1H), 6.79 (dq, *J* = 3.3, 1.0 Hz, 1H), 2.85 (td, *J* = 7.5, 1.0 Hz, 2H), 1.85 – 1.66 (m, 2H), 1.60 – 1.48 (m, 2H), 1.35 – 1.28 (m, 1H), 1.22 (s, 6H). ^{13}C NMR (75 MHz, $CDCl_3$) δ 145.47, 126.83, 124.25, 123.04, 71.01, 43.36, 30.43, 29.41, 26.78.

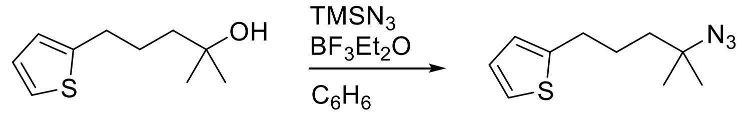

Synthesized according to a literature procedure.[S33] In an oven dried Schlenk under an argon atmosphere 2-methyl-5-(thiophen-2-yl)pentan-2-ol (6.67 g; 36.2 mmol; 1.0 eq) and $TMSN_3$ (5.8 mL; 43.4 mmol; 1.2 eq) was dissolved in anhydrous C_6H_6 (200 mL). BF_3Et_2O (5.4 mL; 43.4 mmol; 1.2 eq) was added dropwise and the solution was stirred for 16 h. The obtained mixture was quenched with water (100 mL), extracted with Et_2O (3x 100 mL), washed with brine (100 mL), dried over Na_2SO_4, filtered and concentrated. The crude product was purified by column chromatography over SiO_2 using hexane as eluent. The product was obtained as a colorless oil (1.40 g; 6.7 mmol; 18%).

Spectral data were consistent with previously reported characterization of the product.[S33] ^{1}H NMR (300 MHz, $CDCl_3$) δ 7.11 (dd, *J* = 5.1, 1.2 Hz, 1H), 6.92 (dd, *J* = 5.1, 3.4 Hz, 1H), 6.79 (dq, *J* = 3.3, 1.0 Hz, 1H), 2.85 (td, *J* = 7.5, 1.0 Hz, 2H), 1.85 – 1.66 (m, 2H), 1.60 – 1.48 (m, 2H), 1.35 – 1.28 (m, 1H), 1.22 (s, 6H). ^{13}C NMR (75 MHz, $CDCl_3$) δ 145.47, 126.83, 124.25, 123.04, 71.01, 43.36, 30.43, 29.41, 26.78.

Substrate **13a**

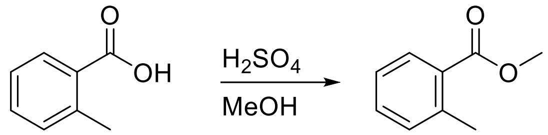

Synthesized according to a literature procedure.[S32] 2-methylbenzoic acid (8.00 g; 58.8 mmol; 1.0 eq) was dissolved in MeOH (100 mL) and 10 drops of concentrated sulphuric acid were added. The solution was stirred for 72 h at 60 °C and concentrated under reduced pressure. Water (100 mL) was added and the emulsion was extracted with Et_2O (3x 100 mL), washed with brine (100 mL), dried over Na_2SO_4, filtered and concentrated. The product was obtained as a colorless oil (8.14 g; 54.2 mmol; 92%).

Spectral data were consistent with previously reported characterization of the product.[S32] ^{1}H NMR (300 MHz, $CDCl_3$) δ 7.83 (dd, *J* = 8.1, 1.5 Hz, 1H), 7.32 (td, *J* = 7.5, 1.5 Hz, 1H), 7.23 – 7.10 (m, 2H), 3.81 (s, 3H), 2.53 (s, 3H). ^{13}C NMR (75 MHz, $CDCl_3$) δ 168.21, 140.30, 132.08, 131.80, 130.68, 129.69, 125.81, 51.92, 21.84.

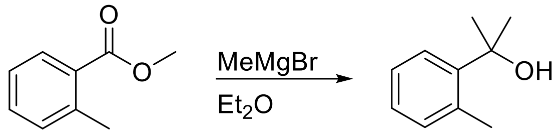

Synthesized according to a literature procedure.[S32] In an oven dried Schlenk under an argon atmosphere methyl 2-methylbenzoate (8.14 g; 54.2 mmol; 1.0 eq) was dissolved in anhydrous Et_2O (200 mL) and cooled to 0 °C. A solution of 3.0 M MeMgBr (54.2 mL; 162.6 mmol; 3.0 eq) in Et_2O was added dropwise and the obtained white suspension was stirred for 16 h. The mixture was quenched with concentrated aqueous NH_4Cl (50 mL) solution and extracted with Et_2O (3x 100 mL), washed with brine (100 mL), dried over Na_2SO_4, filtered and concentrated. The product was obtained as a colorless oil (7.28 g; 48.5 mmol; 89%).

Spectral data were consistent with previously reported characterization of the product.[S32] ^{1}H NMR (300 MHz, $CDCl_3$) δ 7.51 – 7.40 (m, 1H), 7.20 – 7.13 (m, 3H), 2.61 (s, 3H), 1.67 (s, 6H). ^{13}C NMR (75 MHz, $CDCl_3$) δ 145.85, 136.03, 132.78, 127.15, 125.76, 125.34, 73.79, 30.95, 22.33.

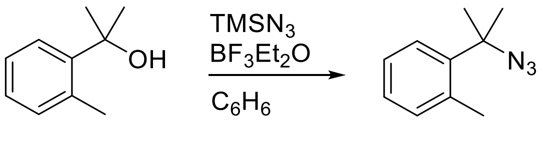

Synthesized according to a literature procedure.[S32] In an oven dried Schlenk under an argon atmosphere 2-(o-tolyl)propan-2-ol (7.14 g; 47.5 mmol; 1.0 eq) and $TMSN_3$ (7.6 mL; 57.0 mmol; 1.2 eq) was dissolved in anhydrous C_6H_6 (200 mL). BF_3Et_2O (7.0 mL; 57.0 mmol; 1.2 eq) was added dropwise and the solution was stirred for 16 h. The obtained mixture was quenched with water (100 mL), extracted with Et_2O (3x 100 mL), washed with brine (100 mL), dried over Na_2SO_4, filtered and concentrated. The crude product was purified by column chromatography over SiO_2 using hexane as eluent. The product was obtained as a colorless oil (3.60 g; 20.5 mmol; 43%).

Spectral data were consistent with previously reported characterization of the product.[S32] ^{1}H NMR (300 MHz, $CDCl_3$) δ 7.33 – 7.21 (m, 1H), 7.17 – 7.02 (m, 3H), 2.52 (s, 3H), 1.61 (s, 6H). ^{13}C NMR (75 MHz, $CDCl_3$) δ 141.22, 136.58, 133.01, 127.88, 126.04, 125.91, 64.57, 27.91, 21.68.

<u>Substrate **14a**</u>

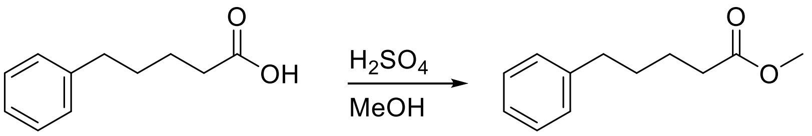

Synthesized according to a literature procedure.[S34] 5-phenylpentanoic acid (8.00 g; 44.9 mmol; 1.0 eq) was dissolved in MeOH (100 mL) and 10 drops of concentrated sulphuric acid were added. The solution was stirred for 16 h and concentrated under reduced pressure. Water (100 mL) was added and the emulsion was extracted with Et_2O (3x 100 mL), washed with brine (100 mL), dried over Na_2SO_4, filtered and concentrated. The product was obtained as a colorless oil (7.92 g; 41.2 mmol; 92%).

Spectral data were consistent with previously reported characterization of the product.[S34] ^{1}H NMR (300 MHz, $CDCl_3$) δ 7.25 – 7.16 (m, 2H), 7.15 – 7.06 (m, 3H), 3.59 (s, 3H), 2.62 – 2.49 (m, 2H), 2.34 – 2.20 (m, 2H), 1.69 – 1.49 (m, 4H). ^{13}C NMR (75 MHz, $CDCl_3$) δ 174.22, 142.26, 128.51, 128.45, 125.91, 51.62, 35.70, 34.08, 31.03, 24.72.

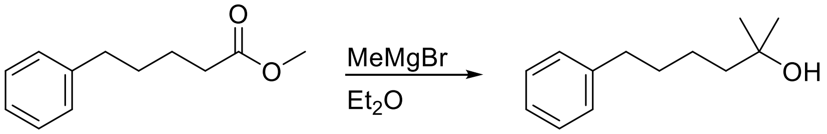

Synthesized according to a literature procedure.[S35] In an oven dried Schlenk under an argon atmosphere methyl 5-phenylpentanoate (7.92 g; 41.2 mmol; 1.0 eq) was dissolved in anhydrous Et_2O (200 mL) and cooled to 0 °C. A solution of 3.0 M MeMgBr (41.2 mL; 123.6 mmol; 3.0 eq) in Et_2O was added dropwise and the obtained white suspension was stirred for 16 h. The mixture was quenched with concentrated aqueous NH_4Cl (50 mL) solution and extracted with Et_2O (3x 100 mL), washed with brine (100 mL), dried over Na_2SO_4, filtered and concentrated. The product was obtained as a colorless oil (7.28 g; 37.9 mmol; 92%).

Spectral data were consistent with previously reported characterization of the product.[S35] ^{1}H NMR (300 MHz, $CDCl_3$) δ 7.28 – 7.16 (m, 2H), 7.16 – 6.99 (m, 3H), 2.56 (t, J = 8.1 Hz, 2H), 1.67 – 1.49 (m, 2H), 1.49 – 1.39 (m, 2H), 1.39 – 1.26 (m, 2H), 1.21 (s, 1H), 1.13 (s, 6H). ^{13}C NMR (75 MHz, $CDCl_3$) δ 142.77, 128.52, 128.41, 125.79, 71.14, 43.90, 36.09, 32.18, 29.39, 24.19.

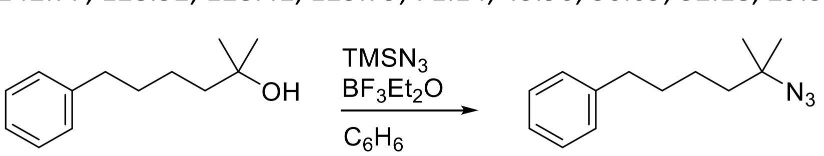

Synthesized according to a literature procedure.[S36] In an oven dried Schlenk under an argon atmosphere 2-methyl-6-phenylhexan-2-ol (7.23 g; 37.6 mmol; 1.0 eq) and $TMSN_3$ (6.0 mL; 45.1 mmol; 1.2 eq) was dissolved in anhydrous C_6H_6 (200 mL). BF_3Et_2O (5.6 mL; 45.1 mmol; 1.2 eq) was added dropwise and the solution was stirred for 16 h. The obtained mixture was

quenched with water (100 mL), extracted with Et_2O (3x 100 mL), washed with brine (100 mL), dried over Na_2SO_4, filtered and concentrated. The crude product was purified by column chromatography over SiO_2 using hexane as eluent. The product was obtained as a colorless oil (3.69 g; 17.0 mmol; 45%).

Spectral data were consistent with previously reported characterization of the product.[S36] ^{1}H NMR (300 MHz, $CDCl_3$) δ 7.26 – 7.15 (m, 2H), 7.15 – 7.05 (m, 3H), 2.55 (t, 2H), 1.62 – 1.49 (m, 2H), 1.49 – 1.40 (m, 2H), 1.40 – 1.26 (m, 2H), 1.17 (s, 6H). ^{13}C NMR (75 MHz, $CDCl_3$) δ 142.57, 128.50, 128.44, 125.85, 61.79, 41.44, 35.99, 31.89, 26.13, 24.10.

<u>Substrate **15a**</u>

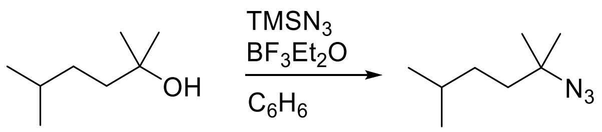

Synthesized according to a literature procedure.[S28] In an oven dried Schlenk under an argon atmosphere 2,5-dimethylhexan-2-ol (5.25 g; 40.3 mmol; 1.0 eq) and $TMSN_3$ (6.4 mL; 48.4 mmol; 1.2 eq) was dissolved in anhydrous C_6H_6 (200 mL). BF_3Et_2O (6.0 mL; 48.4 mmol; 1.2 eq) was added dropwise and the solution was stirred for 16 h. The obtained mixture was quenched with water (100 mL), extracted with Et_2O (3x 100 mL), washed with brine (100 mL), dried over Na_2SO_4, filtered and concentrated. The crude product was purified by column chromatography over SiO_2 using hexane as eluent. The product was obtained as a colorless oil (3.14 g; 20.2 mmol; 50%).

Spectral data were consistent with previously reported characterization of the product.[S28] ^{1}H NMR (300 MHz, $CDCl_3$) δ 1.58 – 1.42 (m, 3H), 1.30 – 1.16 (m, 8H), 0.90 (d, J = 6.6 Hz, 6H). ^{13}C NMR (75 MHz, $CDCl_3$) δ 61.88, 39.37, 33.34, 28.49, 26.14, 22.72.

<u>Substrate **16a**</u>

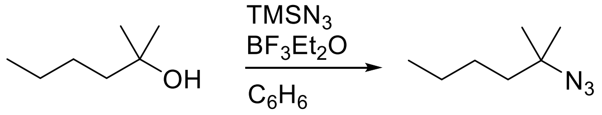

Synthesized according to a literature procedure.[S28] In an oven dried Schlenk under an argon atmosphere 2-methylhexan-2-ol (5.23 g; 45.0 mmol; 1.0 eq) and $TMSN_3$ (7.2 mL; 54.0 mmol; 1.2 eq) was dissolved in anhydrous C_6H_6 (200 mL). BF_3Et_2O (6.7 mL; 54.0 mmol; 1.2 eq) was added dropwise and the solution was stirred for 16 h. The obtained mixture was quenched with water (100 mL), extracted with Et_2O (3x 100 mL), washed with brine (100 mL), dried over Na_2SO_4, filtered and concentrated. The crude product was purified by column chromatography over SiO_2 using hexane as eluent. The product was obtained as a colorless oil (3.69 g; 26.1 mmol; 58%).

Spectral data were consistent with previously reported characterization of the product.[S28] ^{1}H NMR (300 MHz, $CDCl_3$) δ 1.54 – 1.42 (m, 2H), 1.42 – 1.27 (m, 4H), 1.25 (s, 6H), 0.99 – 0.84 (m, 3H). ^{13}C NMR (75 MHz, $CDCl_3$) δ 61.83, 41.32, 26.57, 26.13, 23.15, 14.16.

Substrate **17a**

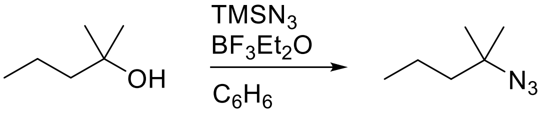

Synthesized according to a literature procedure.[S28] In an oven dried Schlenk under an argon atmosphere 2-methylpentan-2-ol (5.25 g; 51.4 mmol; 1.0 eq) and $TMSN_3$ (8.2 mL; 61.7 mmol; 1.2 eq) was dissolved in anhydrous C_6H_6 (200 mL). BF_3Et_2O (7.6 mL; 61.7 mmol; 1.2 eq) was added dropwise and the solution was stirred for 16 h. The obtained mixture was quenched with water (100 mL), extracted with Et_2O (3x 100 mL), washed with brine (100 mL), dried over Na_2SO_4, filtered and concentrated. The crude product was purified by column chromatography over SiO_2 using hexane as eluent. The product was obtained as a colorless oil (4.99 g; 51.4 mmol; 76%).

Spectral data were consistent with previously reported characterization of the product.[S28] ^1H NMR (300 MHz, $CDCl_3$) δ 1.53 – 1.30 (m, 4H), 1.25 (s, 6H), 0.93 (t, J = 7.4 Hz, 3H). ^{13}C NMR (75 MHz, $CDCl_3$) δ 61.83, 43.90, 26.13, 17.70, 14.52.

Substrate **18a**

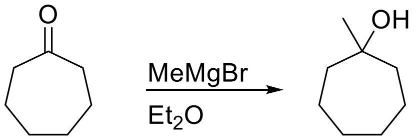

Synthesized according to a literature procedure.[S37] In an oven dried Schlenk under an argon atmosphere cycloheptanone (5.15 g; 45.9 mmol; 1.0 eq) was dissolved in anhydrous Et_2O (200 mL) and cooled to 0 °C. A solution of 3.0 M MeMgBr (30.6 mL; 91.8 mmol; 2.0 eq) in Et_2O was added dropwise and the obtained white suspension was stirred for 16 h. The mixture was quenched with concentrated aqueous NH_4Cl (50 mL) solution and extracted with Et_2O (3x 100 mL), washed with brine (100 mL), dried over Na_2SO_4, filtered and concentrated. The product was obtained as a colorless oil (4.28 g; 33.4 mmol; 73%).

Spectral data were consistent with previously reported characterization of the product.[S37] ^1H NMR (300 MHz, $CDCl_3$) δ 1.76 – 1.45 (m, 12H), 1.44 – 1.29 (m, 3H), 1.22 (s, 3H). ^{13}C NMR (75 MHz, $CDCl_3$) δ 74.10, 43.17, 31.29, 29.85, 22.76.

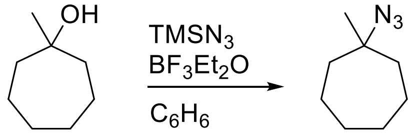

Synthesized according to a literature procedure.[S37] In an oven dried Schlenk under an argon atmosphere 1-methylcycloheptan-1-ol (4.13 g; 32.2 mmol; 1.0 eq) and $TMSN_3$ (5.1 mL; 38.6 mmol; 1.2 eq) was dissolved in anhydrous C_6H_6 (200 mL). BF_3Et_2O (4.8 mL; 38.6 mmol; 1.2 eq) was added dropwise and the solution was stirred for 16 h. The obtained mixture was quenched with water (100 mL), extracted with Et_2O (3x 100 mL), washed with brine (100 mL),

dried over Na_2SO_4, filtered and concentrated. The crude product was purified by column chromatography over SiO_2 using hexane as eluent. The product was obtained as a colorless oil (2.12 g; 13.8 mmol; 43%).

Spectral data were consistent with previously reported characterization of the product.[S37] ^{1}H NMR (300 MHz, $CDCl_3$) δ 1.86 – 1.33 (m, 12H), 1.29 (s, 3H). ^{13}C NMR (75 MHz, $CDCl_3$) δ 65.36, 40.24, 29.44, 27.59, 22.68.

<u>Substrate **19a**</u>

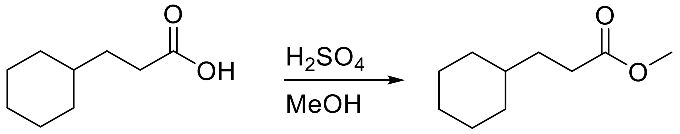

Synthesized according to a literature procedure.[S38] 3-cyclohexylpropanoic acid (8.06 g; 51.6 mmol; 1.0 eq) was dissolved in MeOH (100 mL) and 10 drops of concentrated sulphuric acid were added. The solution was stirred for 72 h at 60 °C and concentrated under reduced pressure. Water (100 mL) was added and the emulsion was extracted with Et_2O (3x 100 mL), washed with brine (100 mL), dried over Na_2SO_4, filtered and concentrated. The product was obtained as a colorless oil (8.22 g; 48.3 mmol; 94%).

Spectral data were consistent with previously reported characterization of the product.[S38] ^{1}H NMR (300 MHz, $CDCl_3$) δ 3.60 (s, 3H), 2.25 (t, J = 7.7 Hz, 2H), 1.73 – 1.51 (m, 5H), 1.46 (q, J = 7.2 Hz, 2H), 1.27 – 0.97 (m, 4H), 0.92 – 0.74 (m, 2H). ^{13}C NMR (75 MHz, $CDCl_3$) δ 174.52, 51.39, 37.24, 32.98, 32.36, 31.65, 26.55, 26.23.

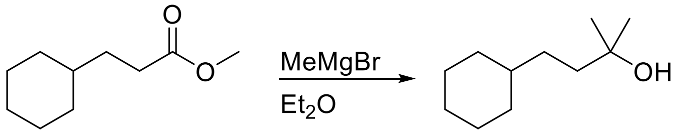

Synthesized according to a literature procedure.[S39] In an oven dried Schlenk under an argon atmosphere methyl 3-cyclohexylpropanoate (8.08 g; 47.5 mmol; 1.0 eq) was dissolved in anhydrous Et_2O (200 mL) and cooled to 0 °C. A solution of 3.0 M MeMgBr (47.5 mL; 122.4 mmol; 3.0 eq) in Et_2O was added dropwise and the obtained white suspension was stirred for 16 h. The mixture was quenched with concentrated aqueous NH_4Cl (50 mL) solution and extracted with Et_2O (3x 100 mL), washed with brine (100 mL), dried over Na_2SO_4, filtered and concentrated. The product was obtained as a colorless oil (5.67 g; 33.3 mmol; 70%).

Spectral data were consistent with previously reported characterization of the product.[S39] ^{1}H NMR (300 MHz, $CDCl_3$) δ 1.77 – 1.53 (m, 7H), 1.51 – 1.36 (m, 2H), 1.34 – 1.00 (m, 13H), 0.94 – 0.75 (m, 2H). ^{13}C NMR (75 MHz, $CDCl_3$) δ 71.08, 41.30, 38.27, 33.51, 31.99, 29.20, 26.76, 26.46.

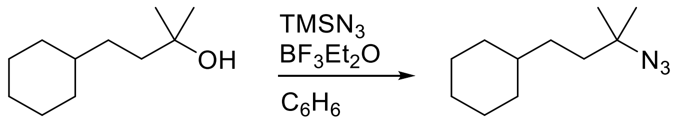

Synthesized according to a literature procedure.[S32] In an oven dried Schlenk under an argon atmosphere 4-cyclohexyl-2-methylbutan-2-ol (5.67 g; 33.3 mmol; 1.0 eq) and $TMSN_3$ (5.3 mL; 40.0 mmol; 1.2 eq) was dissolved in anhydrous C_6H_6 (200 mL). BF_3Et_2O (4.9 mL; 40.0 mmol; 1.2 eq) was added dropwise and the solution was stirred for 16 h. The obtained mixture was quenched with water (100 mL), extracted with Et_2O (3x 100 mL), washed with brine (100 mL), dried over Na_2SO_4, filtered and concentrated. The crude product was purified by column chromatography over SiO_2 using hexane as eluent. The product was obtained as a colorless oil (2.27 g; 11.6 mmol; 35%).

Spectral data were consistent with previously reported characterization of the product.[S32] ^{1}H NMR (300 MHz, $CDCl_3$) δ 1.79 – 1.57 (m, 5H), 1.54 – 1.40 (m, 2H), 1.36 – 1.01 (m, 12H), 0.98 – 0.79 (m, 2H). ^{13}C NMR (75 MHz, $CDCl_3$) δ 61.92, 38.89, 38.15, 33.51, 31.90, 26.78, 26.49, 26.12.

<u>Substrate **20a**</u>

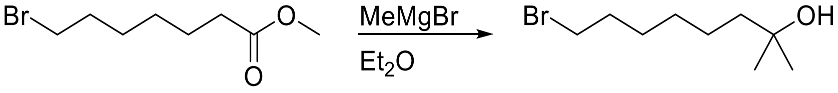

Synthesized according to a literature procedure.[S40] In an oven dried Schlenk under an argon atmosphere methyl 7-bromoheptanoate (5.00 g; 22.4 mmol; 1.0 eq) was dissolved in anhydrous Et_2O (200 mL) and cooled to 0 °C. A solution of 3.0 M MeMgBr (22.4 mL; 67.2 mmol; 3.0 eq) in Et_2O was added dropwise and the obtained white suspension was stirred for 16 h. The mixture was quenched with concentrated aqueous NH_4Cl (50 mL) solution and extracted with Et_2O (3x 100 mL), washed with brine (100 mL), dried over Na_2SO_4, filtered and concentrated. The product was obtained as a colorless oil (5.00 g; 22.4 mmol; >99%).

Spectral data were consistent with previously reported characterization of the product.[S40] ^{1}H NMR (300 MHz, $CDCl_3$) δ 3.41 (t, *J* = 6.8 Hz, 2H), 1.86 (p, *J* = 6.9 Hz, 2H), 1.52 – 1.26 (m, 7H), 1.21 (s, 6H). ^{13}C NMR (75 MHz, $CDCl_3$) δ 71.12, 43.94, 34.10, 32.89, 29.43, 29.40, 28.29, 24.30.

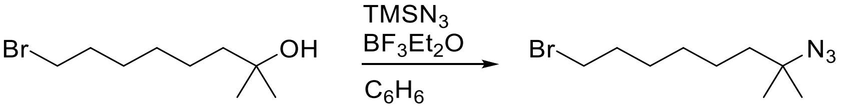

Synthesized according to a literature procedure.[S32] In an oven dried Schlenk under an argon atmosphere 8-bromo-2-methyloctan-2-ol (5.00 g; 22.4 mmol; 1.0 eq) and $TMSN_3$ (3.6 mL; 26.9 mmol; 1.2 eq) was dissolved in anhydrous C_6H_6 (200 mL). BF_3Et_2O (3.3 mL; 26.9 mmol; 1.2 eq) was added dropwise and the solution was stirred for 16 h. The obtained mixture was quenched with water (100 mL), extracted with Et_2O (3x 100 mL), washed with brine (100 mL), dried over Na_2SO_4, filtered and concentrated. The crude product was purified by column chromatography over SiO_2 using hexane as eluent. The product was obtained as a colorless oil (2.10 g; 8.46 mmol; 38%).

Spectral data were consistent with previously reported characterization of the product.[S32] ^{1}H NMR (300 MHz, $CDCl_3$) δ 3.40 (t, *J* = 6.8 Hz, 2H), 1.85 (p, *J* = 6.9 Hz, 2H), 1.52 – 1.40 (m, 4H),

1.40 – 1.28 (m, 4H), 1.24 (s, 6H). ^{13}C NMR (75 MHz, $CDCl_3$) δ 61.61, 41.34, 33.87, 32.70, 29.02, 28.07, 25.99, 24.07.

Substrate **1a-d₂**

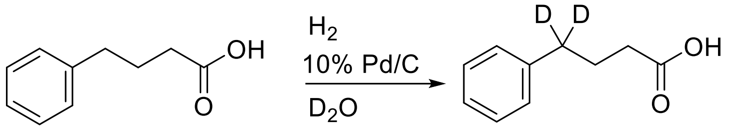

Synthesized according to a literature procedure.[S29] 4-phenylbutanoic acid (5.35 g; 32.6 mmol; 1.0 eq) and 10% Pd/C (0.519 g; 0.48 mmol; 0.015 eq) were suspended in D_2O (25 mL). The obtained black suspension was purged three times with H_2 and stirred for 72 h at 50 °C. The black suspension was filtered through Celite and washed with Et_2O (200 mL). The water layer was extracted with Et_2O (3x 100 mL) and the organic layers were combined, washed with brine, dried over Na_2SO_4, filtered and concentrated. The product was obtained as a colorless oil (4.32 g; 26.0 mmol; 80%). A deuteration of 96% was achieved according to ^1H NMR.

Spectral data were consistent with previously reported characterization of the product.[S29] ^1H NMR (300 MHz, C_6D_6) δ 7.22 – 6.81 (m, 5H), 2.00 (t, *J* = 7.4 Hz, 2H), 1.69 (t, *J* = 7.3 Hz, 2H).

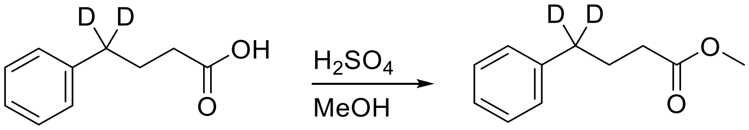

Synthesized according to a literature procedure.[S29] 4-phenylbutanoic-4,4-d_2 acid (4.32 g; 26.0 mmol; 1.0 eq) was dissolved in MeOH (100 mL) and 10 drops of concentrated sulphuric acid were added. The solution was stirred for 16 h and concentrated under reduced pressure. Water (100 mL) was added and the emulsion was extracted with Et_2O (3x 250 mL), washed with brine (100 mL), dried over Na_2SO_4, filtered and concentrated. The product was obtained as a colorless oil (4.06 g; 22.5 mmol; 87%).

Spectral data were consistent with previously reported characterization of the product.[S29] ^1H NMR (300 MHz, $CDCl_3$) δ 7.32 – 7.17 (m, 2H), 7.17 – 7.03 (m, 3H), 3.59 (s, 3H), 2.26 (t, *J* = 7.5 Hz, 2H), 1.87 (t, *J* = 7.4 Hz, 2H). ^{13}C NMR (75 MHz, $CDCl_3$) δ 174.09, 141.43, 128.60, 128.51, 126.11, 51.64, 35.49 – 33.79 (m), 33.47, 26.46.

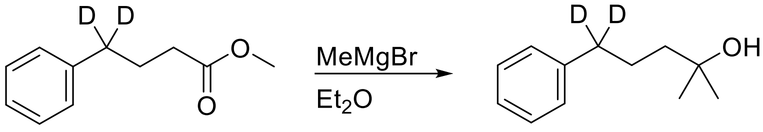

Synthesized according to a literature procedure.[S29] In an oven dried Schlenk under an argon atmosphere methyl 4-phenylbutanoate-4,4-d_2 (3.97 g; 22.0 mmol; 1.0 eq) was dissolved in anhydrous Et_2O (300 mL) and cooled to 0 °C. A solution of 3.0 M MeMgBr (22.03 mL; 66.1 mmol; 3.0 eq) in Et_2O was added dropwise and the obtained white suspension was stirred for

16 h. The mixture was quenched with concentrated aqueous NH_4Cl (100 mL) solution and extracted with Et_2O (3x 100 mL), washed with brine (100 mL), dried over Na_2SO_4, filtered and concentrated. The product was obtained as a colorless oil (3,66 g; 20.3 mmol; 92%).

Spectral data were consistent with previously reported characterization of the product.[S29] ^{1}H NMR (300 MHz, $CDCl_3$) δ 7.31 – 7.16 (m, 2H), 7.16 – 7.03 (m, 3H), 1.68 – 1.56 (m, 2H), 1.50 – 1.38 (m, 2H), 1.14 (s, 6H).

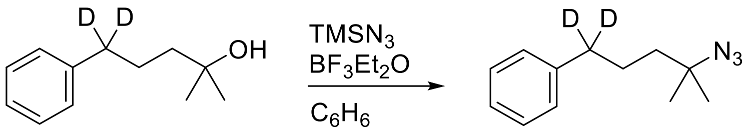

Synthesized according to a literature procedure.[S29] In an oven dried Schlenk under an argon atmosphere 2-methyl-5-phenylpentan-5,5-d_2-2-ol (3.60 g; 20.0 mmol; 1.0 eq) and $TMSN_3$ (3.2 mL; 24.0 mmol; 1.2 eq) was dissolved in anhydrous C_6H_6 (300 mL). BF_3Et_2O (3.0 mL; 24.0 mmol; 1.2 eq) was added dropwise and the solution was stirred for 16 h. The obtained mixture was quenched with water (100 mL), extracted with Et_2O (3x 100 mL), washed with brine (100 mL), dried over Na_2SO_4, filtered and concentrated. The crude product was purified by column chromatography over SiO_2 using hexane as eluent. The product was obtained as a colorless oil (1.48 g; 7.2 mmol; 36%).

Spectral data were consistent with previously reported characterization of the product.[S29] ^{1}H NMR (300 MHz, $CDCl_3$) δ 7.26 – 7.17 (m, 2H), 7.16 – 7.07 (m, 3H), 1.65 – 1.55 (m, 2H), 1.49 – 1.40 (m, 2H), 1.18 (s, 6H). ^{13}C NMR (75 MHz, $CDCl_3$) δ 142.10, 128.50, 126.00, 61.70, 41.13, 36.24 – 34.68 (m), 26.12.

Catalysis

General procedure

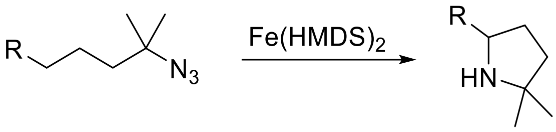

Inside an argon filled glovebox, a stock solution was made by dissolving $Fe(HMDS)_2$ (9.3 mg; 0.025 mmol) in 2 mL of deuterated solvent. A stock solution of internal standard was made by dissolving 1,3,5-trimethoxybenzene (45.5 mg; 0.0271 mmol) in 1 mL of deuterated solvent. The corresponding azide (25 mmol) weighed into a vial, stock solution of $Fe(HMDS)_2$ (0.2 mL), internal standard stock solution (0.1 mL) and deuterated solvent (0.2 mL) were added. The contents of the vial was transferred into a J Young NMR tube. The NMR tube was taken outside the glovebox and heated in an oil bath (Figure S1). Yields and conversions were determined by ^1H NMR spectroscopy using the benzylic CH and CH_2 resonances, respectively. Small quantities of cyclic imine and of the acyclic amine (from formal azide reduction) were also detected in some of the product NMR spectra. Catalytic runs with lower catalyst loadings were performed by dilution of the $Fe(HMDS)_2$ stock solution.

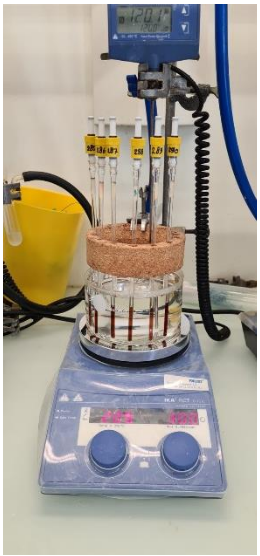

Figure S1: Typical setup for running catalytic experiments.

Characterization of C–H aminated products

All products were characterized as crude mixtures after catalysis was completed, unless stated otherwise.

Product **1b**

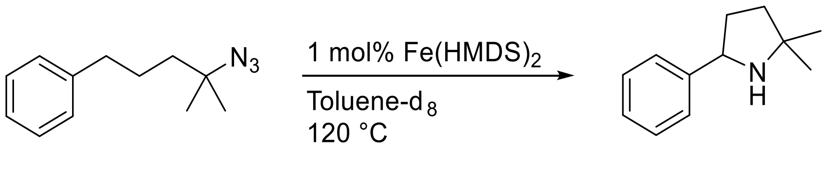

Spectral data were consistent with previously reported characterization of the product.[S36] ^{1}H NMR (300 MHz, Tol) δ 7.42 – 7.35 (m, 2H), 7.20 (t, *J* = 7.4 Hz, 2H), 7.12 – 7.08 (m, 1H), 4.10 (q, *J* = 7.4 Hz, 1H), 2.07 – 1.91 (m, 1H), 1.70 – 1.50 (m, 2H), 1.50 – 1.36 (m, 1H), 1.17 (s, 3H), 1.04 (s, 3H).

Product **5b**

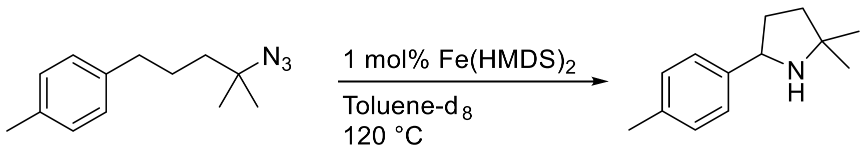

Spectral data were consistent with previously reported characterization of the product.[S36] ^{1}H NMR (300 MHz, Tol) δ 7.35 (d, *J* = 8.0 Hz, 2H), 7.06 (d, *J* = 7.9 Hz, 2H), 4.16 (q, J = 7.5 Hz, 1H), 2.22 (s, 3H), 2.10 – 1.97 (m, 1H), 1.77 – 1.64 (m, 1H), 1.63 – 1.56 (m, 1H), 1.55 – 1.43 (m, 1H), 1.22 (s, 3H), 1.09 (s, 3H).

Product **6b**

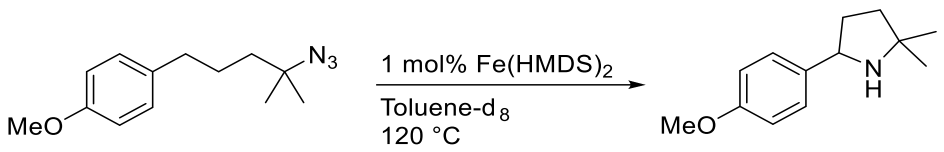

Spectral data were consistent with previously reported characterization of the product.[S36] ^{1}H NMR (300 MHz, Tol) δ 7.30 (d, *J* = 8.1 Hz, 2H), 6.79 (d, *J* = 8.7 Hz, 2H), 4.11 (q, *J* = 7.4 Hz, 1H), 3.39 (s, 3H), 2.09 – 1.91 (m, 1H), 1.70 – 1.40 (m, 3H), 1.18 (s, 3H), 1.06 (s, 3H).

Product **7b**

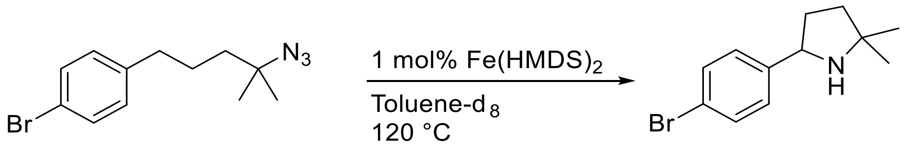

Spectral data were consistent with previously reported characterization of the product.[S36] ^{1}H NMR (300 MHz, Tol) δ 7.27 (d, *J* = 8.1 Hz, 2H), 7.07 (d, *J* = 8.1 Hz, 2H), 4.45 – 3.62 (m, 1H), 2.05 – 1.79 (m, 1H), 1.60 – 1.37 (m, 3H), 1.12 (s, 3H), 1.04 (s, 3H).

Product **8b**

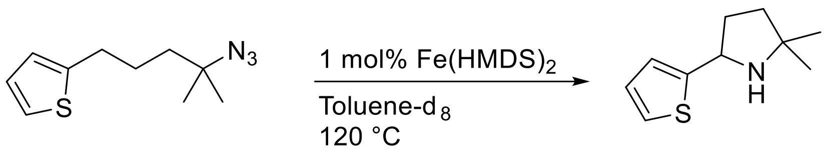

Spectral data were consistent with previously reported characterization of the product.[S36] ^{1}H NMR (300 MHz, Tol) δ 6.90 – 6.85 (m, 1H), 6.79 – 6.76 (m, 2H), 4.31 (q, *J* = 7.4 Hz, 1H), 2.06 – 1.92 (m, 1H), 1.79 – 1.69 (m, 1H), 1.63 – 1.49 (m, 1H), 1.43 – 1.33 (m, 1H), 1.13 (s, 3H), 0.96 (s, 3H).

Product **9b**

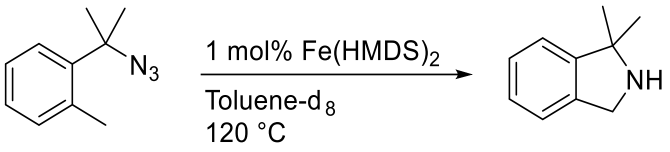

Spectral data were consistent with previously reported characterization of the product.[S36] ^{1}H NMR (300 MHz, Tol) δ 7.08 – 7.06 (m, 2H), 7.00 – 6.98 (m, 2H), 3.96 (s, 1H), 3.93 (s, 1H), 1.25 (s, 6H).

Product **10b**

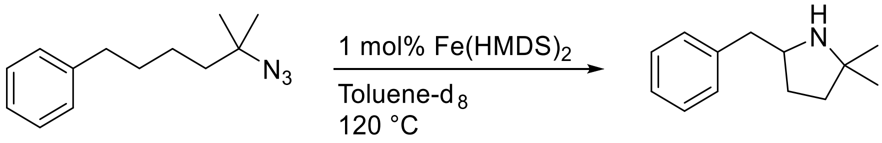

Spectral data were consistent with previously reported characterization of the product.[S36] ^{1}H NMR (300 MHz, Tol) δ 7.23 – 6.95 (m, 5H), 3.33 – 3.24 (m, 1H), 2.67 – 2.47 (m, 1H), 1.78 – 1.58 (m, 1H), 1.58 – 1.25 (m, 3H), 1.09 (s, 3H), 0.95 (s, 3H).

Product **11b**

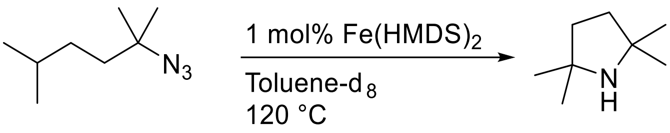

Spectral data were consistent with previously reported characterization of the product.[S36] ^{1}H NMR (300 MHz, Tol) δ 1.56 (s, 4H), 1.08 (s, 12H).

Product **12b**

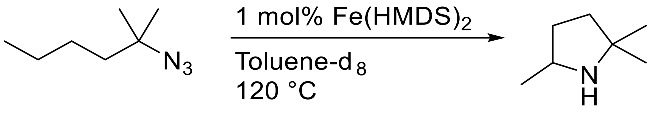

Spectral data were consistent with previously reported characterization of the product.[S36] ^{1}H NMR (300 MHz, Tol) δ 3.21 – 3.01 (m, 1H), 1.84 – 1.68 (m, 1H), 1.55 – 1.34 (m, 1H), 1.29 – 1.12 (m, 2H), 1.12 (s, 3H), 1.03 (d, *J* = 6.1 Hz, 3H), 1.03 (s, 3H), 0.87 – 0.78 (m, 1H).

Product **13b**

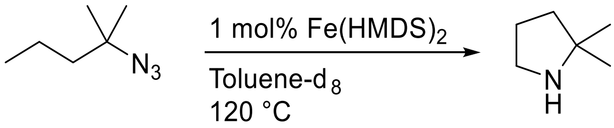

Spectral data were consistent with previously reported characterization of the product.[S36] ^{1}H NMR (300 MHz, Tol) δ 2.80 (q, J = 7.1 Hz, 2H), 1.65 – 1.52 (m, 2H), 1.36 – 1.28 (m, 3H), 1.03 (s, 6H).

Product **14b**

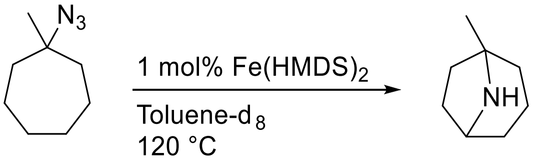

Spectral data were consistent with previously reported characterization of the product.[S36] ^{1}H NMR (300 MHz, Tol) δ 3.31 – 3.22 (m, 1H), 1.75 – 1.51 (m), 1.52 – 1.34 (m), 1.34 – 1.11 (m), 1.06 (s, 3H).

Product **15b**

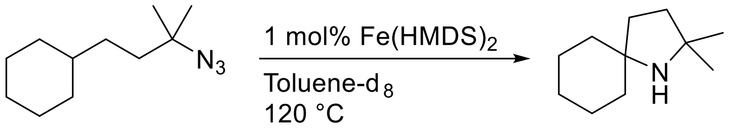

Spectral data were consistent with previously reported characterization of the product.[S36] ^{1}H NMR (300 MHz, Tol) δ 1.59 – 1.45 (m, 6H), 1.34 (q, J = 7.5, 5.3 Hz, 8H), 1.09 (s, 6H).

Product **1b-d$_2$**

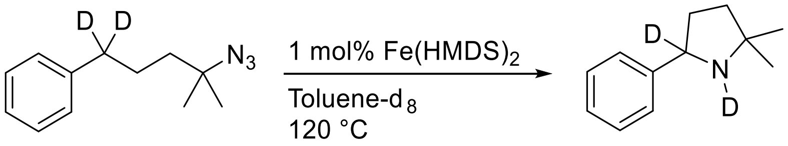

Spectral data were consistent with previously reported characterization of the product.[S36] ^{1}H NMR (300 MHz, Tol) δ 7.44 – 7.33 (m, 2H), 7.27 – 7.14 (m, 2H), 7.14 – 7.06 (m, 1H), 2.05 – 1.92 (m, 1H), 1.69 – 1.50 (m, 2H), 1.46 – 1.35 (m, 1H), 1.16 (s, 3H), 1.04 (s, 3H).

Kinetics

General procedure

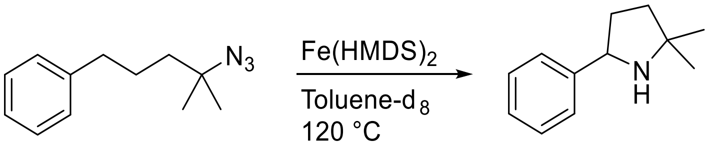

Inside an argon filled glovebox, a stock solution of $Fe(HMDS)_2$ was made in toluene-d_8 and a stock solution of 1,3,5-trimethoxybenzene (internal standard) was made in toluene-d_8. (4-azido-4-methylpentyl)benzene was weighed inside a vial stock solution of 1,3,5-trimethoxybenzene (0.1 mL) and stock solution of $Fe(HMDS)_2$ (0.2-0.4 mL) was added. The mixture was diluted with toluene-d_8 to 5.5 mL, this mixture was transferred into a J Young NMR tube. The NMR tube was taken outside the glovebox and heated in an oil bath at 120 °C. Samples were measured by removing the NMR tube from the oil bath and directly cooling it in an ice bath. After NMR measurement, the NMR tube was put back in the oil bath. The recorded time is the cumulative time heated in the oil bath.

Variation of substrate concentration

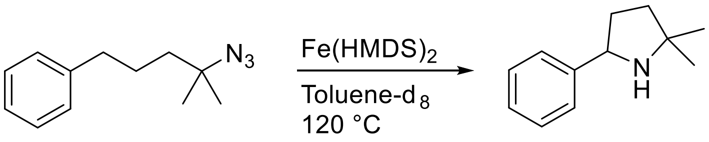

[cat] = 4.48 mM
[sub] = 0.11 - 0.89 M

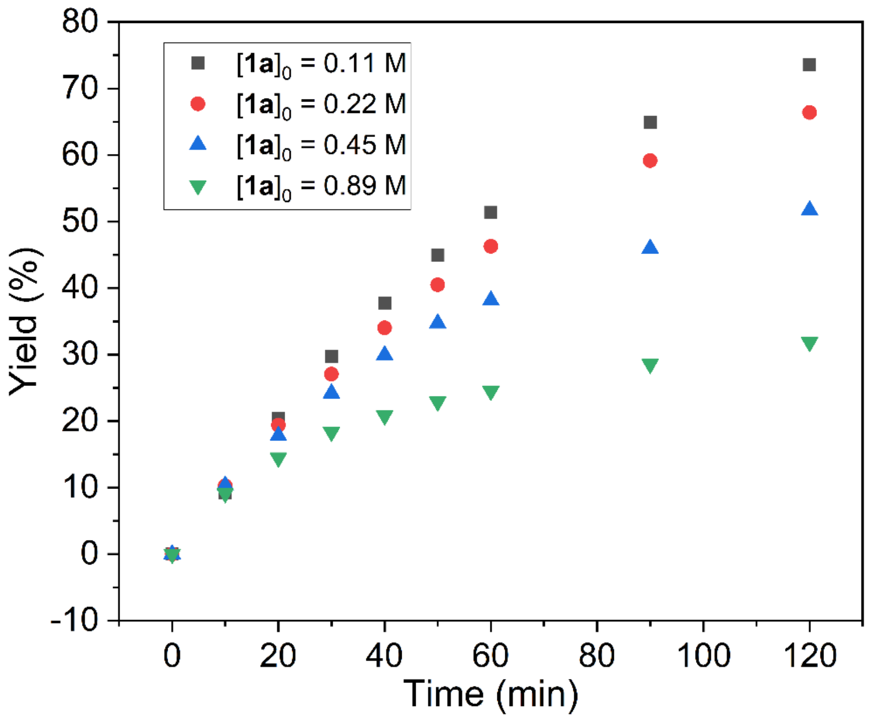

Figure S2: Yield of the N-heterocycle by intramolecular C–H amination over time with varying substrate concentrations at t=0 ($[sub]_0$ = 0.11 – 0.89 M) with [cat] = 4.48 mM. Yields were determined by ^1H NMR spectroscopy.

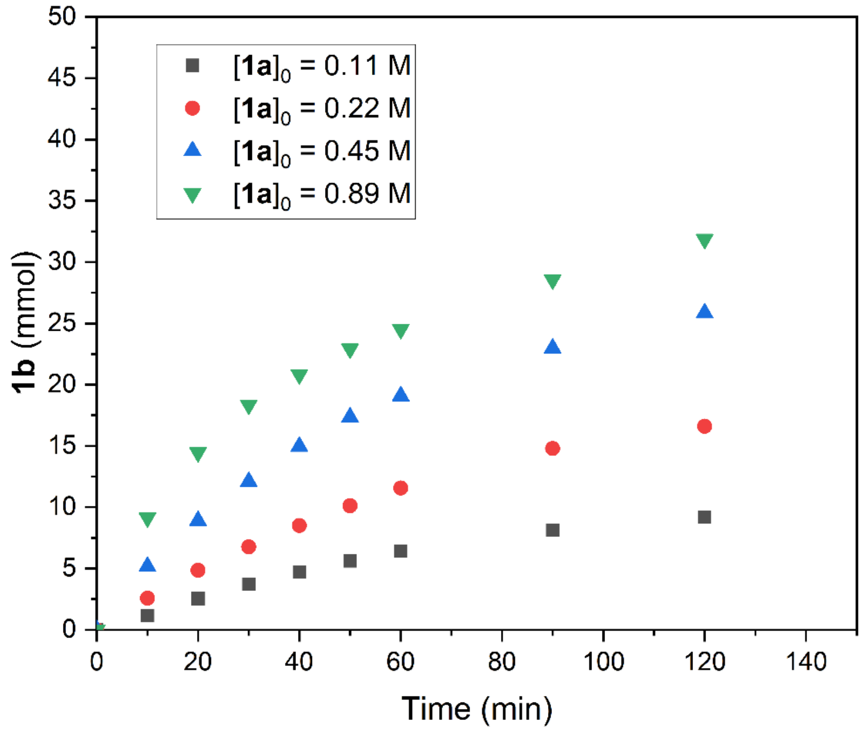

Figure S3: Formation of the *N*-heterocycle by intramolecular C–H amination over time with varying substrate concentrations at t=0 ($[sub]_0$ = 0.11 – 0.89 M) with [cat] = 4.48 mM. Amount of product was determined by ^{1}H NMR spectroscopy.

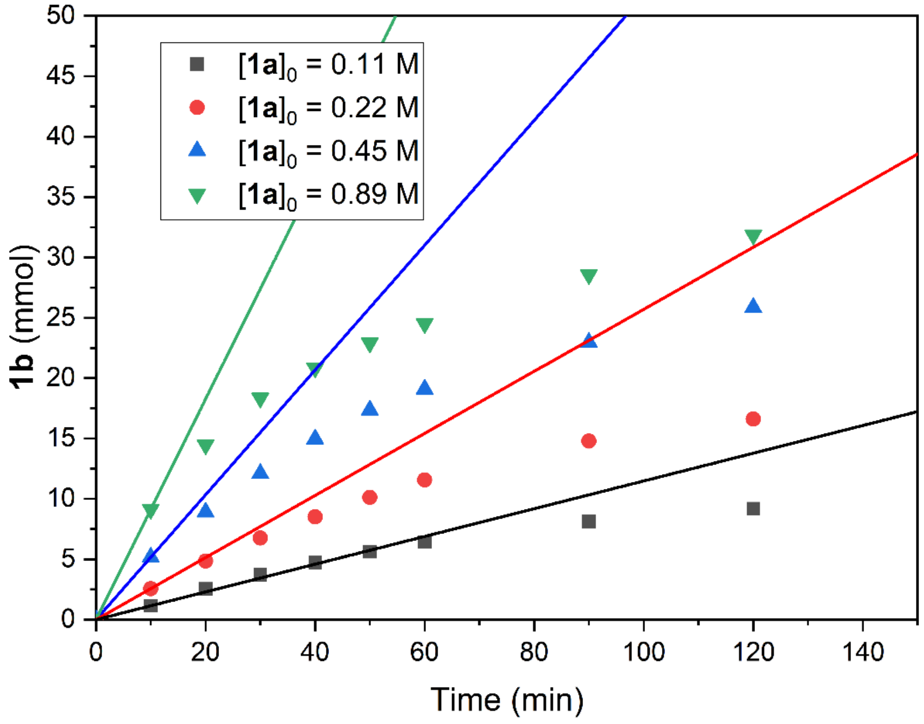

Figure S4: Formation of the *N*-heterocycle by intramolecular C–H amination over time with varying substrate concentrations at t=0 ($[sub]_0$ = 0.11 – 0.89 M) with [cat] = 4.48 mM. Amount of product was determined by ^{1}H NMR spectroscopy. Straight lines represent the initial reaction rate.

Table S1: Initial reaction rate with varying substrate concentrations at t=0 ($[sub]_0$ = 223.6 – 1788.8 mM) with [cat] = 4.48 mM.

$[sub]_0$	$R_{initial}$
0.11	0.1148
0.22	0.257
0.45	0.5167
0.89	0.9137

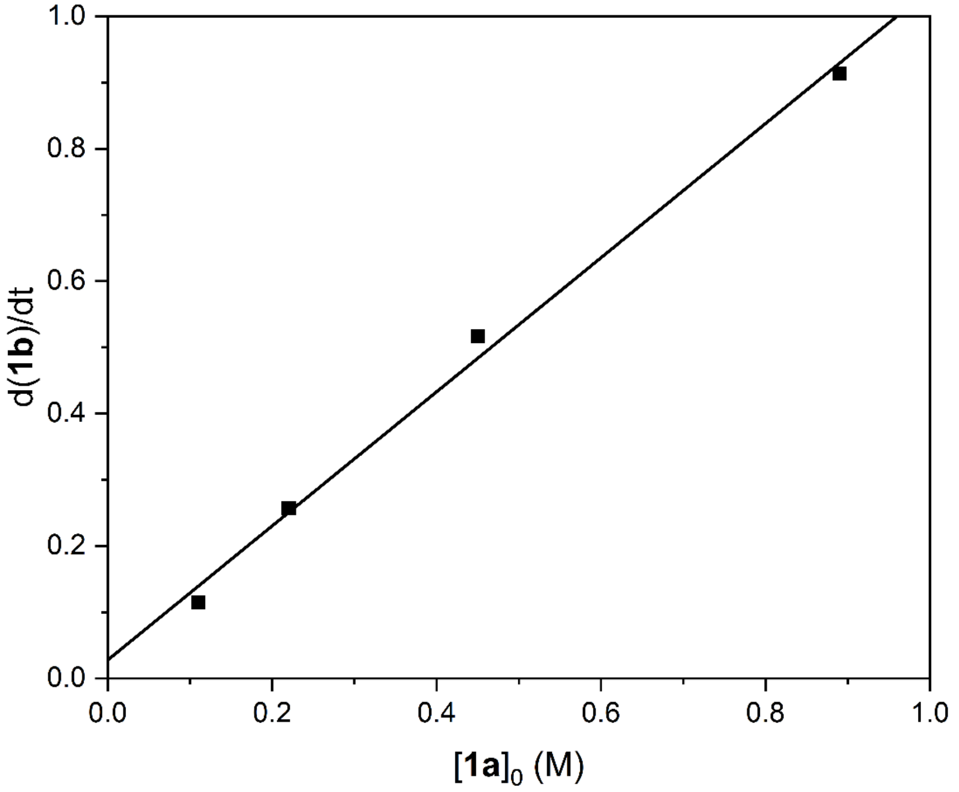

Figure S5: Initial reaction rate with varying substrate concentrations at t=0 ($[sub]_0$ = 0.11 – 0.89 M) with [cat] = 4.48 mM.

Variation of catalyst concentration

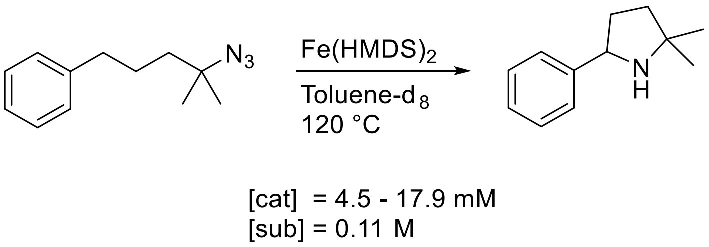

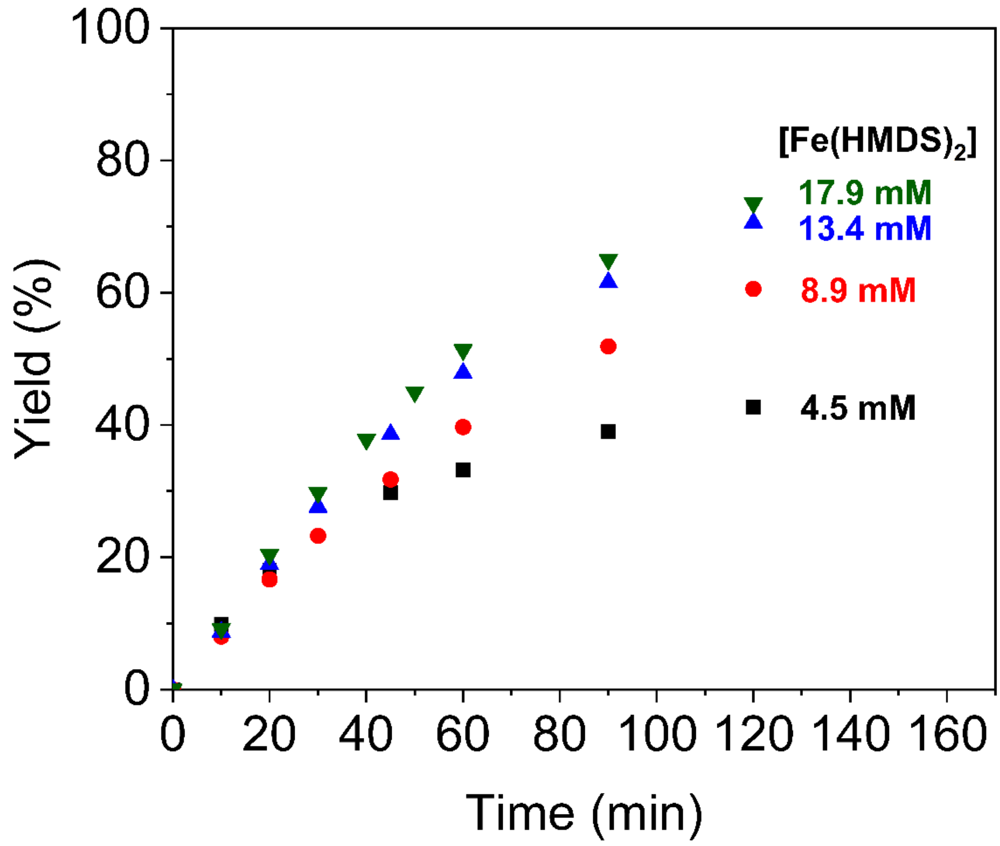

Figure S6: Formation of the *N*-heterocycle by intramolecular C–H amination over time with varying catalyst concentrations ([cat] = 4.5 – 17.9 mM with $[sub]_0$ = 0.11 M). Amount of product was determined by ^{1}H NMR spectroscopy.

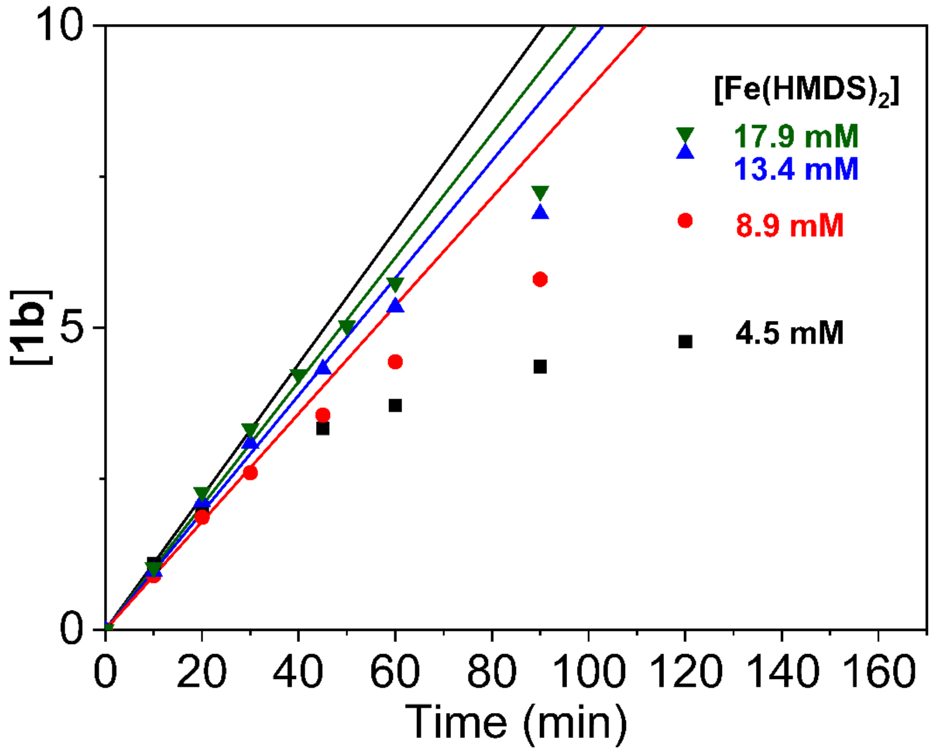

Figure S7: Formation of the N-heterocycle by intramolecular C–H amination over time with varying catalyst concentrations ([cat] = 4.5 – 17.9 mM with $[sub]_0$ = 0.11 M). Amount of product was determined by ^{1}H NMR spectroscopy. Straight lines represent the initial reaction rate.

Table 2: Maximum reaction rate with varying catalyst concentrations ([cat] = 4.5 – 17.9 mM with $[sub]_0$ = 0.11 M).

[Cat]	Rmax
4.5	1.10162
8.9	0.89455
13.4	0.97042
17.9	1.0269

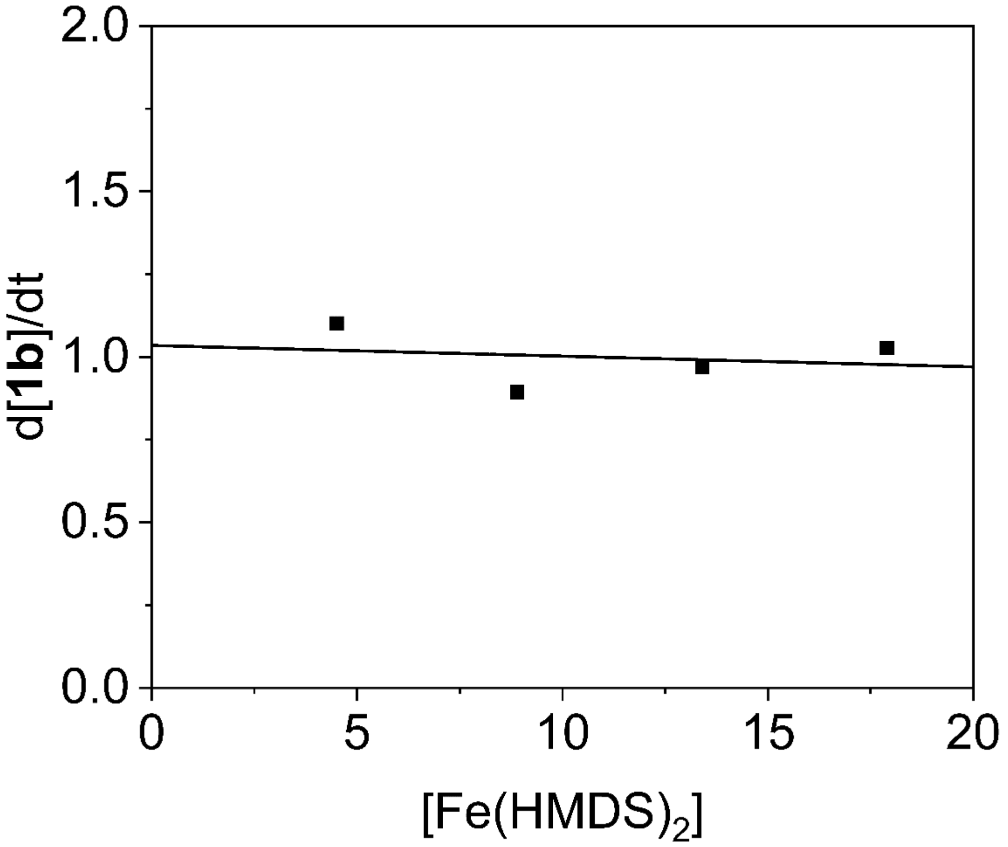

Figure S8: Initial reaction rate with varying catalyst concentrations at t=0 ([cat] = 4.5 – 17.9 mM with $[sub]_0$ = 0.11 M).

Product inhibition studies

The product used for these studies is a mixture of the amine **1b** product and the minor imine and linear amine side products. These were obtained from a regular catalytic run without using internal standard. The organic products were separated quantitatively from the iron by filtering the catalytic mixture over aluminum oxide and washing with pentane. Before use in catalytic experiments the product was degassed by three freeze-pump-thaw cycles and dried over 4 Å molecular sieves.

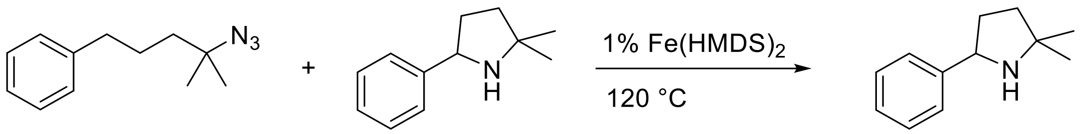

The catalysis was performed under standard conditions described before (1 mol% catalyst, toluene-d_8, 120 °C). Two independent catalytic runs were performed (Figure S9): one where one equivalent of product was added at t = 0 (red) and the other where the same amount of product was added at t = 20 min (black). A time conversion profile was obtained for both experiments and compared to a standard run without addition of product (blue). The time conversion plot shows that upon addition of product **1b**, the rate of the reaction decreases slightly, though conversion remains on-going. The minor decrease in rate is not compatible with a product inhibition scenario and might instead be attributed to catalyst decomposition in presence of the amine product.

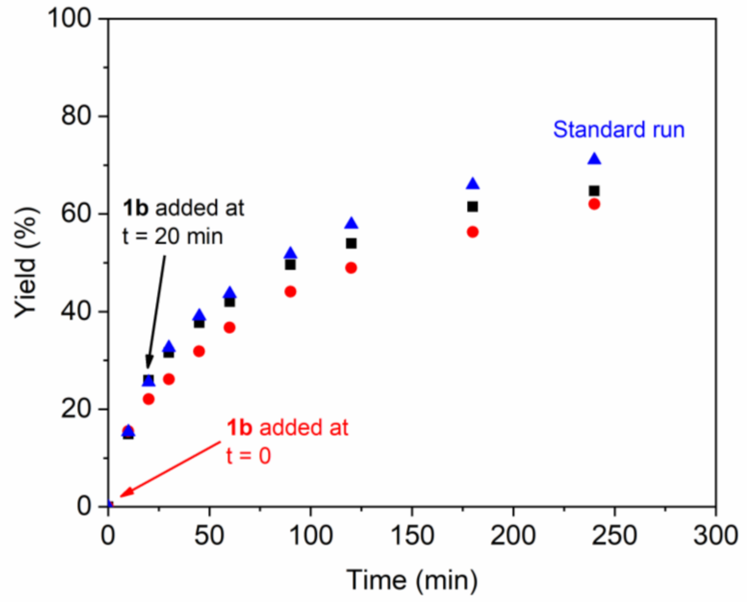

Figure S9: Formation of the N-heterocycle **1b** from azide **1a** by intramolecular C–H amination over time with addition of product **1b** at different reaction times (red: addition at t = 0; black: addition at t = 20 min; blue: standard run).

Catalyst decomposition studies

The catalysis was performed under standard conditions described before (1 mol% catalyst, toluene-d_8, 120 °C). Two independent catalytic runs were performed (Figure S10): one run where after 70% conversion, the reaction mixture was refilled with substrate **1a** to its initial concentration (red). In the other run, no substrate **1a** was added at the beginning and instead, 20mol% of **1b** were added to the catalyst and this mixture was heated at 120 °C for 2.5 h and only thereafter, substrate **1a** (1 equiv) was added (blue). Both time-conversion profiles were compared to the standard run (black). Both these experiments show a significant decrease in catalytic rate, indicating that in the presence of amine **1b** the activity of the catalyst is lost.

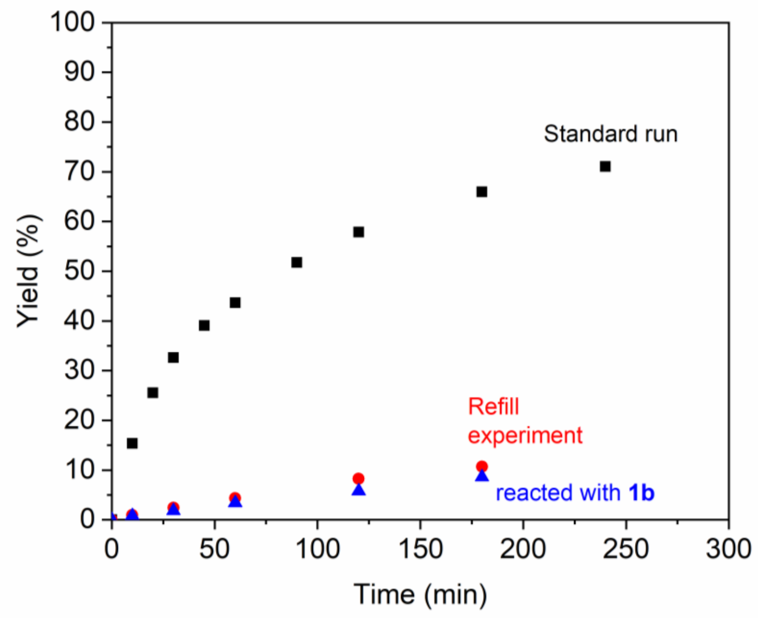

Figure S10: Time-dependent conversion plots for the formation of the N-heterocycle **1b** by intramolecular C–H amination of azide **1a**. Black: first run; red: conversion after refilling substrate **1a** to its initial substrate concentration of the first run (after the first run reached 70% conversion; blue: conversion of the azide after preheating the catalyst in the presence of product (20 mol%) for 2.5 h.

Intermolecular competition Kinetic isotope effect

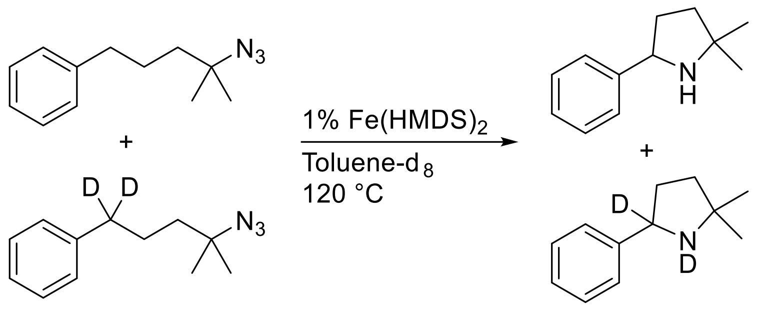

A 1:1 mixture of deuterated and non-deuterated substrate was used and the reaction was followed overtime under standard conditions.

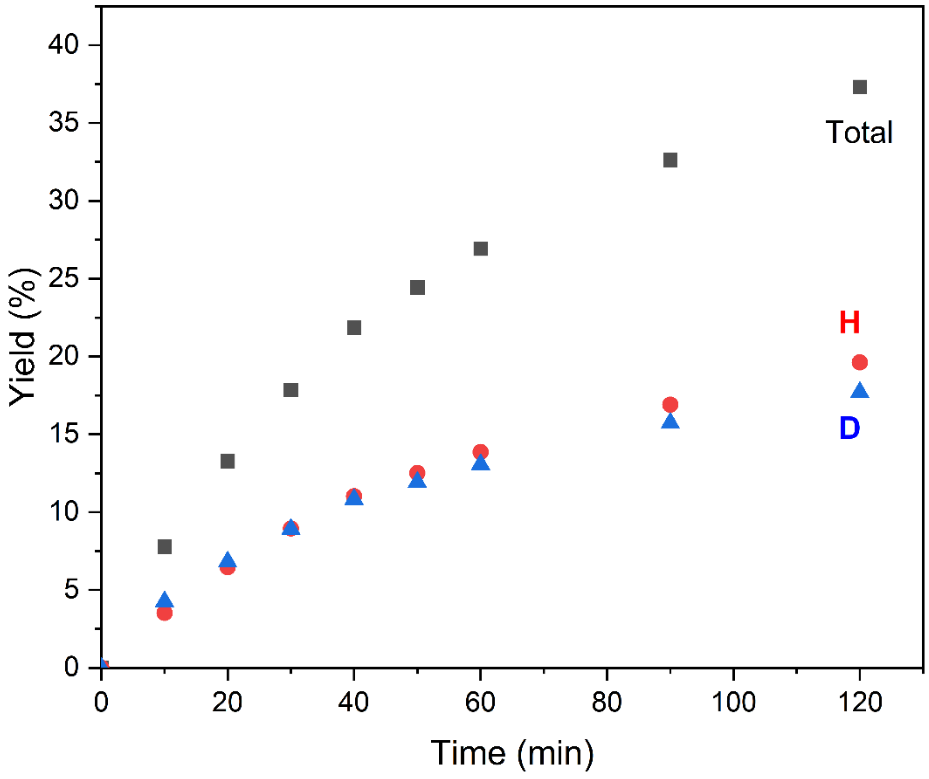

Figure S11: Yield of pyrrolidine by intramolecular C–H amination over time for the intermolecular KIE experiment ($[sub]_0$ = 0.45 M) with [cat] = 4.5 mM.

Intermolecular Kinetic isotope effect

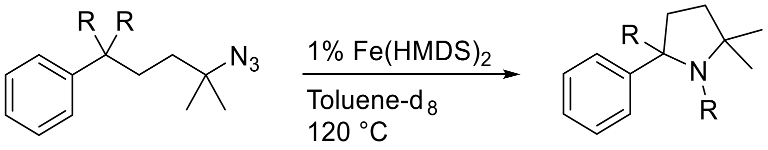

Two separate reactions with exclusively the deuterated and non-deuterated substrate were followed overtime under standard conditions.

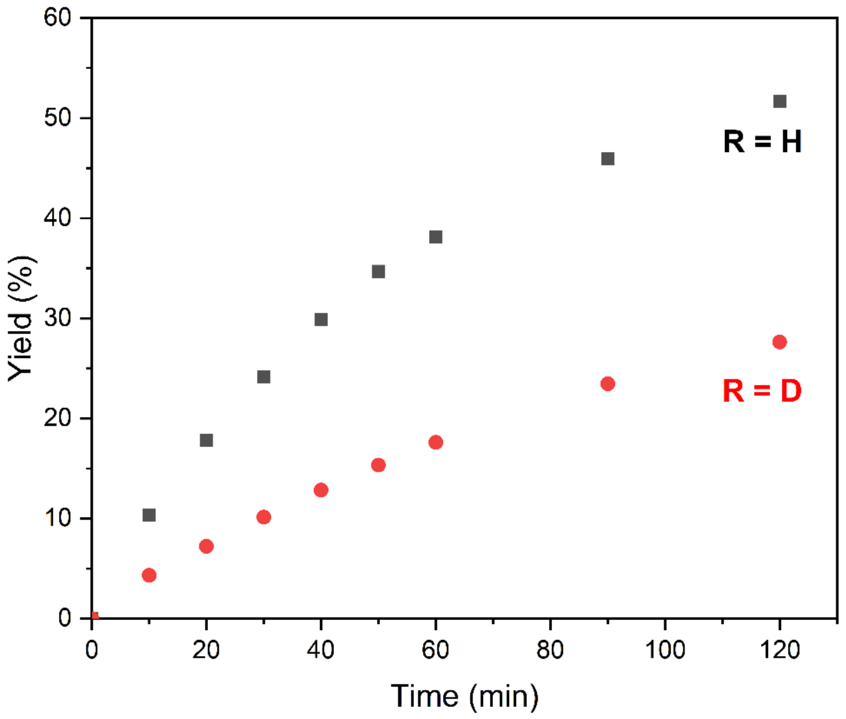

Figure S12: Yield of pyrrolidine by intramolecular C–H amination over time of the deuterated and non-deuterated **1b**.

Stoichiometric experiments

^{1}H NMR spectroscopy

Inside an argon filled glovebox Fe(HMDS)$_2$ (2.0, 1.0 or 0.5 eq.) was dissolved in C_6D_6 (1 mL) and added to (4-azido-4-methylpentyl)benzene (**1a**) (0.050 mg; 0.25 mmol; 1.0 eq). The obtained yellow solution was transferred into a J Young NMR tube and a ^{1}H NMR spectrum was measured. After leaving the solution for 24 h, a second ^{1}H NMR spectrum was measured.

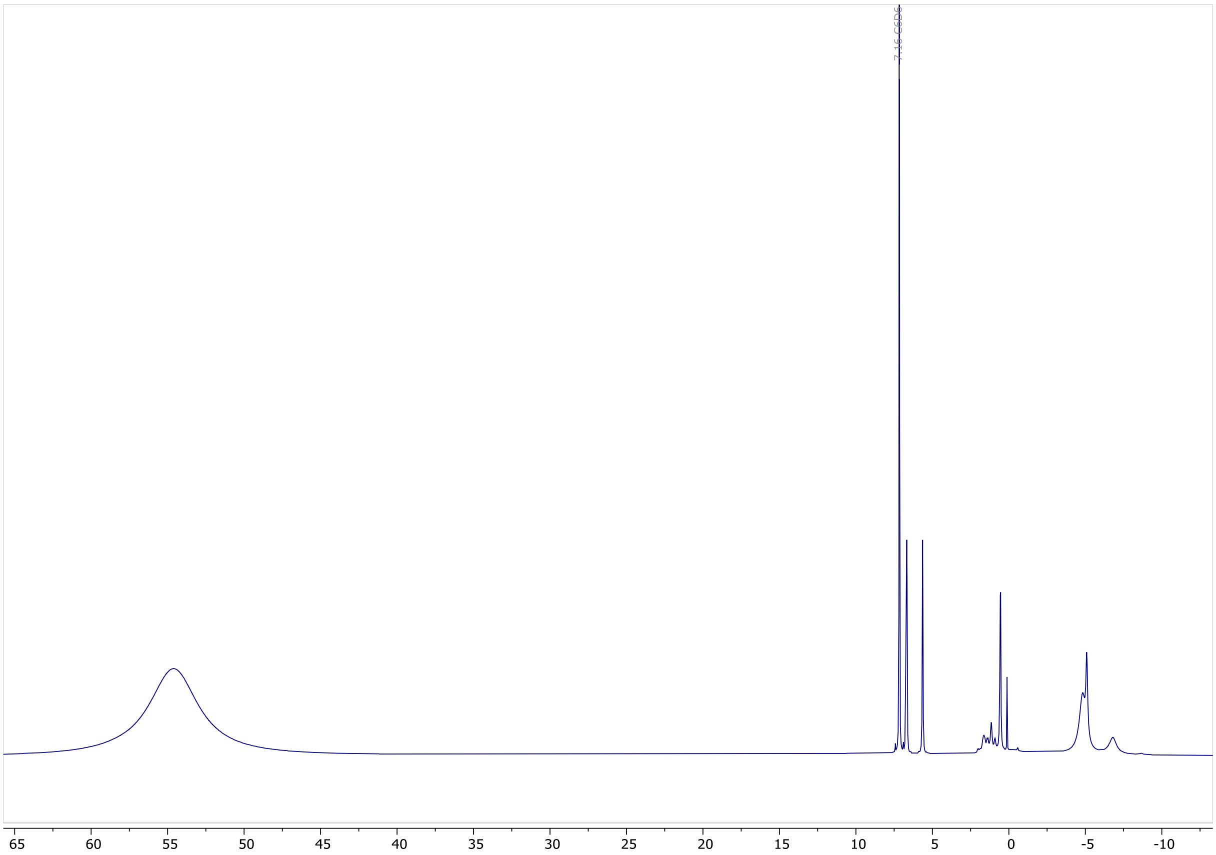

Figure S13: ^{1}H NMR spectra of the reaction with Fe(HMDS)$_2$ in C_6D_6 with 0.5 equivalents of substrate **1a** at t = 0.

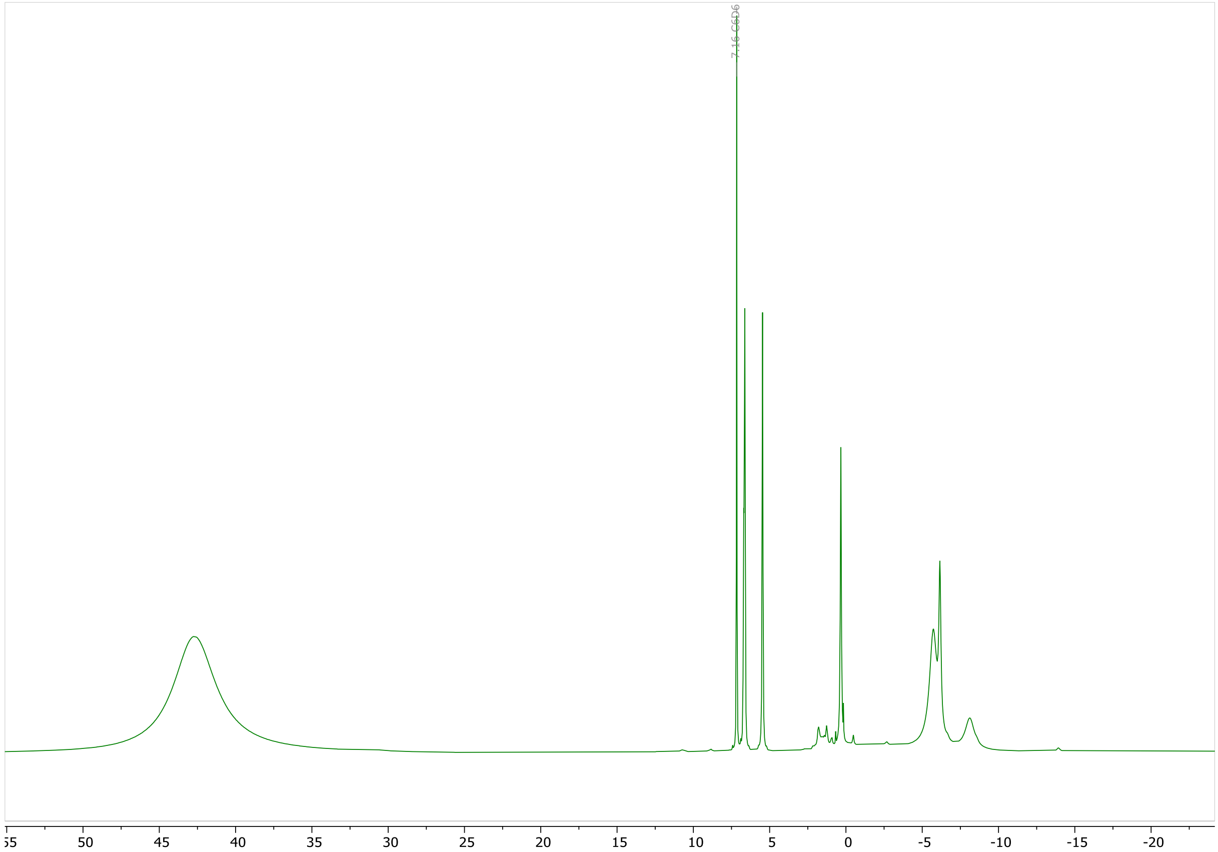

Figure S14: ^{1}H NMR spectra of the reaction with $Fe(HMDS)_2$ in C_6D_6 with 1.0 equivalents of substrate **1a** at t = 0.

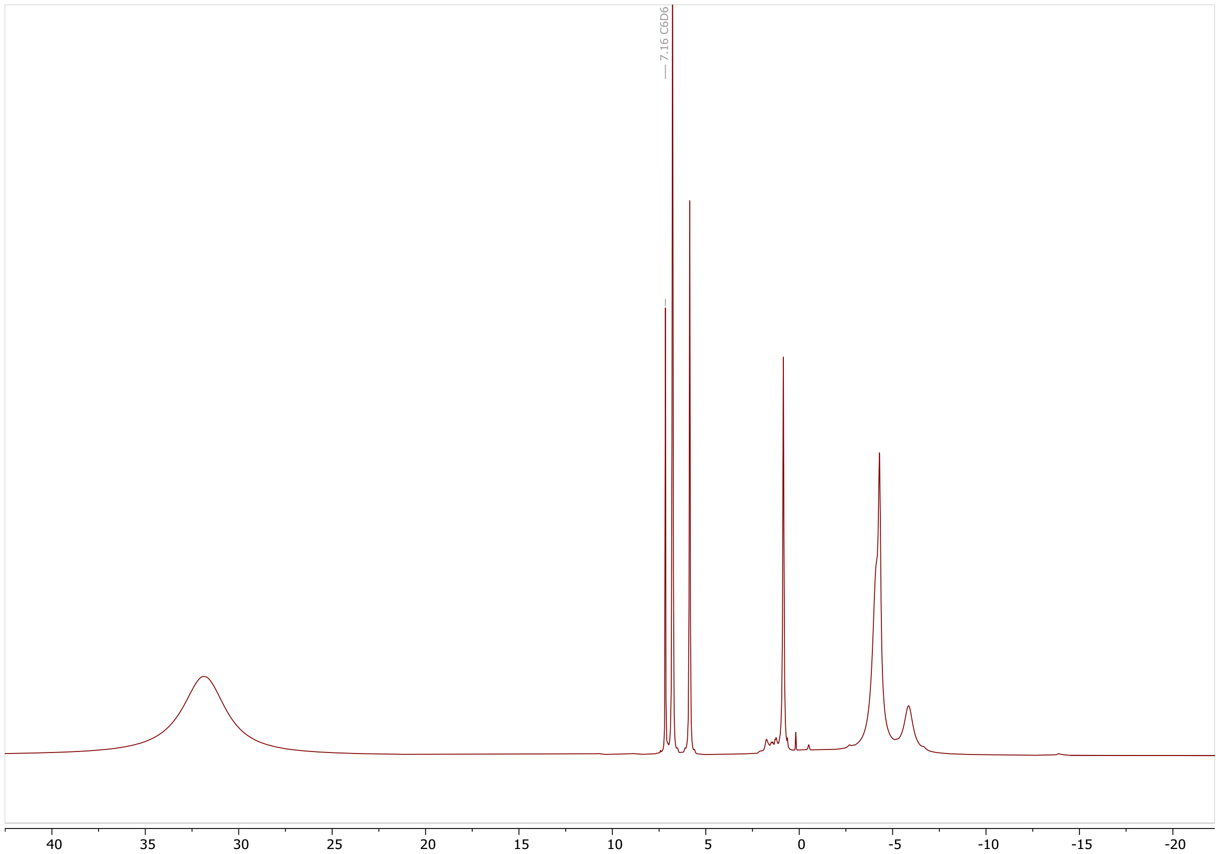

Figure S15: ^{1}H NMR spectra of the reaction with $Fe(HMDS)_2$ in C_6D_6 with 2.0 equivalents of substrate **1a** at t = 0.

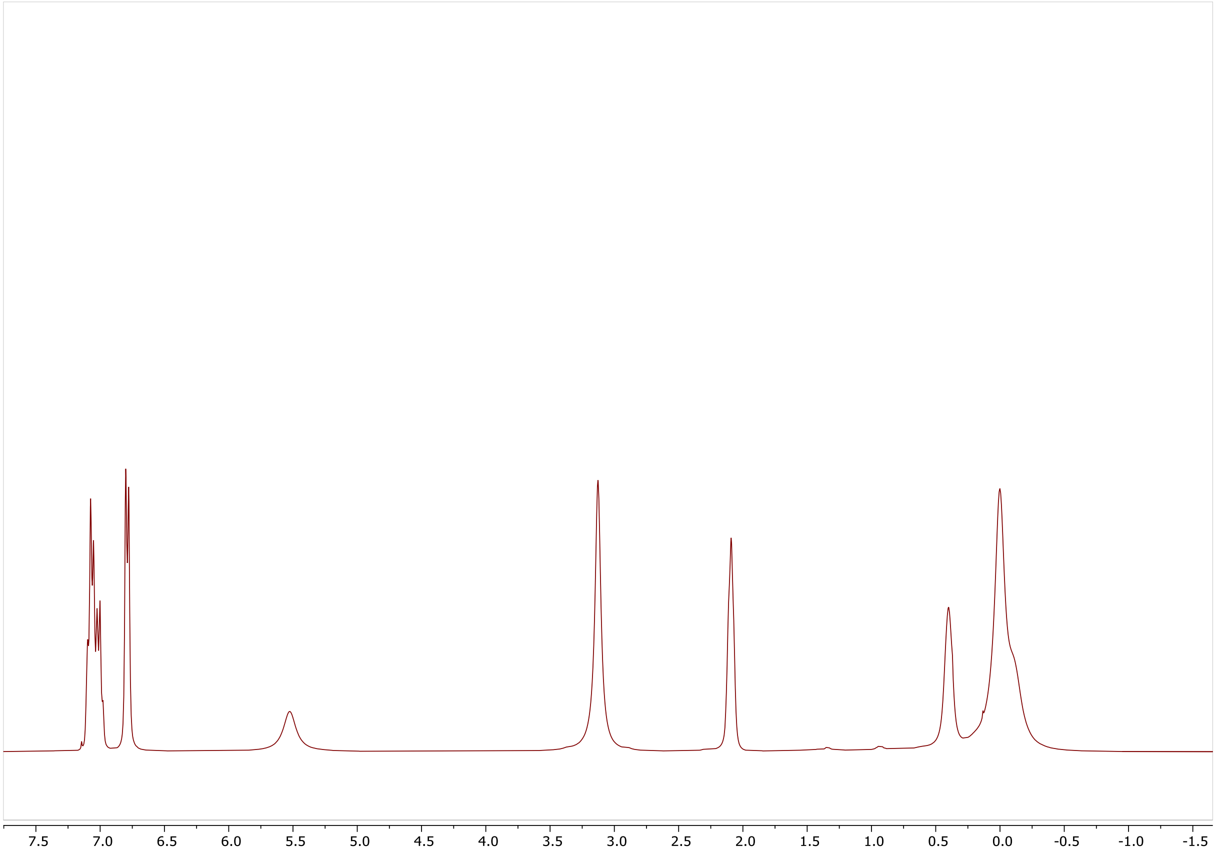

Figure S16: ^{1}H NMR spectrum of substrate **1a** and 10 mol% Fe(HMDS)$_2$ at t = 0 in toluene-d$_8$ with 1,3,5-Trimethoxybenzene as internal standard.

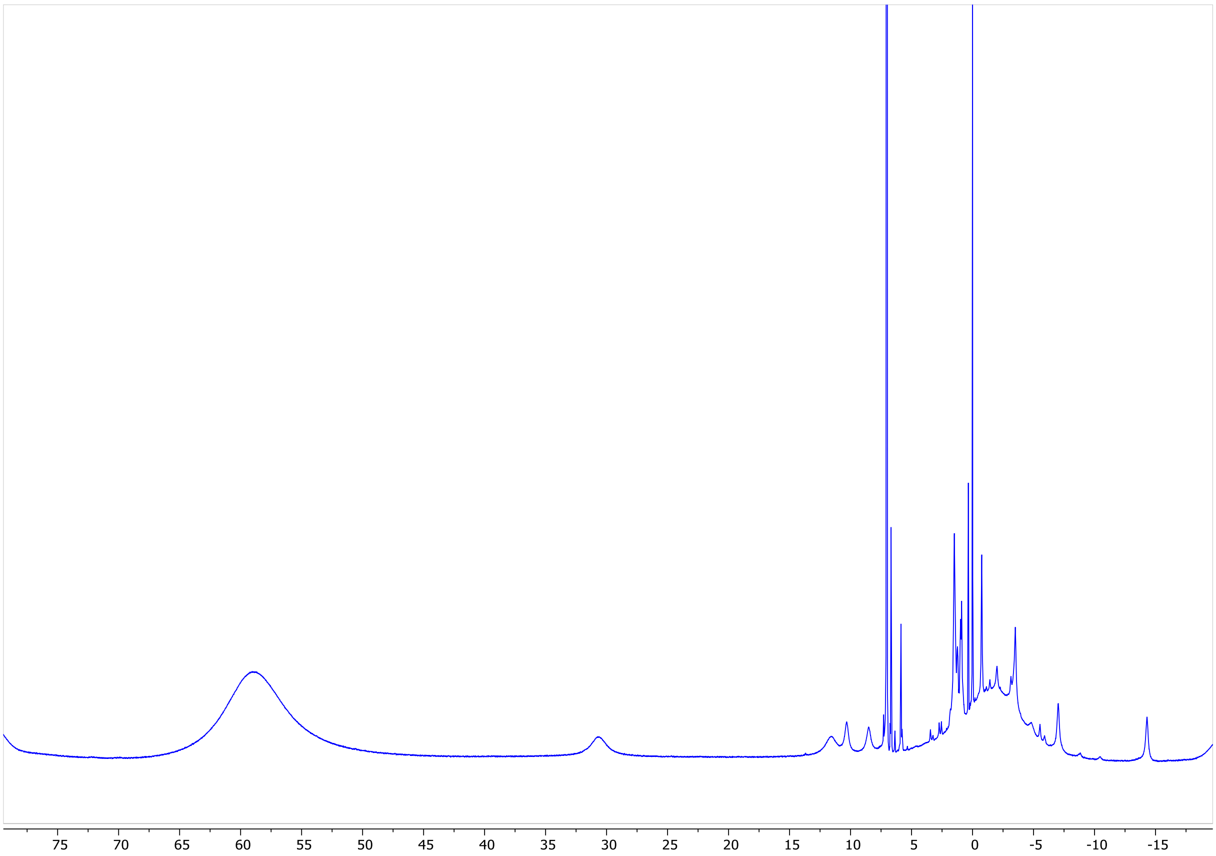

Figure S17: ^{1}H NMR spectra of the reaction with Fe(HMDS)$_2$ in C_6D_6 with 0.5 equivalents of substrate **1a** at t = 24 h.

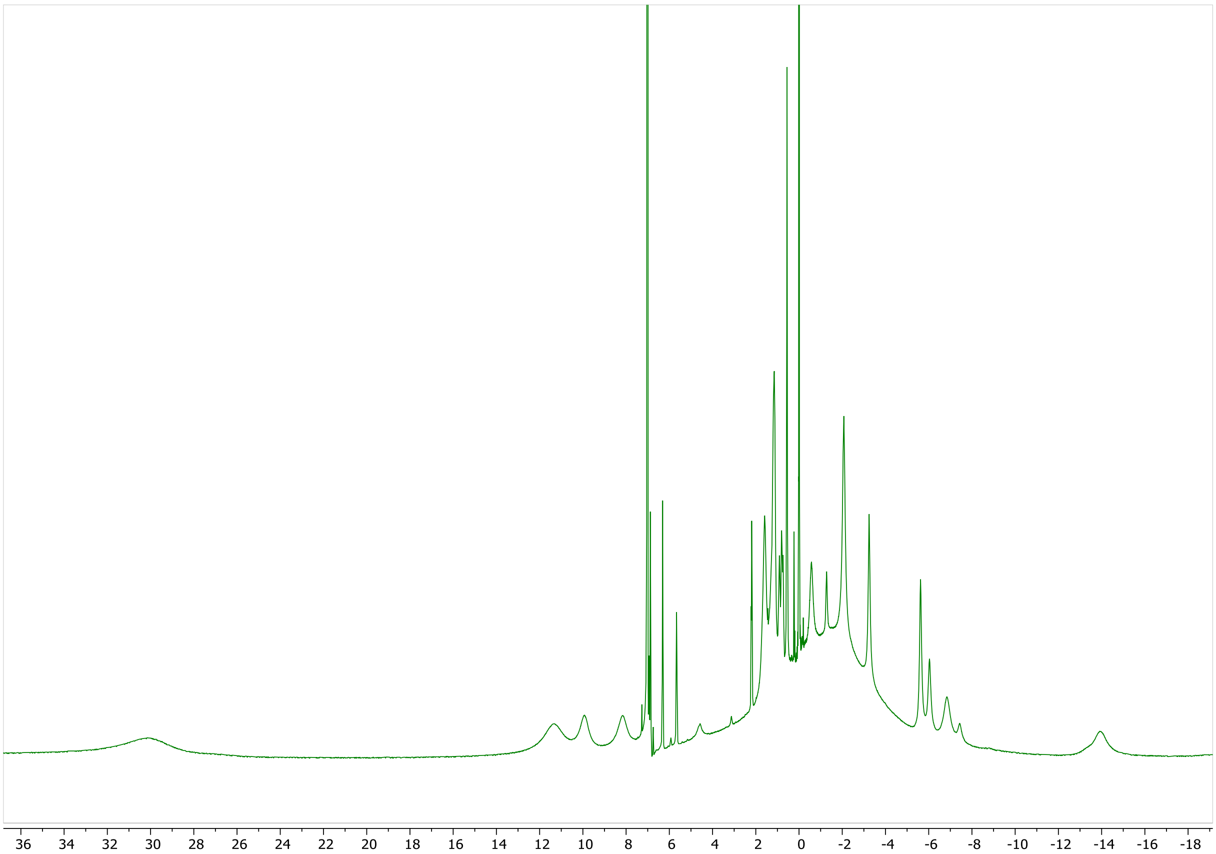

Figure S18: ^{1}H NMR spectra of the reaction with $Fe(HMDS)_2$ in C_6D_6 with 1.0 equivalents of substrate **1a** at t = 24 h.

FTIR-spectroscopy

Inside an argon filled glovebox $Fe(HMDS)_2$ (92.6 mg; 0.25 mmol; 1.0 eq) was dissolved in C_6H_6 (1 mL) and added to (4-azido-4-methylpentyl)benzene (**1a**) (0.050 mg; 0.25 mmol; 1.0 eq). Of the obtained yellow solution 0.5 mL was transferred into a Schlenk and taken outside the glovebox. An FTIR-spectrum was measured by inserting a fiber probe in the Schlenk through a septum. After leaving the unmeasured solution in the glovebox for 24 h, another 0.5 mL was taken outside the glovebox and an FTIR-spectrum was measured, according to the same method. Separately, an FTIR-spectrum was measured without the addition of $Fe(HMDS)_2$ using the same procedure.

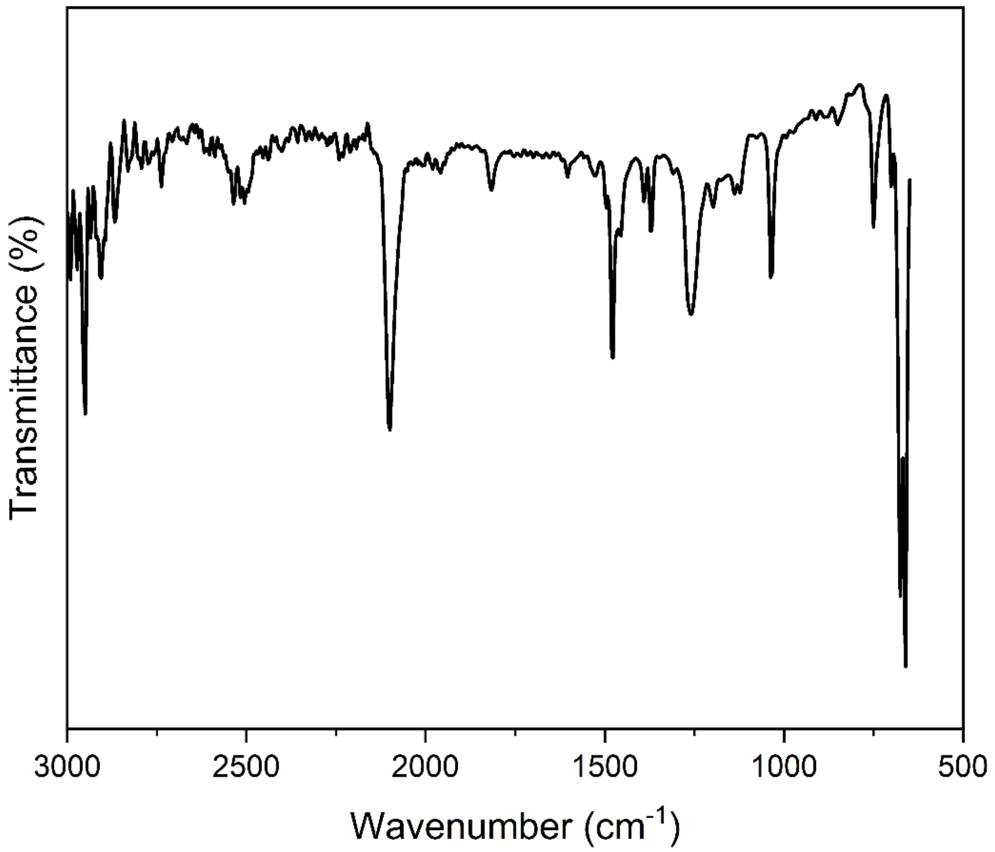

Figure S19: FTIR-spectrum of (4-azido-4-methylpentyl)benzene (**1a**) in C_6H_6.

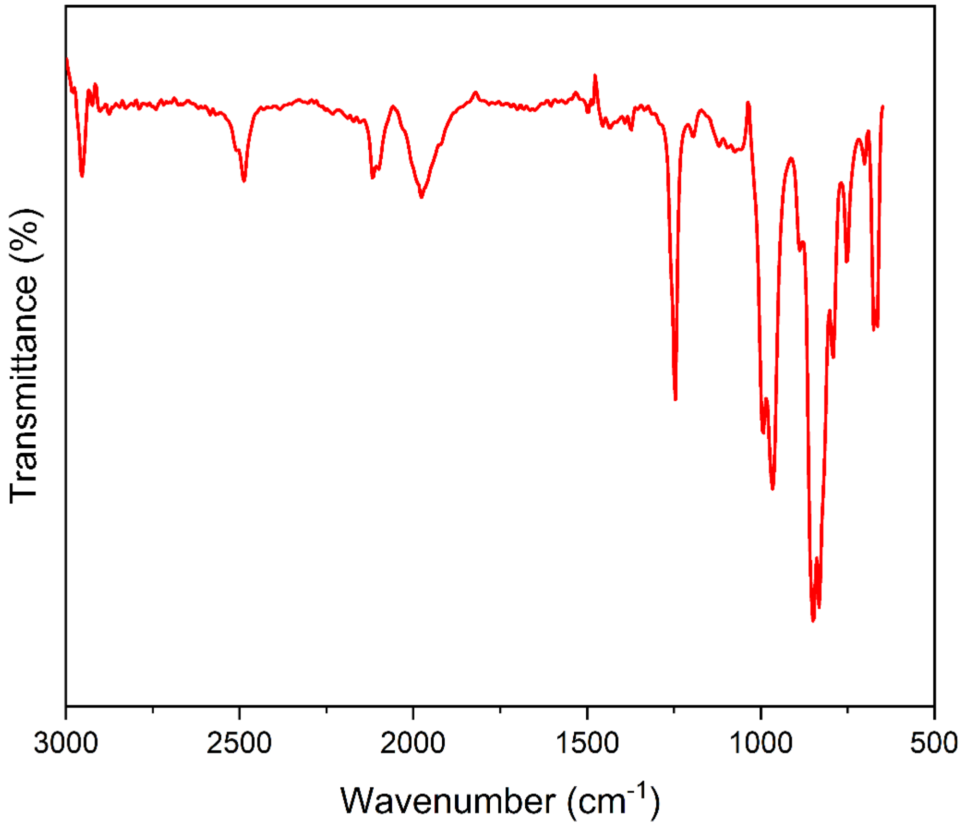

Figure S20: FTIR-spectrum of (4-azido-4-methylpentyl)benzene (**1a**) with $Fe(HMDS)_2$ in C_6H_6 at t = 0.

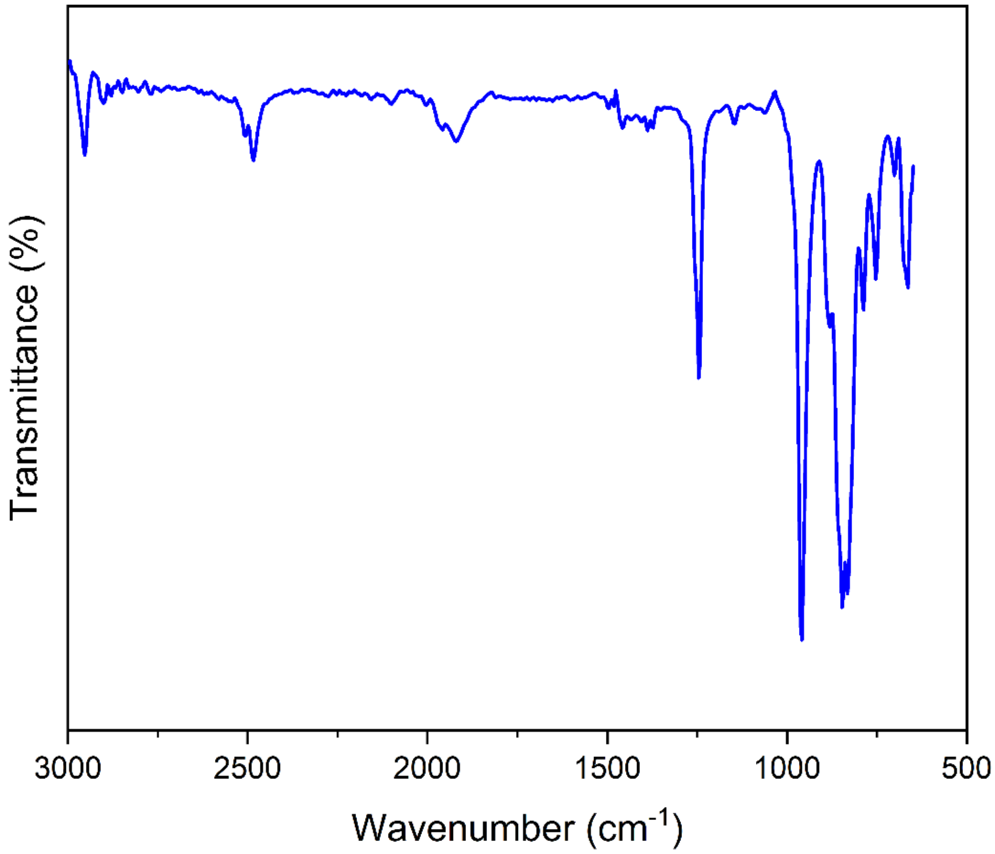

Figure S21: FTIR-spectrum of (4-azido-4-methylpentyl)benzene (**1a**) with $Fe(HMDS)_2$ in C_6H_6 at t = 24h.

Computational details

Density Functional Theory

Table S3: DFT calculated energies for non-metal containing compounds.

Compound	Multiplicity (S)	Energy (kcal/mol)
**i1a**	1	-396197.924
iN_2	1	-68734.09019
**i1b**	1	-327522.374
**i1b-d$_2$**	1	-327526.774
iHHMDS	1	-548231.8155

Table S4: DFT calculated energies and expectation values of the iron compounds on the catalytic cycle in various spin states.

Compound	Multiplicity (S)	$<S^2>$	Energy (kcal/mol)	Relative energy among different spin states (kcal/mol)
**Fe(HMDS)$_2$**	**5**	**6.01**	**-1888700.594**	**0.00**
***Anti*-i2**	**5**	**6.02**	**-2284897.486**	**0.00**
***Anti*-i2**	3	2.82	-2284870.613	26.87
***Anti*-TS-i2/i3**	**5**	**6.52**	**-2284872.517**	**0.00**
***Anti*-i3**	**5**	**6.42**	**-2216182.146**	**0.00**
***Gauche*-i3**	**5**	**6.48**	**-2216184.322**	**0.00**
***Gauche*-i3**	3	2.52	-2216180.732	3.59
***Gauche*-i3**	BS 5 (3,1)	2.79	-2216181.258	3.06
***Gauche*-i3**	7	12.03	-2216177.614	6.71
**TS-i3/i4**	**5**	**6.69**	**-2216173.076**	**0.00**
**i4**	**5**	**6.02**	**-2216231.094**	**0.00**
**i4**	3	2.75	-2216205.708	25.39

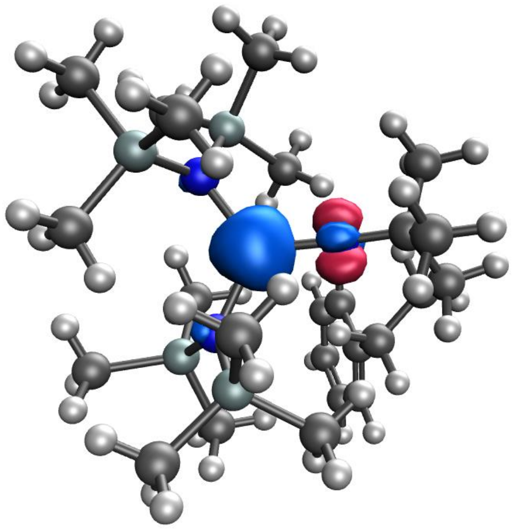

Figure S22: Spin density plot of ***Gauche*-i3** in the ground state (pentet) spin state, α-spin is represented in blue and β-spin in red.

Table S5: DFT calculated energies and expectation values of the iron compounds outside the catalytic cycle.

	Multiplicity (S)	$\langle S^2 \rangle$	Energy (kcal/mol)
***Gauche*-i2**	5	6.02	-2284899.143
***Gauche*-TS-i2/i3**	5	6.52	-2284872.517
**i17**	5	6.02	-1667989.562
**i18**	5	6.02	-1995516.626
**i19**	5	6.01	-1447274.357
**i20**	5	6.02	-1774802.592
**i4-d$_2$**	5	6.02	-2216235.688

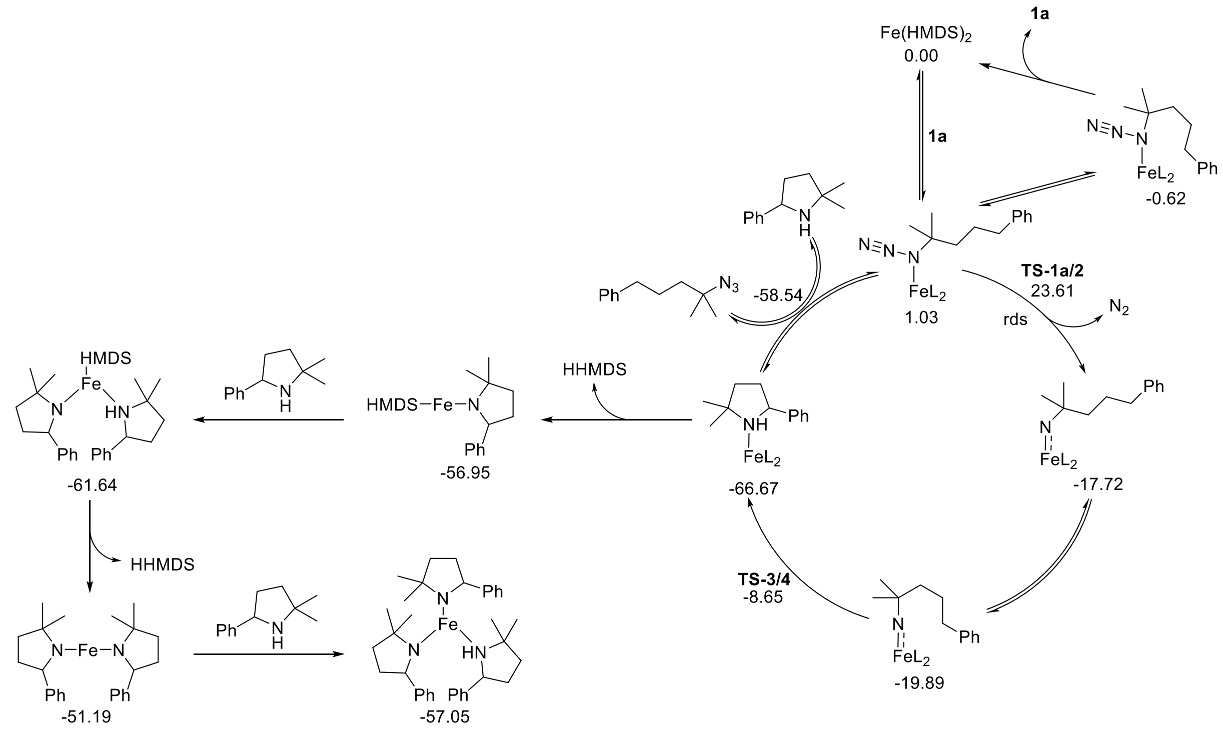

Scheme 1: Proposed reaction mechanism of the intramolecular C–H amination catalyzed by Fe(HMDS)$_2$, including the proposed decomposition pathway and off-cycle intermediates. Energies calculated by DFT are giving in kcal/mol.

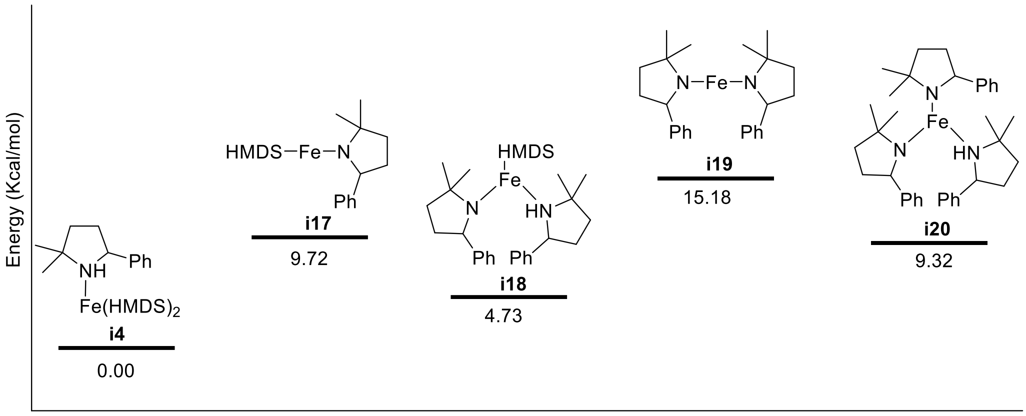

Scheme 2: Reaction coordinate profile of the proposed decomposition pathway. Energies calculated by DFT are giving in kcal/mol. Structures of the optimized intermediates and transition states are displayed in the SI.

We hypothesized a catalyst decomposition pathway, in which a coordinated HMDS group deprotonates the coordinated amine in the resting state **4**, leading to discoordination of H–N($SiMe_3$)$_2$ and pyrrolidinyl bonding to iron (**i17**). According to DFT calculations this transformation is 9.7 kcal/mol uphill, only slightly higher than the substitution with another substrate molecule and therefore potentially competitive as a decomposition pathway (Scheme S1-2). Coordination of a second **1b** to form **i18** is exergonic by 4.7 kcal/mol. Deprotonation of this amine by the remaining HMDS and subsequent decoordination of a second equivalent H–N($SiMe_3$)$_2$, leads to an iron complex **i19** and is 10 kcal/mol uphill. Coordination of yet another pyrrolidine **1b** stabilizes this compound by 5.9 kcal/mol and is predicted to yield iron complex **i20** with three coordinated pyrrolidine molecules, two of which deprotonated. Attempts to isolate and characterize this degradation product have not been successful so far, though the decoordinated H–N($SiMe_3$)$_2$ was unambiguously identified by ^{1}H NMR spectroscopy.

NEVPT2-CASSCF

```
----------------------------------------------
CAS-SCF STATES FOR BLOCK  1 MULT= 5 NROOTS= 1
----------------------------------------------

ROOT   0:  E=    -3521.8860454098 Eh
      0.54548 [   307]: 22211110
      0.17375 [   292]: 22111111
      0.07736 [   269]: 21211210
      0.07680 [   277]: 22011112
      0.03836 [   234]: 21111211
      0.03402 [   212]: 21011212
      0.02529 [   186]: 12211120
      0.00908 [   151]: 12111121
      0.00723 [   130]: 12011122
      0.00356 [    72]: 11111221
```

Figure S23: Configurations for **i3** on the pentet energy surface calculated by NEVPT2-CASSCF(10,8).

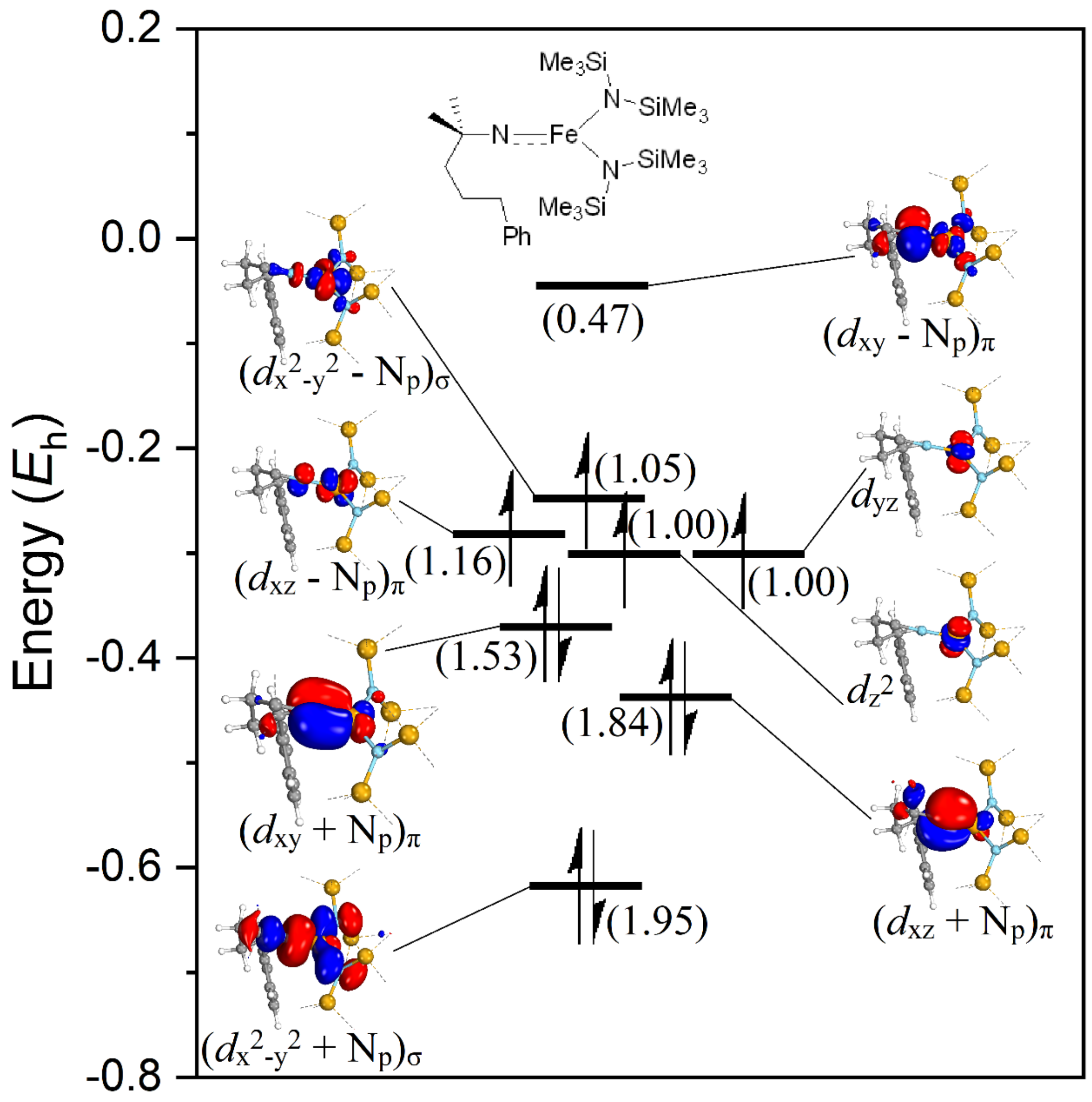

Figure S24: Active space orbitals from a NEVPT2-CASSCF (10,8) calculation on **i3**. Orbital filling of the main contribution (55%) is illustrated. Partial occupation due to multi-reference character described in brackets per orbital. Isosurface is set at 90. All Me-groups omitted for clarity.

NEVPT2-CASSCF (12,9) calculations on the DFT optimized **TS-i3/i4** were performed. Again, significant multi-reference character was observed with the main electronic configuration contributing 36% towards the ground state (Figure S23-24). The lowest energy doubly occupied orbital within the active space, displays a bonding combination of σ-symmetry of the benzylic carbon atom with the C–N bond of the nitrene, showing the concerted pathway of the C–N and N–H bond formation obtained from DFT calculations. The next higher MO displays a bonding combination between the p-orbitals of the nitrene and benzylic carbon with the s-orbital of the hydrogen, displaying the transition of the hydrogen atom transfer (HAT). Higher in energy, partially filled orbitals within the active space consist of anti-bonding combinations between the C–N–H interactions and non-bonding metal d-orbitals. Multi-reference character shifts significant electron density in the LUMO (occupancy 0.59), leading to spin density at both the nitrene moiety and the benzylic carbon. This orbital displays a bonding interaction between the C–H interaction and the N–H interaction of opposite sign. Occupancy of this orbital hence facilitates the HAT.

```
---------------------------------------------
CAS-SCF STATES FOR BLOCK  1 MULT= 5 NROOTS= 1
---------------------------------------------

ROOT   0:  E=   -3521.8292053472 Eh
      0.36228 [   713]: 222211110
      0.29180 [   698]: 222111111
      0.07318 [   675]: 221211210
      0.05072 [   683]: 222011112
      0.03561 [   640]: 221111211
      0.03067 [   701]: 222111210
      0.02622 [   396]: 122211120
      0.01487 [   361]: 122111121
      0.01299 [   618]: 221011212
      0.01280 [   591]: 212211111
      0.00965 [   695]: 222111012
      0.00615 [   556]: 212111112
      0.00530 [   340]: 122011122
      0.00467 [   704]: 222112110
      0.00371 [   637]: 221111112
      0.00354 [   672]: 221211111
      0.00321 [   319]: 121211220
      0.00300 [   477]: 211111212
      0.00280 [   699]: 222111120
      0.00256 [   685]: 222011211
```

Figure S25: Configurations for **TS-i3/i4** on the pentet energy surface calculated by NEVPT2-CASSCF(12,9).

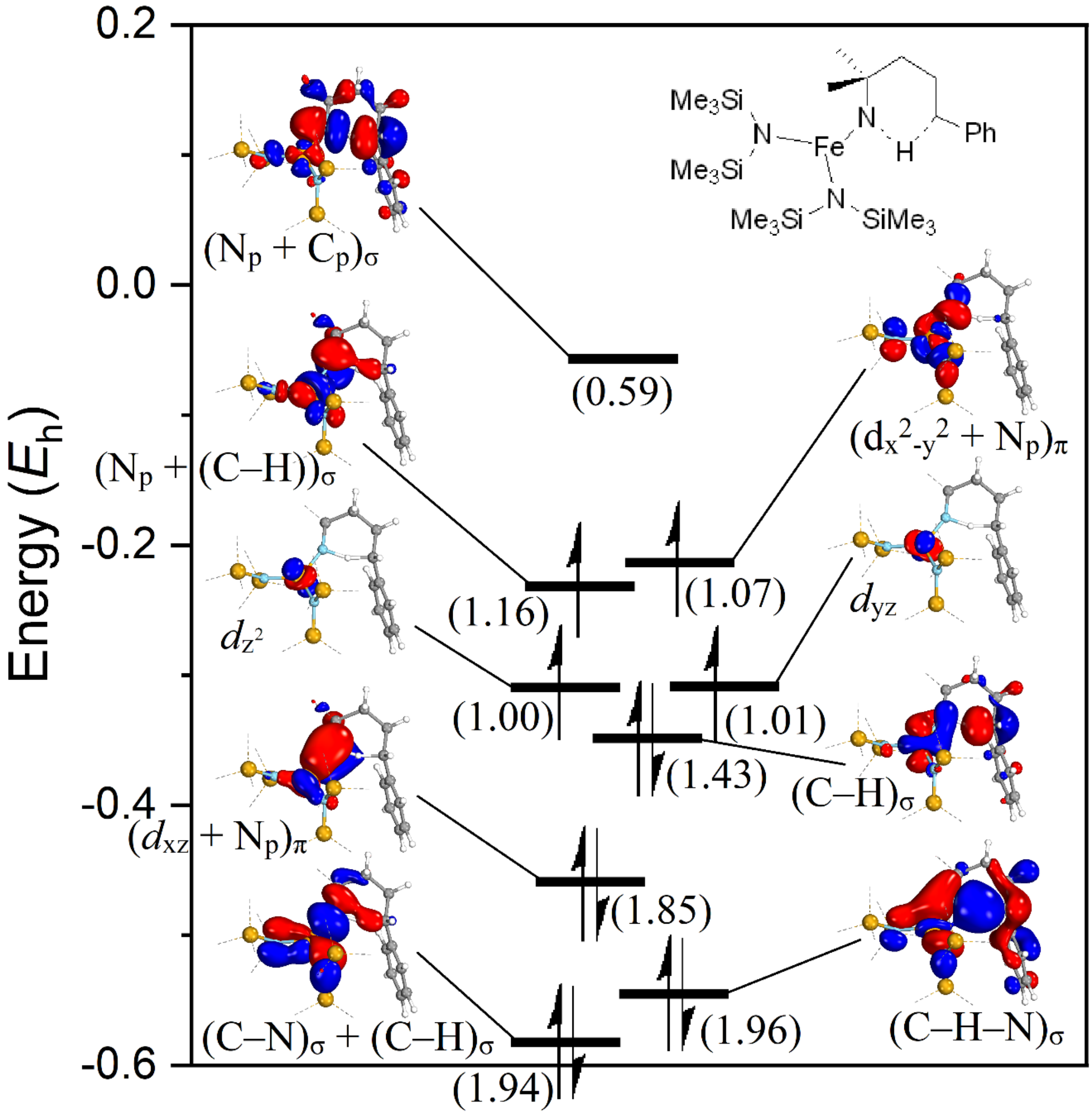

Figure S26: Active space orbitals from a NEVPT2-CASSCF (12,9) calculation on **TS-i3/i4**. Orbital filling of the main contribution (36%) is illustrated. Partial occupation due to multi-reference character described in brackets per orbital. Isosurface is set at 90. All Me-groups omitted for clarity.

DFT calculated structures

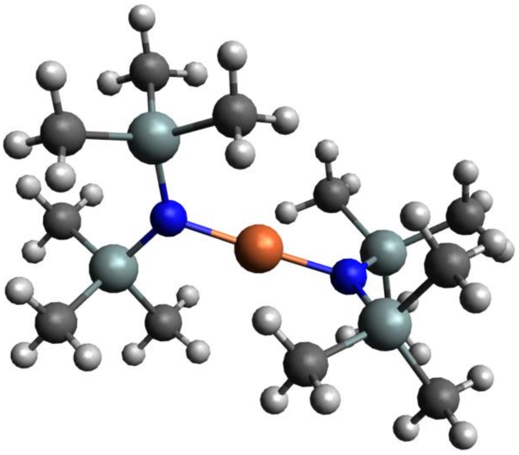

Figure S27: Geometry optimized structure of $Fe(HMDS)_2$ by DFT (B3LYP/def2-TZVP) in the pentet spin state. Orange = Fe, blue = N, mint = Si, grey = C, off-white = H.

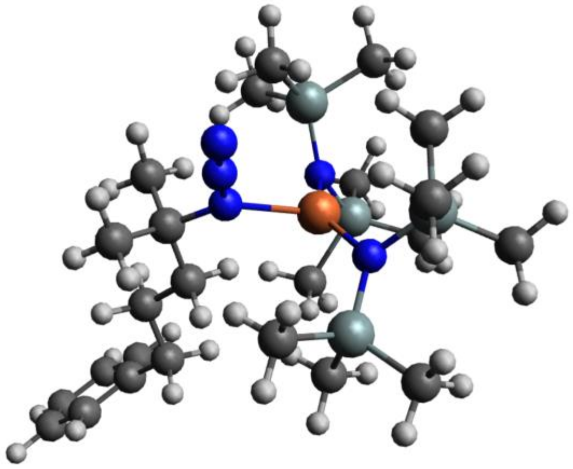

Figure S28: Geometry optimized structure of ***Anti*-i2** by DFT (B3LYP/def2-TZVP) in the pentet spin state. Orange = Fe, blue = N, mint = Si, grey = C, off-white = H.

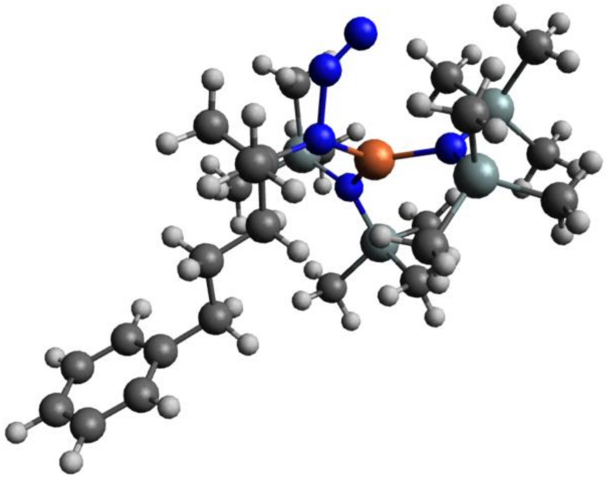

Figure S29: Geometry optimized structure of ***Anti*-TS-i2/i3** by DFT (B3LYP/def2-TZVP) in the pentet spin state. Orange = Fe, blue = N, mint = Si, grey = C, off-white = H.

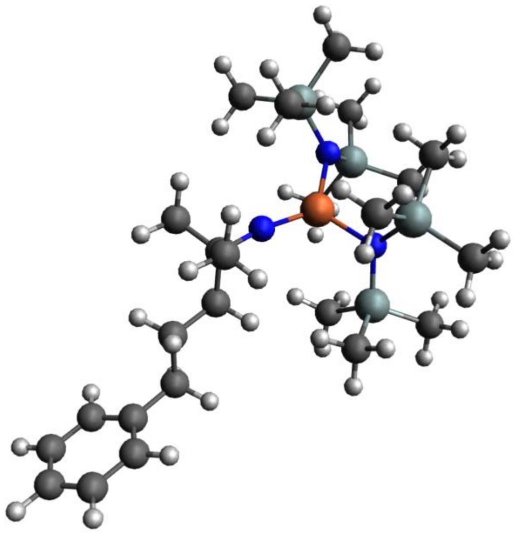

Figure S30: Geometry optimized structure of ***Anti*-i3** by DFT (B3LYP/def2-TZVP) in the pentet spin state. Orange = Fe, blue = N, mint = Si, grey = C, off-white = H.

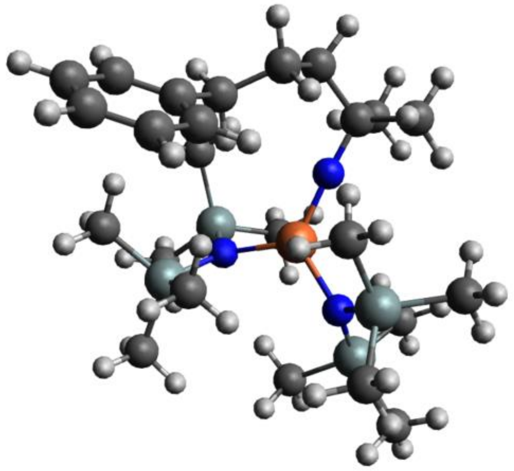

Figure S31: Geometry optimized structure of ***Gauche*-i3** by DFT (B3LYP/def2-TZVP) in the pentet spin state. Orange = Fe, blue = N, mint = Si, grey = C, off-white = H.

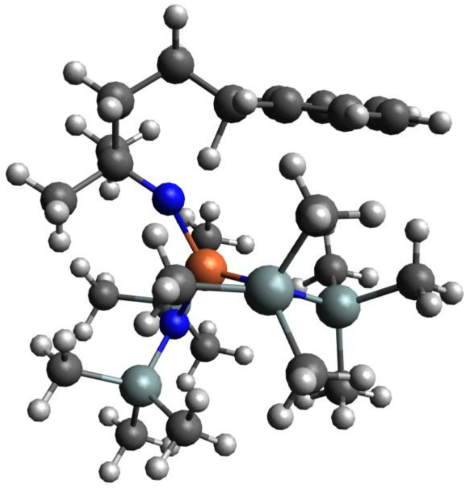

Figure S32: Geometry optimized structure of **TS-i3/i4** by DFT (B3LYP/def2-TZVP) in the pentet spin state. Orange = Fe, blue = N, mint = Si, grey = C, off-white = H.

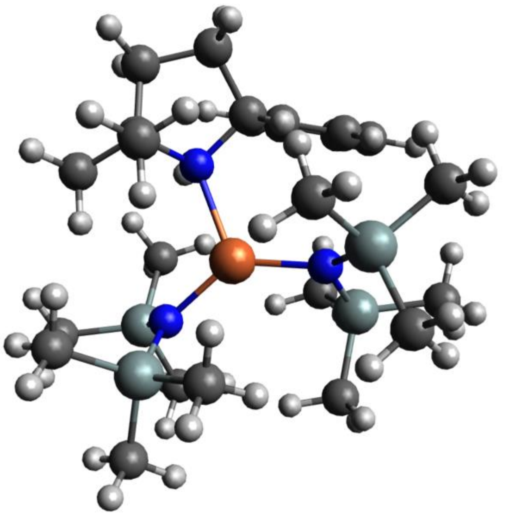

Figure S33: Geometry optimized structure of **i4** by DFT (B3LYP/def2-TZVP) in the pentet spin state. Orange = Fe, blue = N, mint = Si, grey = C, off-white = H.

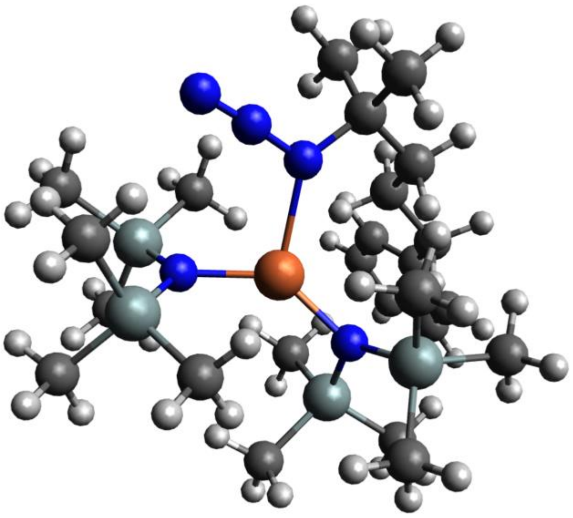

Figure S34: Geometry optimized structure of ***Gauche*-i2** by DFT (B3LYP/def2-TZVP) in the pentet spin state. Orange = Fe, blue = N, mint = Si, grey = C, off-white = H.

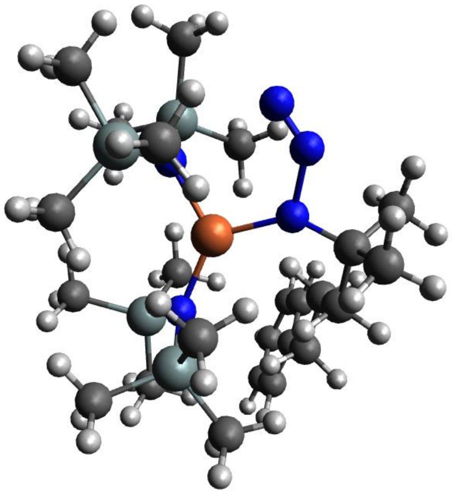

Figure 35: Geometry optimized structure of ***Gauche*-TS-i2/i3** by DFT (B3LYP/def2-TZVP) in the pentet spin state. Orange = Fe, blue = N, mint = Si, grey = C, off-white = H.

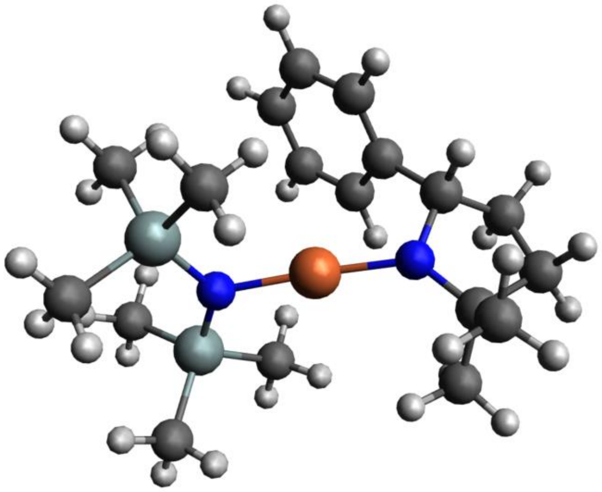

Figure S36: Geometry optimized structure of **i17** by DFT (B3LYP/def2-TZVP) in the pentet spin state. Orange = Fe, blue = N, mint = Si, grey = C, off-white = H.

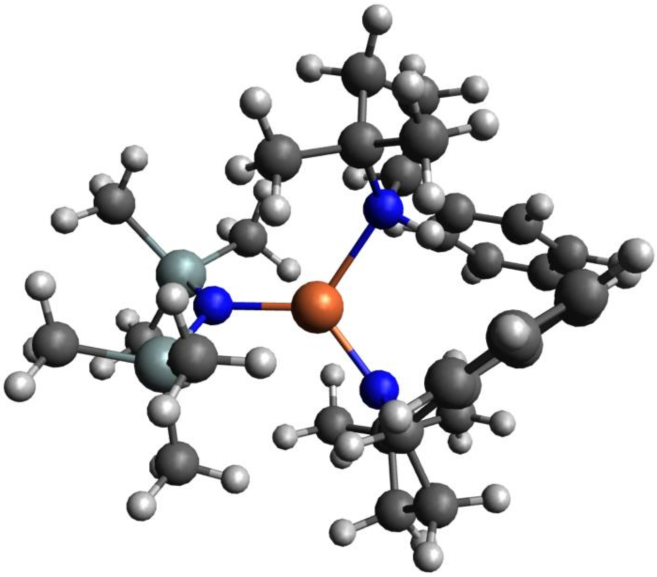

Figure S37: Geometry optimized structure of **i18** by DFT (B3LYP/def2-TZVP) in the pentet spin state. Orange = Fe, blue = N, mint = Si, grey = C, off-white = H.

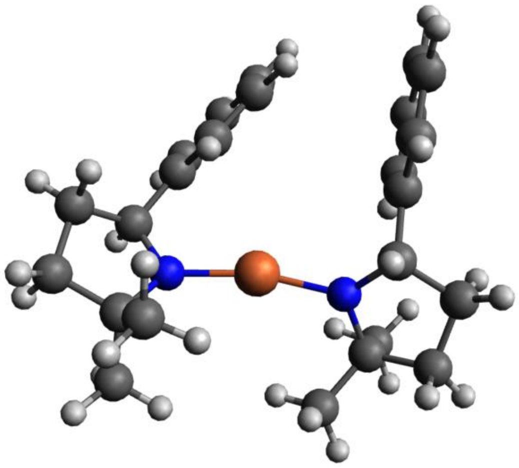

Figure S38: Geometry optimized structure of **i19** by DFT (B3LYP/def2-TZVP) in the pentet spin state. Orange = Fe, blue = N, mint = Si, grey = C, off-white = H.

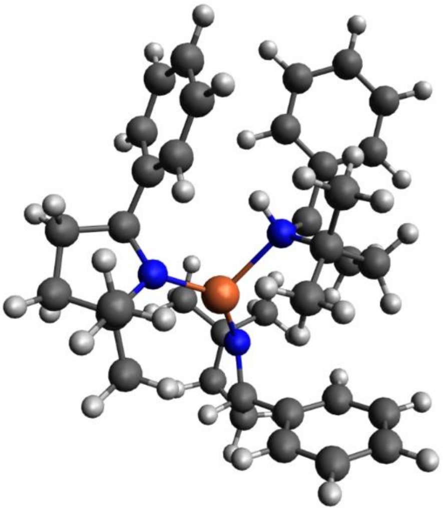

Figure S39: Geometry optimized structure of **i20** by DFT (B3LYP/def2-TZVP) in the pentet spin state. Orange = Fe, blue = N, mint = Si, grey = C, off-white = H.

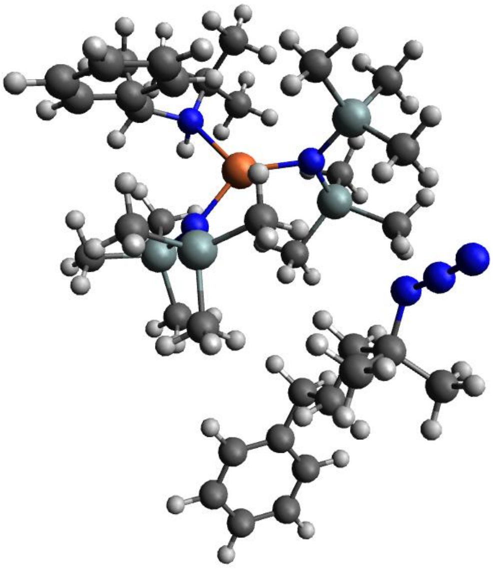

Figure 40: Geometry optimized structure of **i4** with coordinated **1a** by DFT (B3LYP/def2-TZVP) in the pentet spin state. Decoordination of **1a** occurs during the optimization cycles. Orange = Fe, blue = N, mint = Si, grey = C, off-white = H.

DFT calculated coordinates

$Fe(HMDS)_2$

Fe	-0.42587249019967	1.00900743186828	-0.39844950110438
N	-0.70546999354268	2.62440226271172	0.51743412007911
N	-0.09881036003191	-0.62774584560694	-1.26029961194515
Si	-2.14278800285869	3.47555316100229	0.09093095347191
C	-3.47437387347352	3.26742215837428	1.40027414106141
C	-2.78303725583558	2.73907329644076	-1.52702331507815
C	-1.82088877929053	5.30795138919617	-0.17261199665504
Si	0.52332080878579	3.05634733800026	1.64758501170223
C	1.49481323895441	1.48940497088558	2.05815831830953
C	-0.20151586757439	3.75430526038770	3.23413007837850
C	1.71379893533275	4.31116418299334	0.91273446011661
Si	0.71448172182398	-0.53256789217911	-2.77695736740190
C	1.37132609799793	1.22808448294263	-2.96701391318531
C	-0.45153060996199	-0.88406506775107	-4.20797086644627
C	2.16188389565091	-1.72748781991408	-2.86962845382693
Si	-0.60532196240412	-2.02194942996221	-0.37949381010435
C	-1.87689044835615	-1.45343912593293	0.89663577038833
C	0.83225109528511	-2.81578144915132	0.53430543074564
C	-1.40167725093761	-3.31105618519436	-1.49091664213621
H	0.57379446794572	1.97674156456496	-2.91206235376485
H	1.85375882759626	1.35632180664560	-3.93968492944560
H	2.11378692216162	1.47009426795687	-2.20183829655134
H	2.87080945730023	-1.55078861205534	-2.05770392407317
H	2.69853129299926	-1.62093632213775	-3.81599837336737
H	1.82549092506341	-2.76468100551097	-2.79777537688967
H	1.30812479919669	-2.09739340799922	1.20644678399669
H	1.59407619469557	-3.17107947844366	-0.16283673885581
H	0.50223594898639	-3.66904927509670	1.13290497037328
H	-1.48433331878989	-0.67793089100657	1.56379255273402
H	-2.18364648186214	-2.28617478851104	1.53520454963069
H	-2.77645347040022	-1.05912193766112	0.41706770878914
H	0.87182564856260	0.75830348439631	2.57953339371243
H	2.34657929640277	1.71615554004206	2.70489434527297
H	1.90299617683723	1.00421689076225	1.16427559394163
H	-0.89002725462545	3.04434683150403	3.69762007179745
H	-0.75283378386842	4.67881656389529	3.04656220096145
H	0.58545368749162	3.98267886639559	3.95761682931718
H	-1.47441203657710	5.78810027600187	0.74567161905367
H	-2.73062670315987	5.82443159715856	-0.48995631557272
H	-1.05893919408880	5.46555822569193	-0.93914761409040
H	-3.69798617606619	2.20911911593905	1.55629597079273
H	-4.40248757413919	3.76949098517695	1.11431262338688
H	-3.15145938590952	3.68133485180318	2.35782947498413
H	-2.94329667264353	1.65684737192544	-1.46111191429514
H	-2.09487673848667	2.92739391331915	-2.35515034213114
H	-3.74683121383892	3.17899409334496	-1.79707269405845
H	-2.25375339059986	-2.89351915206576	-2.03174670264295
H	-1.75803457127092	-4.16277372866781	-0.90566962350959
H	-0.69338962666788	-3.69435963899189	-2.22950764244869

H	-1.28849261834332	-0.18133532800499	-4.20805681316442
H	-0.86617067941511	-1.89184021669627	-4.13892163243997
H	0.06031476416003	-0.79830919188740	-5.17023321565769
H	2.16932002406847	3.91891677130200	-0.00011014596998
H	2.51780475139410	4.55923568133192	1.61075966016623
H	1.19839880652704	5.23804115646739	0.65208349364886

***Anti*-i2**

Fe	-0.77670658948903	0.85531336782510	0.37368999194961
N	-0.82040772889697	2.67862083172624	-0.23306947683774
N	-0.93109020509628	-0.98068585695592	-0.16329044367715
Si	-2.31922906712646	3.17413440398177	-0.92932934543835
C	-3.53127292565615	1.73156240141670	-0.88053372815576
C	-2.15905541961325	3.67236452549467	-2.73686864810971
C	-3.09454822827386	4.59497976091707	0.03400979881999
Si	0.53941894783789	3.70591829119347	0.01084679470143
C	2.13542689868156	2.79132206922994	-0.39813530622558
C	0.62454960769057	4.28682322649392	1.80401007882959
C	0.52988187540523	5.25281040170701	-1.06332234037429
Si	0.09146300476966	-1.34646771368075	-1.51162581562678
C	1.90623941295139	-1.14533298361165	-1.03937755990164
C	-0.27446994355831	-0.16877164006627	-2.93428836633851
C	-0.07667788798968	-3.11131378015774	-2.14878398915496
Si	-2.25101917425173	-1.94695823050725	0.38289665190446
C	-3.54188995447900	-2.22670139699128	-0.96126290575859
C	-3.16553860287124	-1.12643359528709	1.81580019181340
C	-1.68273148199111	-3.63378458017690	1.00408685346214
H	2.15644226295772	-0.11395811762051	-0.78686079180887
H	2.55391613506549	-1.44066732435247	-1.86925447584664
H	2.15285912461995	-1.77779075760710	-0.18277312939567
H	0.26631409139620	-3.83890669650462	-1.41098490804734
H	0.54895342429936	-3.22550577701557	-3.03833081426106
H	-1.09751748717133	-3.37150528878244	-2.43032316212179
H	-3.50012697963990	-0.11569571037438	1.57631885601057
H	-2.56391183534663	-1.08089273509766	2.72250590315152
H	-4.05969244488474	-1.71148978419082	2.04799689084576
H	-4.03313780545281	-1.28930207231221	-1.22940672468211
H	-4.31455770974276	-2.91828849477378	-0.61388213738204
H	-3.10801261921336	-2.64630693721852	-1.87005886191703
H	2.28594814955636	1.90865923814457	0.22348537023871
H	3.00366610464208	3.44056761242832	-0.25589868552180
H	2.12928641871597	2.46320728854400	-1.44011161236876
H	0.69889560954352	3.44251555373600	2.49124117652715
H	-0.27755897701617	4.84479327634755	2.06635969363449
H	1.48588403595247	4.93694463438532	1.97896570693607
H	-3.26290042396235	4.30611580832795	1.07474662346818
H	-4.05885707220458	4.88227571043998	-0.39380205400374
H	-2.45720361757662	5.48161419427856	0.03638523062954
H	-3.12963275359525	0.84777576609147	-1.38034941258670
H	-4.45338886318788	2.00713948268266	-1.39930146661436
H	-3.80864119764321	1.45065414463929	0.13749714595513

H	-1.69968549639809	2.86812409143196	-3.31647621931923
H	-1.55151303343443	4.56839375220495	-2.86609159721365
H	-3.14415445393384	3.87406300572346	-3.16680239070238
H	-1.30813734340087	-4.25789213515526	0.19182373530752
H	-2.51411269659186	-4.16719845587491	1.47329705731593
H	-0.88606204469782	-3.54215321929422	1.74596596894901
H	-0.13645474595583	0.87113631713282	-2.62769661259792
H	-1.30913069721457	-0.27831654784483	-3.26880661419956
H	0.37837674180031	-0.35573626326059	-3.79101038943336
H	0.59511848142064	5.00650607575660	-2.12450071694776
H	1.39678007457763	5.87018101493264	-0.81184973369354
H	-0.36178776131643	5.86359190292483	-0.91286869601812
N	-0.58198448709316	0.99903991185609	2.56700432166524
N	-1.49218890338277	1.59377407332622	3.14727596375093
C	0.48902280238823	0.34331747190020	3.43071389882633
N	-2.35589234775592	2.16588337223457	3.58611861932117
C	0.03128596479750	-1.07722687957432	3.74647041589187
C	0.70470621129175	1.15484700147656	4.70408147878674
C	1.72901272422814	0.36120458138769	2.52951802949217
H	-0.91398012343825	-1.07005227428315	4.29034804946258
H	0.77095613265735	-1.57932527494548	4.36995055205589
H	-0.09843733825517	-1.64979789438782	2.82945443045265
H	-0.19009992492375	1.17108595117519	5.32862483256374
H	0.99054435899155	2.18207197513396	4.47489650981577
H	1.50114656779371	0.70166318730671	5.29235394732835
H	1.44419134935609	-0.05226744668333	1.55796086740523
C	2.93560097124877	-0.42055063922444	3.03909713327510
H	2.01181741577930	1.40265999510911	2.36432102362089
H	2.70471122470446	-1.48720043306833	3.07933535653758
H	3.19494647990417	-0.11942040005099	4.05751406897272
C	4.16295541277890	-0.21666372784763	2.13851856158355
H	3.89985127005250	-0.47589235356457	1.11088605990264
C	5.33468892859635	-1.04778673532203	2.58770795486235
H	4.43465602490311	0.84254082680969	2.13705111009822
C	6.22415802377321	-0.57186819292685	3.55094930438587
C	7.27463562309912	-1.36067893781147	4.00441092769728
C	7.45222898411396	-2.64351834585960	3.49859291063396
C	5.52621583789794	-2.33496132011829	2.08562179164461
C	6.57485908605933	-3.12822788762502	2.53520688011980
H	7.95754501093226	-0.97230966014284	4.74963241450019
H	8.27159414549775	-3.25831766990717	3.84847329597013
H	4.84948425697965	-2.71591544298985	1.32925825032028
H	6.70980871399268	-4.12305690902779	2.12953959328654
H	6.09479447002142	0.42964802222070	3.94581085760158

***Anti*-TS-i2/i3**

Fe	-0.66255935001556	1.14902472029592	0.61759446655280
N	-0.99135379581392	2.92068380060134	-0.01689045347442
N	-0.60271941275959	-0.54973186077364	-0.25243730357395
Si	-2.47719576609633	3.10455873462075	-0.89001632211864
C	-3.54356525040645	1.57193534889436	-0.62815927368913

C	-2.19385970726060	3.30273461065258	-2.73851464440723
C	-3.46682414434538	4.57544671690278	-0.26152948263085
Si	0.08957129541612	4.23345699750082	0.32590662256145
C	1.84807717919682	3.61744287632641	0.59127362491940
C	-0.41312984504398	5.17889108059895	1.87256500095129
C	0.16574093262512	5.45520302591494	-1.10553034856275
Si	0.24198309800512	-0.60894091116782	-1.76611616865428
C	1.37581816063786	0.88564782651385	-1.90098790417825
C	-0.93181005673505	-0.59087056085707	-3.23443648670094
C	1.32372185946702	-2.14476953847679	-1.88892078086245
Si	-1.43479737752479	-1.90793170786107	0.42673487552079
C	-2.38673428321176	-2.87621164872133	-0.87664931451404
C	-2.70017542397074	-1.33089542144801	1.69740577073483
C	-0.24963915997825	-3.11792932720387	1.25557370047264
H	0.82225770995047	1.82583506584096	-1.88308264631055
H	1.93441245343095	0.84602451456585	-2.83977761054309
H	2.10565028930200	0.90488778150699	-1.08775071608268
H	2.03536359053111	-2.18783085889225	-1.06145982710603
H	1.89566812472760	-2.13037496274796	-2.82052000776437
H	0.73902613291436	-3.06646831434089	-1.87725767069160
H	-3.51867208019000	-0.79242102950095	1.21626176260274
H	-2.27374400027676	-0.68251330163519	2.46298070809928
H	-3.13261571409710	-2.19597325869140	2.20730809487283
H	-3.10647283585479	-2.23982553428536	-1.39554267329378
H	-2.94152449675681	-3.69024653327777	-0.40197698711579
H	-1.73066298844451	-3.32152842774800	-1.62687939002904
H	1.89571026031123	2.86349284813754	1.37686693144984
H	2.48343886814529	4.45192078040495	0.90027974175111
H	2.27542907017176	3.19327543726733	-0.31806835057847
H	-0.27289858543391	4.57377039610719	2.76973645319625
H	-1.46105060996164	5.48091495386090	1.83454652837152
H	0.19460569857154	6.08117681638765	1.98306351791361
H	-3.69241734160968	4.46036382644317	0.80097404133074
H	-4.41447769443594	4.65668802135743	-0.80075753644833
H	-2.93298705651727	5.51873422458291	-0.38936237245091
H	-3.07397901090361	0.66094791907667	-1.00310681471037
H	-4.49248386962095	1.69481201016463	-1.15738111297904
H	-3.78386001768832	1.42549127973140	0.42839837679959
H	-1.54828076091244	2.51163012808991	-3.12451091296656
H	-1.72209327310787	4.25824208400987	-2.97194251223133
H	-3.14079542125301	3.25642910682631	-3.28318802820173
H	0.46648599320837	-3.51883675137181	0.53621769181813
H	-0.80115622124921	-3.96047852194954	1.68163876006484
H	0.32021759270855	-2.65483380409678	2.06157981875590
H	-1.63390751797993	0.24235931660695	-3.17178019521115
H	-1.51493140748807	-1.51094842573739	-3.29304148242552
H	-0.37702072911147	-0.48848493954722	-4.17115938544014
H	0.48487099158266	4.96226948451834	-2.02630155484643
H	0.89149310378588	6.24136108859304	-0.87980932686171
H	-0.79215028524025	5.94051100804775	-1.29989399832403
N	-0.22472041435817	1.25899359657304	2.46097965982275

N	-1.35797093199517	1.96280579216587	3.36373831922483
C	0.73285759912192	0.61106268882915	3.36315530099106
N	-2.23591090101591	2.63555708717030	3.20314289316330
C	0.06672557015595	-0.47320432522520	4.21656299658896
C	1.40470578048303	1.65337914094635	4.26392016689721
C	1.76236722885642	-0.00213544392538	2.37791788009205
H	-0.72940615092436	-0.03298069860926	4.81865894490997
H	0.78413981446266	-0.93144704379605	4.89732904131545
H	-0.36762011752328	-1.25349060118451	3.59512706003256
H	0.67154565861036	2.10383892052192	4.93567583256369
H	1.85349689554242	2.44687857571124	3.66638016651627
H	2.18062386130261	1.19612366604744	4.87851195107791
H	1.21957342767687	-0.58503327167572	1.63397912376257
C	2.83442300211395	-0.89488853479635	2.99338180030905
H	2.24689452574680	0.81578388756630	1.83879847331618
H	2.37681305748106	-1.73615975252018	3.51801404286921
H	3.42935201117656	-0.35218424348295	3.73207087881972
C	3.76615047113502	-1.45060670945829	1.90618911443221
H	3.15919156545040	-1.95355430193735	1.14851050823533
C	4.78190350172161	-2.41248899139223	2.46031183527110
H	4.27113497227685	-0.62036264018204	1.40506741233259
C	6.02715473391895	-1.96684514303205	2.90242713111107
C	6.94494995633175	-2.84940567388587	3.45984800599196
C	6.62894551084807	-4.19736564726446	3.58416999302913
C	4.47743194702930	-3.76793604487900	2.58945068238244
C	5.39125223047549	-4.65472481745526	3.14546331210860
H	7.90857330074844	-2.48512439597491	3.79342571561083
H	7.34355044097702	-4.88723458474807	4.01444274140832
H	3.51529613625039	-4.13080503607234	2.24588165755311
H	5.13881110393945	-5.70410204463841	3.23274398306627
H	6.28220729858947	-0.91771160001441	2.80385648243379

***Anti*-i3**

Fe	-0.48413788983541	0.06408488475503	-0.10635571053331
N	-0.46336670954451	1.88088406440900	-0.63616814227310
N	-1.59015361969074	-1.08481240287668	-1.14301417812094
Si	-1.95034049220983	2.73515004566778	-0.32325706653912
C	-2.28969321197542	2.71912553112865	1.53010826332878
C	-3.39995359441597	1.94611371950685	-1.21471545474592
C	-1.87293901550127	4.53061495799067	-0.87673130774047
Si	1.09558750682398	2.62697916870363	-0.78867089194082
C	2.41134488934048	1.31583091154617	-1.10640145572395
C	1.54911004237267	3.58900417276941	0.76656864469141
C	1.18634031231274	3.79173314542458	-2.26198372062676
Si	-1.10131137903144	-1.26275839547560	-2.79834385805319
C	0.72822374707140	-1.71615677798086	-2.82961122749849
C	-1.34515446364102	0.32448239180396	-3.76968718725298
C	-2.01924098428761	-2.63536054065309	-3.70005867372452
Si	-2.69568036560748	-2.07459581601097	-0.24937376976292
C	-3.14409692912847	-1.21854524395629	1.36959669982962
C	-1.92671591988961	-3.74103390186934	0.16273547156797

C	-4.31443660786694	-2.34382270300051	-1.16917382455894
H	1.35075768885778	-0.98310785347734	-2.31292093292269
H	1.10006079154159	-1.78879811615906	-3.85526972641087
H	0.88798340504665	-2.68147455489534	-2.34291120347247
H	-1.96560761681238	-3.59109420452431	-3.17510000446108
H	-1.56080073217238	-2.77417256597563	-4.68324961883411
H	-3.07025125846362	-2.39089690759240	-3.85753207997145
H	-1.00754476454941	-3.60337495924112	0.73716614844984
H	-1.67086821288949	-4.29269647198479	-0.74474432247365
H	-2.60384157992623	-4.36408432728979	0.75305100583621
H	-2.26902006630149	-1.02814265444359	1.99299561534594
H	-3.82722209815568	-1.85213152252471	1.94168578675633
H	-3.65081550844393	-0.26698679017823	1.19470061387044
H	2.35180771072709	0.45212363747729	-0.44320295403249
H	3.40163711579464	1.75879402130371	-0.96820388743234
H	2.35834776134236	0.95998253344140	-2.13711519521856
H	1.51503167570320	2.95120598417660	1.65169297719704
H	0.85453721625615	4.41549319523521	0.93146019422890
H	2.55473994778978	4.01132660220255	0.69310130305066
H	-1.08648651192405	5.09939503092078	-0.37839291820311
H	-2.82659044149315	5.00863969405283	-0.63598602211647
H	-1.72408408742353	4.61277886831817	-1.95437474390781
H	-2.37447344526843	1.70241187862710	1.91947233523723
H	-3.21934211009034	3.24265829228097	1.76859440116820
H	-1.47723938408340	3.20948566389160	2.07144117151025
H	-3.46657428384298	0.87527056179954	-1.02914047251288
H	-3.30700306455668	2.08380833760579	-2.29322473875639
H	-4.33507644362370	2.41246289207274	-0.89327911180936
H	-4.69919921178385	-1.40238383022638	-1.56794734122821
H	-5.06421871072615	-2.74506950241548	-0.48180219780348
H	-4.21544373677479	-3.04535338313805	-1.99691029075131
H	-0.88180767838021	1.17105101212759	-3.26134998040074
H	-2.40760309878210	0.54699546911801	-3.88713642072318
H	-0.90813069606112	0.23663199742076	-4.76792625862783
H	0.81790089404548	3.30144231120469	-3.16601765688903
H	2.22835147325938	4.07376852411293	-2.43855386322905
H	0.61496783506206	4.70762380436431	-2.11767842654259
N	0.30040147630222	-0.38726200562820	1.36436288795471
C	0.97894079048266	-0.47334879551110	2.60456710797824
C	0.87241175130493	-1.94871530343770	3.05304003508187
C	0.28556629922662	0.44087638149386	3.63289868646274
C	2.45915069652916	-0.06507405517859	2.38521998649390
H	-0.17334127078252	-2.24182977792338	3.14172727721208
H	1.35198753444279	-2.07873031091899	4.02358497988148
H	1.35682262974135	-2.60292881140957	2.32801745412607
H	-0.77116250372001	0.18522274151539	3.70763370603793
H	0.36130833230060	1.48439528229694	3.32583113436689
H	0.73737676949712	0.33347640767138	4.61939350034571
H	2.87741755080274	-0.73706137012891	1.63083908667129
C	3.35686425776695	-0.06020717199965	3.61845775332472
H	2.46097996712084	0.93333201522659	1.94285475719700

H	3.40772800292679	-1.05586778122648	4.06632330030513
H	2.95202913868640	0.60522884135937	4.38427092067334
C	4.78089269303393	0.40177448902300	3.27425807226971
H	5.20497904091297	-0.27081262598405	2.52379851319069
C	5.67924148072443	0.45114948967153	4.48118921222749
H	4.72903630132324	1.39272163000421	2.81420446040681
C	6.48284195393246	-0.63606532660638	4.82304712047829
C	7.27919523007755	-0.60638417310800	5.96210163921270
C	7.28329708350261	0.51671985206731	6.78099894708311
C	5.69441590954896	1.57299682714202	5.31087382599772
C	6.48768754076259	1.60836919614016	6.45087558991600
H	7.90003716969672	-1.45891266963840	6.20763795981525
H	7.90513727924878	0.54344059596623	7.66663677827934
H	5.08066284392650	2.42983136503356	5.05641552085675
H	6.48896580350427	2.49020035553914	7.07934267229626
H	6.48826415898369	-1.51345517102214	4.18630331961431

***Gauche*-i3**

Fe	-0.15283174117812	0.68300842640829	0.94541461585494
N	-0.35889329554737	2.54829011174776	0.62893808048952
N	-0.36546448612758	-0.49806383400573	-0.52073922983277
Si	-1.95031894954280	3.05789369408630	0.14456696831878
C	-3.16277869556056	2.66964401106167	1.53315387181235
C	-2.50469745610797	2.19026949795230	-1.42300820373003
C	-2.05666713640930	4.90478812113770	-0.20209736923652
Si	0.82856278591536	3.60488905942773	1.32489040866828
C	2.36783002762741	2.63412442703696	1.81135566581808
C	0.18036945269195	4.45006205431243	2.87734090737184
C	1.40715527210609	4.91167610956856	0.10244575090472
Si	0.75368041933898	-0.38742663563328	-1.84676620528118
C	2.08079308725772	0.88097882199530	-1.45344499917131
C	-0.06885029348402	0.13735131100697	-3.45182029563362
C	1.61038770162898	-2.03971061099890	-2.12152499648102
Si	-1.51160199989799	-1.78307703433147	-0.30066372407304
C	-2.96853456378313	-1.15807882530826	0.71403597700410
C	-0.75471538621647	-3.24186592218296	0.61154730765921
C	-2.20039050832803	-2.38695008544950	-1.94340880724880
H	1.65428741953259	1.86995147450730	-1.28175998457915
H	2.77072778993145	0.95327448096420	-2.29836808578772
H	2.67712500509914	0.59657851925148	-0.58477850677853
H	2.16467099772887	-2.34447367161698	-1.23252564518943
H	2.32604549654003	-1.96237164374403	-2.94447012349820
H	0.90579128566836	-2.83513619822762	-2.37128118252749
H	-0.39123444350657	-2.93501629911197	1.59330929367371
H	0.09057504424297	-3.66023892158523	0.06220344490646
H	-1.49070506794377	-4.03668997335094	0.75857745676301
H	-2.65525059212549	-0.76593876779839	1.68358636943269
H	-3.67024218597687	-1.97407063986674	0.90713013652484
H	-3.51363557237423	-0.37044868183439	0.19078618079514
H	2.13986707603162	1.77604027279356	2.44375464973054
H	3.04217333571457	3.28754383321149	2.37149377437393

H	2.90766683034968	2.27931927748250	0.93326155340692
H	-0.07858279119976	3.71042416015872	3.63813414389914
H	-0.71346754226641	5.04354918743172	2.67685042220826
H	0.93335438868470	5.11813726655015	3.30391751251550
H	-1.77801125499731	5.52221767104260	0.65281505943026
H	-3.09169189614167	5.14749044882763	-0.45883065026002
H	-1.43118739641873	5.19193688703656	-1.04874356419744
H	-3.17997731225874	1.60363999433184	1.76900312390270
H	-4.18217847748247	2.96574038598293	1.27192094825614
H	-2.87744547862862	3.20322101863512	2.44306133469572
H	-2.39429188039067	1.10884612599455	-1.36297597278872
H	-1.91336766050329	2.53358948900146	-2.27429439055461
H	-3.55298055134549	2.42092848991455	-1.63119614223958
H	-2.68425707955328	-1.57404764553294	-2.48826629657458
H	-2.95004314042994	-3.16231880758640	-1.76364667036652
H	-1.43262002253881	-2.81484891382553	-2.58977781228195
H	-0.50771310576932	1.13137501611381	-3.35917476555672
H	-0.85914548488934	-0.54955658788181	-3.75693644421460
H	0.67264757644258	0.17096916540309	-4.25494218987377
H	1.70281575607345	4.45091574572482	-0.84280422119977
H	2.27945714023053	5.43358514207320	0.50583724789520
H	0.64229522857868	5.65770974163278	-0.11084451679875
N	0.04732990092175	0.02054381156809	2.53882756972558
C	0.10977110057063	-0.47602839265753	3.85922720656144
C	-1.27532610443104	-0.97715054788950	4.31183473071689
C	0.53963613417772	0.70706621978234	4.76122332014262
C	1.13512157290817	-1.64216563244961	3.98867535967986
H	-2.00123427899693	-0.16393006269494	4.28317015111394
H	-1.22726150121640	-1.36670459640841	5.33096643630522
H	-1.62269753436201	-1.77456544496669	3.65484589443484
H	-0.14263809045140	1.54604275567242	4.63011418735674
H	1.54742132524172	1.04238903189758	4.52420427708893
H	0.50995154573801	0.39057256624266	5.80516075838659
H	1.26741588979012	-1.81108493925896	5.06020184469519
C	2.49592166847629	-1.43788748533780	3.31512601090166
H	0.66925264511091	-2.54807466830465	3.59303021220078
H	3.26620240079024	-1.95497829596803	3.89297204532144
H	2.76639689906606	-0.38099892354833	3.33343749497480
C	2.53618196163067	-1.96263008812968	1.87620006253296
H	2.28375959645778	-3.02576345827419	1.88320060978313
C	3.85165088739316	-1.77926552664040	1.16527004209136
H	1.74696431632410	-1.47640482455342	1.29739486449969
C	4.48926653096756	-0.53780227682169	1.12143506676157
C	5.66950546165639	-0.36422798566496	0.41038775827230
C	6.24146036260193	-1.43355187211269	-0.27007278475217
C	4.44281700602953	-2.84483188257865	0.48694573881080
C	5.62404489346603	-2.67735104188643	-0.22672653410194
H	6.14118100737426	0.61021927552313	0.38420145473239
H	7.16019395602355	-1.29839175006924	-0.82633097002485
H	3.96474228683787	-3.81719925282411	0.51166124107078
H	6.06052936721811	-3.51940849538096	-0.74916877406393

H 4.05732312419345 0.30749404379963 1.63990351442503

TS-i3/i4

Fe	0.16023845116257	0.56806142685797	0.80838447180381
N	-0.42734637422677	2.37665880670638	0.61528564803468
N	0.09419535026875	-0.71503411380995	-0.62066447534583
Si	-2.10345580544090	2.69357630117911	0.28455555695971
C	-3.13404916753221	2.39155731694696	1.83121597351507
C	-2.76619772550828	1.57795062265768	-1.07075166723598
C	-2.42027823036413	4.46286409698288	-0.27508653991912
Si	0.71799513319474	3.59494461168577	1.06826554147554
C	2.40382294541515	2.81778895899269	1.45109022924346
C	0.14091454748305	4.54405832597143	2.59007514127965
C	1.03634708118559	4.82427368833671	-0.31890801120360
Si	1.05453945601641	-0.32063800527830	-2.00613081313160
C	2.52094756906149	0.72164759725260	-1.45954341168472
C	0.10201388340972	0.69124938619010	-3.27194539003367
C	1.72459116300622	-1.85933691719654	-2.86065884428556
Si	-0.99689224673209	-2.05125859241978	-0.54139022002833
C	-2.15863534474930	-1.85640925576442	0.92928975231176
C	-0.12672903301463	-3.70773395092886	-0.32348290209986
C	-2.03818842269490	-2.18216387599557	-2.10702902643675
H	2.20117289695504	1.70841376603645	-1.12083535403068
H	3.19949624731657	0.88127015106733	-2.30197506327801
H	3.09529068082018	0.24884896532542	-0.66155917496293
H	2.32720189585867	-2.45536850640197	-2.17466711301082
H	2.36127988888174	-1.56823272490253	-3.70045946870364
H	0.93020031718177	-2.49526692379739	-3.25516429630940
H	0.39515655253530	-3.75898859110600	0.63273376107867
H	0.59971168827440	-3.89342692072266	-1.11584038407222
H	-0.85661121517491	-4.52197092429634	-0.34309042079316
H	-1.60418507823929	-1.88909135271332	1.86808571758439
H	-2.88764156302583	-2.67112931298500	0.93559279948082
H	-2.71914045081536	-0.91938384753333	0.90570929764295
H	2.36995286988068	1.76936877084256	1.75126136741900
H	2.87439838193181	3.36092528832196	2.27494529050745
H	3.06898273273496	2.88747322834668	0.58989266203309
H	-0.03915584058665	3.87358027613362	3.43294141859591
H	-0.78946972809472	5.08167593612937	2.39627398686839
H	0.88894187266520	5.27886715135324	2.89974882926593
H	-2.11680834384323	5.20908830385579	0.46061959154771
H	-3.49342470695662	4.58763622959369	-0.44449981156631
H	-1.90954256191476	4.68401895043688	-1.21378769088024
H	-3.08386093951420	1.34638379750793	2.14418495089839
H	-4.18541723568922	2.63487344902140	1.65591856721158
H	-2.78166781256267	3.00380566110400	2.66440506492448
H	-2.56975650335974	0.52511594249010	-0.87910451274200
H	-2.32098788190047	1.81959214731519	-2.03635033062595
H	-3.84868314590751	1.70664697861440	-1.15656396975824
H	-2.61045915397993	-1.27125804642955	-2.28918518291719
H	-2.74488591198302	-3.01252893571247	-2.02477630341091

H	-1.41924920759607	-2.36517211886529	-2.98785572449030
H	-0.18176225142576	1.65553318674528	-2.84597608867224
H	-0.81011192696274	0.18383463045629	-3.59011434867412
H	0.71134629248241	0.88161473306126	-4.15969482845137
H	1.28642041795130	4.29848620443852	-1.24368324877585
H	1.88511660537563	5.46402788087712	-0.06020380240494
H	0.18151926039787	5.46931088053303	-0.51935987971557
N	0.49359631278486	-0.17279053801202	2.43211911627632
C	0.43867537610396	-0.01630800231905	3.86964206803599
C	-0.99498674023287	0.39581548871280	4.24495660839145
C	1.41239406742865	1.06677582848729	4.36636707860376
C	0.75793129217788	-1.37708384383547	4.54402281998393
H	-1.24614483794402	1.34664888420475	3.77808905460741
H	-1.08447426390500	0.50560225839581	5.32780888831606
H	-1.71051821453338	-0.35359237275397	3.90703343576591
H	1.14317549237272	2.03475737167816	3.94918103009530
H	2.43726183073366	0.85358505658946	4.06866733558397
H	1.37786334168762	1.14201891173230	5.45555865581503
H	0.74599446705372	-1.24659121567389	5.62817751864617
C	2.09691166217128	-1.95379867798472	4.07972829433949
H	-0.04391723419127	-2.07693605421814	4.29198085784833
H	2.26787378310379	-2.91779230957072	4.56989750132304
H	2.91470034223927	-1.30368451680086	4.38945928194715
C	2.09049530966077	-2.13942962799701	2.57584793424146
H	1.54952982500459	-3.03156506611962	2.26528955537750
C	3.27734722699894	-1.88081905194741	1.75020572963485
H	1.26304911479308	-1.22753234468285	2.25114371897963
C	4.17078858061264	-0.83837019648359	2.03652932052905
C	5.24593312945572	-0.56968650866878	1.20375587458115
C	5.45792898653617	-1.33532532509664	0.06133122840542
C	3.49870770349496	-2.63228582077094	0.58707672920214
C	4.57757678189549	-2.36842871876331	-0.24226099841316
H	5.91655924740704	0.24628925110120	1.44106368189469
H	6.29490975941941	-1.12223768547892	-0.59066473984128
H	2.80850524894031	-3.42726469310932	0.33704316149424
H	4.72823506611809	-2.96474910208428	-1.13298404384396
H	4.01629697096024	-0.22227211103887	2.91135598214727

i4

Fe	0.10292487082026	0.47460691129743	0.68790468708904
N	-0.09995901248086	2.37471531277742	0.41164202426641
N	0.02059724896646	-1.08730532769125	-0.44567367140842
Si	-1.69836547230721	2.93795604006865	0.08491408282375
C	-2.47903086652410	3.70708434494250	1.61967578495953
C	-2.80400183713888	1.51758459511680	-0.46456669782637
C	-1.76151044337202	4.22789630875443	-1.28779202637000
Si	1.29262064968400	3.38472106590455	0.34888267014522
C	2.78583290000435	2.58937356999045	1.19856339889496
C	1.02043770096318	5.02381843948675	1.24278078943816
C	1.81234351708266	3.77849351146006	-1.41684769874716
Si	0.96775657081257	-0.87932452191675	-1.88125023878457

C	2.64956669543857	-0.12348725143916	-1.50560156563168
C	0.10802901518423	0.28669507390575	-3.08591941333405
C	1.31075136767773	-2.50486174654876	-2.77376540420061
Si	-1.21390173672926	-2.27787708092278	-0.30742527950521
C	-2.23361669705412	-2.07341522478283	1.26627999644525
C	-0.53924550010531	-4.03945036583105	-0.19188926605043
C	-2.42046767740787	-2.23129901897763	-1.75429985976214
H	2.56477138584164	0.90984332042845	-1.17315651775587
H	3.25916558359791	-0.11764691426820	-2.41400668115107
H	3.19177826777648	-0.68181296019817	-0.74283040151783
H	1.88860436449224	-3.18658288587543	-2.14597210129936
H	1.89844040430294	-2.30489950254512	-3.67386427333153
H	0.40109934388626	-3.02178959815854	-3.08371725200305
H	0.00951161994091	-4.19771285250746	0.73981646473114
H	0.12428755947501	-4.29180668475342	-1.01857538341079
H	-1.36772302203253	-4.75363327249218	-0.20034620069890
H	-1.64273244991259	-2.28449794005263	2.15944593350912
H	-3.05750577662248	-2.79223051723348	1.25134902627919
H	-2.66805129885829	-1.07984920038561	1.37554886872947
H	2.67907079734564	2.59239234901174	2.28537392649419
H	3.67839478919161	3.17798485730763	0.96972253284771
H	2.98935186491474	1.56970212550512	0.86864983525327
H	0.76020335678970	4.86732984154241	2.29207525794215
H	0.22124153614787	5.61156767611909	0.78742061542037
H	1.93045977364549	5.62958872122940	1.21235423537025
H	-1.18400241088199	5.12277191279545	-1.04897885521697
H	-2.79576740779874	4.54038940203133	-1.45621304610043
H	-1.37919015794534	3.82316528086542	-2.22732940682213
H	-2.55026466272628	2.98269897293755	2.43482888524802
H	-3.48962652679624	4.06707914327763	1.40803651288783
H	-1.89194503992462	4.55455528618924	1.97982706743902
H	-2.96988774889196	0.78433496151012	0.32676098855346
H	-2.37547764095538	0.99333235937171	-1.32006731977952
H	-3.78399786516548	1.90123436925441	-0.76143783615278
H	-2.93378290367134	-1.26909354803004	-1.80320513314068
H	-3.17951138760780	-3.01177684713531	-1.65328093887067
H	-1.91488903426911	-2.38471549815793	-2.70996082831182
H	-0.04812541077797	1.26129329072550	-2.61728941520352
H	-0.86839298751652	-0.09702862733240	-3.38896762668040
H	0.70449772685304	0.44176760287762	-3.98911961273170
H	1.97133281772042	2.87011913972282	-2.00008255360697
H	2.74238303308648	4.35348830224273	-1.43163020776745
H	1.04855395496155	4.36653936096117	-1.92795796900140
N	0.91964851711029	0.11776871312391	2.67004525661302
C	0.21929580419038	0.60912922083532	3.90811683000051
C	-1.25118593174007	0.20373991794708	3.86597382001844
C	0.34123534526016	2.12240542859695	4.00731950853399
C	0.97301909302537	-0.13327359949809	5.02490386659268
H	-1.74940689074536	0.65072486387830	3.00517198581993
H	-1.75475278189123	0.55283244070230	4.76822845591567
H	-1.37815995281898	-0.87533072005586	3.80524189408022

H	-0.09703430318735	2.60416652246811	3.13461563840245
H	1.38570993420708	2.42929920708128	4.08509936160904
H	-0.17804500826902	2.47710742664806	4.89831431233115
H	0.39737087737746	-0.17840464250646	5.94960209747565
C	1.27983329287891	-1.51669235664283	4.44222271287461
H	1.90126988010707	0.40135815460697	5.24290665239279
H	0.49944146754721	-2.23395035370833	4.69628350411158
H	2.22052938948589	-1.92023979896760	4.81166175277414
C	1.32029383974486	-1.31115882341341	2.90052819783317
C	2.64584375457048	-1.60827708008561	2.24786895018892
C	3.79639318679422	-0.90164817512815	2.60220886147663
C	5.01246165594525	-1.17539200668803	1.99133694878887
C	5.09810849151116	-2.17306258538264	1.02562117668637
C	2.74144062616577	-2.59830618155328	1.27494138963314
C	3.96011534613995	-2.88655475013989	0.67249771711223
H	5.89341176226998	-0.61115922466631	2.26877847937167
H	6.04526096081924	-2.38521381410322	0.54696166681893
H	1.85058596823717	-3.12490152762079	0.96643568150294
H	4.01313787477164	-3.65394209997285	-0.08894512978957
H	3.75033507720132	-0.12855956716810	3.36118784119226
H	0.56910543176567	-1.93862021319186	2.42831742928880
H	1.77883155039559	0.65755956222982	2.59555023775749

***Gauche*-i2**

Fe	-0.37617634603660	0.72666088398143	0.92876094110329
N	-0.72086865071775	2.61289234421402	0.81660279489353
N	-0.09005360109431	-0.73190957187574	-0.28043724252073
Si	-2.23933618383295	3.09727649014578	0.14298353295872
C	-3.42956043071488	3.70596330697888	1.47053638400912
C	-3.08078317862433	1.65370776989764	-0.72564269489056
C	-2.05069178577952	4.46834493021352	-1.13530634266508
Si	0.52875476765072	3.72774887316752	1.23274398942792
C	1.82986964266743	2.89728888701787	2.32282764122143
C	-0.13460811241175	5.19233824949922	2.21553358790839
C	1.42461599090092	4.41130846753427	-0.27468292839720
Si	0.98041310621493	-0.34391628265855	-1.59185550092931
C	2.48104507673291	0.60833890821901	-0.98584502290679
C	0.13080166810179	0.75875347606122	-2.86062941250061
C	1.61679950899803	-1.88337506330639	-2.47161291279213
Si	-1.24156691054064	-2.01342028588138	-0.36479636801351
C	-2.51693839998537	-1.88343119903854	1.02223820181914
C	-0.44304124901655	-3.70949743047574	-0.15841716994631
C	-2.19620065086386	-2.02526766312486	-1.98838489098780
H	2.19400012668365	1.50964888043060	-0.44389488815467
H	3.08511905776587	0.92552272627886	-1.84040130770942
H	3.12068291231673	0.01196156690320	-0.33732776484709
H	2.17310144301255	-2.52852750128869	-1.78941968445578
H	2.29964767883911	-1.58754139070333	-3.27251343167445
H	0.81731234513227	-2.47443496096354	-2.92139914679276
H	0.06604586722976	-3.79856236611019	0.80355038661761
H	0.28827921562222	-3.91421938766745	-0.94141282067892

H	-1.20487485204044	-4.49305149208886	-0.20165336138716
H	-2.07746564725095	-2.09993403212088	1.99601329060597
H	-3.30769655473629	-2.62006606794167	0.85558541449070
H	-2.99064096775281	-0.90252604922065	1.07883005941758
H	1.41363435751738	2.56545860370077	3.27566040953070
H	2.62199108650813	3.61669470080991	2.54710773961246
H	2.30224473380304	2.04004696583151	1.83916033121253
H	-0.63349799665446	4.86312653670729	3.12962342297702
H	-0.85328749194424	5.77737484765703	1.63783745932647
H	0.68063251255991	5.86332277480766	2.49975535487848
H	-1.64618102957770	5.38648320663576	-0.70501967759743
H	-3.02524058916600	4.71076139130018	-1.56765606485900
H	-1.39085317830122	4.16079630208686	-1.94936086503246
H	-3.63982246795712	2.92416963576859	2.20447427092576
H	-4.38230176368256	4.00499880576387	1.02444893535057
H	-3.02914721784728	4.56507979167452	2.01117786277889
H	-3.40501116117954	0.88219232019296	-0.02513900558548
H	-2.42856435072198	1.18689667479318	-1.46373846055683
H	-3.97317350374494	2.01146330809738	-1.24658160019294
H	-2.76079206412231	-1.10003838258241	-2.11697433252426
H	-2.90669202162410	-2.85628429934337	-2.00516254196173
H	-1.53891234692674	-2.13748177702295	-2.85255187495919
H	-0.16585895898822	1.70424583081153	-2.40012363745133
H	-0.76453340245766	0.29490977176110	-3.27838506972270
H	0.80549862515448	0.99194999520834	-3.68882616724132
H	1.81693050094609	3.61251121742691	-0.90598229664850
H	2.26501209325075	5.04138778601025	0.02974484269463
H	0.75879423794392	5.01910430378479	-0.88879912205252
N	-0.54422297133553	0.44849501404542	3.12617272075963
C	-0.03162921220714	-0.60585158029055	4.10731424955857
C	-1.23714104911361	-1.26398350022777	4.77718406675452
C	0.86021321091209	0.06909235169824	5.14817014836542
C	0.73300730192336	-1.62745730183461	3.26266359909233
H	-1.83993501498488	-0.53226823630915	5.31857554919207
H	-0.89439988224057	-2.00815831188733	5.49721125272697
H	-1.87242795464595	-1.75951441921686	4.04491634668092
H	0.28563075458382	0.74009458206295	5.78909293700747
H	1.65896482151955	0.64002289568460	4.67908537306053
H	1.31044134851939	-0.69135477755356	5.78772199097810
H	0.88816796538082	-2.50912720756421	3.89044650705545
C	2.07711158172460	-1.17354905811025	2.70279345149063
H	0.08679206743238	-1.94596942958556	2.44417477943294
H	2.78122937505110	-1.01635580679319	3.52220938734852
H	1.97297113187434	-0.21041949026012	2.19571668854023
C	2.63211810036492	-2.19737507460480	1.72192635080470
H	2.64402123710414	-3.18370804893692	2.20018075749596
C	4.00694520829261	-1.91421645291518	1.16396016588119
H	1.93172650537148	-2.27714199950933	0.88736366569007
C	4.79780874819238	-0.84800250726098	1.58629842314432
C	6.04261186987158	-0.60839930752977	1.01057353867012
C	6.51905658155715	-1.43376006023075	0.00228233245702

C	4.50292479136376	-2.74045385848839	0.15209847027102
C	5.74158980118537	-2.50623432872601	-0.42514972673090
H	6.63473271943620	0.23258726220198	1.34913634773295
H	7.48363120839067	-1.24363718206640	-0.45052480950943
H	3.89972013655058	-3.57215688861787	-0.19359390327658
H	6.09929909576812	-3.15599747267982	-1.21397069402414
H	4.44194842811897	-0.17726945877319	2.35636907043197
N	-1.12010791227948	1.39834850426463	3.67311258861023
N	-1.64610348294018	2.28778582205608	4.11117512918146

***Gauche*-TS-i2/i3**

Fe	-0.29023619292583	0.80842485131009	1.14066266186246
N	-0.78575327572062	2.64499452443478	0.89111885584909
N	-0.10872165302395	-0.55277907412025	-0.18516826541242
Si	-2.40944745507455	2.95840148083392	0.34350339365541
C	-3.66234692915737	2.32706241871701	1.59843194093991
C	-2.71470314512596	2.07570648922456	-1.28712971564011
C	-2.76610558217082	4.78306550568562	0.07084780620510
Si	0.50522870480371	3.80229522846510	0.99753412124199
C	1.91968100701560	3.02813723108069	1.98229060581717
C	0.07306837163045	5.40573603312726	1.87708792586750
C	1.11149579471343	4.26597690147098	-0.72323103103069
Si	1.06215942624051	-0.31492158258515	-1.45502835827661
C	2.56854644373667	0.60923552359751	-0.82094760722485
C	0.36121384456787	0.70334543713758	-2.86962697340226
C	1.68204703825385	-1.95136356558178	-2.15013099682448
Si	-1.33837649514548	-1.77447347556346	-0.30875778431478
C	-2.76670677317946	-1.35363372645289	0.84393141973030
C	-0.70582280724925	-3.49061394080388	0.13916454626118
C	-2.07267962738061	-1.89387523988737	-2.03897773432991
H	2.33357670534370	1.61544765249589	-0.47748732727242
H	3.27963674510245	0.71797819501847	-1.64458325072112
H	3.08780063610391	0.08364247004743	-0.02095217333701
H	2.16753218421783	-2.55442185425706	-1.38270500331197
H	2.42635796741376	-1.74958487836883	-2.92503252300644
H	0.89051121480955	-2.54951745341228	-2.60293708292082
H	-0.41190096862185	-3.56862077172953	1.18651983927444
H	0.15569963463872	-3.76988015415206	-0.46923838180636
H	-1.49126078601858	-4.23059088167031	-0.03850640282681
H	-2.44682012506231	-1.12830190111594	1.86205971533363
H	-3.45196292905368	-2.20366986080444	0.90016409961381
H	-3.33943634728903	-0.50041285426344	0.47675413217617
H	1.65283994256385	2.95311211460744	3.03773760696181
H	2.80406475399262	3.66648249232430	1.90461552707484
H	2.22712294281521	2.03563294906372	1.64759325126004
H	-0.45281597890095	5.22118421318903	2.81365984349612
H	-0.54099635512466	6.06283046684185	1.26162381813349
H	0.99842554404175	5.94196308440653	2.10722058179738
H	-2.72455723052698	5.34963877830970	1.00135779825635
H	-3.77608846475117	4.88426104986365	-0.33625277797221
H	-2.07812438000485	5.24248849156690	-0.64142328495976

H	-3.44720798412299	1.31171235213717	1.93552686585695
H	-4.66472811420653	2.32418302106469	1.16168481945626
H	-3.68242495835638	2.96769355711216	2.48136718882366
H	-2.47986619533952	1.01404075712939	-1.22116629661261
H	-2.09334097802065	2.49395178057745	-2.08110418977071
H	-3.76046802888558	2.17240670777063	-1.59048290611993
H	-2.50874024130458	-0.94595513181114	-2.35776335959299
H	-2.86890753663750	-2.64365041928678	-2.04112700105953
H	-1.33973690762104	-2.19384652329102	-2.78887057348281
H	0.06476284971901	1.69302561296526	-2.51825633822383
H	-0.51479451404942	0.23560566186362	-3.32033548018063
H	1.11256650976194	0.83787928569506	-3.65249386583559
H	1.44961865315150	3.40284314276155	-1.29715639106906
H	1.93864429201890	4.97898117024626	-0.66842633503402
H	0.30566522653940	4.73959541618288	-1.28954484083618
N	-0.32977224054850	0.58437753474571	3.01308791059944
C	0.02221609538271	-0.47450796145167	3.97502789876271
C	-1.24034765561226	-1.09801874824768	4.58723492675668
C	0.91505052639689	0.08524929353416	5.09174640607027
C	0.76678370129945	-1.56721986345722	3.18576129947526
H	-1.84709098966453	-0.33503866326973	5.07470686607931
H	-0.96733516655668	-1.84775041288677	5.33242007751845
H	-1.84613460082554	-1.58342428983851	3.82220888146204
H	0.34816685171278	0.75433166137038	5.73920302172407
H	1.75427702854907	0.64270759808390	4.67778932552607
H	1.30684234523465	-0.72800141431771	5.70605170793918
H	0.83614997321963	-2.44298981269414	3.83772062415861
C	2.16347376932749	-1.21954038032621	2.68157889572551
H	0.14443038461495	-1.86393540076956	2.34246064969530
H	2.83736933389272	-1.08346459209544	3.52946418016275
H	2.14868566545184	-0.26545512885144	2.14847210501519
C	2.69403403817282	-2.30811612251322	1.75839522775610
H	2.66611810100182	-3.26776566841101	2.28758909757325
C	4.08358583886521	-2.10252894099406	1.20203570445335
H	1.99828206411171	-2.41608465712249	0.92271811026141
C	4.92489725128811	-1.07362295716305	1.62013491301381
C	6.18976499573104	-0.91107257939882	1.06169159752043
C	6.63741063373463	-1.77826180190930	0.07532717564628
C	4.55026616858122	-2.97014932209845	0.21108378812909
C	5.80931935038183	-2.81339762881172	-0.34858588782630
H	6.82268833939150	-0.09999390798668	1.39952051583352
H	7.61924516728091	-1.65026506912619	-0.36200194126371
H	3.91147426053593	-3.77733570659802	-0.12889080429832
H	6.14455662719258	-3.49659524363999	-1.11890380554013
H	4.59581466691167	-0.37776566773898	2.37979816567923
N	-0.99238693406032	1.73368735845577	3.86410227007021
N	-1.24747306414005	2.80440973635908	3.60338698378375

i17

Fe	-5.38812725253540	1.82817247508399	0.45382814055333

N	-3.59098908383105	2.01930310169700	0.95013662793376
C	-2.82930364156872	2.96480139591612	0.14142820709948
N	-7.22970591995452	1.58497228981918	0.08032310073422
Si	-8.24461582130343	2.92581032993934	0.44967022637322
C	-7.26994915926029	4.12198109374632	1.53824313015440
C	-9.79657547613592	2.40023648495910	1.37503526490468
C	-8.76169074905368	3.85610639286228	-1.10073725492044
Si	-7.68060689923110	0.07238543482208	-0.60111509160721
C	-6.13717071573746	-0.76730184902678	-1.29513593558059
C	-8.91408997933821	0.26496408719155	-2.00949166589612
C	-8.42331056014330	-1.07497540761339	0.69204936618674
H	-7.87985826094199	4.19329960075842	-1.64919363982981
H	-9.36764564563026	4.73309608418868	-0.85687547629630
H	-9.34571726409977	3.22000798248895	-1.76867353776374
H	-6.98978890610462	3.66956187739862	2.49294506516135
H	-7.86519053224042	5.01225539113151	1.75928161418779
H	-6.35540794321542	4.46677613579945	1.04494382830578
H	-9.54464658147842	1.86761430564871	2.29488385136530
H	-10.41519232557066	1.73660481076774	0.76579706531044
H	-10.40825627718924	3.26609897037876	1.64214828411865
H	-5.72783938533594	-0.20726647523547	-2.13868597973279
H	-6.37517468742582	-1.77330645769942	-1.65103690098990
H	-5.34622669588236	-0.87554343201313	-0.54587842372839
H	-8.51599945871924	0.91572168537111	-2.79127306039495
H	-9.85509703636203	0.69872492348600	-1.66316297098959
H	-9.14531465941992	-0.70377832176754	-2.46043344050669
H	-7.71468772641914	-1.24526518284919	1.50668564455901
H	-8.68520671641459	-2.04715754929534	0.26556023146055
H	-9.32804678511352	-0.64542191614676	1.12763033388195
C	-3.66857045868987	3.38447874741842	-1.04505088694731
C	-1.51906147746062	2.21848815365575	-0.13919552087909
H	-2.58519643939523	3.87801956249533	0.70947696461469
H	-1.67583505202533	1.47996885229047	-0.92796725747509
C	-1.25944014782354	1.53541220992609	1.20643129250577
H	-0.70921907752266	2.87988070953891	-0.45172171390114
C	-2.67454618539464	1.25649207153616	1.80468729707347
H	-0.70506538449143	2.20819817661456	1.86434654940326
H	-0.67194836095381	0.62169865599030	1.11046966618917
C	-2.75668033429857	1.74235688522935	3.25801045261860
C	-3.02800299685850	-0.23217296448984	1.73959619638821
H	-2.57797269056089	2.81785589158444	3.30888219823388
H	-2.01638620183610	1.24068438275445	3.88749494346377
H	-3.74827380835061	1.54470163436059	3.67069955274490
H	-2.91998903070684	-0.60731193724043	0.72030442260716
H	-4.06350253247559	-0.39285035839841	2.05340262153694
H	-2.38648601577806	-0.82271104970633	2.39773575232265
C	-4.12457801339488	4.69667847178642	-1.17454042925600
C	-4.97987416575609	5.05667771973650	-2.20883372858411
C	-5.39918326177494	4.10501527767602	-3.13193314543271
C	-4.95252637774420	2.79456380682194	-3.01903877020570
C	-4.09367946835559	2.43646626492510	-1.98511678520976

H	-3.81325358552180	5.43596868914004	-0.44633828754773
H	-5.33079591936473	6.07779452220229	-2.28640973601108
H	-6.07508111272787	4.38228797103392	-3.93027095148156
H	-5.27418366024662	2.04789661470951	-3.73360522497930
H	-3.73764609483346	1.41679277660019	-1.91242207584598

i18

Fe	-5.48633474758505	0.55574475438816	-0.11690188997763
N	-3.96190134235534	1.64475417888246	0.27523348305757
C	-3.74714253928364	2.89656615379736	-0.44918817139202
N	-7.27177919868074	0.68501619125958	0.64321472610827
Si	-7.91031516136649	2.28558475076732	0.65883081153699
C	-7.07370831674755	3.35733181392434	1.96361426228709
C	-9.76873806761564	2.33652874755761	0.98509241317630
C	-7.65805614790604	3.15825834250946	-0.99623183731553
Si	-7.87703418643795	-0.58705425717725	1.61987663479430
C	-6.71811179435806	-2.08078468432970	1.60397060165612
C	-9.53659630536503	-1.24000757294763	1.00364626893184
C	-8.06327317245995	-0.08195757357969	3.42747915026010
H	-6.62665340404006	3.09257463514076	-1.34827434900381
H	-7.90580828469487	4.21910706824222	-0.90059055210175
H	-8.30354396022428	2.73708288627387	-1.76965746657251
H	-7.16706032972005	2.91581598616890	2.95813960343276
H	-7.51082947489932	4.35908664884712	1.99697618540185
H	-6.00914940017901	3.46169351233196	1.74544722195590
H	-10.03989201961632	1.90657899161352	1.95088399765071
H	-10.32191299455878	1.79825780572209	0.21231592237937
H	-10.11358053650305	3.37427671576779	0.97971329146672
H	-6.73446945811489	-2.58549027344042	0.63534062929369
H	-7.04538244909799	-2.81069276893780	2.34956031264142
H	-5.68606364048025	-1.81037588310576	1.83732937491467
H	-9.45945905990853	-1.55652849983307	-0.04028789203250
H	-10.32071109478649	-0.48393803728271	1.06052102487954
H	-9.86156339046744	-2.10386077245075	1.59053943043351
H	-7.09396537657059	0.19887654011016	3.84690548561854
H	-8.46406773124863	-0.90281863870576	4.02845396056022
H	-8.73286651379360	0.77217982938427	3.54668509714904
C	-3.38278548806098	2.70351311044841	-1.90644886199627
C	-2.65563088350725	3.65422527171243	0.34156515195716
H	-4.65376788471915	3.51920381805287	-0.43216242459941
H	-1.66741215958601	3.39610846832402	-0.04358443224895
C	-2.84268955770902	3.11872767540492	1.75675858684924
H	-2.76961537955785	4.73545004059678	0.25830260081352
C	-3.18209341496292	1.63353510401026	1.52566688693332
H	-3.68778667701199	3.61514067757770	2.24016615407672
H	-1.96444340626505	3.25573170107499	2.39057154557937
C	-3.99325797553758	1.04152909087360	2.67951788956188
C	-1.89174592277998	0.81077631800403	1.36126255834941
H	-1.23365645213885	1.24988680866564	0.61082828862196
H	-2.13506181501369	-0.20041247925599	1.04130783861551
H	-1.33636773704988	0.75035009832547	2.30170345943444

C	-3.95132538761156	3.49882826076705	-2.89904823307659
C	-3.57134800229881	3.36479792786734	-4.23192805868324
C	-2.61489500472364	2.42466942535674	-4.59338110192672
C	-2.05148943144788	1.61312458467897	-3.61201892736870
C	-2.43658811194479	1.74802889215831	-2.28513370269298
H	-4.69973627458336	4.23274352061208	-2.62349240600571
H	-4.02712002218009	3.99280196819360	-4.98737405109156
H	-2.31774038760533	2.31708293188912	-5.62874330591381
H	-1.30619079984095	0.87510114325947	-3.88464994309762
H	-2.00721482615166	1.11154217314506	-1.52388774218646
N	-5.02212988581875	-0.65619624358093	-1.88318579264288
C	-4.61205121183216	-2.05794951004496	-1.52913638786540
C	-4.88976766072015	-2.87597905088217	-2.79401282012167
H	-5.29150681881661	-2.40450209880642	-0.75222552762381
C	-3.20849293652343	-2.15984158384281	-0.99813085943751
C	-6.16565049427183	-2.23231144647108	-3.31418574150209
H	-4.99244441849030	-3.93764510899989	-2.57002121292297
H	-4.06894218191651	-2.76543024527270	-3.50573331934800
C	-5.96573342433799	-0.72124106797845	-3.07827969955915
H	-7.02255189474809	-2.58478955618289	-2.73465431826040
H	-6.36726722085896	-2.44451844281842	-4.36410104321551
C	-7.28451802102538	-0.02450449294537	-2.77945635802114
C	-5.27952866165558	-0.05477726597881	-4.26911642721795
H	-4.36558942218469	-0.57938599567775	-4.55342468112801
H	-5.01592568198228	0.97664767200158	-4.03454462341187
H	-5.94777398436614	-0.04416655346352	-5.13145283382538
H	-7.72778444363767	-0.38829486308260	-1.85330359987729
H	-7.98604905509112	-0.20179936111772	-3.59773311734712
H	-7.14582825162844	1.05079154257903	-2.68101623259264
C	-2.97415934271461	-2.75552069348225	0.23873332482994
C	-1.67857074194675	-2.91152317967873	0.71850819843883
C	-0.60032761099450	-2.45972485664082	-0.03069675075621
C	-0.82422936762821	-1.85768594250593	-1.26460549663377
H	-4.93499478418681	1.57375654659675	2.80514553879201
H	-3.43292761781788	1.09219886617039	3.61612836013820
H	-4.22089601245996	-0.01070734047695	2.48980583323619
C	-2.11753834343949	-1.71808587271791	-1.74675077708833
H	-3.81370883368507	-3.09745533867019	0.83099498033095
H	-1.51480922749259	-3.37407903408679	1.68321681034824
H	0.40841713635970	-2.56852401968825	0.34573544190649
H	0.01125125589117	-1.49987631532326	-1.85279790166888
H	-2.27552228516949	-1.25657015876295	-2.71346734782902
H	-4.18947545345452	-0.15154211080864	-2.17770111921907

i19

Fe	-4.24144194191655	-0.11282018803266	-0.72620985373077
N	-3.92617888443632	1.55818809255677	0.06316735906780
C	-3.62047731712411	2.79071087600818	-0.66736587405487
C	-2.35709496054256	2.73156352042115	-1.49872144517932
C	-3.55691239023252	3.89230715417819	0.41317000808317
H	-4.44196300447143	3.04393378578829	-1.35484598994053

H	-2.54227133936522	3.96120291737570	0.80943003750043
C	-4.51956844979293	3.37518641592917	1.47395209386745
H	-3.82176901489970	4.87163953640412	0.01383373820153
C	-4.27105543208712	1.85396231232518	1.46733720520483
H	-5.55168427459850	3.57548405750484	1.17360452972050
H	-4.36431134480601	3.82106513517230	2.45787413225297
C	-5.51740343930177	1.07489522949947	1.89571943506336
C	-3.10654615263595	1.48784804859201	2.40408483937545
H	-2.21518723415064	2.07075323711463	2.16943375741747
H	-2.85333244591247	0.43187722708007	2.28821464172331
H	-3.36577071891188	1.66653753725198	3.45117833527220
C	-2.33428937966257	3.26067116838204	-2.78699805139337
C	-1.15568523443915	3.29551393306084	-3.52661208094565
C	0.02064929038294	2.79619199406932	-2.98219171712462
C	0.00433625417643	2.24872629954440	-1.70241624247114
C	-1.17448964275149	2.21088220940387	-0.97268177001083
H	-3.24958087478716	3.65728748467190	-3.21200482707907
H	-1.15793229277268	3.71245318564302	-4.52620871682758
H	0.94028152042734	2.82188283667760	-3.55312905655627
H	0.91295026978002	1.84169033917973	-1.27664602696955
H	-1.19095467916631	1.76514894808247	0.01300596499181
N	-4.97446002133478	-1.73147895060997	-1.32784957189948
C	-4.10979986403492	-2.75024467388414	-1.90924593507683
C	-4.93305615906077	-3.29770548843530	-3.08459487343277
H	-3.93268972122004	-3.58266798137852	-1.20726324133692
C	-2.76283569662928	-2.16217400249738	-2.25789608422614
C	-6.33798081361633	-3.32195722553152	-2.48596902717300
H	-4.58756984563225	-4.27439118881481	-3.42748271531559
H	-4.87515917886023	-2.60402059715317	-3.92619274378486
C	-6.38623112099950	-2.08345424856912	-1.54687844346165
H	-6.47293195489881	-4.23451380194628	-1.90014286775895
H	-7.12882892271281	-3.29646783489072	-3.23669859675657
C	-7.08038163732125	-2.42335425922028	-0.22235563163727
C	-7.11340265189431	-0.91105050628562	-2.21885048126962
H	-6.67722149913296	-0.70082726689016	-3.19698906178398
H	-7.02542183178469	-0.00749809450153	-1.60777520632163
H	-8.17785611939131	-1.12147105648215	-2.34977529872379
H	-6.53238104481940	-3.20929409912625	0.30004107381923
H	-8.10537504602367	-2.76754113130855	-0.38723111899493
H	-7.11714331934442	-1.54645273175675	0.42807880472394
C	-1.58635424407773	-2.69866510866265	-1.74070068456014
C	-0.35144685401186	-2.13159627014490	-2.04036060653168
C	-0.27969255852570	-1.00847018367959	-2.85387012026059
C	-1.44600983408769	-0.45650034327671	-3.37153293686988
H	-6.35736839233465	1.30970869483405	1.23954414988581
H	-5.80261840684488	1.31637524616638	2.92254880988720
H	-5.33902258405745	-0.00469750188556	1.85485088542408
C	-2.67526350823727	-1.03099769098787	-3.07917670968979
H	-1.64242702664449	-3.56641953990660	-1.09403605062754
H	0.55261004443557	-2.56149721757707	-1.62736249825521
H	0.67677017943925	-0.55356183503249	-3.07437202190954

H	-1.39771611739310	0.43169349978053	-3.98622957888267
H	-3.58030113494875	-0.60692990422998	-3.49690604265760

i20

Fe	-4.32238768365130	0.40303286192344	-1.22219220742845
N	-4.05459761754692	1.58966801407202	0.26746956748803
C	-4.92158922207986	2.74237819792197	0.49338572700107
C	-4.75065926857491	3.88644328052835	-0.48715414767969
C	-4.62852793383590	3.21351770211539	1.94025077716105
H	-5.98258513712120	2.44968333082463	0.44581615135509
H	-3.84344297419154	3.97198173751294	1.92908811980309
C	-4.13993148695448	1.93964354015725	2.61433061320454
H	-5.50635092382759	3.65714840758901	2.41109575533271
C	-3.33455014095884	1.24008546108473	1.50224914514928
H	-4.98973266680638	1.31084280861038	2.89431293322561
H	-3.54812215537441	2.12408861785442	3.51315109270530
C	-3.28945500393688	-0.27351475810444	1.71152070222779
C	-1.88806052324690	1.77235796166928	1.46819472152365
H	-1.86839573077143	2.85813400912993	1.36569639968914
H	-1.35775075242254	1.34595673680786	0.61453685921730
H	-1.33988733614261	1.50599379270211	2.37652147157058
C	-5.82947488475735	4.71768528125646	-0.78942521111850
C	-5.66957451936739	5.83402815330735	-1.60300899461413
C	-4.42487279538179	6.12733615287134	-2.14747905666255
C	-3.34608153267941	5.29464504484351	-1.87073787430884
C	-3.50853828849589	4.18725757571301	-1.04644477601034
H	-6.80418639099923	4.48928066082770	-0.37263774129951
H	-6.52001826612237	6.46894144734131	-1.81956444426583
H	-4.29814413103088	6.98806882998740	-2.79125257033961
H	-2.37491090263191	5.51066515370334	-2.29882872059077
H	-2.67349476021326	3.53674804561216	-0.82171730252230
N	-5.17039176030596	-1.32109261960659	-1.42066966599532
C	-4.50813705886343	-2.55884744825468	-0.99560158089006
C	-5.59593590868047	-3.64473474908911	-1.05486356194054
H	-4.17826319851179	-2.49275150670754	0.05312678234498
C	-3.27236390688247	-2.87042479273770	-1.80959809787305
C	-6.84398915458763	-2.85377483342213	-0.68552232867015
H	-5.38403345268449	-4.47616426817186	-0.38130019140111
H	-5.67754896377003	-4.04933429174077	-2.06540661354988
C	-6.63538989140679	-1.50603584255230	-1.40763557249060
H	-6.87272356370227	-2.68924576763565	0.39522977561914
H	-7.77610732351426	-3.34322783469682	-0.97346372612215
C	-7.33937756153530	-0.36241791715797	-0.66906775201869
C	-7.19547750246972	-1.58905299499955	-2.83976723304132
H	-6.74030795889318	-2.41494737526561	-3.38772487575430
H	-6.98109082430255	-0.67072556202404	-3.38720613589088
H	-8.27951606268733	-1.73410536919253	-2.83730055309745
H	-6.94501838136838	-0.26315772380237	0.34339939909985
H	-8.41789156575373	-0.53212033507930	-0.61061232909427
H	-7.18435843393570	0.59011405539433	-1.18520821878866
C	-2.01994807867055	-2.93592118332952	-1.20382102692601

C	-0.87101652069395	-3.18841789977148	-1.94904855655123
C	-0.96195461200475	-3.38533068248019	-3.31976730205243
C	-2.21042844082073	-3.33320316472798	-3.93695319260888
H	-4.29696785297765	-0.69161051345930	1.70118821073208
H	-2.81455083934530	-0.52441073757595	2.66342411744866
H	-2.71491955950423	-0.75605701260012	0.91755203042097
C	-3.34920674964463	-3.07657188766041	-3.18810162093966
H	-1.94395255559574	-2.77758052656074	-0.13459461945848
H	0.09307512065995	-3.22613682066967	-1.45722375901030
H	-0.07204346435052	-3.58058581163314	-3.90460408727772
H	-2.29273393741177	-3.49409938098480	-5.00507154308931
H	-4.31375510029254	-3.02607722375488	-3.67571280202753
N	-3.85252743941715	1.49960879865103	-3.07582135716493
C	-2.49731994325790	1.20387880808035	-3.68015754499311
C	-1.82210201725331	2.49829231073686	-4.13871822396445
C	-2.82655777275026	0.27159152044948	-4.87543074237121
C	-1.62750167620730	0.50695609173048	-2.63783306264354
H	-2.56561003763043	0.76589787967494	-5.81160927708464
C	-4.33401145393843	0.00944849861554	-4.79163162651745
H	-2.25636265462677	-0.65404601218408	-4.81987563836188
C	-4.88733286631563	1.26199049151452	-4.12567519979018
H	-4.52997557530373	-0.82833345053394	-4.12560808733414
H	-4.79559025713835	-0.20972843499164	-5.75246576643038
H	-5.82881340174792	1.05140665062281	-3.61591768682195
C	-5.11592949476167	2.46818066926203	-5.02094979519355
H	-2.44630064633064	3.04347890167342	-4.84583764068366
H	-0.87138403852214	2.27016773815986	-4.62444391561035
H	-1.61594820625020	3.15008536096637	-3.28764558675103
H	-1.49972015347180	1.13533483366306	-1.75452414062148
H	-0.63940998597060	0.29156375393165	-3.04776917145243
H	-2.06524732581988	-0.44479876148106	-2.33297671691590
C	-4.90420232414946	2.45289597154732	-6.39763854329458
C	-5.13222869436729	3.58969472061504	-7.16654260736500
C	-5.57808592473015	4.76102026215655	-6.56868729760558
C	-5.79869553049565	4.78586475998877	-5.19573630183346
C	-5.57221588179174	3.64945617844220	-4.43361179999935
H	-6.14723595566290	5.68986511577314	-4.71434211694214
H	-5.75201627905250	3.68688316041495	-3.36621093375673
H	-4.96000549252780	3.55651364272016	-8.23503058548293
H	-5.75496483605176	5.64586662154144	-7.16664838895021
H	-4.55673027338925	1.55307350814221	-6.88525764648839
H	-3.87929576743508	2.46578238420381	-2.76033295649415

i4 + 1a

Fe	-2.08635982157803	0.24454088504030	-1.30935080185198
N	-2.43242918815609	1.88698269593590	-0.34667275678858
N	-0.88486628582725	-1.27117742003069	-1.10571723395432
Si	-3.93347572632531	2.29421765048924	0.40843425930126
C	-5.35085436171853	1.12921005825228	-0.03523324821127
C	-4.47784802184352	4.02753989656413	-0.10408145551365
C	-3.90786294447828	2.22086547298808	2.28818156552984

Si	-1.03617051441359	2.89607720292252	-0.14416100493660
C	0.55120295278108	1.91305593308590	-0.39523274207384
C	-0.91525596140151	3.69154242990130	1.55880037441354
C	-0.95874647639231	4.32291295787228	-1.38151193464916
Si	0.45008714236308	-1.59873739227076	-2.13848986392656
C	2.13436954319413	-1.39191993505085	-1.32039150489732
C	0.54709320785987	-0.45729358753557	-3.65499672656937
C	0.37698226377907	-3.35139818917187	-2.83436197870284
Si	-1.24900261421363	-2.25443724669725	0.27263455350188
C	-2.04282356453776	-3.89436826502345	-0.18927408592895
C	-2.44657604404058	-1.36208170781273	1.42037039916226
C	0.27987285152511	-2.68471674382064	1.29551953252725
H	2.29334896452089	-0.36519132271668	-0.98805911390298
H	2.92671272088467	-1.63541814960488	-2.03386588212422
H	2.25672664503891	-2.04540127077415	-0.45849445884276
H	0.57726588112026	-4.09669386459326	-2.06163138538230
H	1.11884717101471	-3.49096077343494	-3.62544379092591
H	-0.61050811430808	-3.57028115723649	-3.24745269069569
H	-2.07651192454245	-0.38903873425338	1.74623040333367
H	-2.61897319490190	-1.97011941817765	2.31305447905949
H	-3.41827383436721	-1.20917209884383	0.94660625237189
H	-2.95810013201373	-3.74178907283759	-0.75853867574655
H	-2.29841870590146	-4.45924962876463	0.71194996555939
H	-1.37640220702772	-4.51295940544690	-0.79242877706506
H	0.59503996867614	1.02827801906506	0.23793669397800
H	1.41043626709933	2.54555929216734	-0.15513936722291
H	0.67387035226007	1.58347200717111	-1.42764630508751
H	-0.94922024955195	2.95338084734593	2.35993205729206
H	-1.70953965538146	4.41948878537745	1.73606970828711
H	0.03607211164801	4.22749684297771	1.62813772114209
H	-3.67316579892104	1.20992403837628	2.62457478333131
H	-4.89931728415913	2.47654411055236	2.67282826675343
H	-3.18748079614219	2.89738739893211	2.74237851100110
H	-5.64188556012007	1.13502856063093	-1.08439404109914
H	-6.22443122455792	1.44847126944075	0.54005137143785
H	-5.13401235529586	0.10247612364886	0.26255326426904
H	-4.56651195963751	4.10990355238404	-1.19032416764002
H	-3.76408518599106	4.78625799587754	0.22432094280464
H	-5.44828907376094	4.27747795573521	0.33270931221954
H	0.94331337924322	-3.36748797937479	0.76110337376210
H	-0.02525405817530	-3.18729941192631	2.21730032805692
H	0.85719197829912	-1.80184603853730	1.57087662087921
H	0.41791326597818	0.60050859308636	-3.41455675886419
H	-0.14335491832400	-0.73047751010222	-4.45726837451220
H	1.55014749572293	-0.55796932344339	-4.07766447741813
H	-0.67823712693678	3.98710460531929	-2.38120691103743
H	-0.20371959371658	5.04611187896488	-1.06002551551198
H	-1.91083076984392	4.85144904274713	-1.45933292426699
N	-1.23944498178044	1.09964993634499	4.17946973843040
C	0.07391929421295	0.47791233109559	4.57564064210477
C	-0.14467826424842	-1.00731609650395	4.86591664402212

C	0.62188933908282	1.20606580884349	5.80398807112430
C	0.95405059948322	0.70059172770261	3.33971162141610
H	-0.85501696904995	-1.14203070621258	5.68409306876783
H	0.79062409085352	-1.48350817458261	5.15997834639534
H	-0.53045170593101	-1.51762015095119	3.98418113502894
H	-0.06277264512584	1.11494126993050	6.64959286536658
H	0.76474266514419	2.26570593893427	5.58803849067816
H	1.57849457681905	0.78085735621547	6.10789935062500
H	0.42668126997608	0.28315644105571	2.47936877738006
C	2.35719188421558	0.10983803470658	3.41234869790036
H	1.01782886512873	1.77713871514951	3.16332877435771
H	2.30903082482042	-0.97602549890896	3.52278212364548
H	2.89129429319332	0.48746451345453	4.28820523359905
C	3.17997541834307	0.43683194680825	2.15864564709316
H	2.64595242229427	0.07859811431474	1.27699913453308
C	4.55265262063493	-0.17826777177517	2.20253586045677
H	3.26095317338704	1.52231575455146	2.05540336982069
C	5.63063800936258	0.50949076558194	2.75951203266484
C	6.88652576223116	-0.07960083659614	2.84797575771041
C	7.08414037786736	-1.37296271142473	2.37806617511350
C	4.76522029492350	-1.47436352416527	1.73201810327898
C	6.01828431170928	-2.06841942734700	1.81781917819596
H	7.71181333311464	0.47248136979641	3.28027110445250
H	8.06176405609602	-1.83333003387920	2.44345045675307
H	3.94030118361272	-2.01840064434746	1.28840523272005
H	6.16401423152961	-3.07377647230834	1.44257946471332
H	5.48360549760682	1.51972645385467	3.12471739340436
N	-2.61854995953838	0.14715657297250	-3.46950892949956
C	-3.26765156176771	1.24772081371274	-4.26959866862004
C	-3.36292279196278	0.61981801599656	-5.66943245611884
C	-2.38008607117528	2.48109269900671	-4.22334559251412
C	-4.64413996095212	1.58778043417823	-3.70968444425270
C	-3.73817697964739	-0.83320219099019	-5.39426115753020
H	-2.38525030296148	0.67874888317687	-6.15601452353542
H	-4.08293733794201	1.13381139431389	-6.30666663212910
C	-3.01807948015098	-1.17450834165002	-4.06036120752581
H	-3.43738787951747	-1.51266772445932	-6.19051555151881
H	-4.81536134182670	-0.94041638130533	-5.26813139921313
H	-1.63170862037310	0.23185751307229	-3.69236348558049
H	-2.09375309824326	-1.71117794529132	-4.27453279803170
C	-3.85589915663984	-2.06376254969439	-3.17827658894780
H	-1.37228958006634	2.25927568905044	-4.58298599728356
H	-2.31253910595479	2.86433887021431	-3.20753184144843
H	-2.79495612470237	3.26694518839320	-4.85575412145400
H	-5.31020190656461	0.72597486994113	-3.68822144433372
H	-5.10981785459824	2.35900373438842	-4.32484353629907
H	-4.54801709722652	1.97522917984159	-2.69782227273736
C	-3.93413821733139	-3.42150833175081	-3.48916353712581
C	-4.76336662857873	-4.26833001793627	-2.76719598544314
C	-5.52143589826034	-3.77186572986129	-1.71138593707725
C	-5.43631906635132	-2.42540348547886	-1.38458430255069

C	-4.61126299260586	-1.58028469114308	-2.11804802552735
H	-4.57418218021654	-0.53386328354539	-1.86090884521449
H	-6.15954460966177	-4.43317096738813	-1.14015492092851
H	-6.01010217648054	-2.02464917310138	-0.55913170809886
H	-3.33646624797917	-3.81973633499985	-4.30098443491652
H	-4.80530531447882	-5.32062324149485	-3.01715377038760
N	-2.13610530448887	1.05422164713920	5.00805843609771
N	-3.03076789576579	1.06051999998142	5.70044740336928

NMR spectra

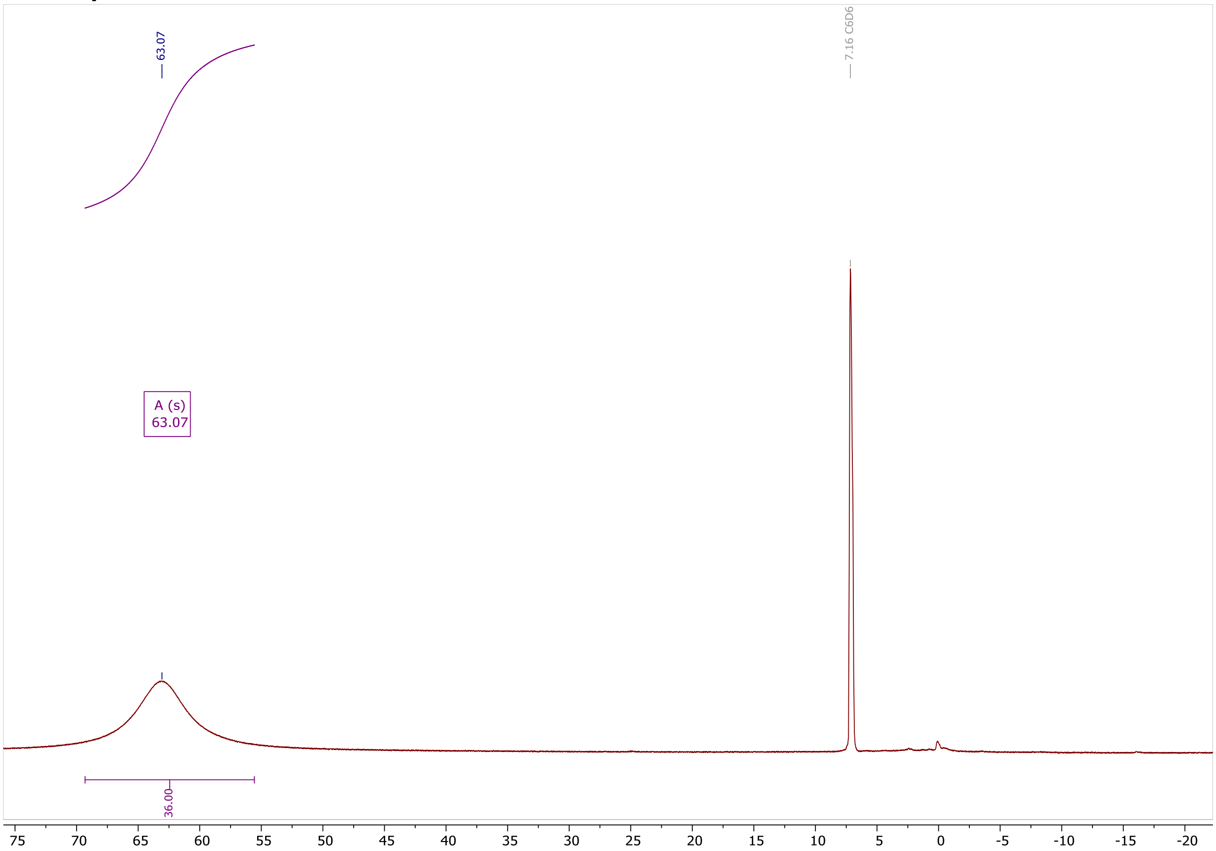

Figure S41: ^{1}H NMR spectrum of $Fe(HMDS)_2$ in C_6D_6.

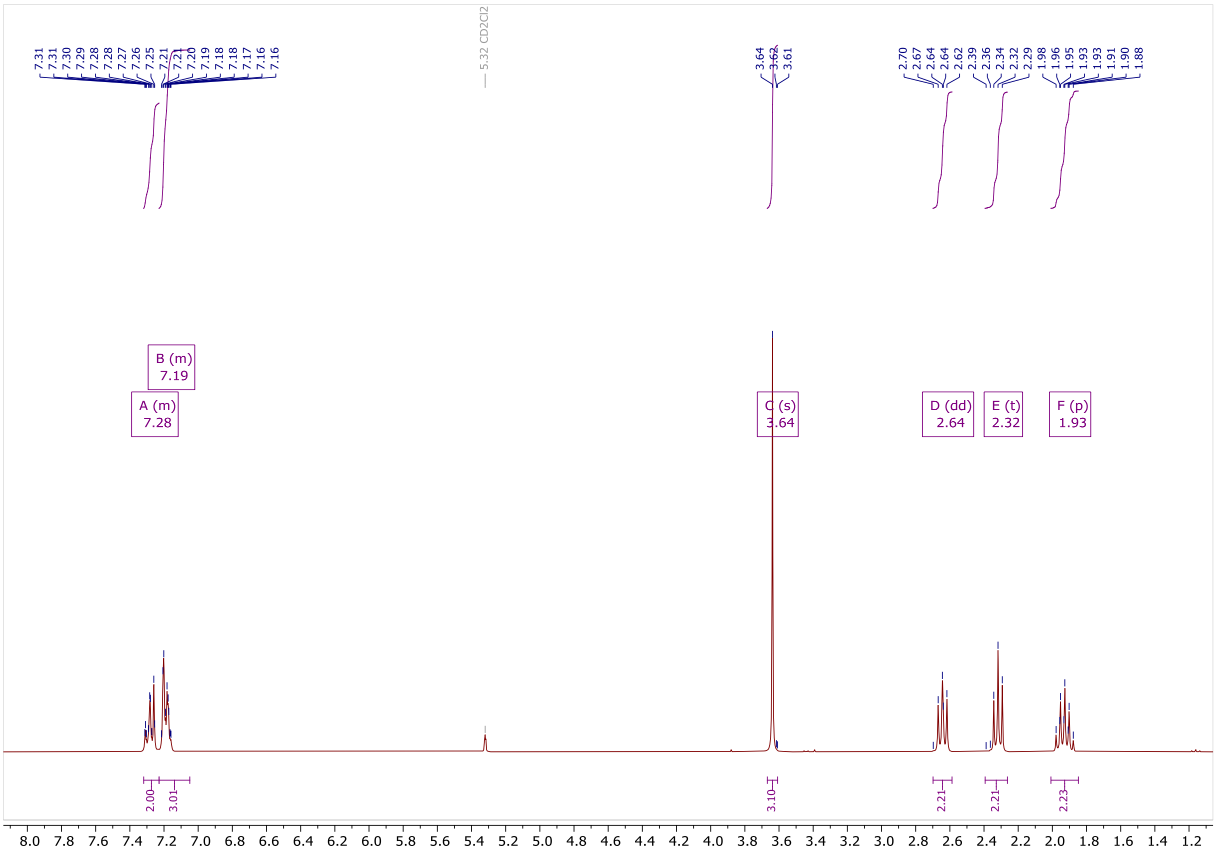

Figure S42: ^{1}H NMR spectrum of methyl 4-phenylbutanoate in CD_2Cl_2.

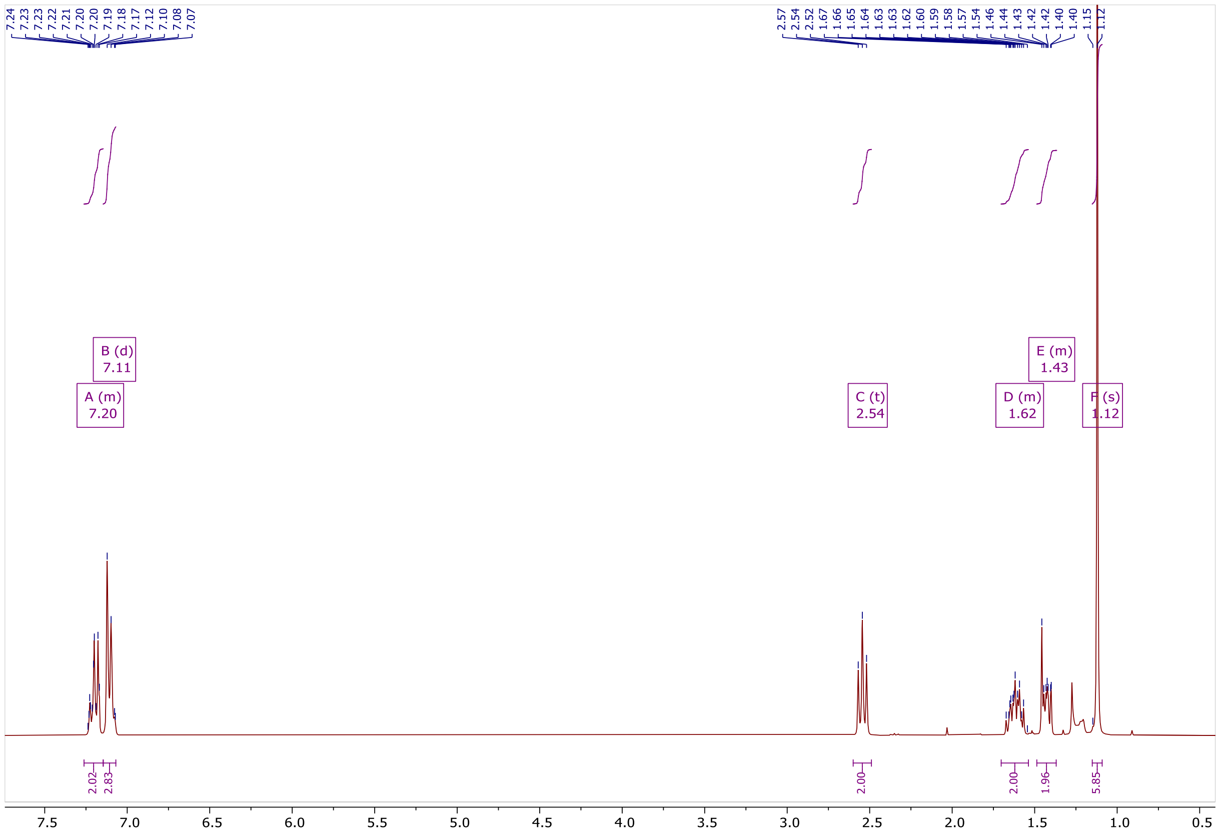

Figure S43: ^{1}H NMR spectrum of 2-methyl-5-phenylpentan-2-ol in $CDCl_3$.

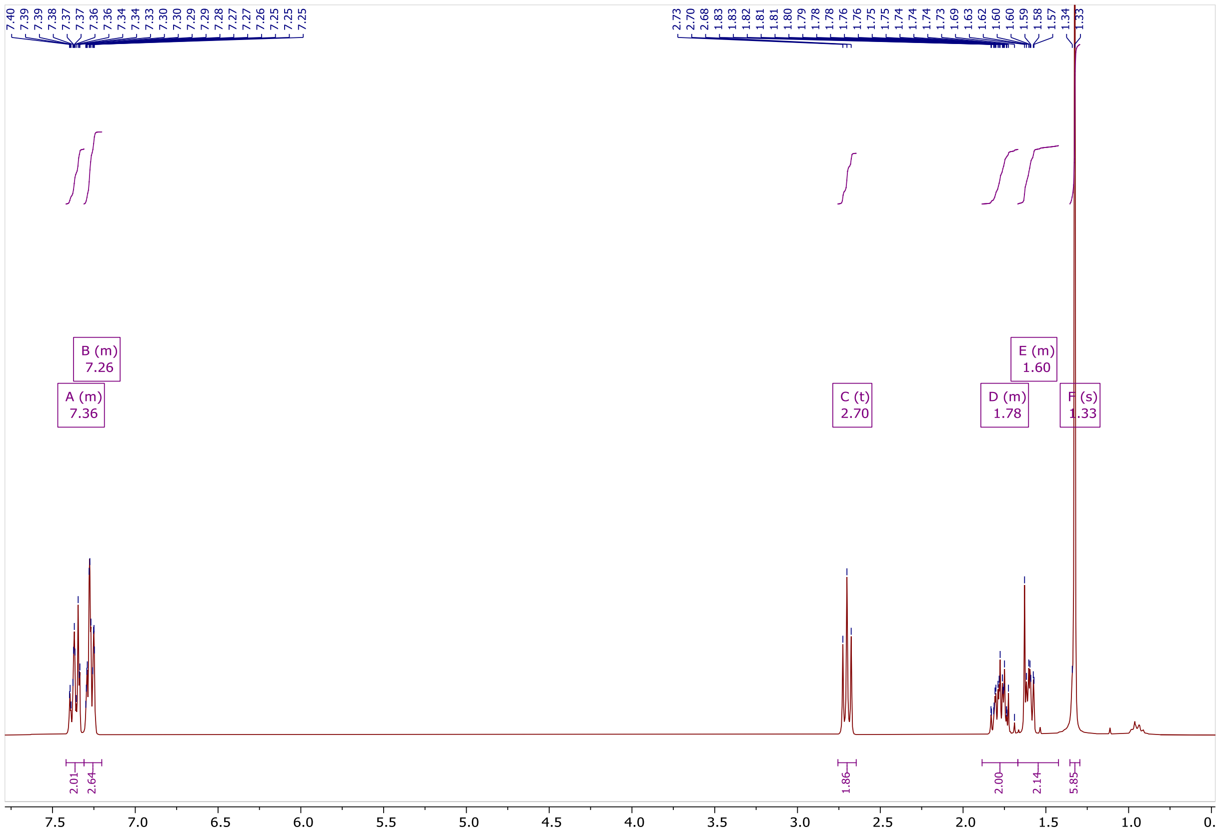

Figure S44: ^{1}H NMR spectrum of substrate **1a** in $CDCl_3$.

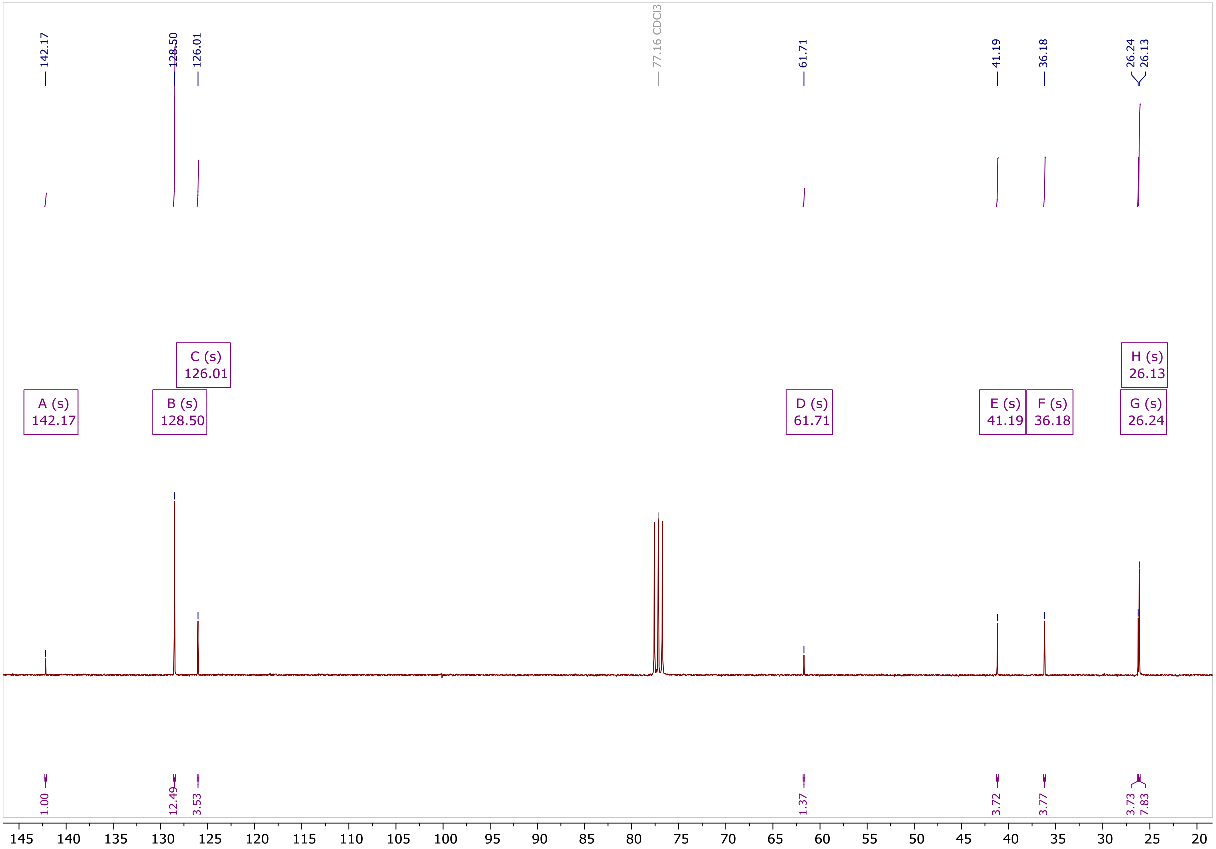

Figure S45: ^{13}C NMR spectrum of substrate **1a** in $CDCl_3$.

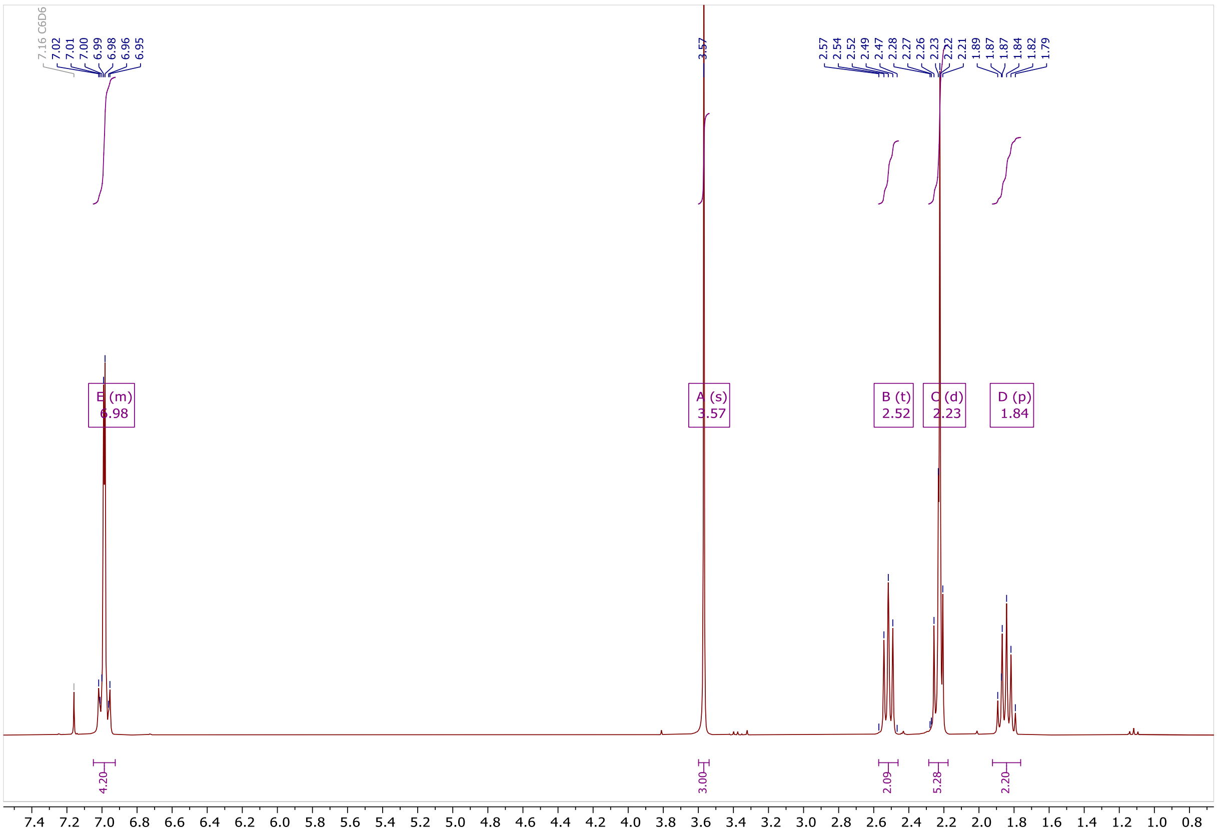

Figure S46: ^{1}H NMR spectrum of 4-(p-tolyl)butanoate in $CDCl_3$.

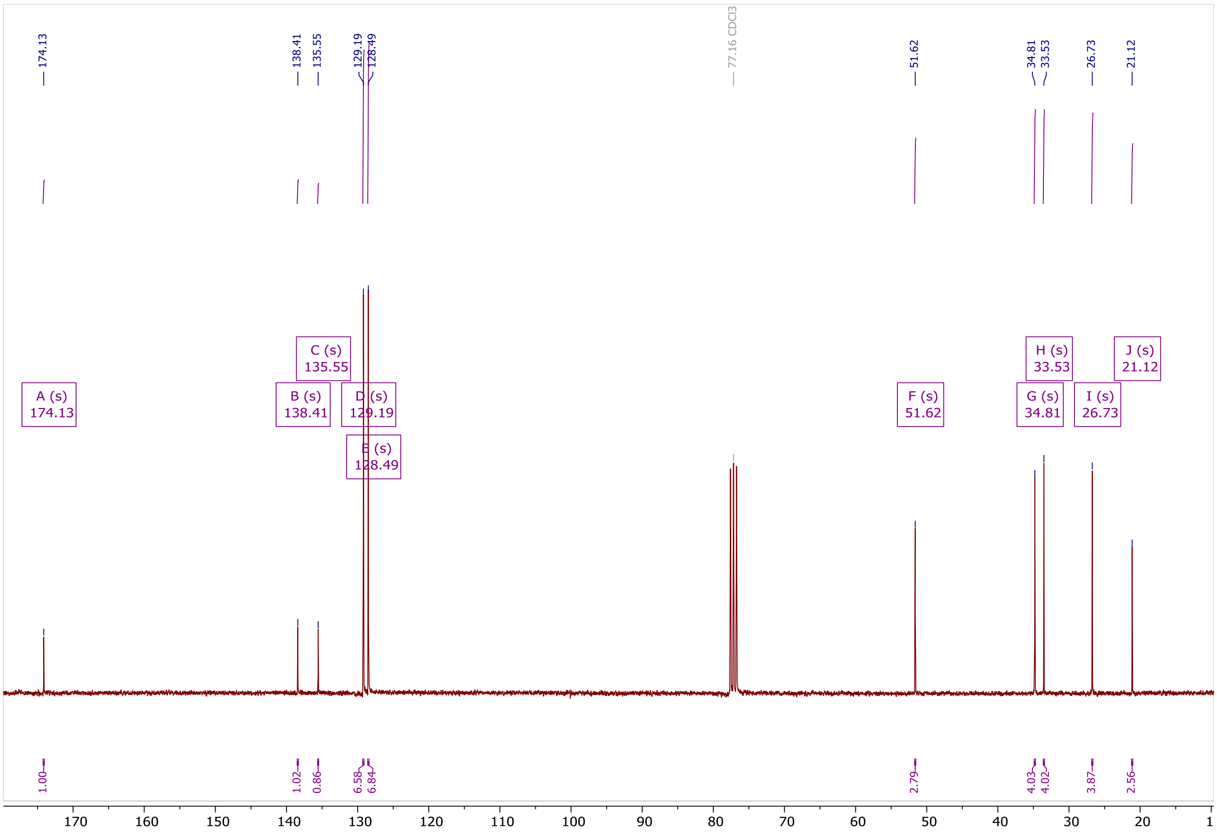

Figure S47: ^{13}C NMR spectrum of 4-(p-tolyl)butanoate in $CDCl_3$.

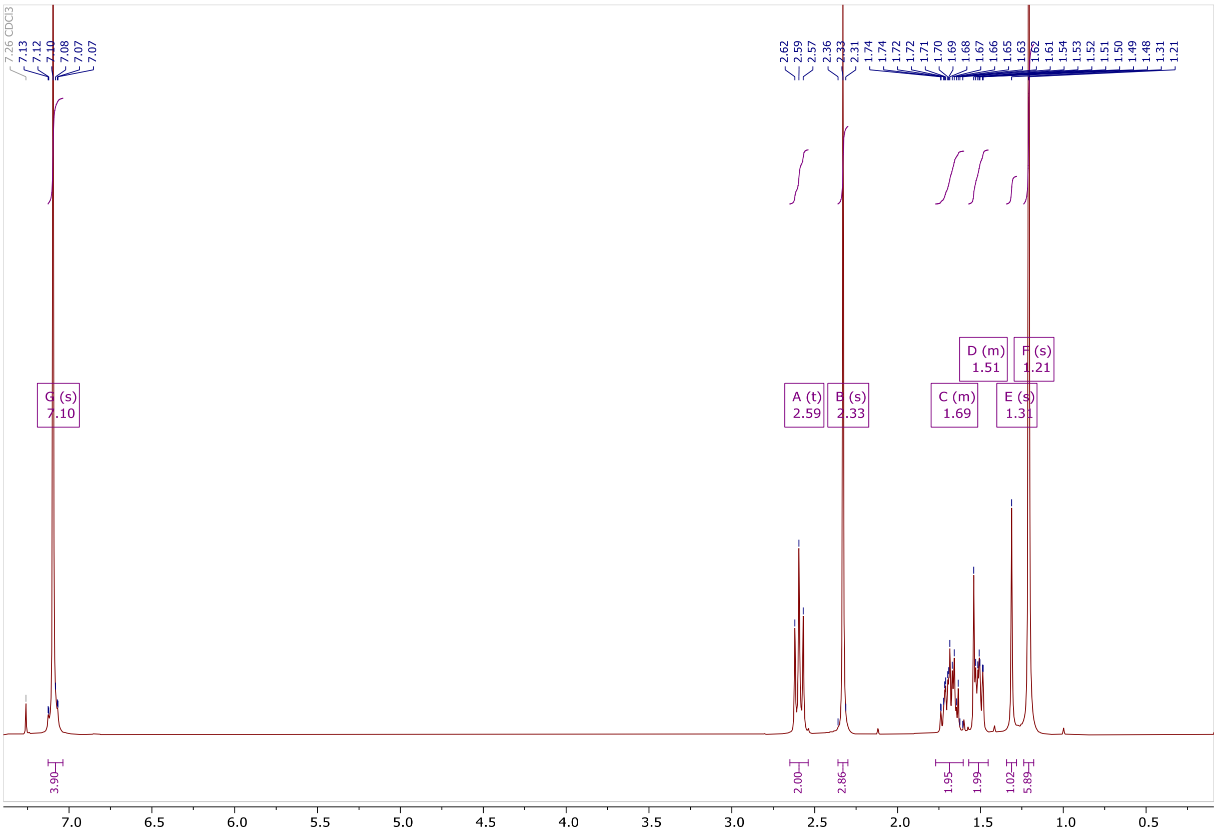

Figure S48: ^{1}H NMR spectrum of 2-methyl-5-(p-tolyl)pentan-2-ol in $CDCl_3$.

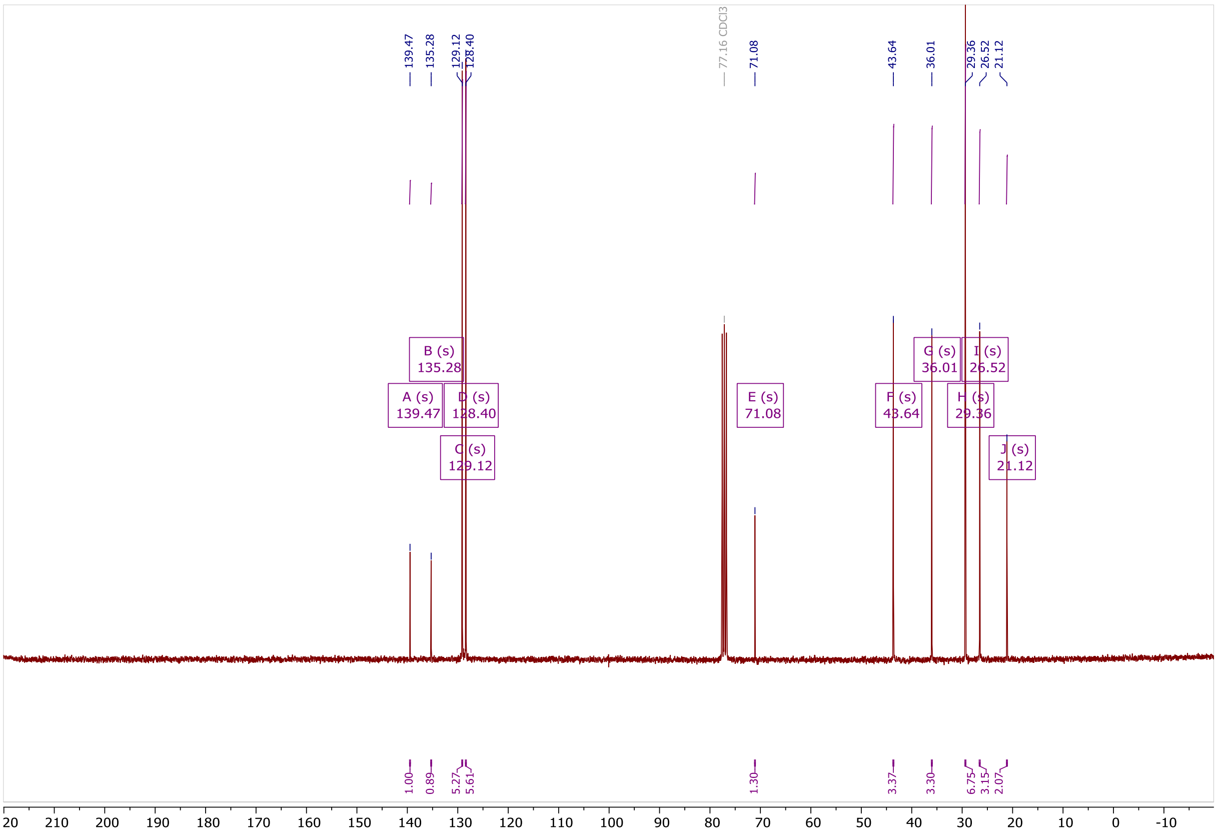

Figure S49: ^{13}C NMR spectrum of 2-methyl-5-(p-tolyl)pentan-2-ol in $CDCl_3$.

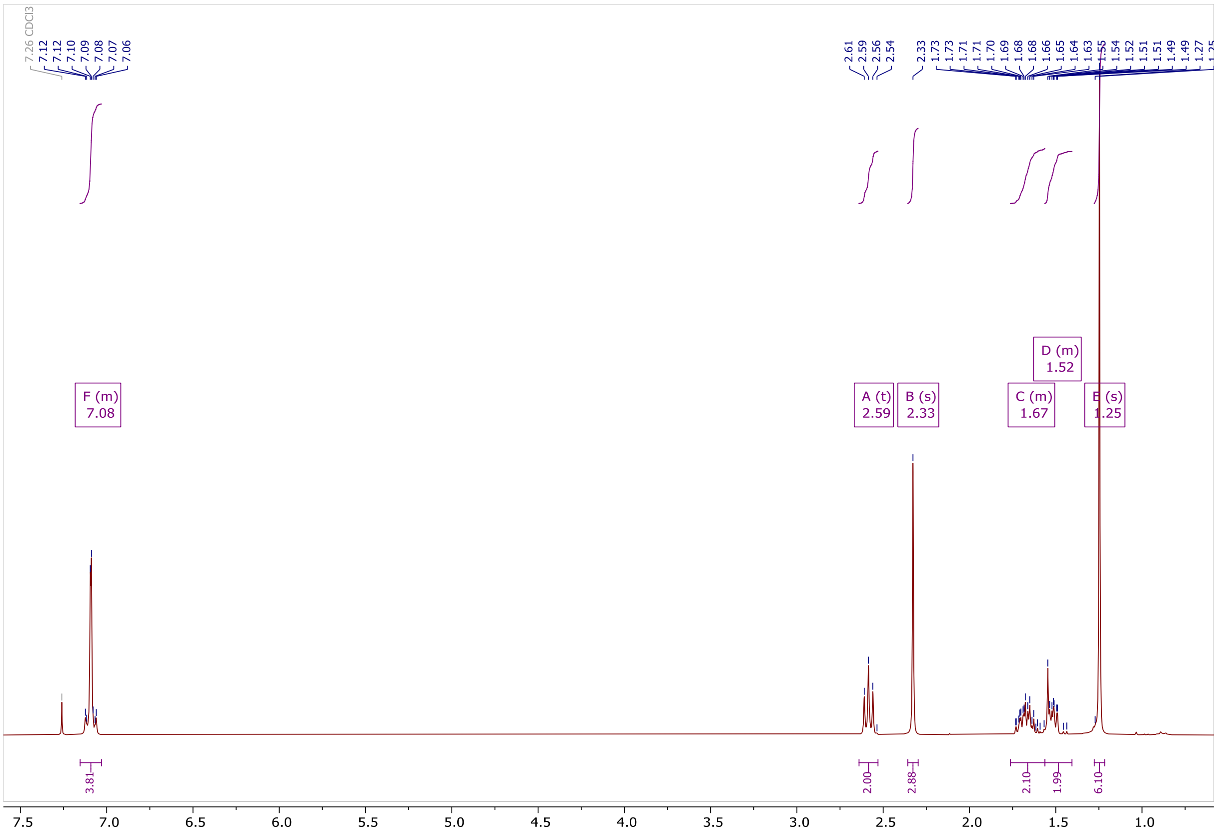

Figure S50: ^{1}H NMR spectrum of substrate **5a** in $CDCl_3$.

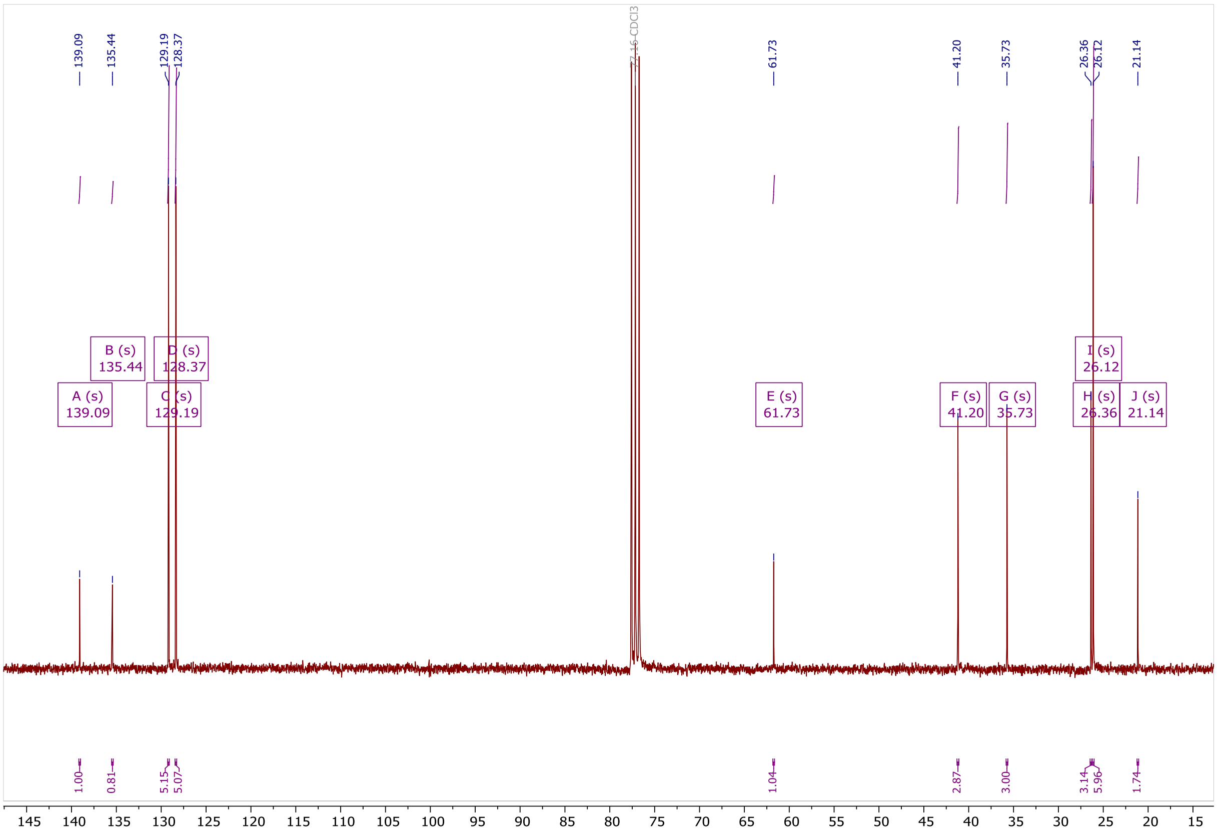

Figure S51: ^{13}C NMR spectrum of substrate **5a** in $CDCl_3$.

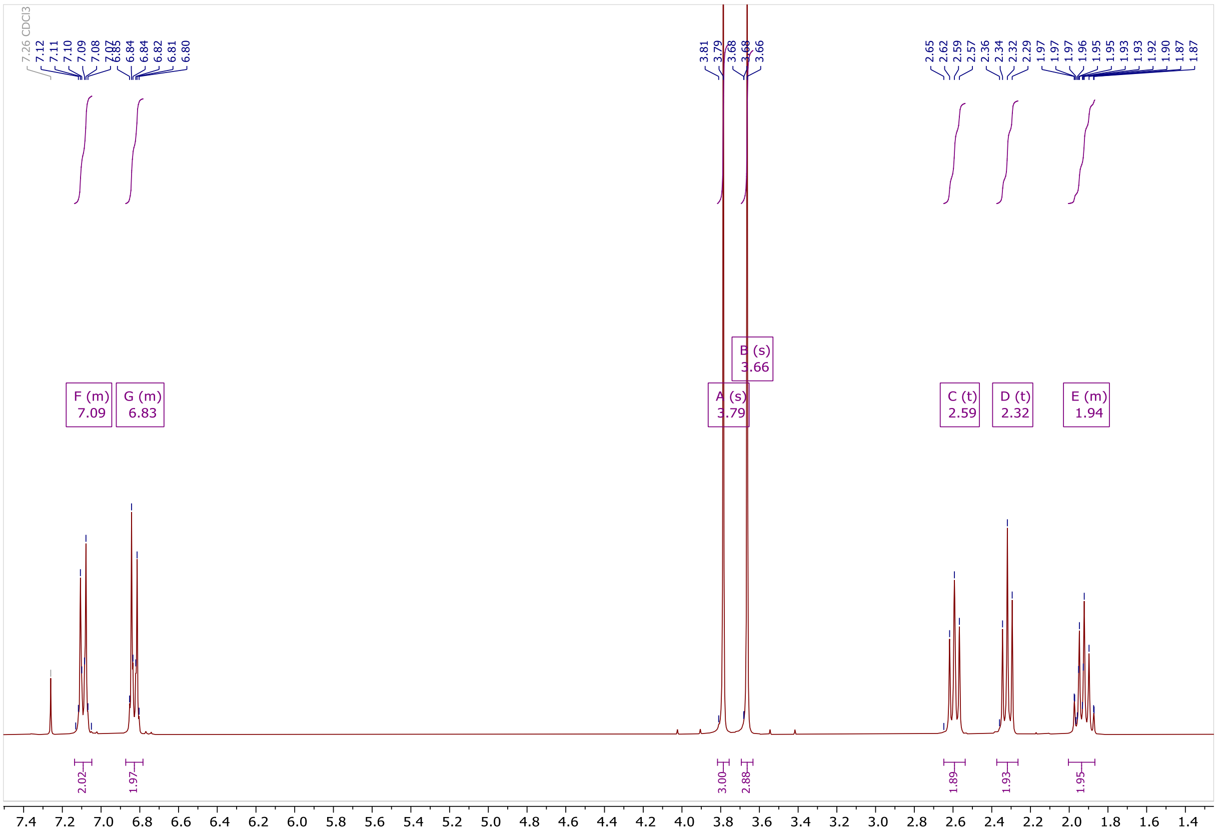

Figure S52: ^{1}H NMR spectrum of methyl 4-(4-methoxyphenyl)butanoate in $CDCl_3$.

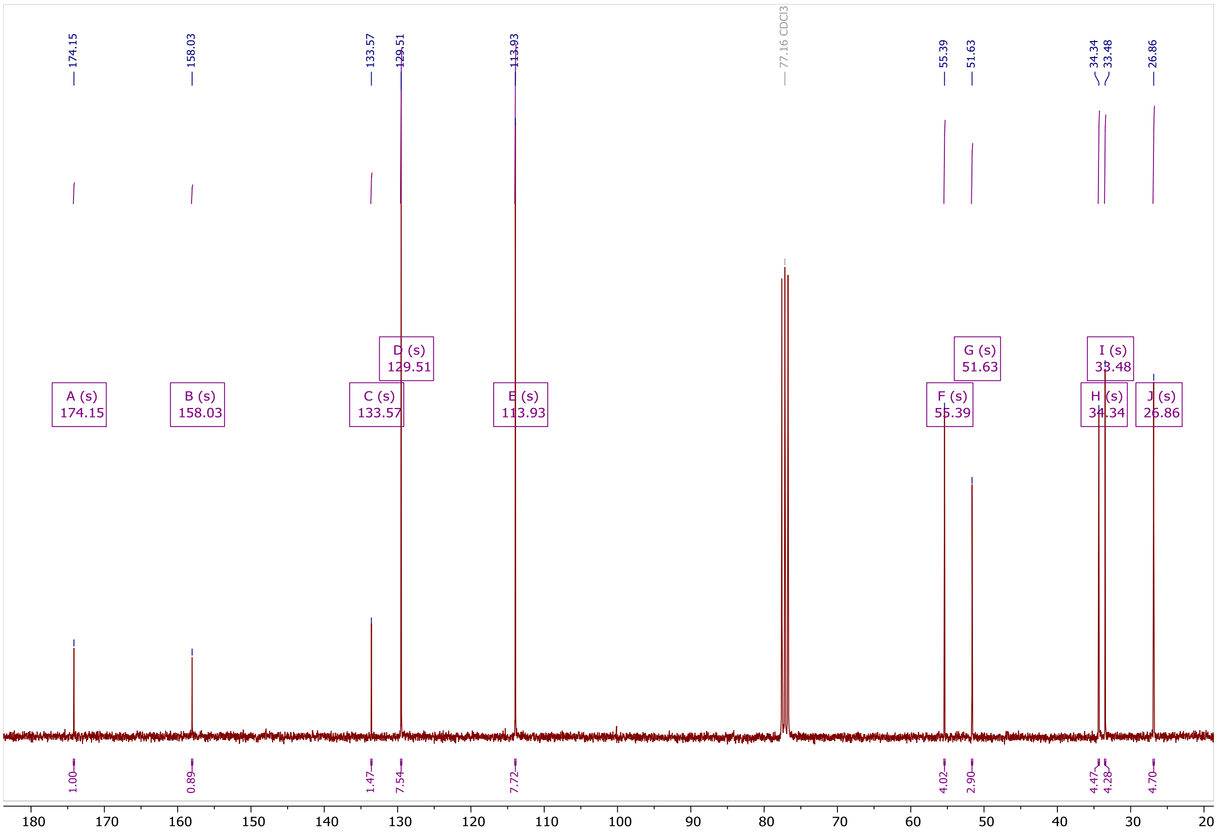

Figure S53: ^{13}C NMR spectrum of methyl 4-(4-methoxyphenyl)butanoate in $CDCl_3$.

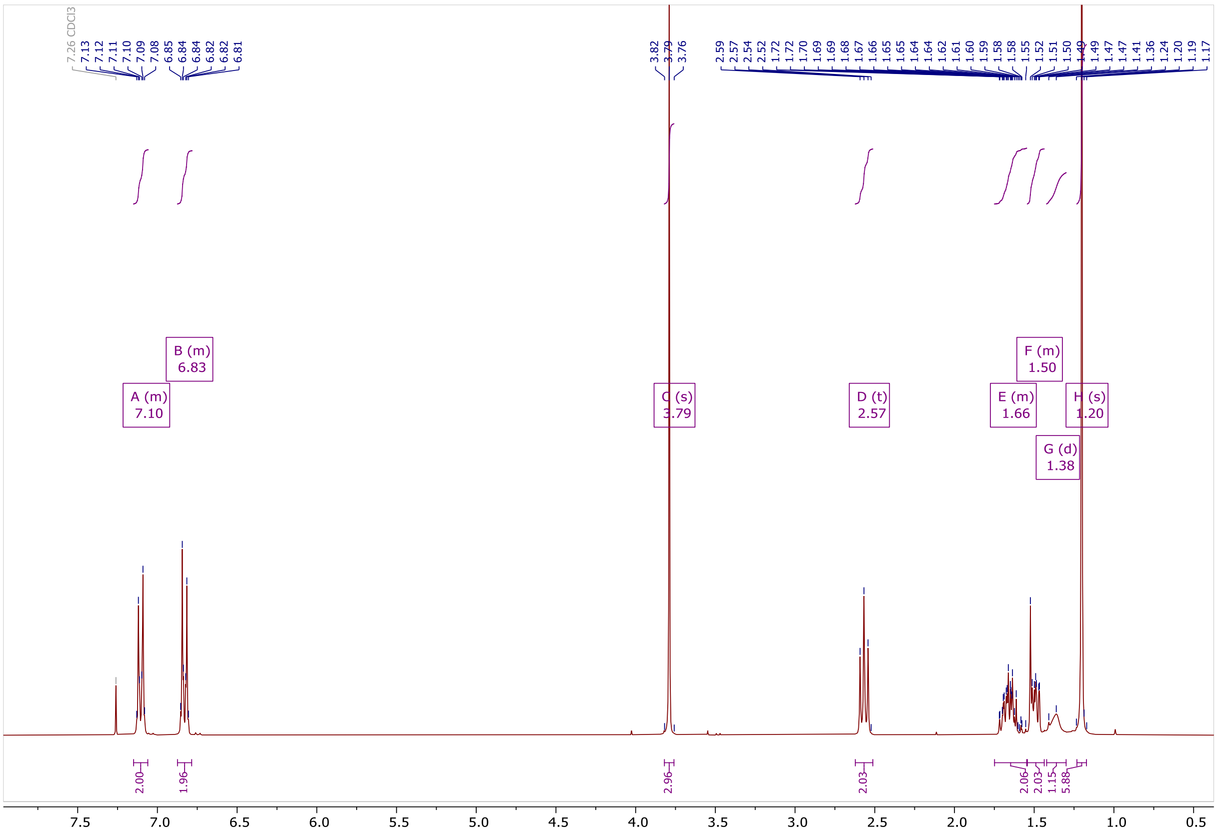

Figure S54: ^{1}H NMR spectrum of 5-(4-methoxyphenyl)-2-methylpentan-2-ol in $CDCl_3$.

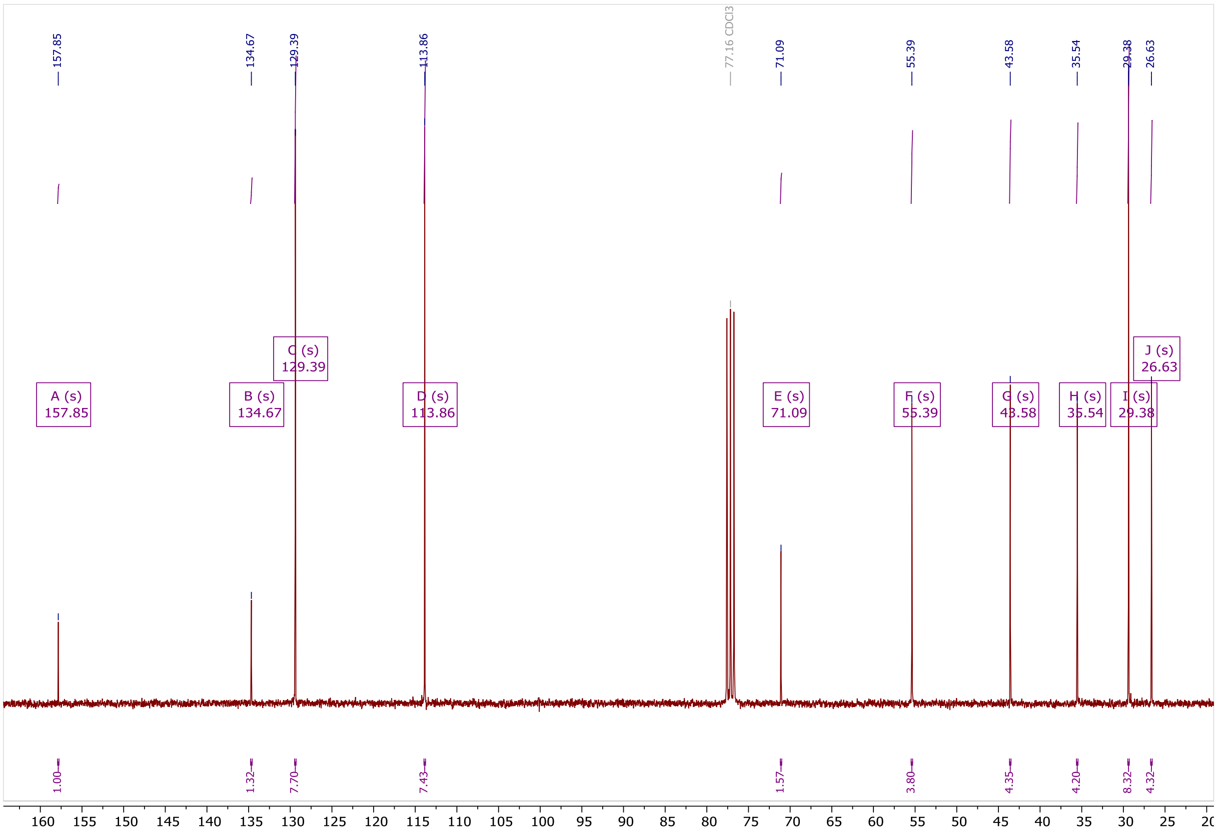

Figure S55: ^{13}C NMR spectrum of 5-(4-methoxyphenyl)-2-methylpentan-2-ol in $CDCl_3$.

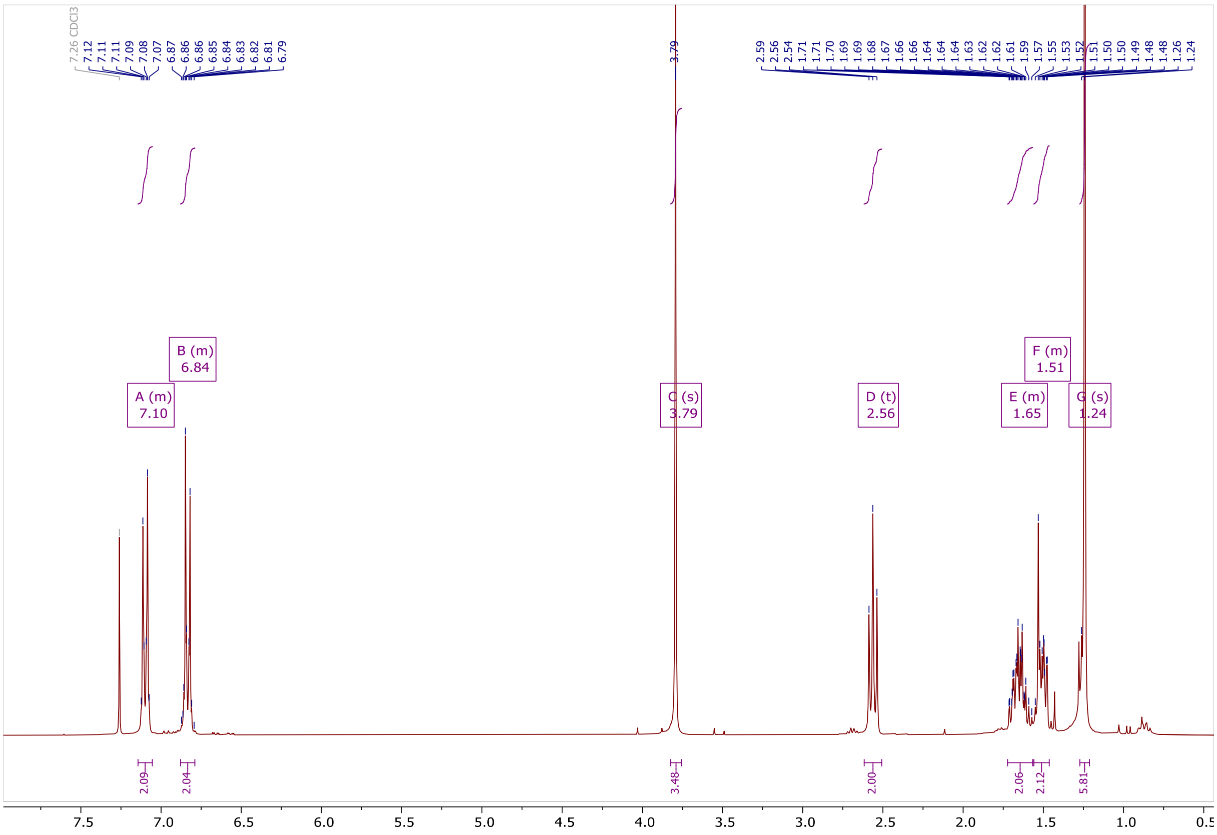

Figure S56: ^{1}H NMR spectrum of substrate **6a** in $CDCl_3$.

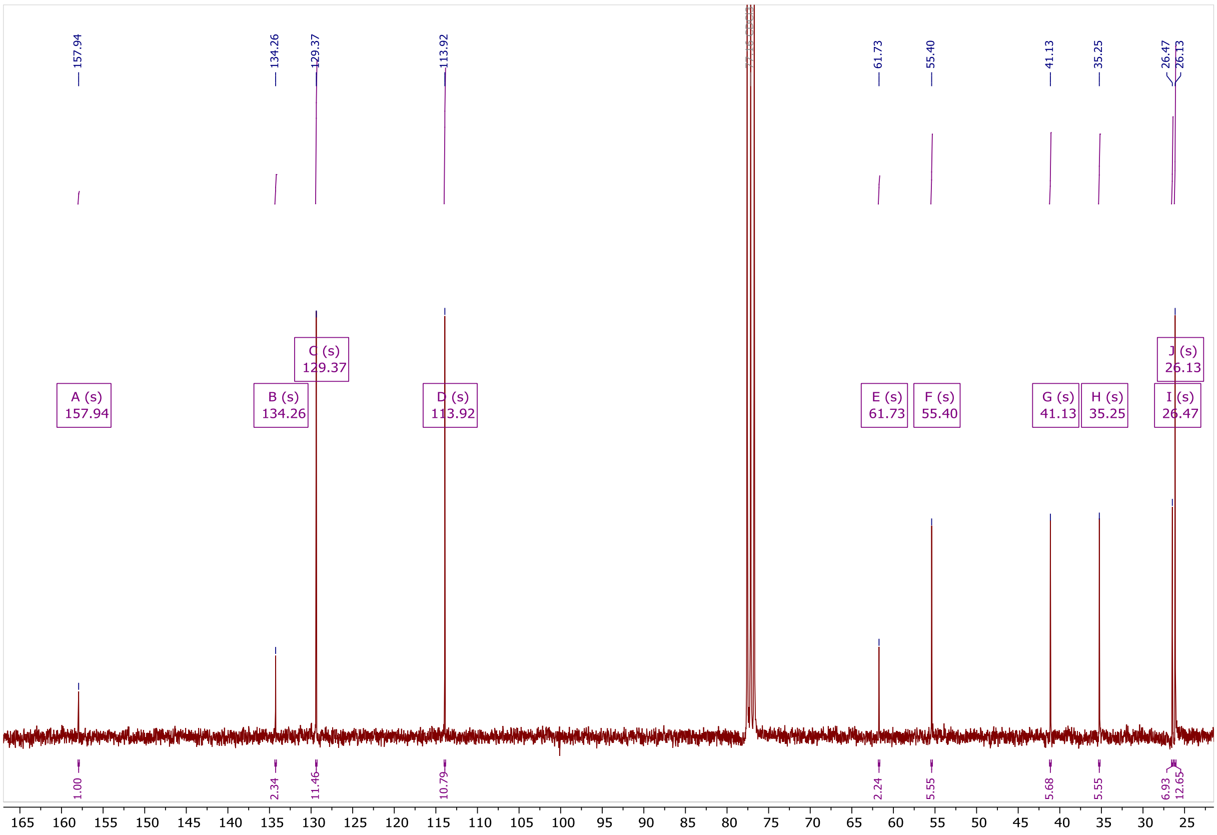

Figure S57: ^{13}C NMR spectrum of substrate **6a** in $CDCl_3$.

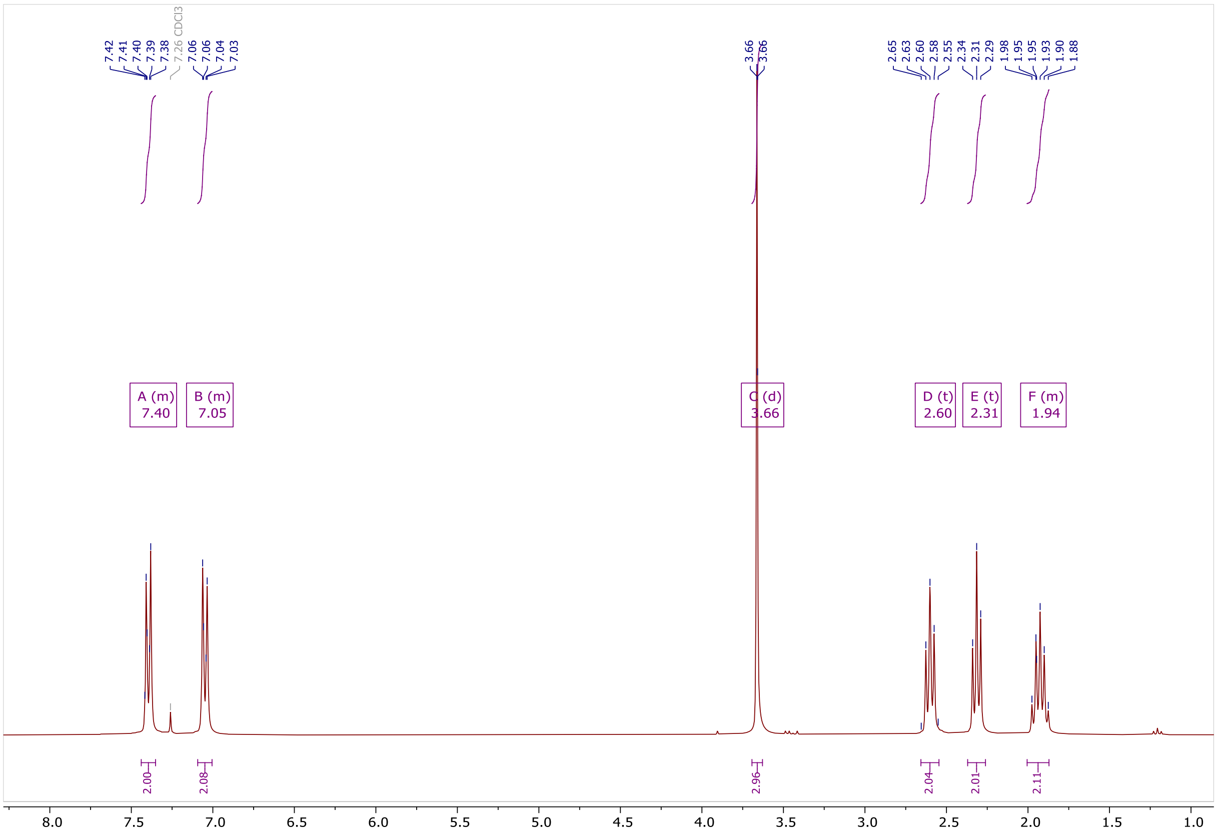

Figure S58: ^{1}H NMR spectrum of methyl 4-(4-bromophenyl)butanoate in $CDCl_3$.

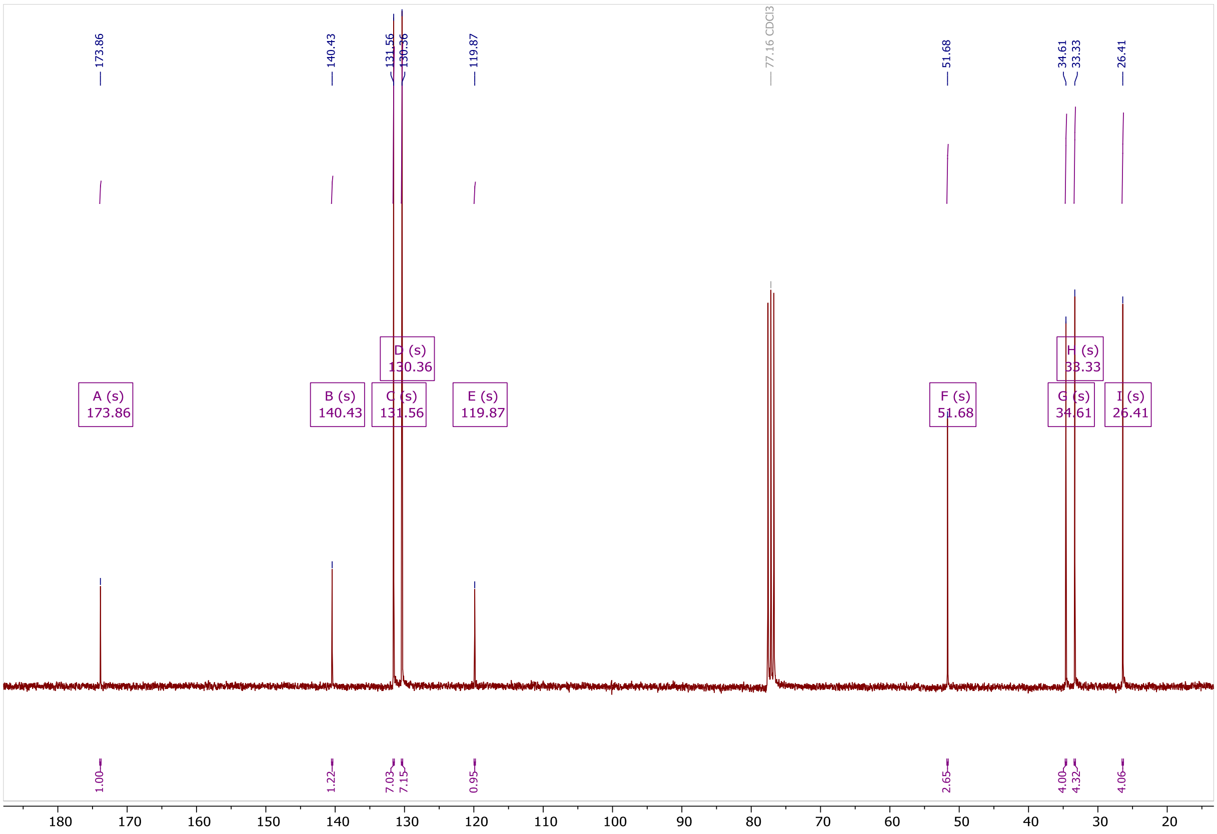

Figure S59: ^{13}C NMR spectrum of methyl 4-(4-bromophenyl)butanoate in $CDCl_3$.

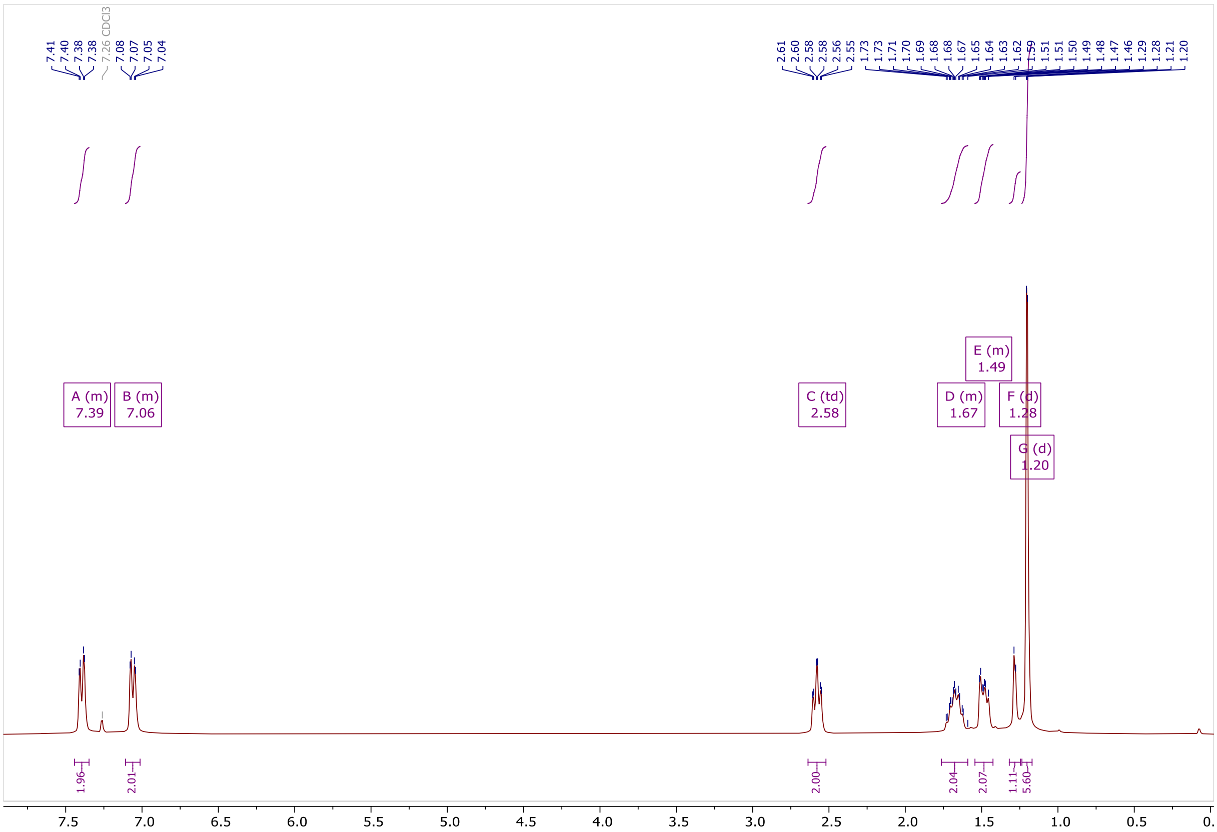

Figure S60: ^{1}H NMR spectrum of 5-(4-bromophenyl)-2-methylpentan-2-ol in $CDCl_3$.

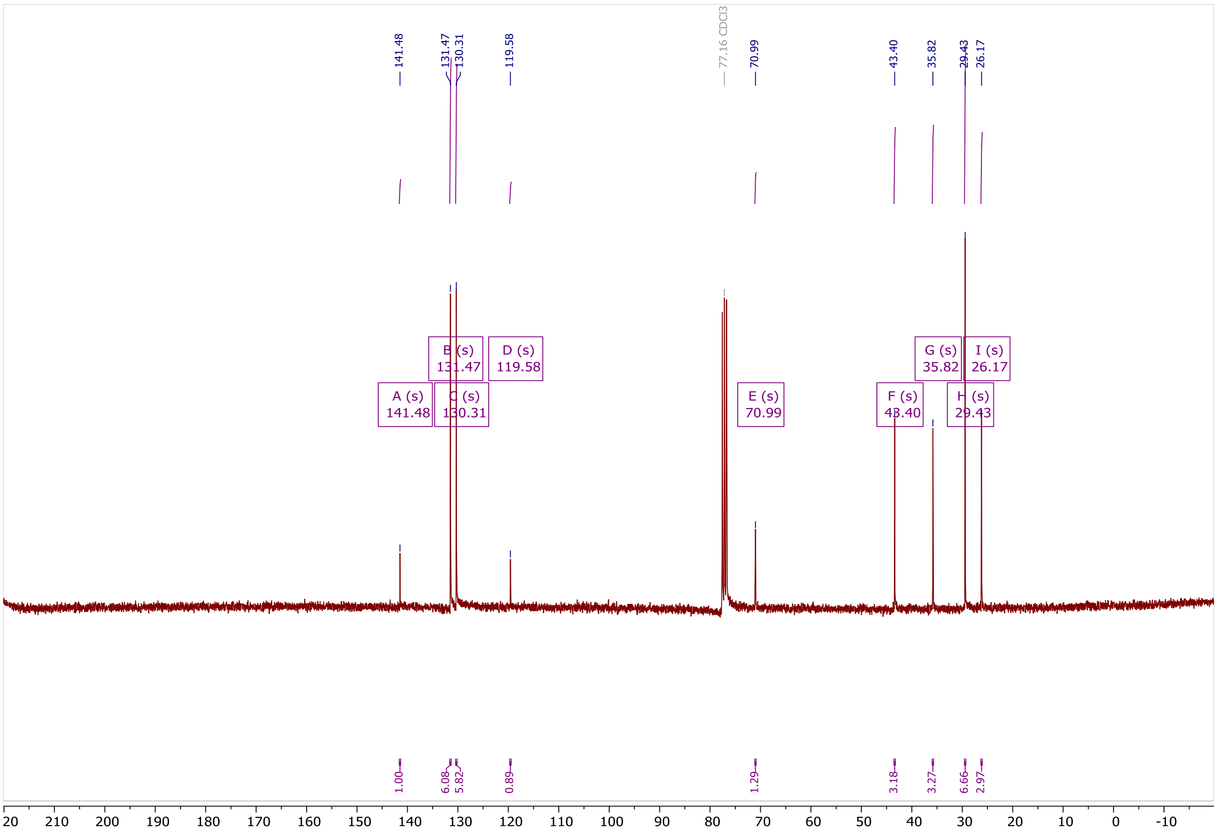

Figure S61: ^{13}C NMR spectrum of 5-(4-bromophenyl)-2-methylpentan-2-ol in $CDCl_3$.

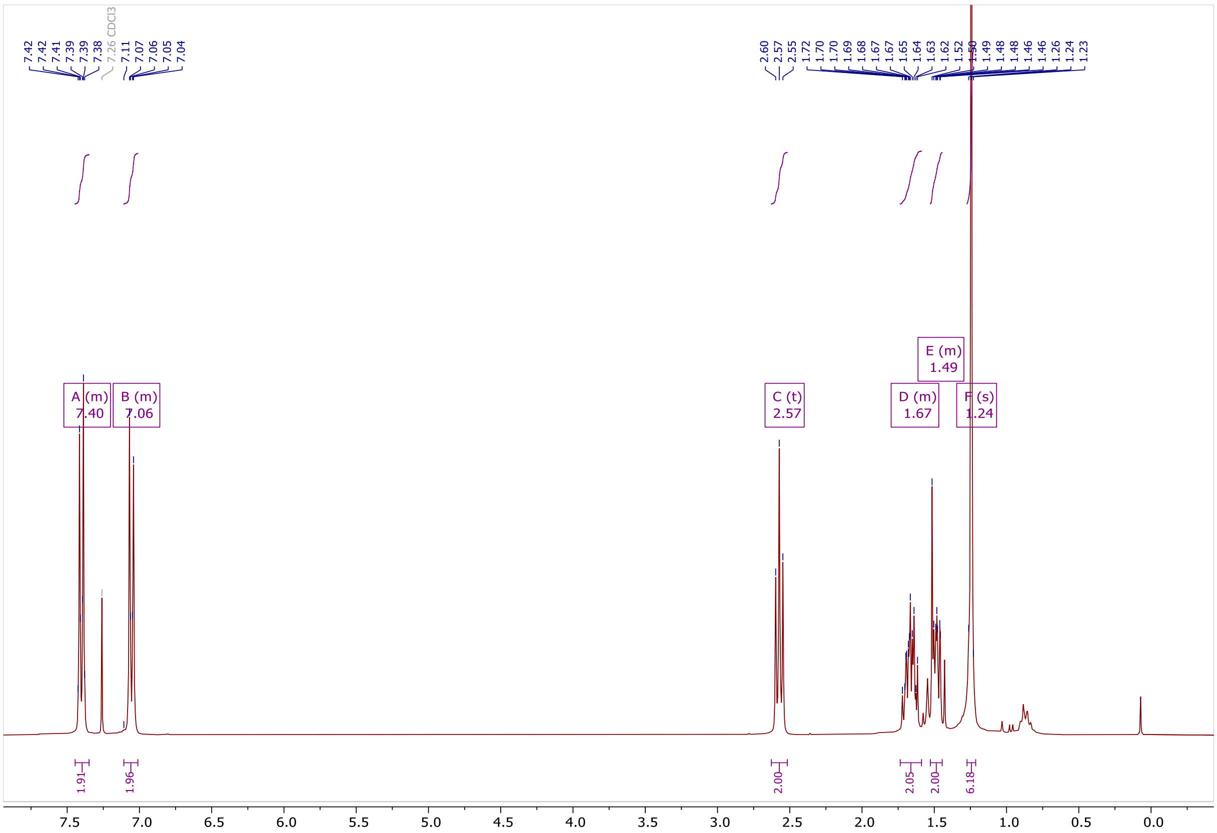

Figure S62: ^{1}H NMR spectrum of substrate **7a** in $CDCl_3$.

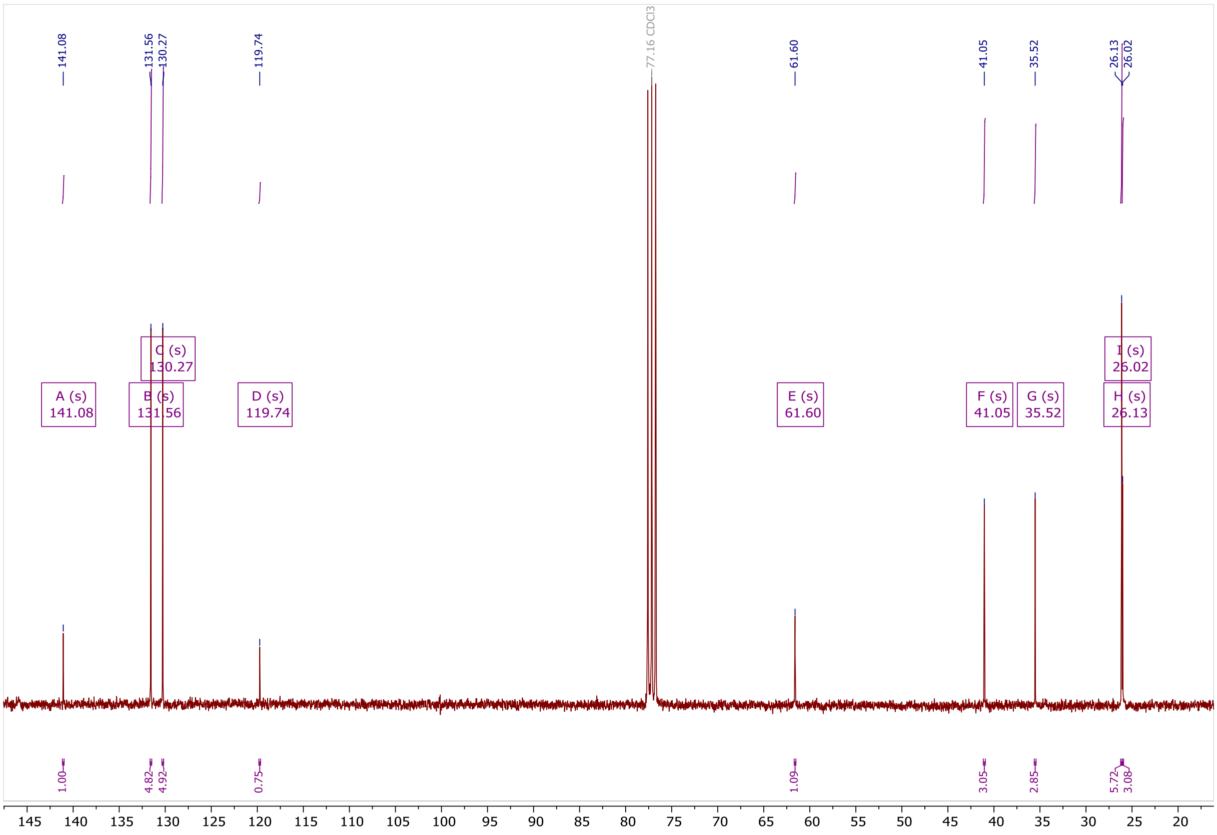

Figure S63: ^{13}C NMR spectrum of substrate **7a** in $CDCl_3$.

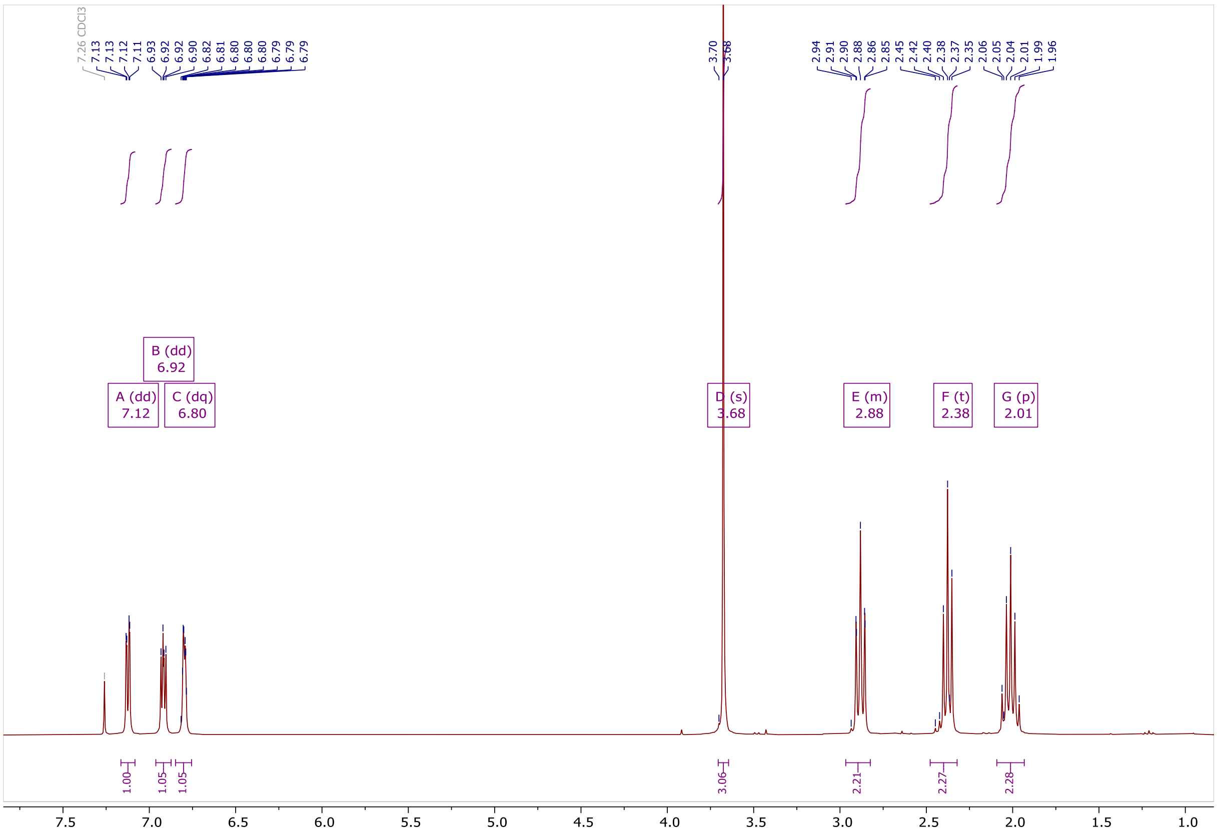

Figure S64: ^{1}H NMR spectrum of methyl 4-(thiophen-2-yl)butanoate in $CDCl_3$.

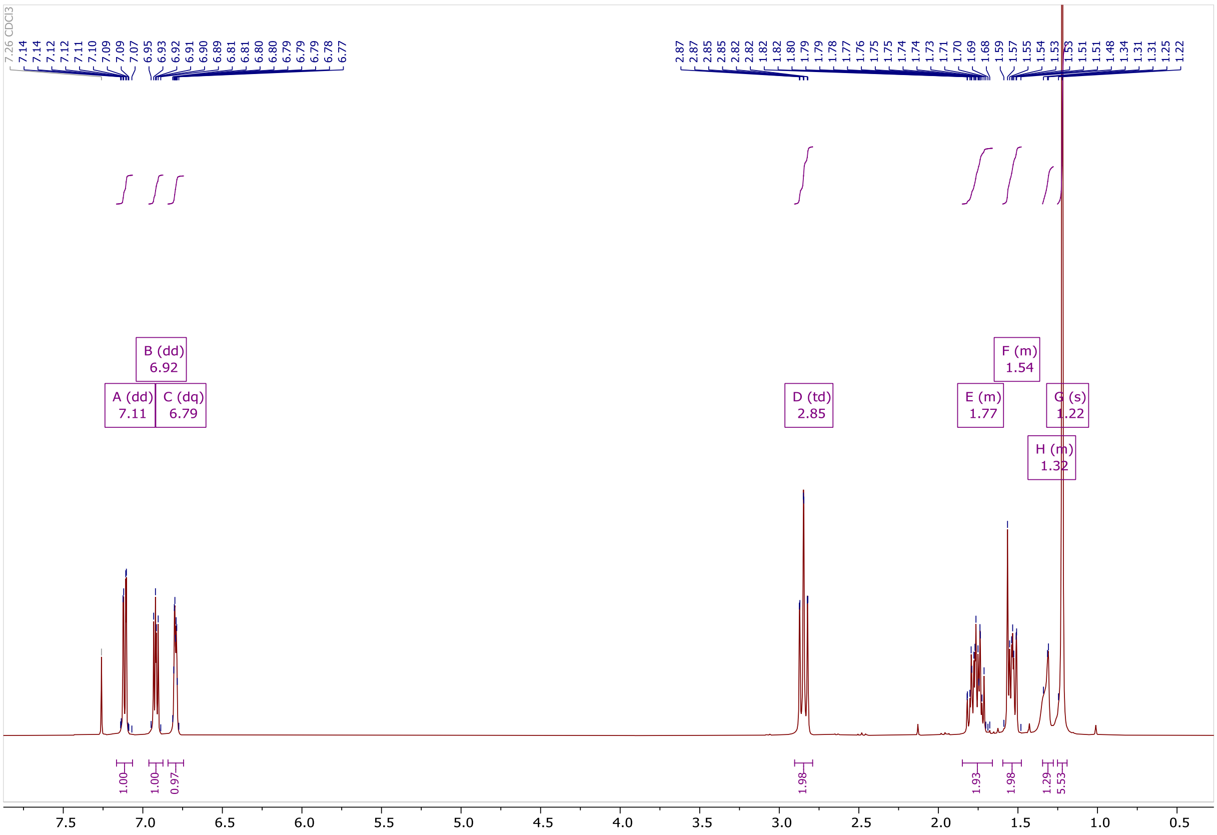

Figure S65: ^{1}H NMR spectrum of 2-methyl-5-(thiophen-2-yl)pentan-2-ol in $CDCl_3$.

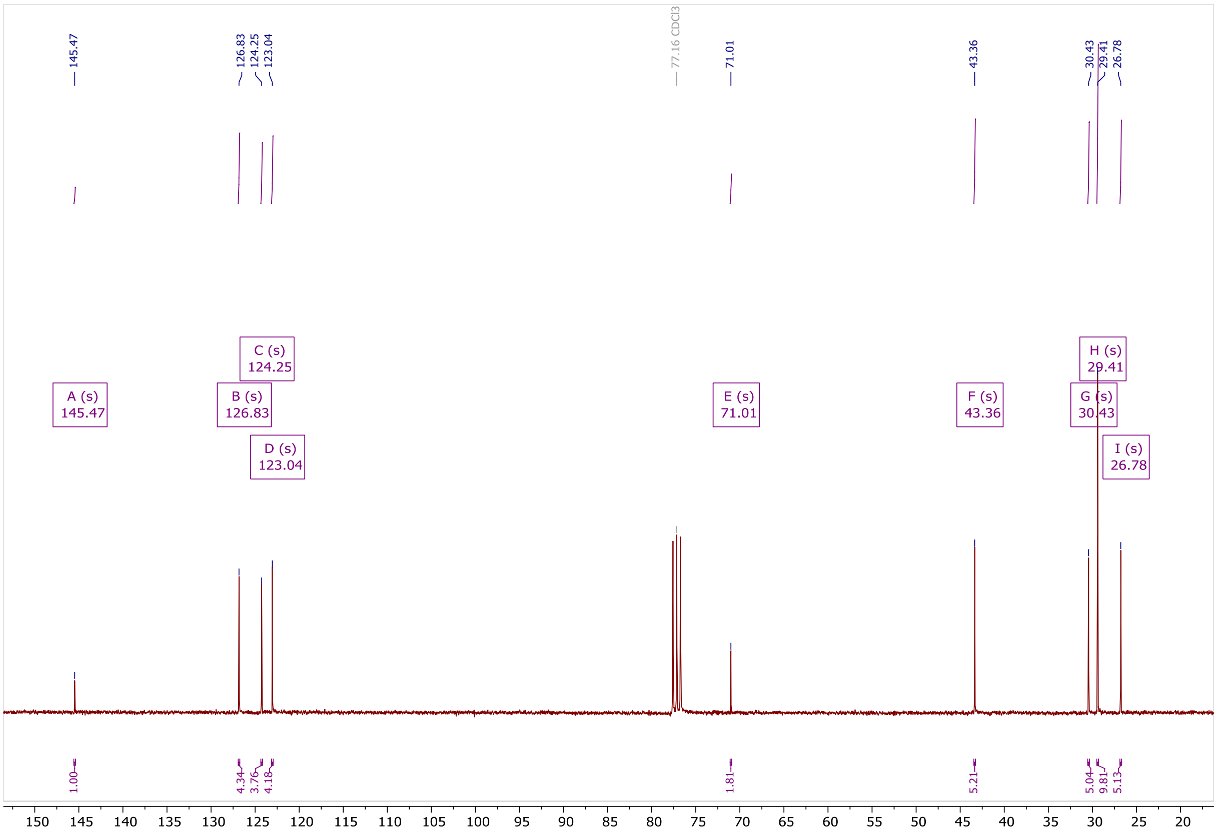

Figure S66: ^{13}C NMR spectrum of 2-methyl-5-(thiophen-2-yl)pentan-2-ol in $CDCl_3$.

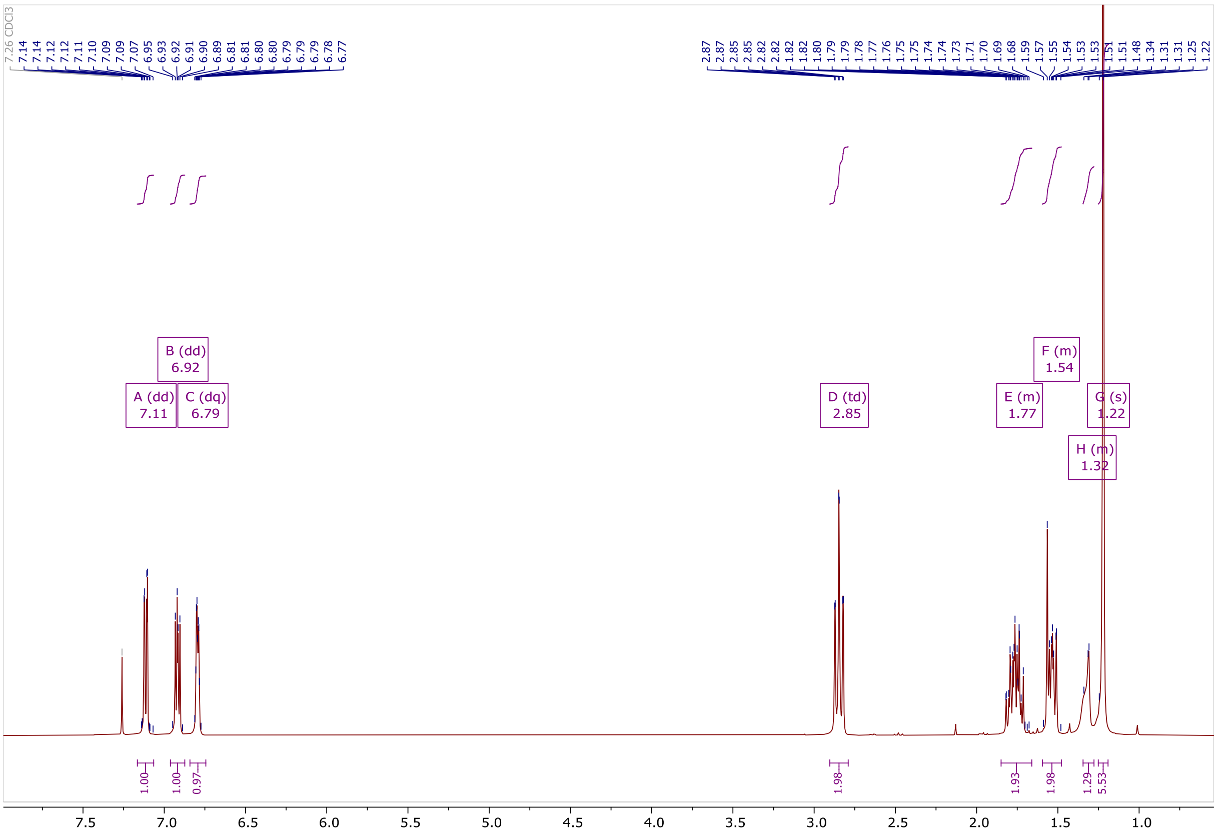

Figure S67: ^{1}H NMR spectrum of substrate **8a** in $CDCl_3$.

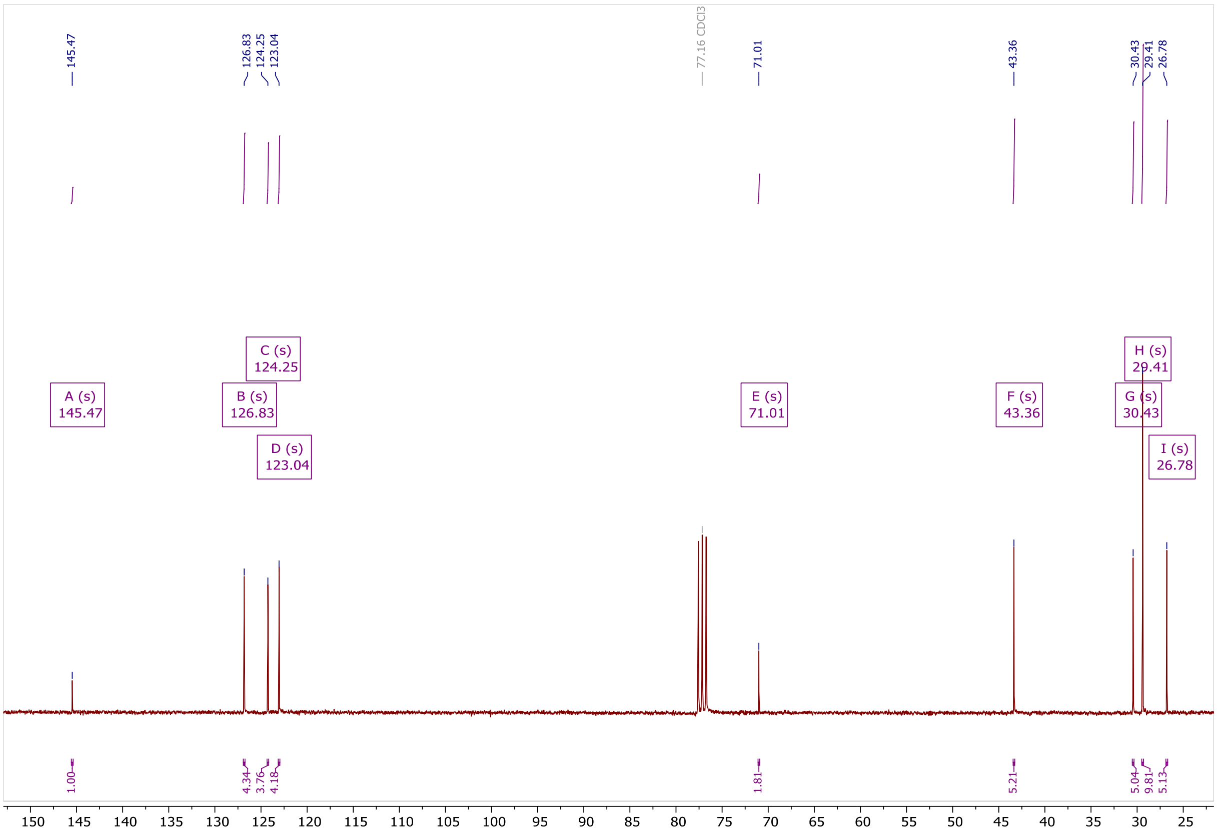

Figure S68: ^{13}C NMR spectrum of substrate **8a** in $CDCl_3$.

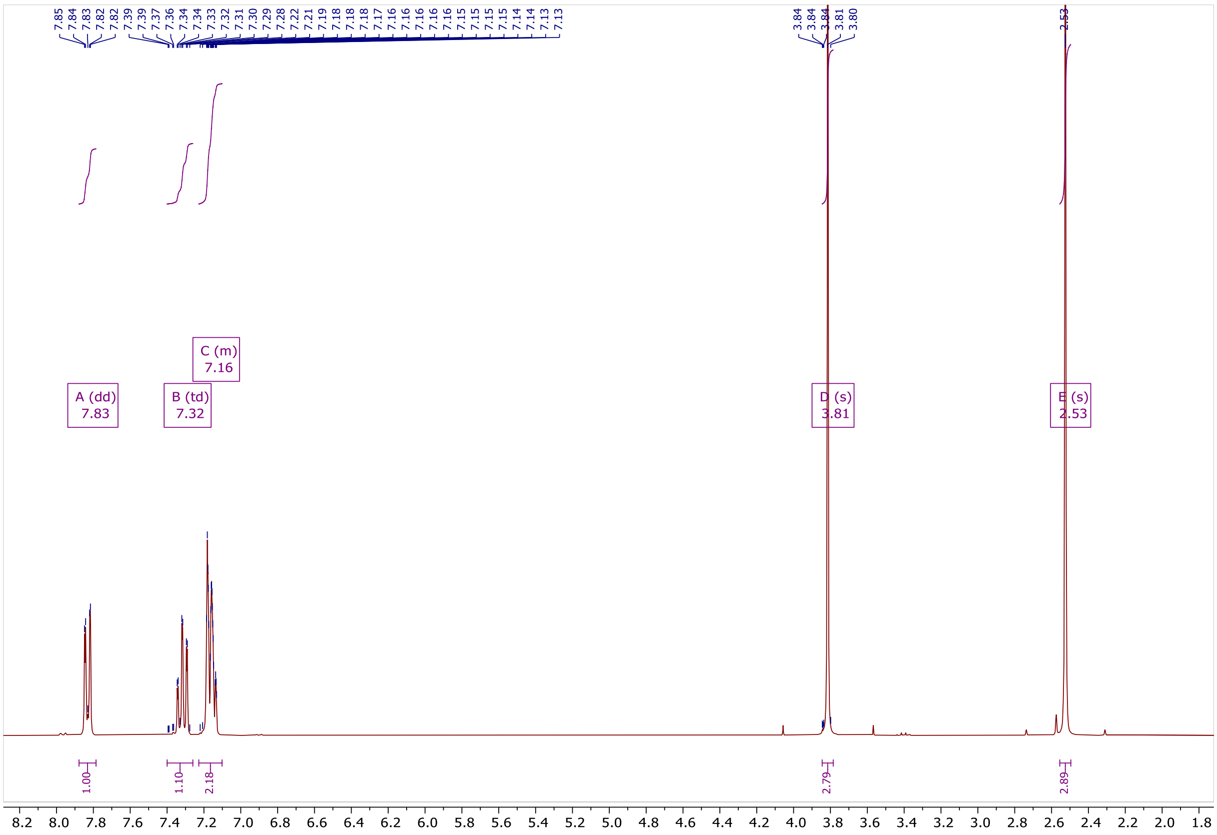

Figure S69: ^{1}H NMR spectrum of methyl 2-methylbenzoate in $CDCl_3$.

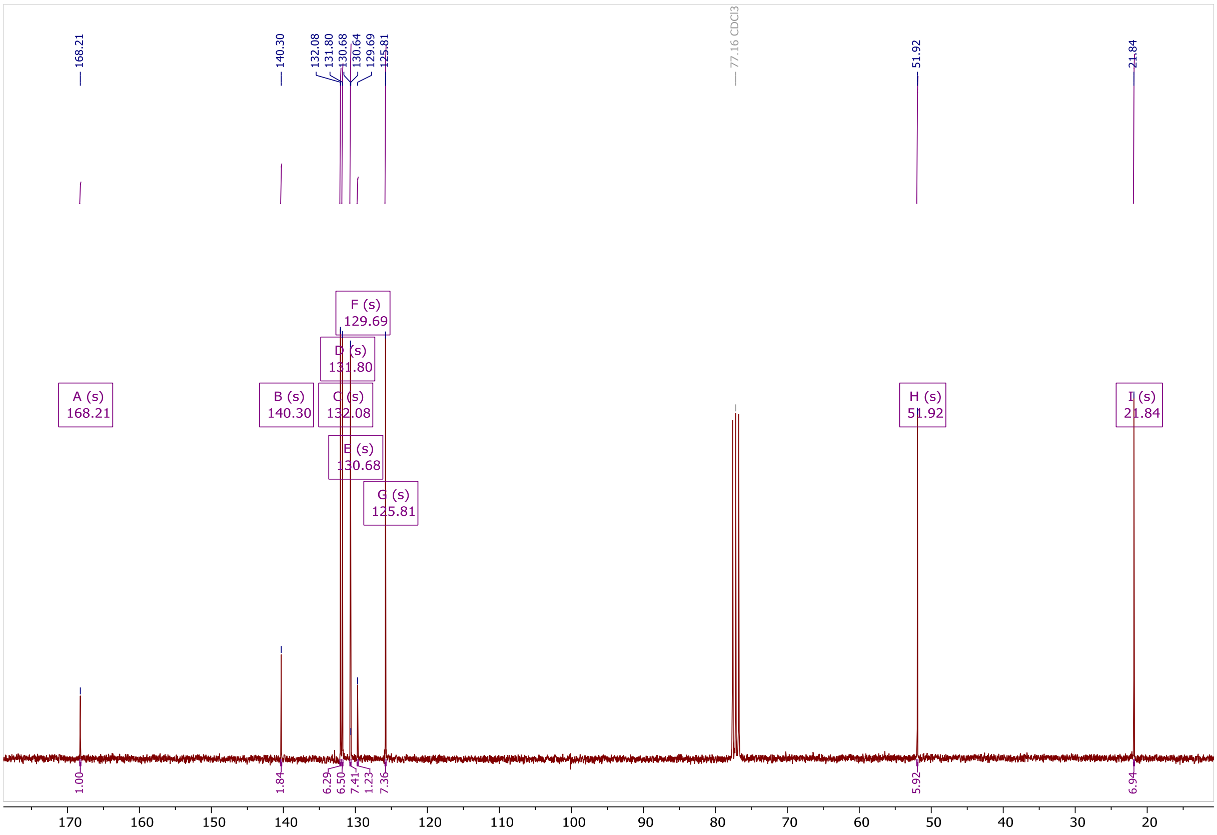

Figure S70: ^{13}C NMR spectrum of methyl 2-methylbenzoate in $CDCl_3$.

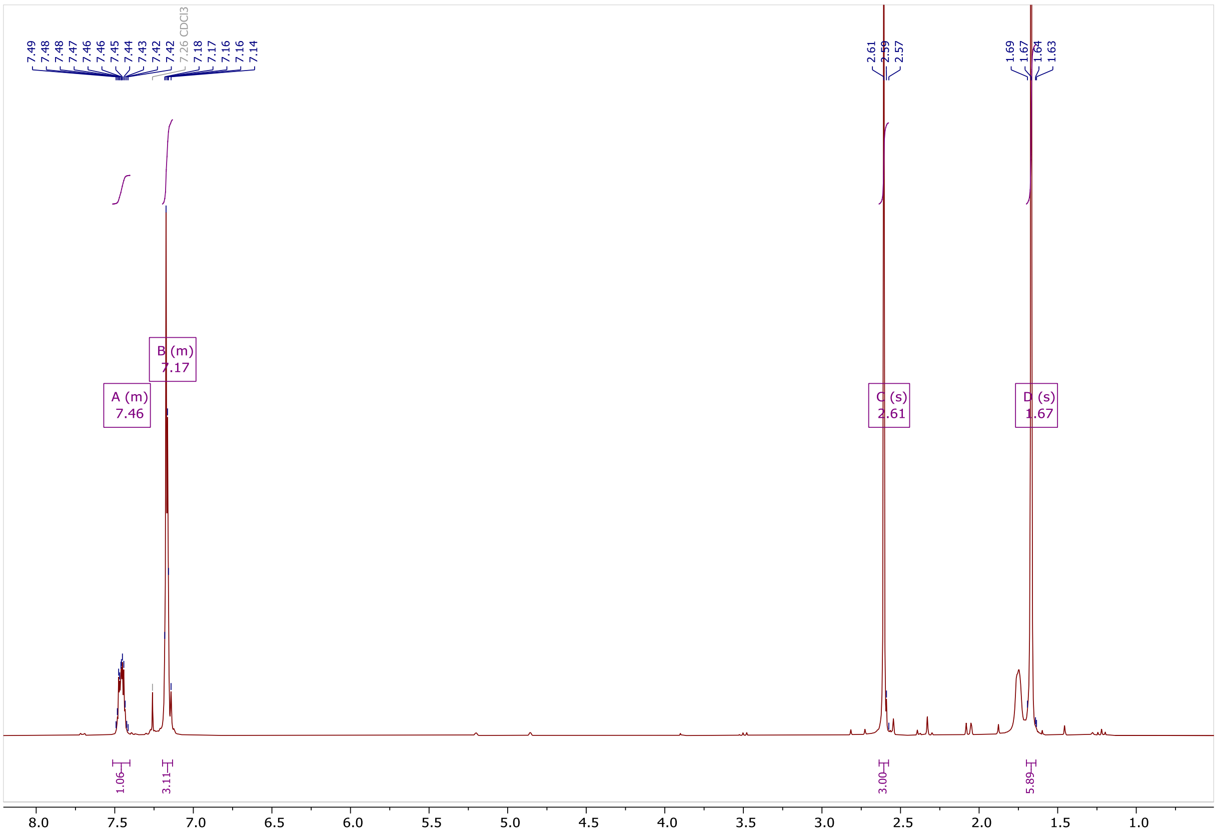

Figure S71: ^{1}H NMR spectrum of 2-(o-tolyl)propan-2-ol in $CDCl_3$.

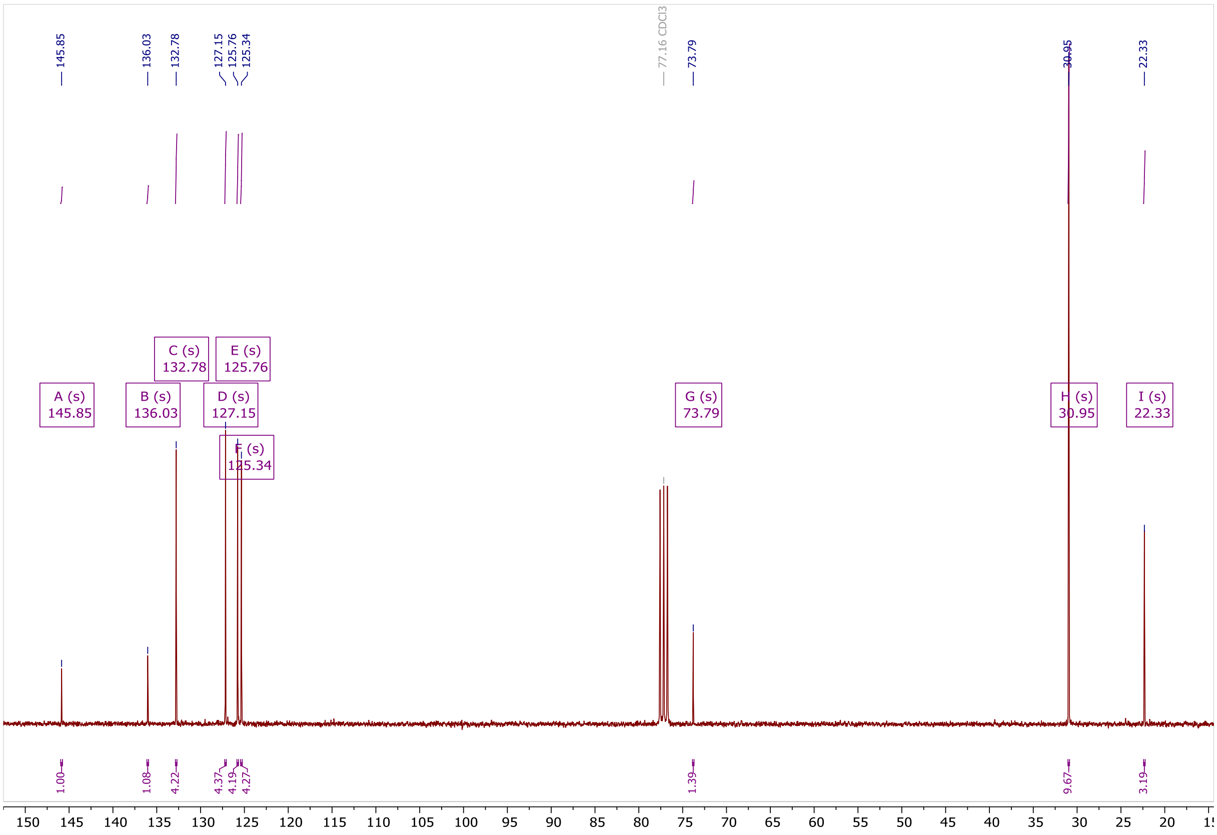

Figure S72: ^{13}C NMR spectrum of 2-(o-tolyl)propan-2-ol in $CDCl_3$.

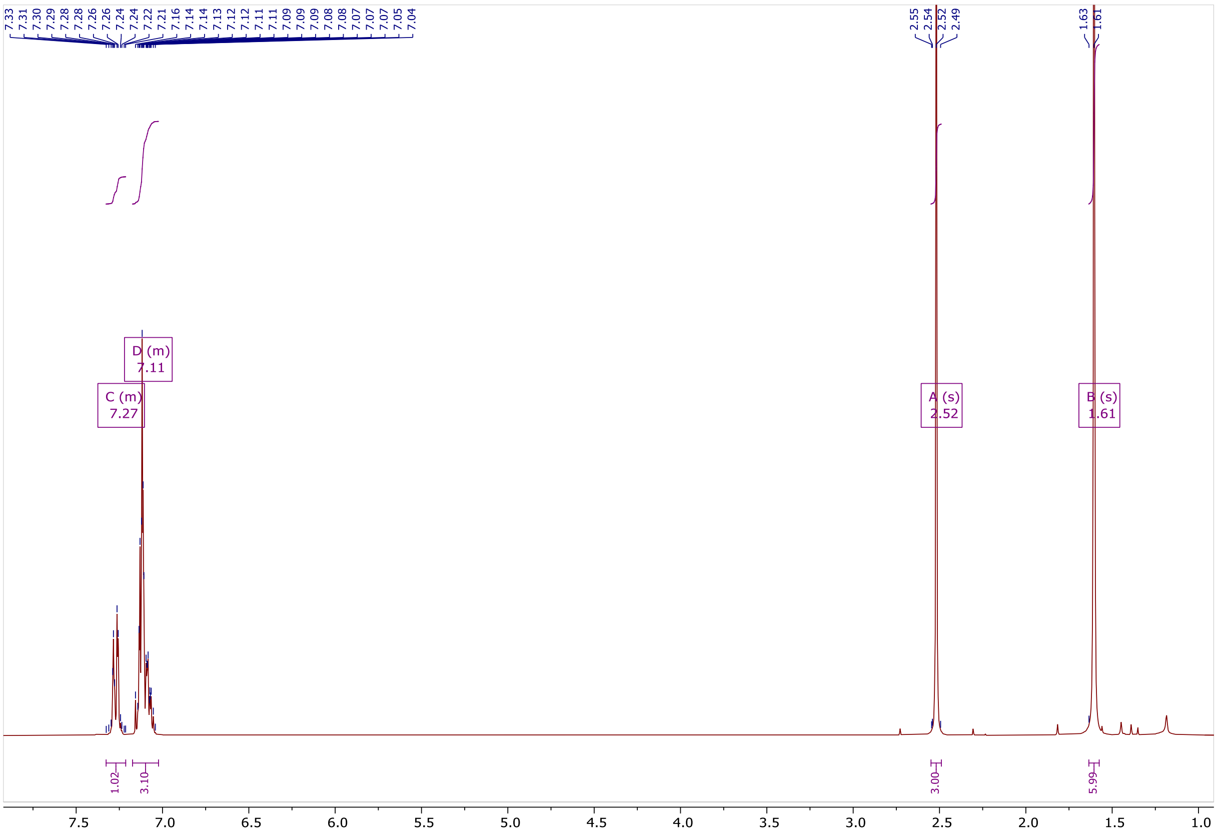

Figure S73: ^{1}H NMR spectrum of substrate **9a** in $CDCl_3$.

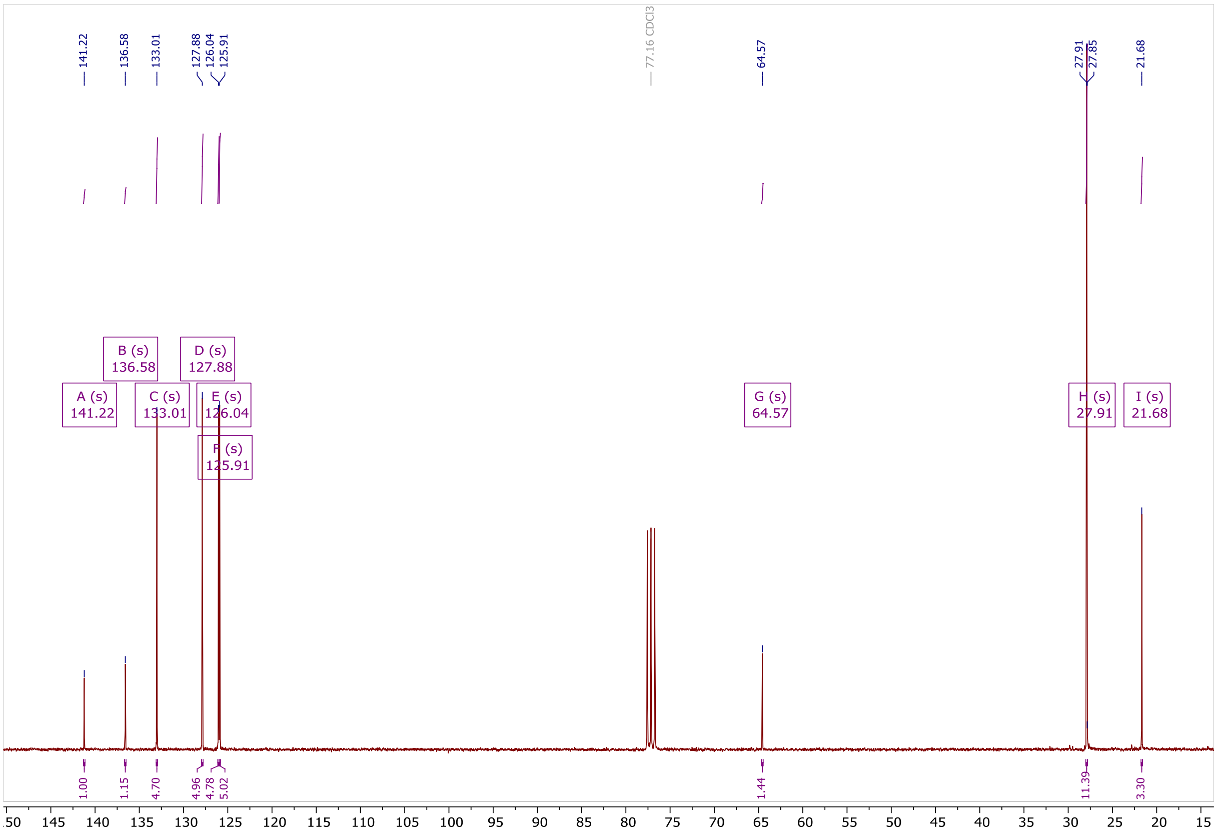

Figure S74: ^{13}C NMR spectrum of substrate **9a** in $CDCl_3$.

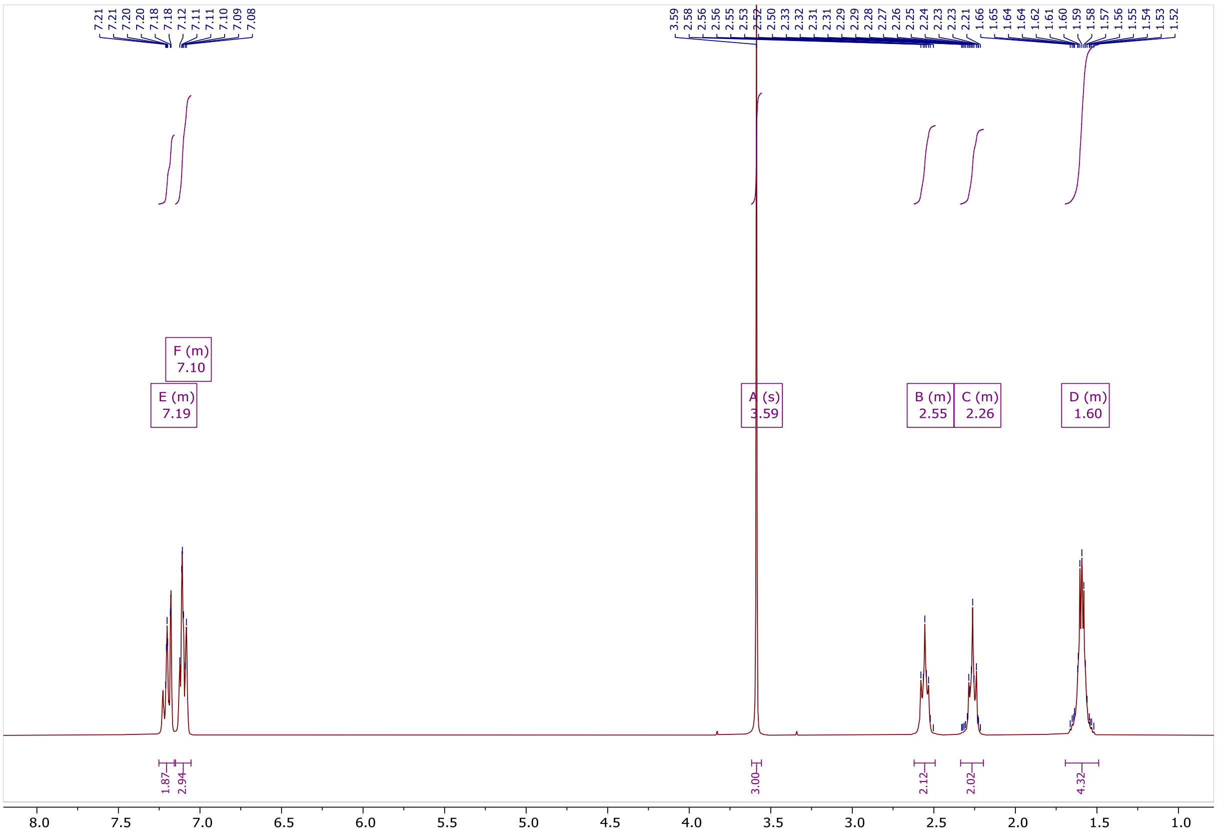

Figure S75: ^{1}H NMR spectrum of methyl 5-phenylpentanoate in $CDCl_3$.

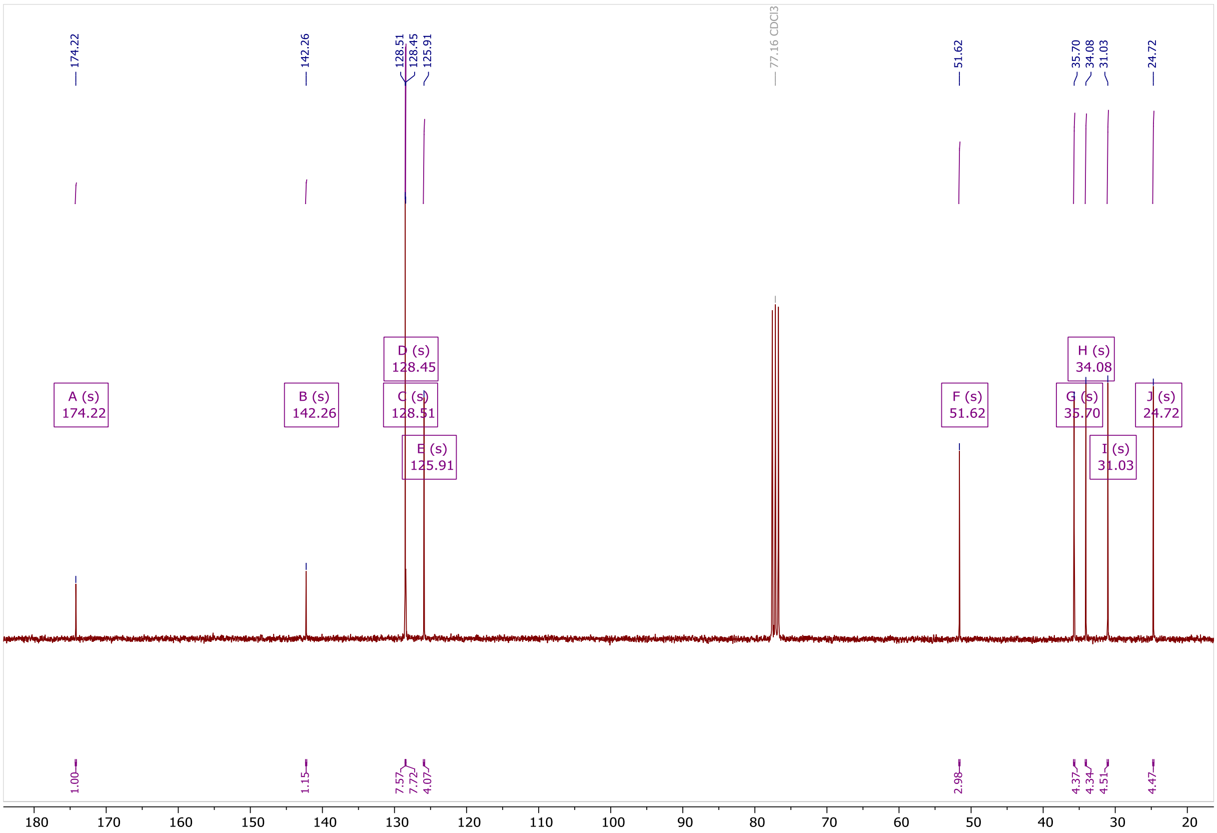

Figure S76: ^{13}C NMR spectrum of methyl 5-phenylpentanoate in $CDCl_3$.

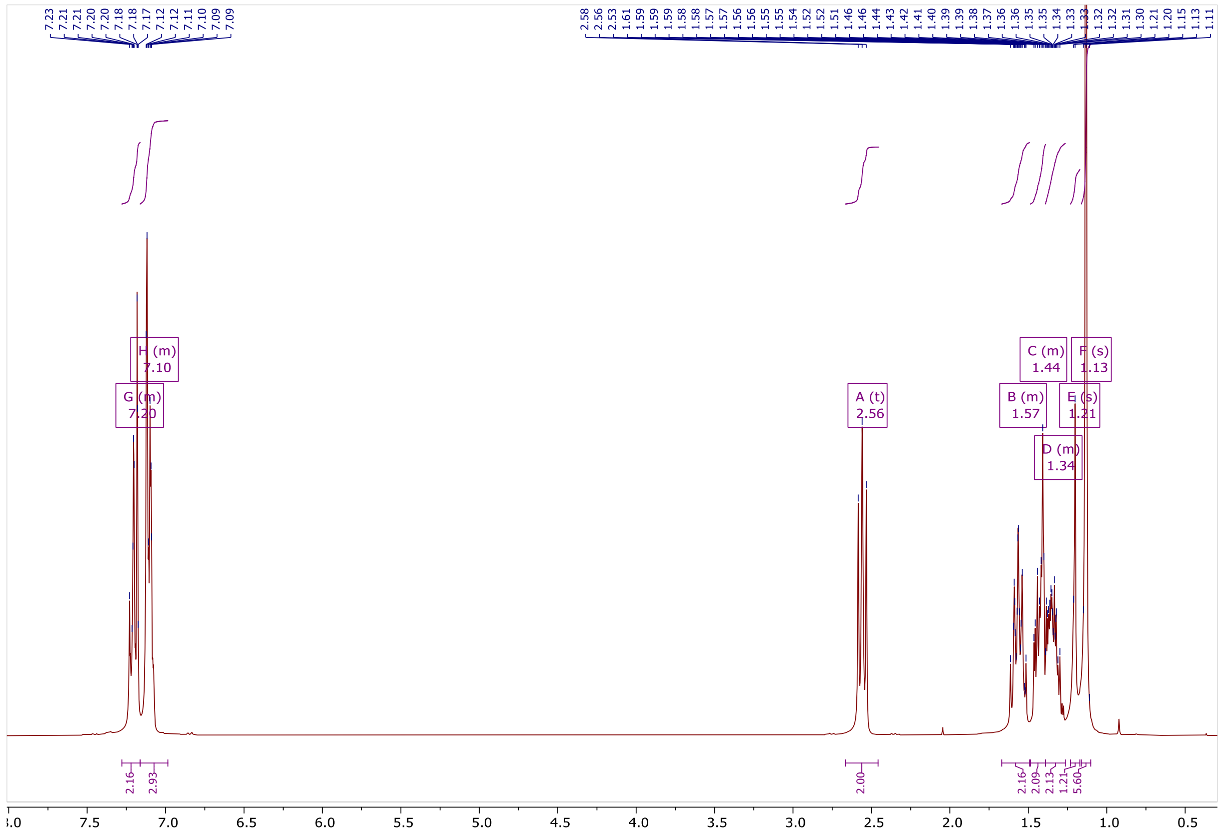

Figure S77: ^{1}H NMR spectrum of 2-methyl-6-phenylhexan-2-ol in $CDCl_3$.

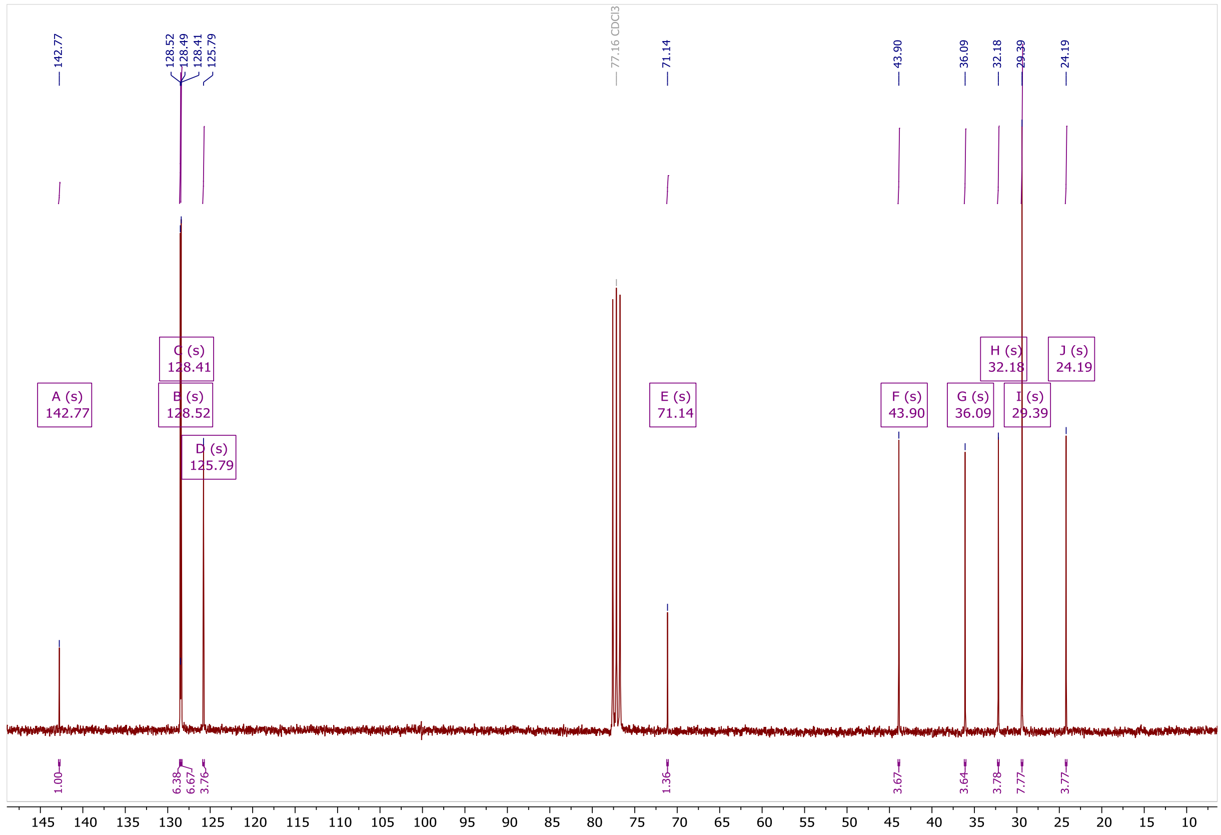

Figure S78: ^{13}C NMR spectrum of 2-methyl-6-phenylhexan-2-ol in $CDCl_3$.

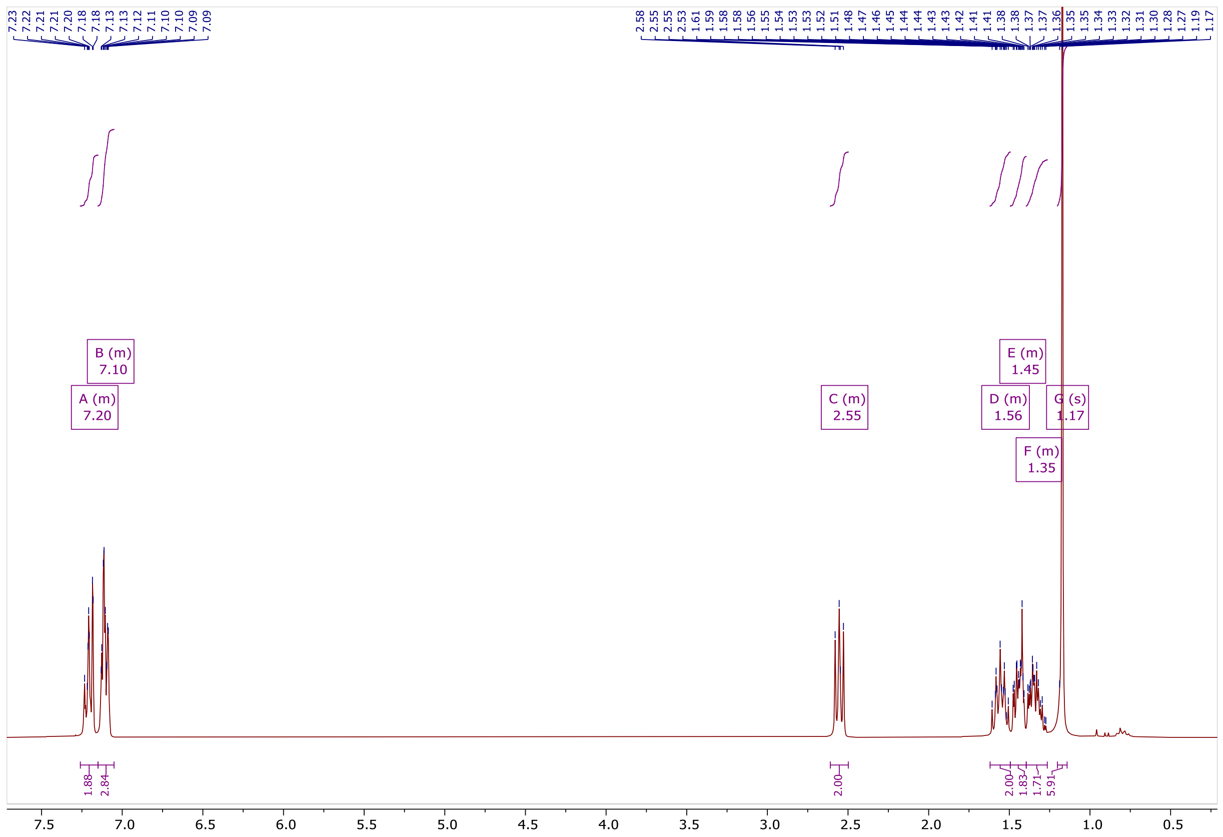

Figure S79: ^{1}H NMR spectrum of substrate **10a** in $CDCl_3$.

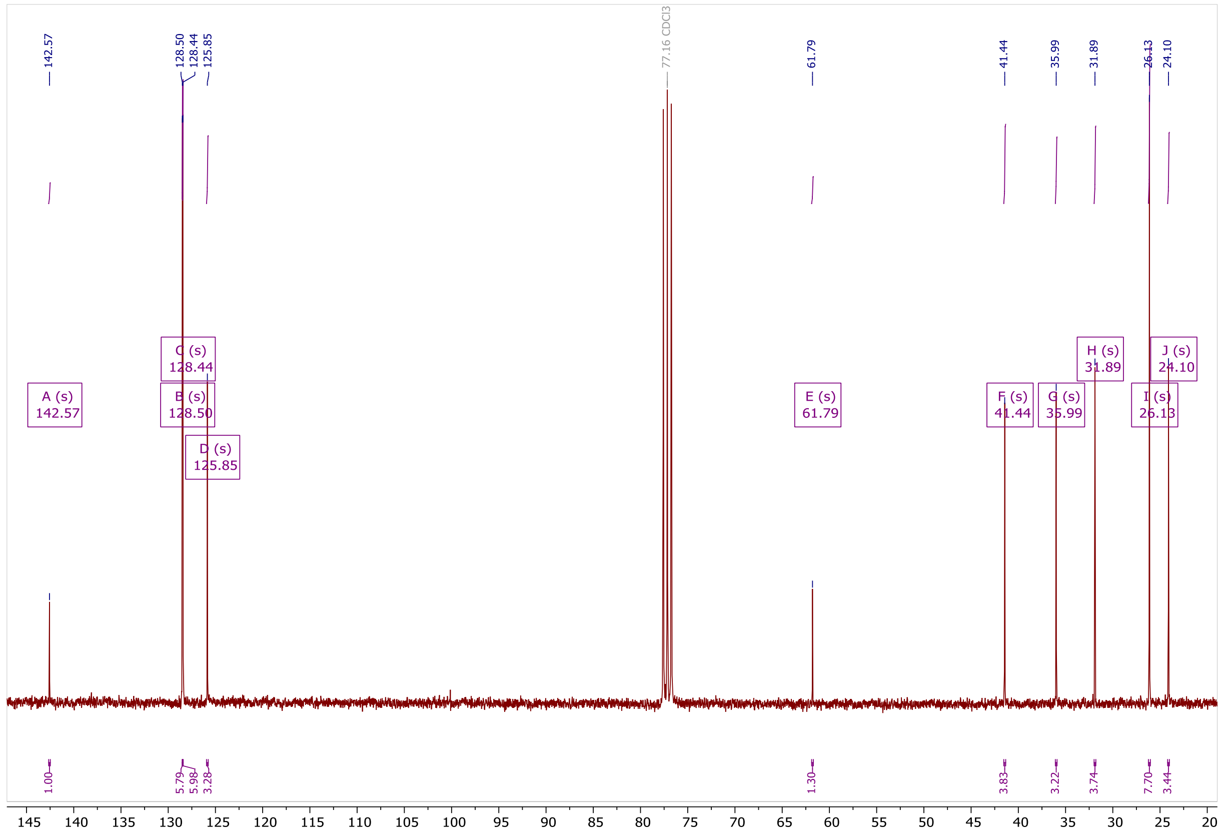

Figure S80: ^{13}C NMR spectrum of **10a** in $CDCl_3$.

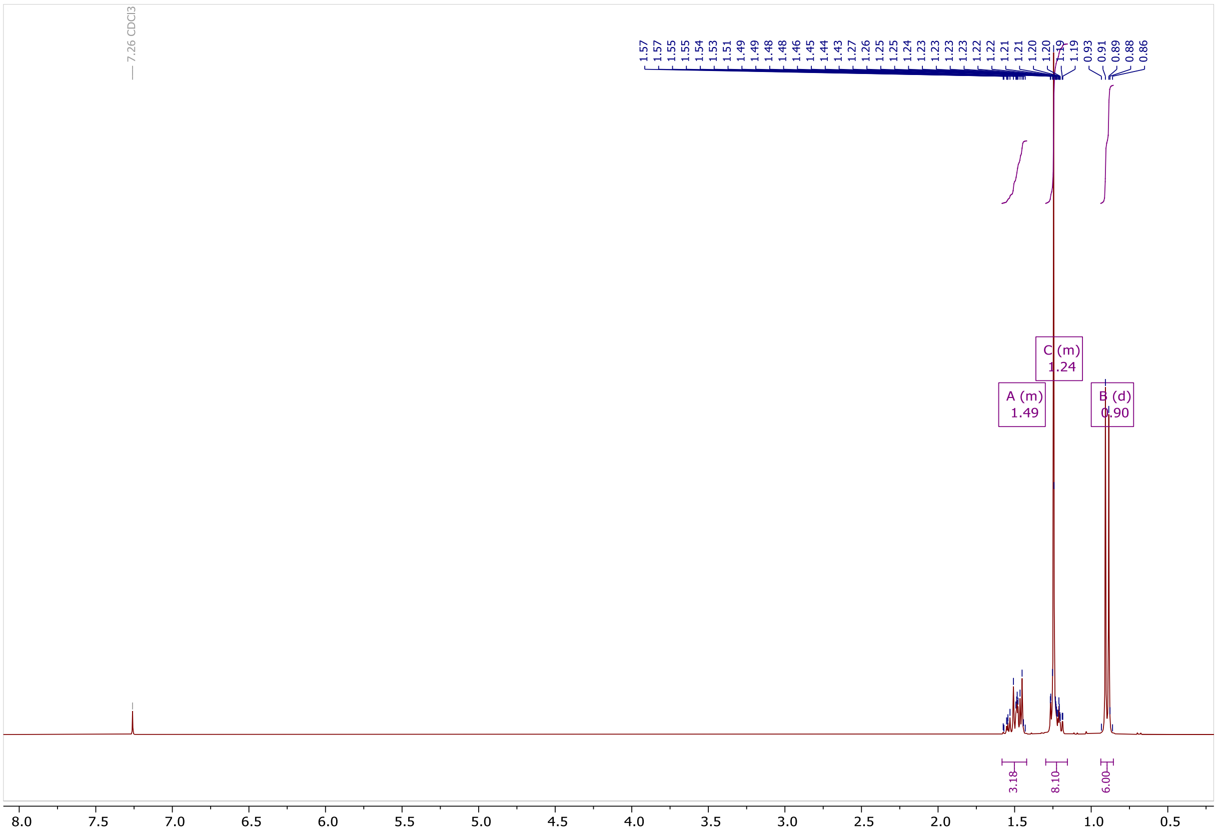

Figure S81: ^{1}H NMR spectrum of substrate **11a** in $CDCl_3$.

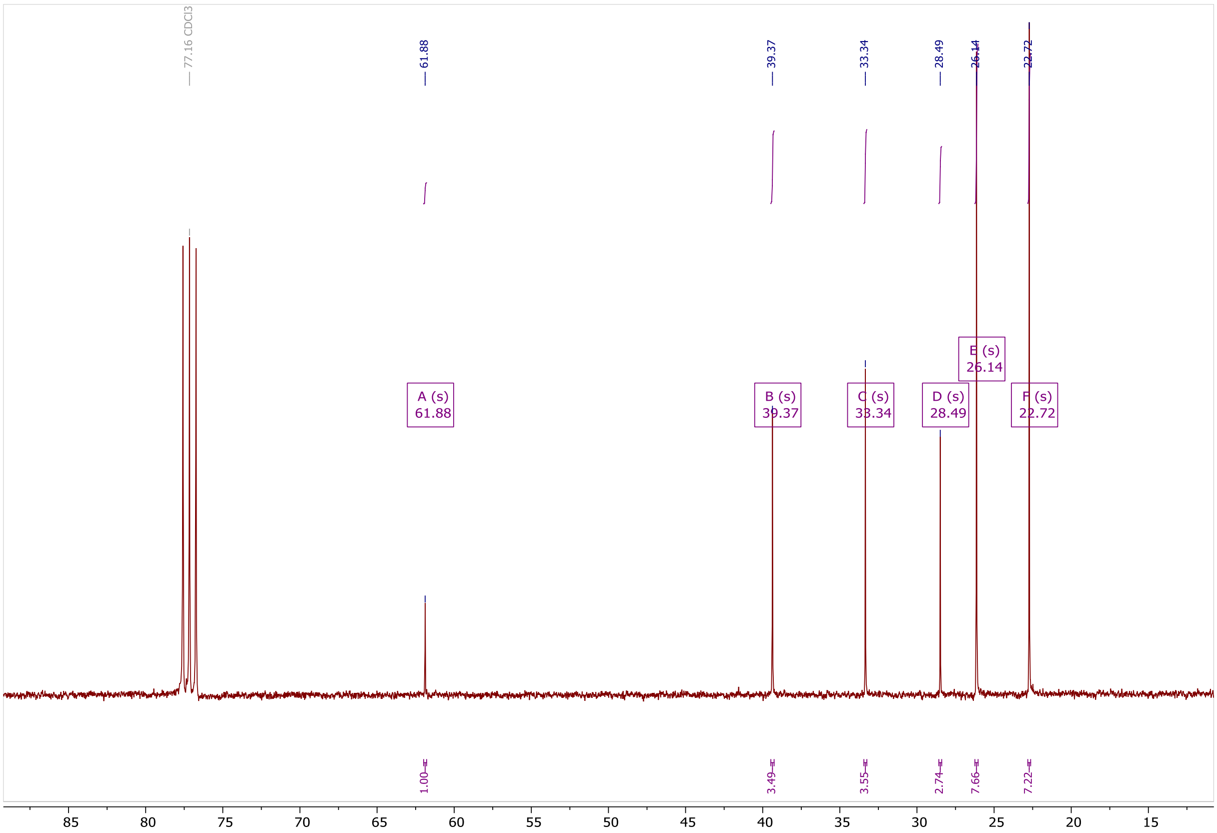

Figure S82: ^{13}C NMR spectrum of substrate **11a** in $CDCl_3$.

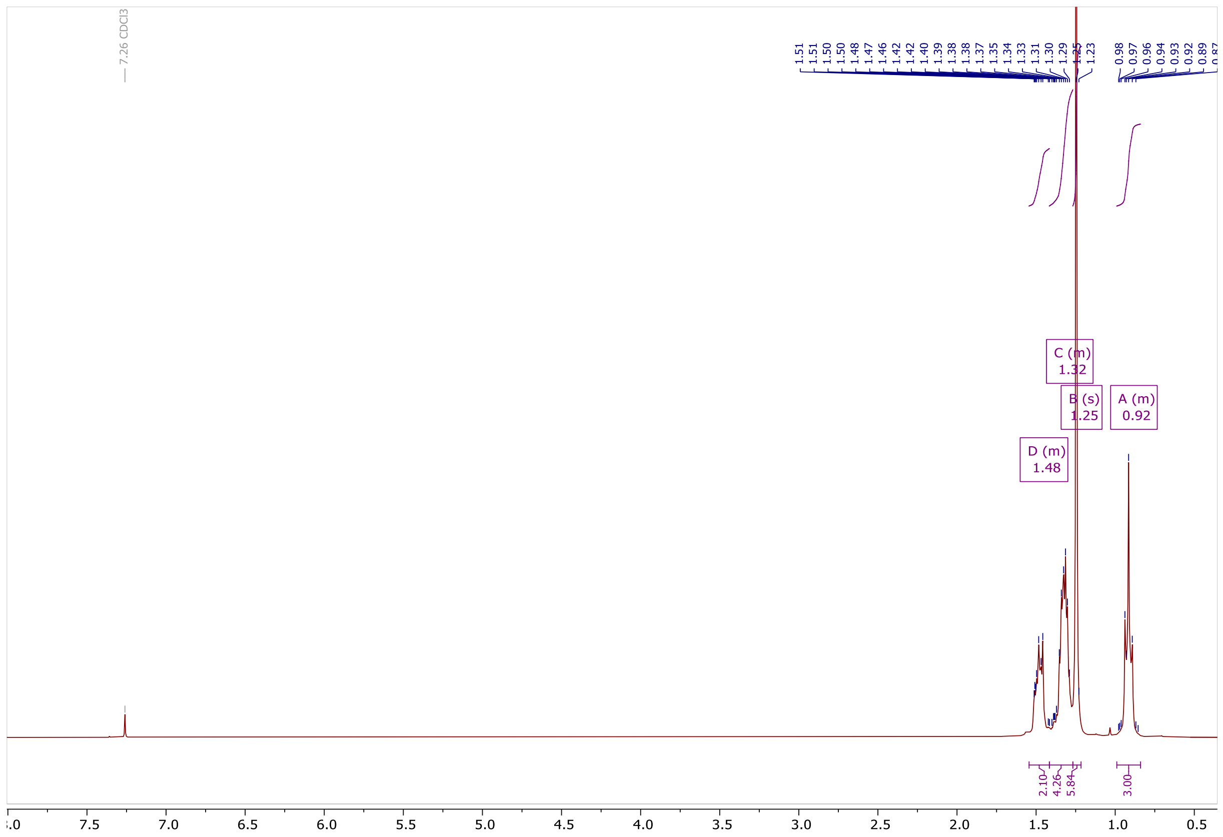

Figure S83: ^{1}H NMR spectrum of substrate **12a** in $CDCl_3$.

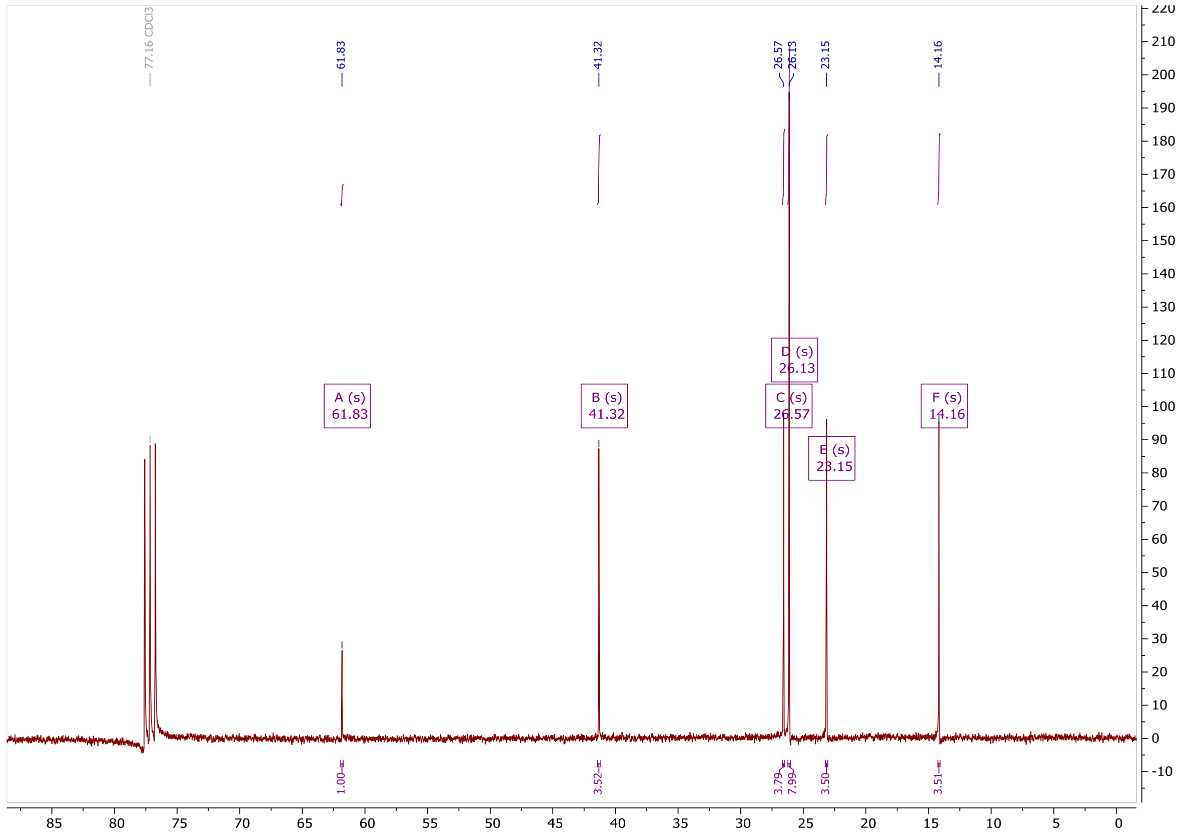

Figure S84: ^{13}C NMR spectrum of substrate **12a** in $CDCl_3$.

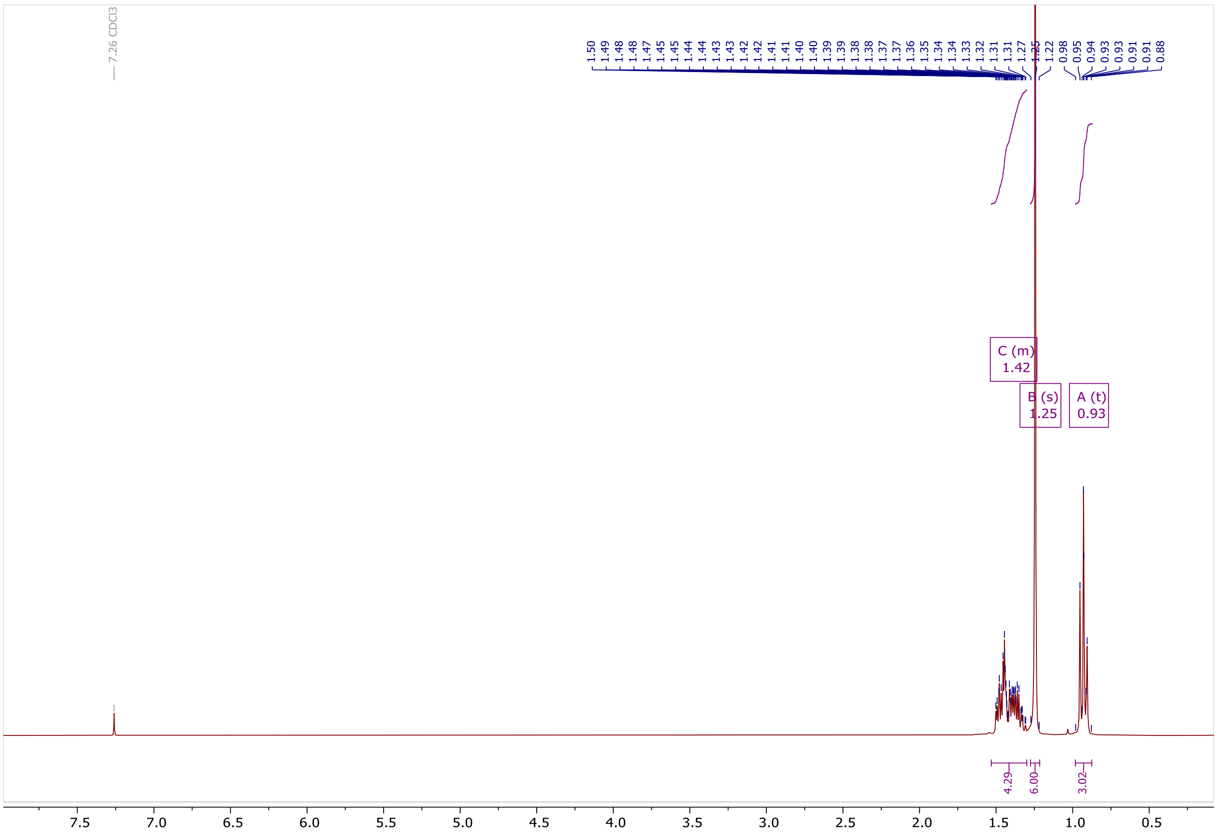

Figure S85: ^{1}H NMR spectrum of substrate **13a** in $CDCl_3$.

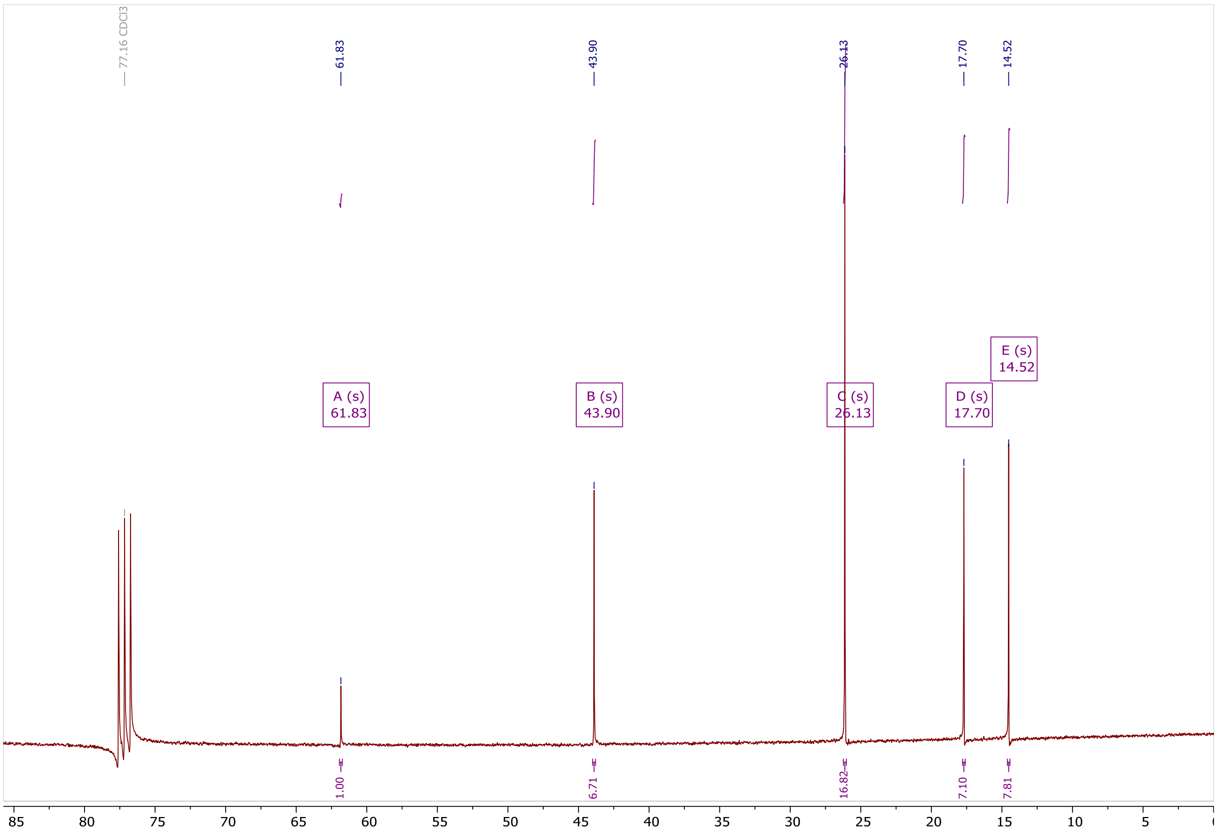

Figure S86: ^{13}C NMR spectrum of substrate **13a** in $CDCl_3$.

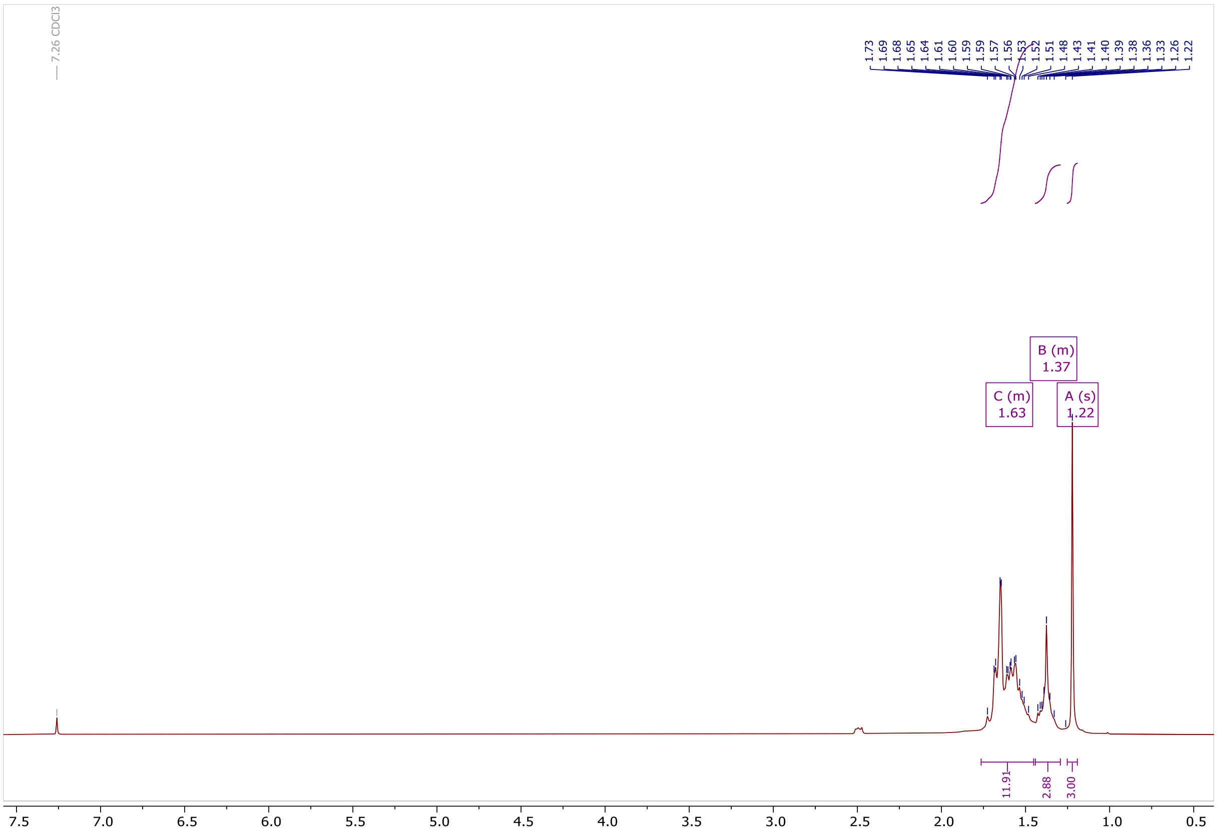

Figure S87: ^{1}H NMR spectrum of 1-methylcycloheptan-1-ol in $CDCl_3$.

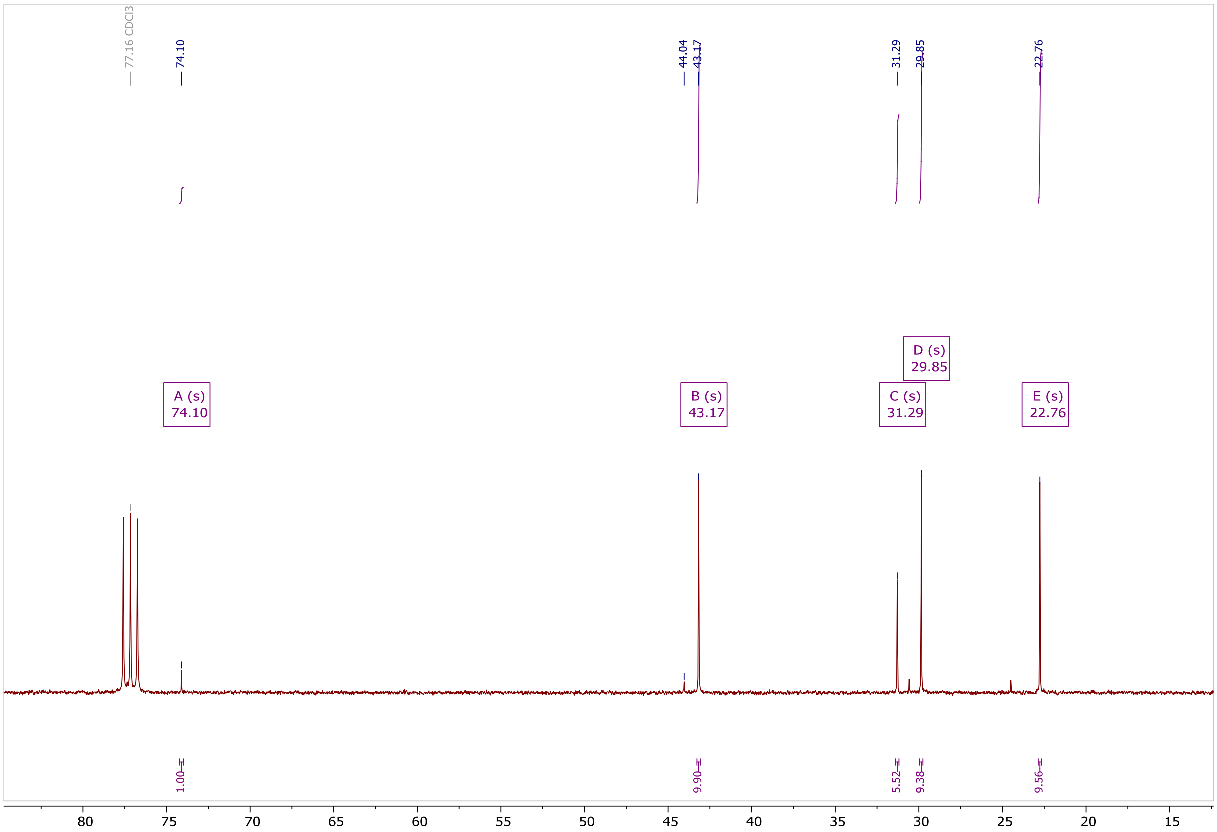

Figure S88: ^{13}C NMR spectrum of 1-methylcycloheptan-1-ol in $CDCl_3$.

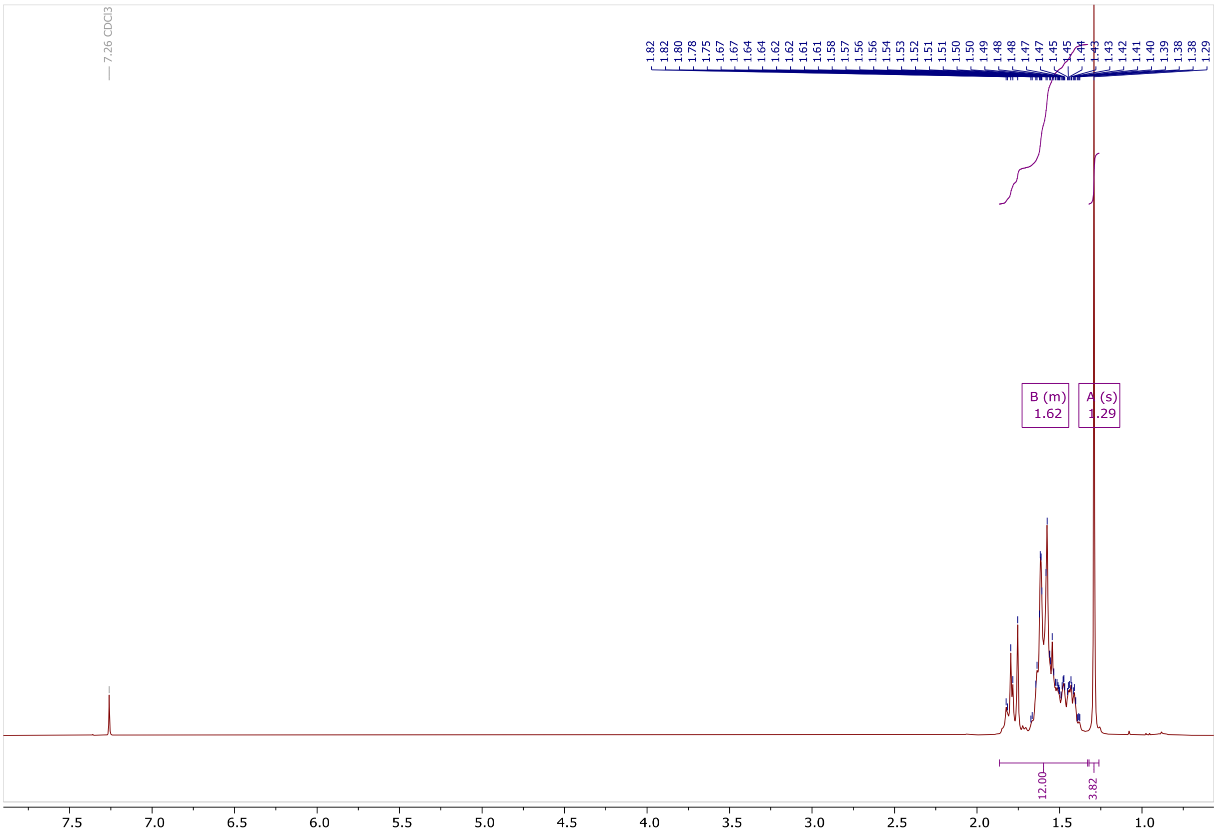

Figure S89: ^{1}H NMR spectrum of substrate **14a** in $CDCl_3$.

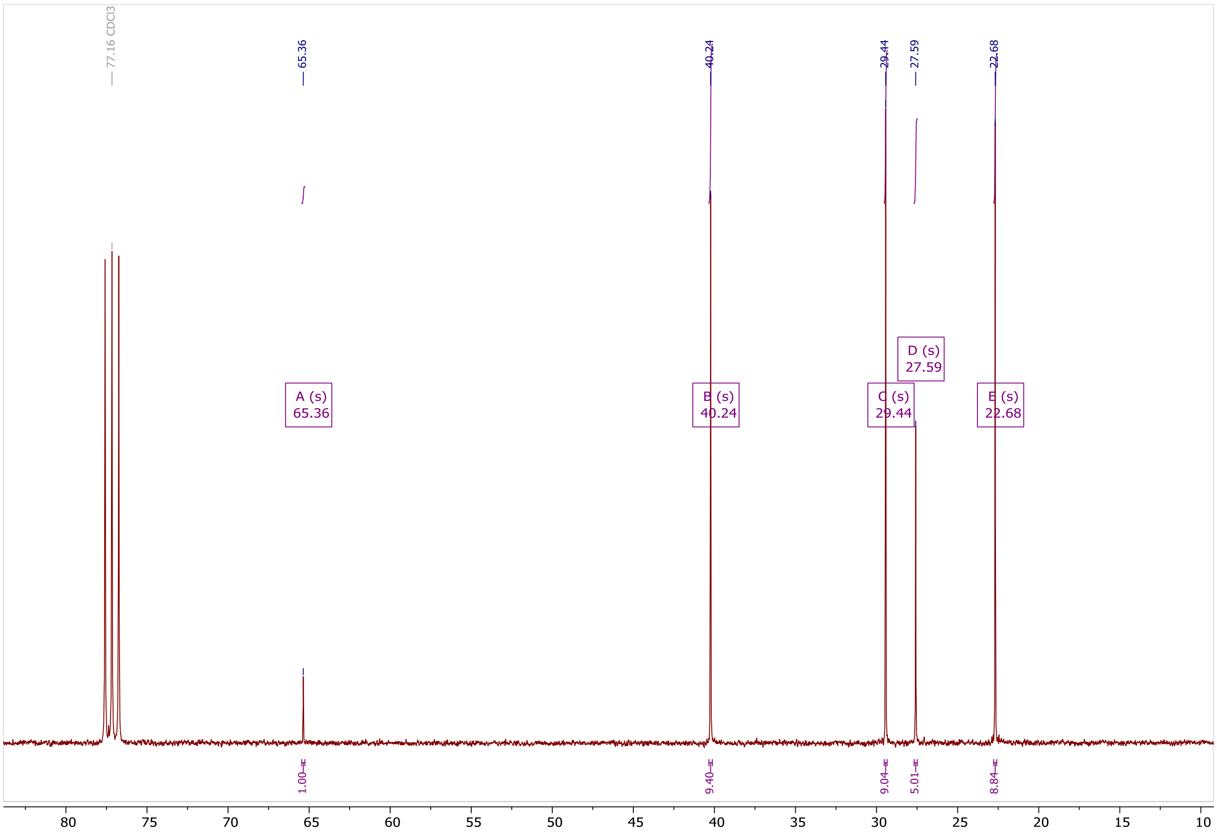

Figure S90: ^{13}C NMR spectrum of substrate **14a** in $CDCl_3$.

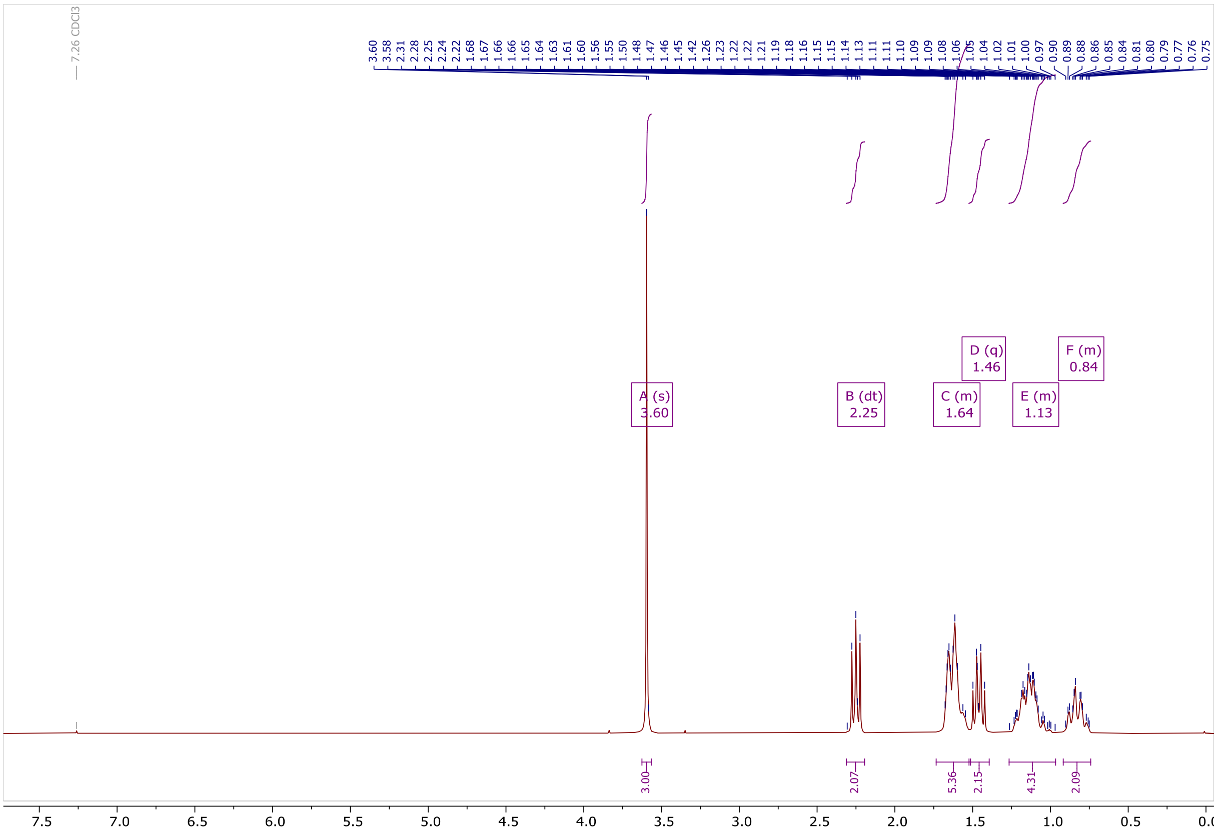

Figure S91: ^{1}H NMR spectrum of methyl 3-cyclohexylpropanoate in $CDCl_3$.

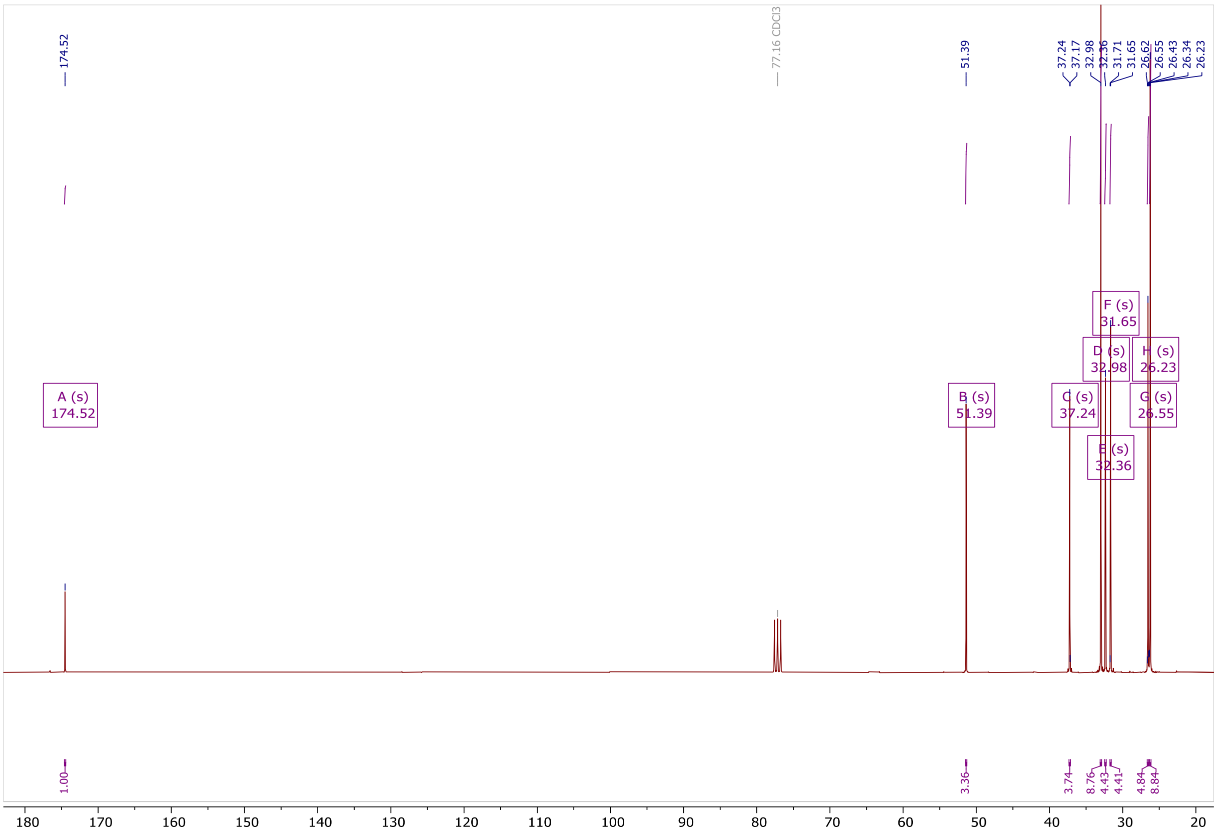

Figure S92: ^{13}C NMR spectrum of methyl 3-cyclohexylpropanoate in $CDCl_3$.

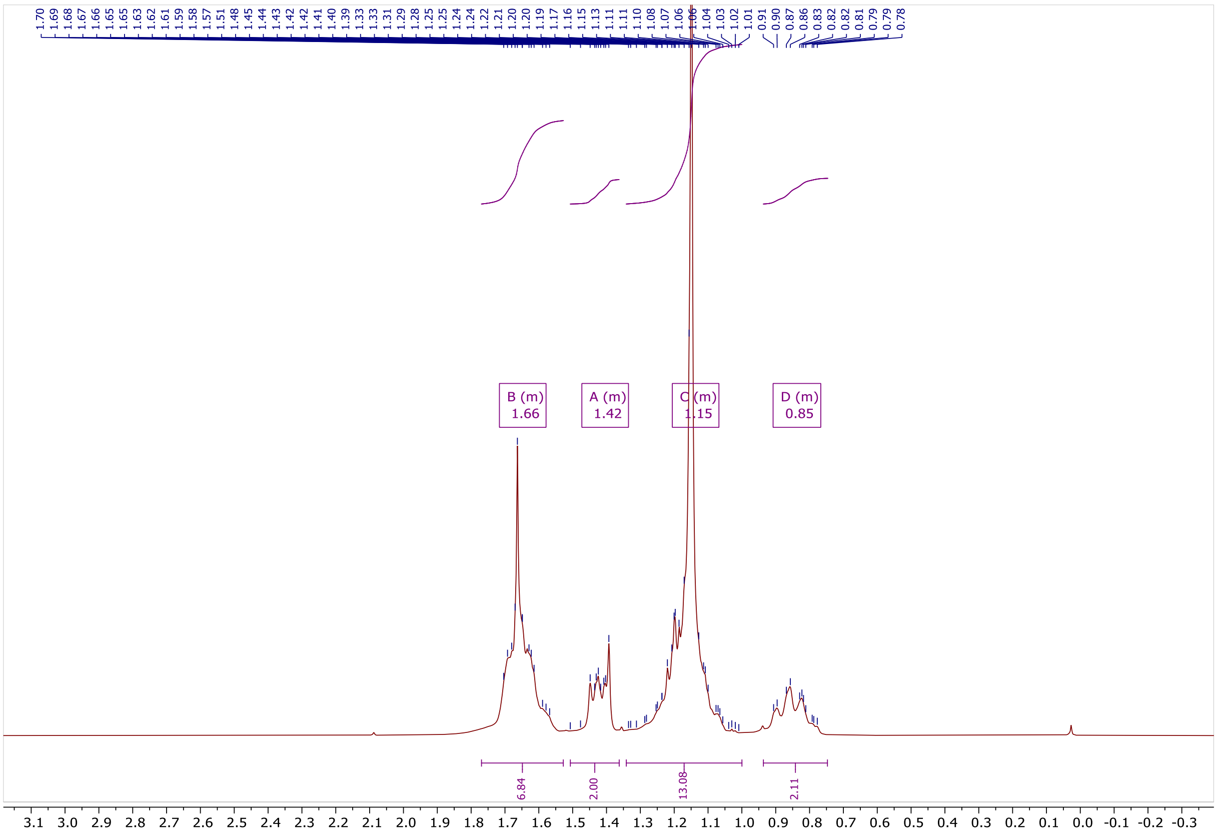

Figure S93: ^{1}H NMR spectrum of 4-cyclohexyl-2-methylbutan-2-ol in $CDCl_3$.

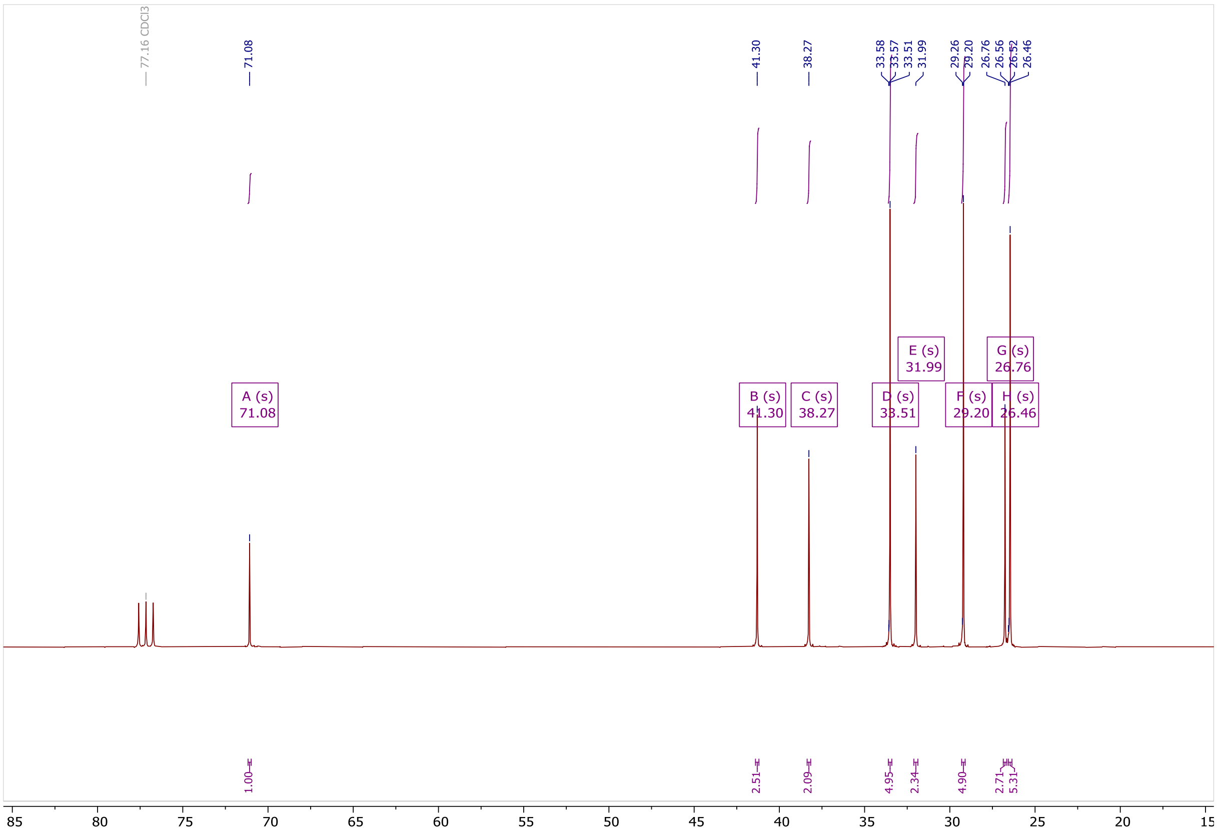

Figure S94: ^{13}C NMR spectrum of 4-cyclohexyl-2-methylbutan-2-ol in $CDCl_3$.

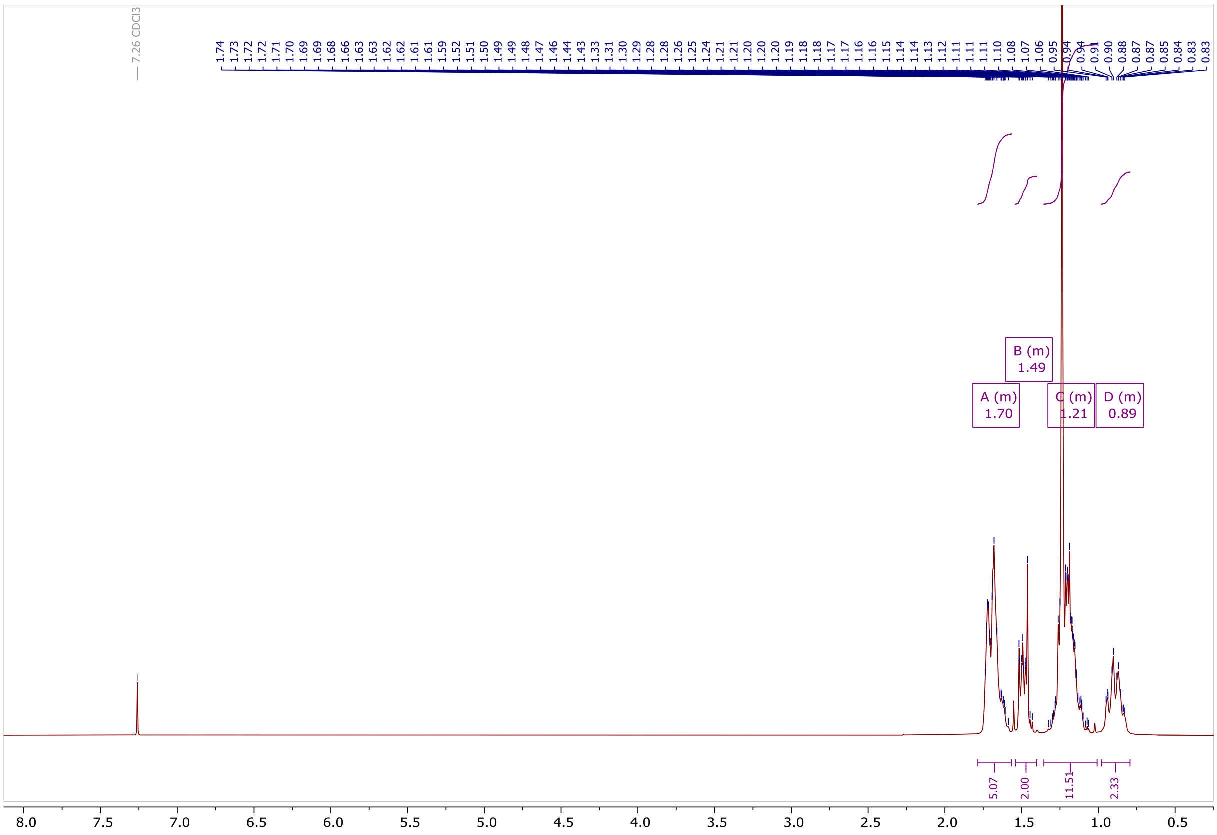

Figure S95: ^{1}H NMR spectrum of substrate **15a** in $CDCl_3$.

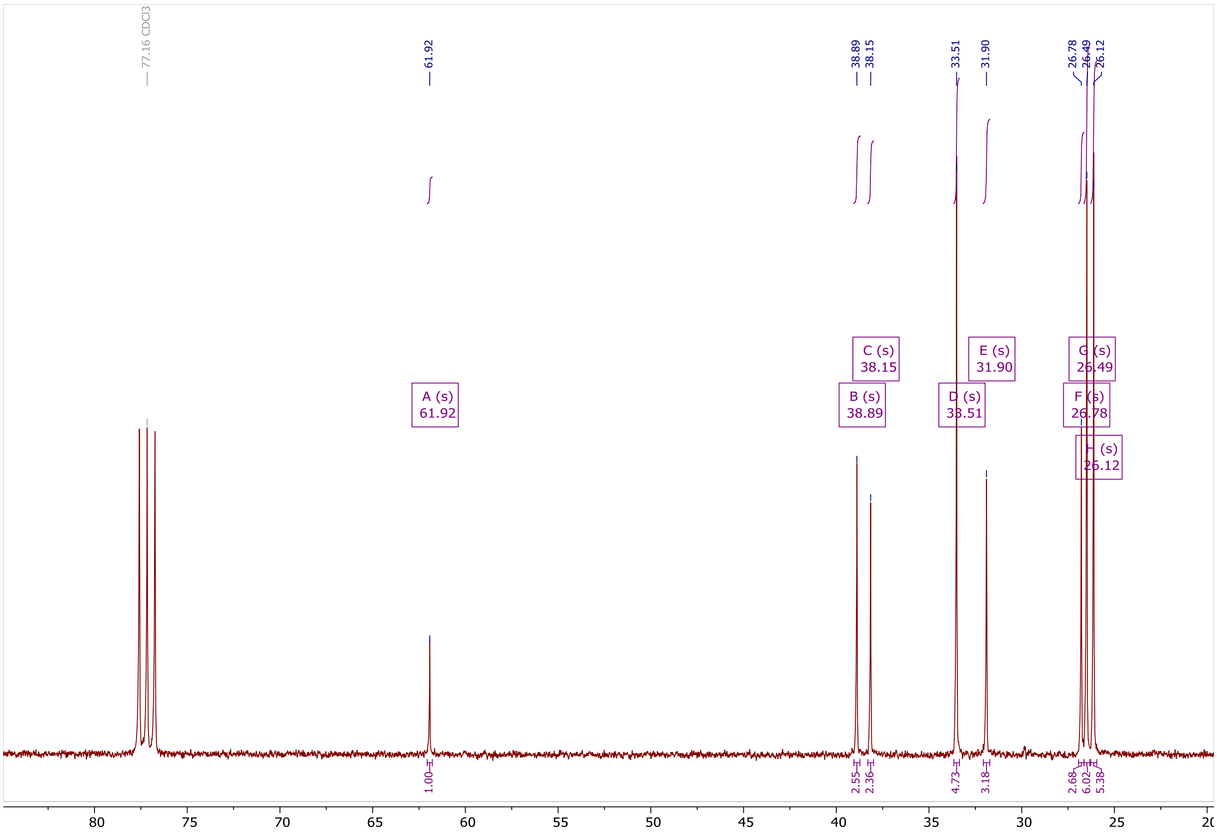

Figure S96: ^{13}C NMR spectrum of substrate **15a** in $CDCl_3$.

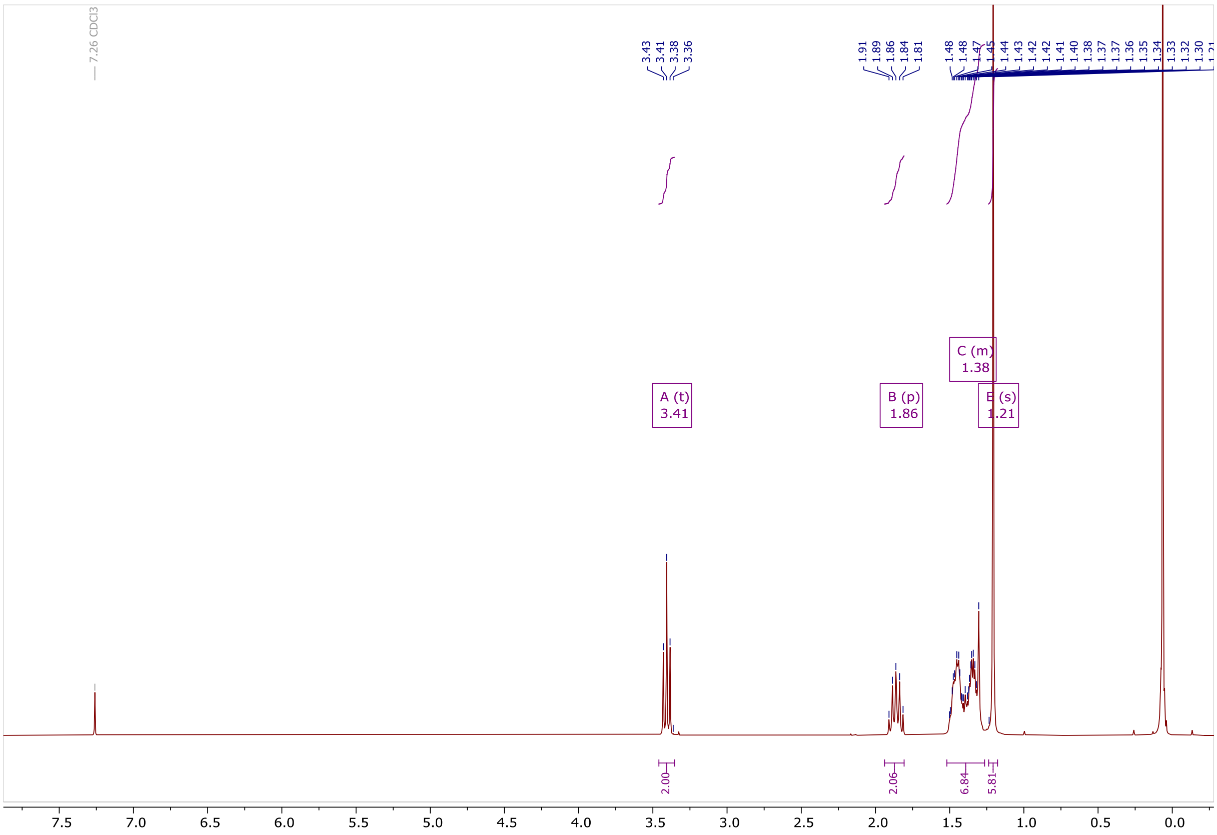

Figure S97: ^{1}H NMR spectrum of 8-bromo-2-methyloctan-2-ol in $CDCl_3$.

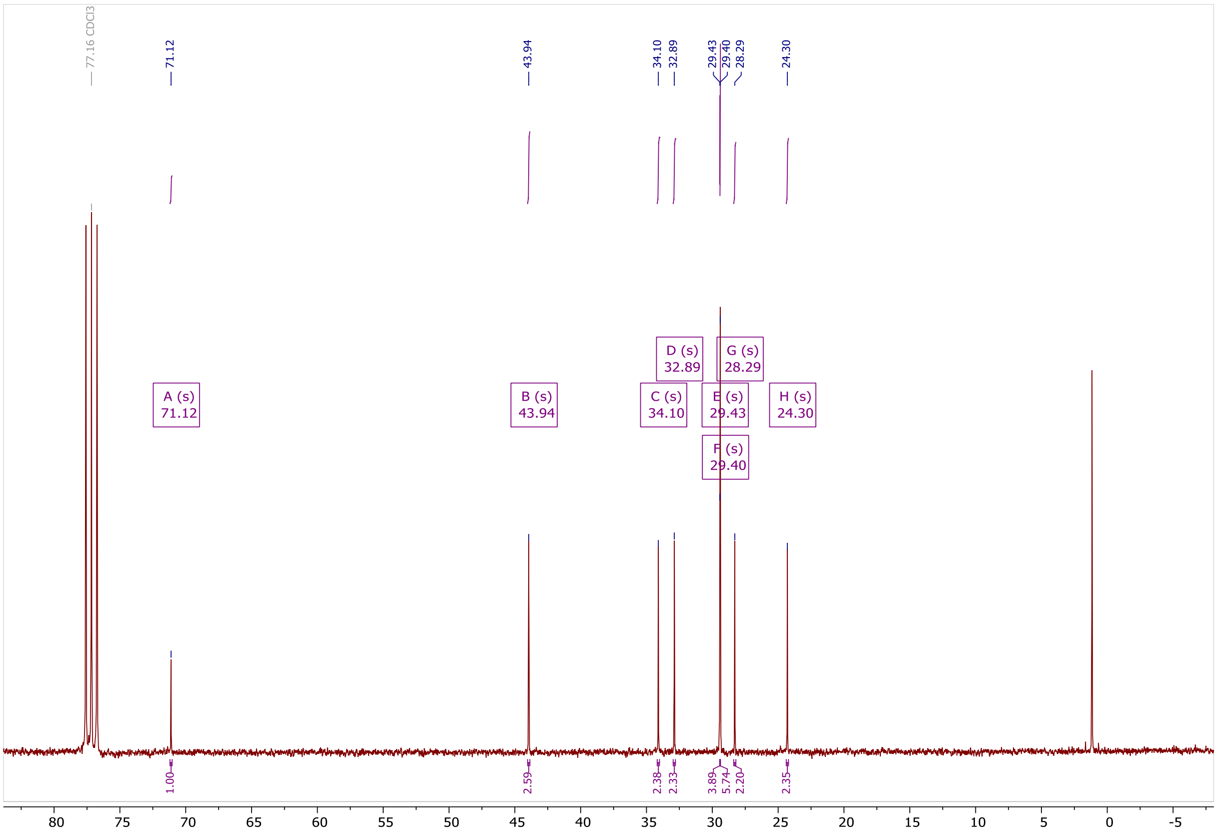

Figure S98: ^{13}C NMR spectrum of 8-bromo-2-methyloctan-2-ol in $CDCl_3$.

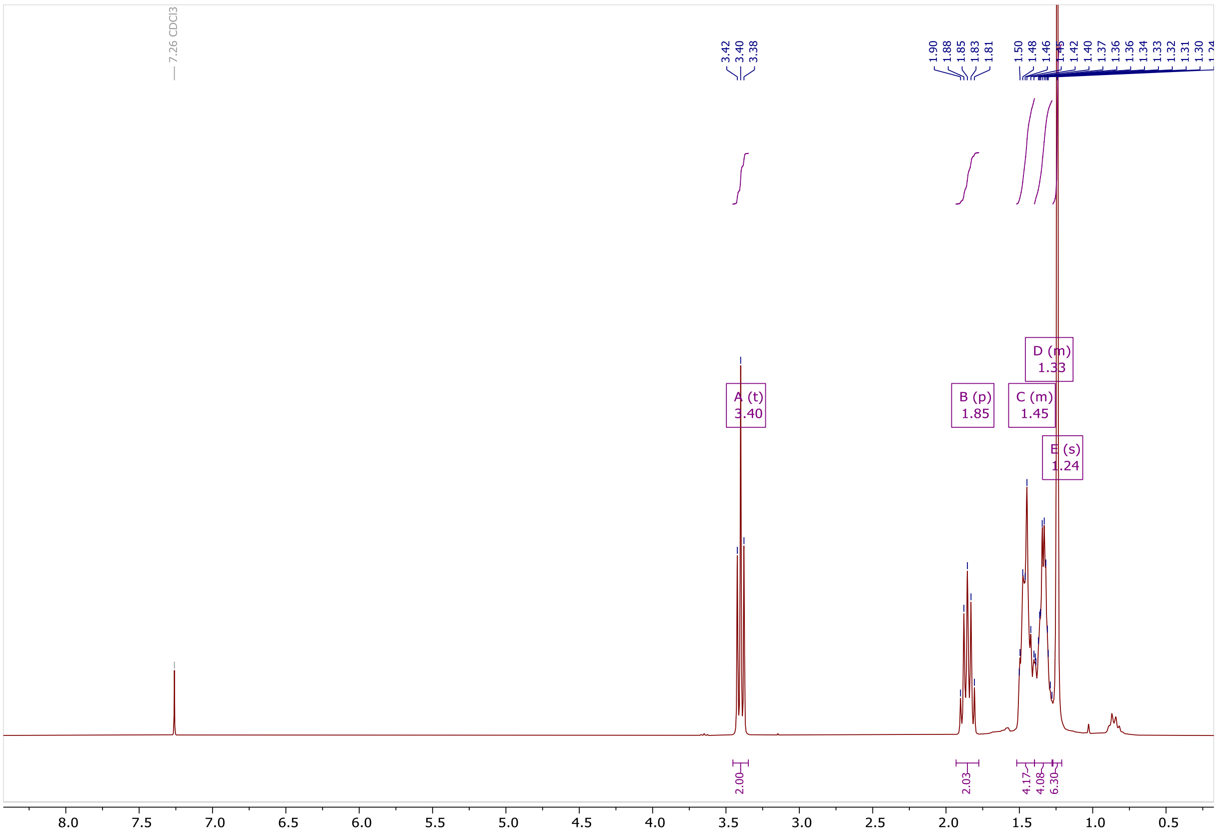

Figure S99: ^{13}C NMR spectrum of substrate **16a** in $CDCl_3$.

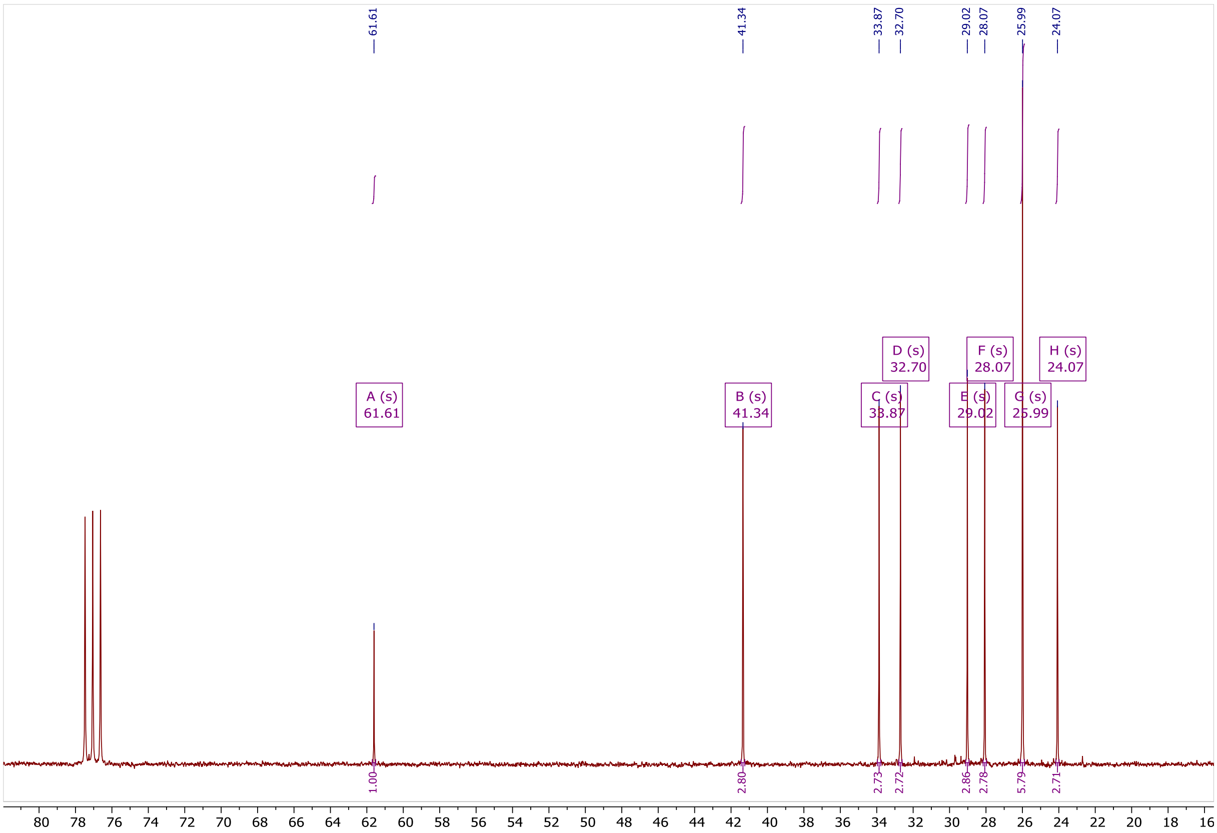

Figure S100: ^{13}C NMR spectrum of substrate **16a** in $CDCl_3$.

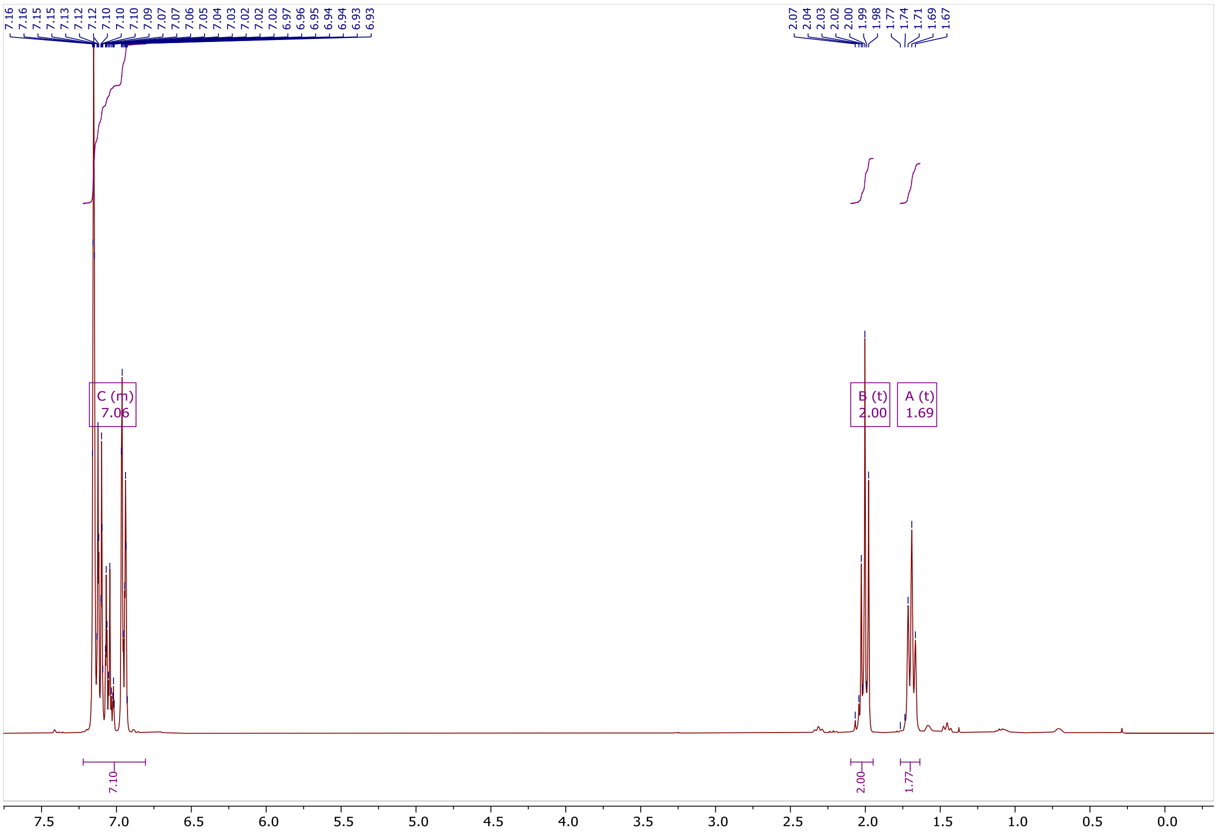

Figure S101: ^{1}H NMR spectrum of 4-phenylbutanoic-4,4-d_2 acid in C_6D_6.

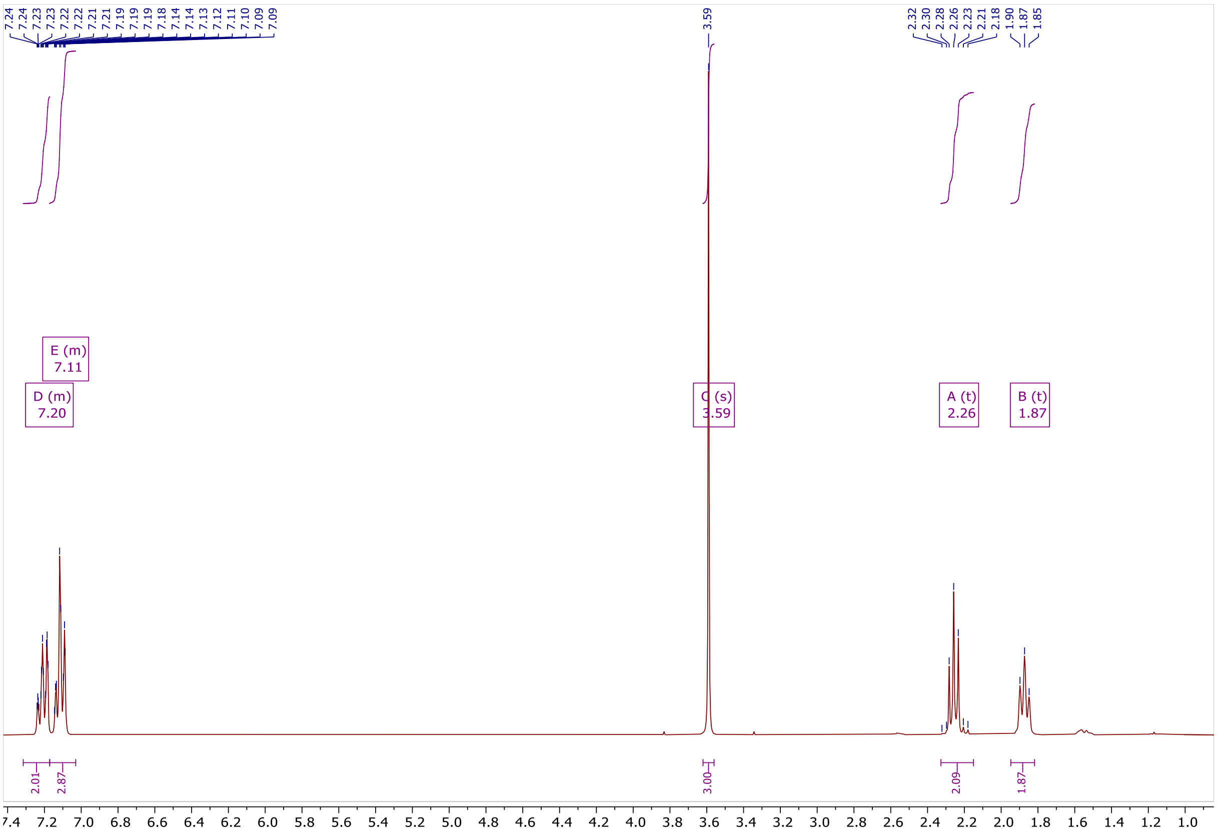

Figure S102: ^{1}H NMR spectrum of methyl 4-phenylbutanoate-4,4-d_2 in $CDCl_3$.

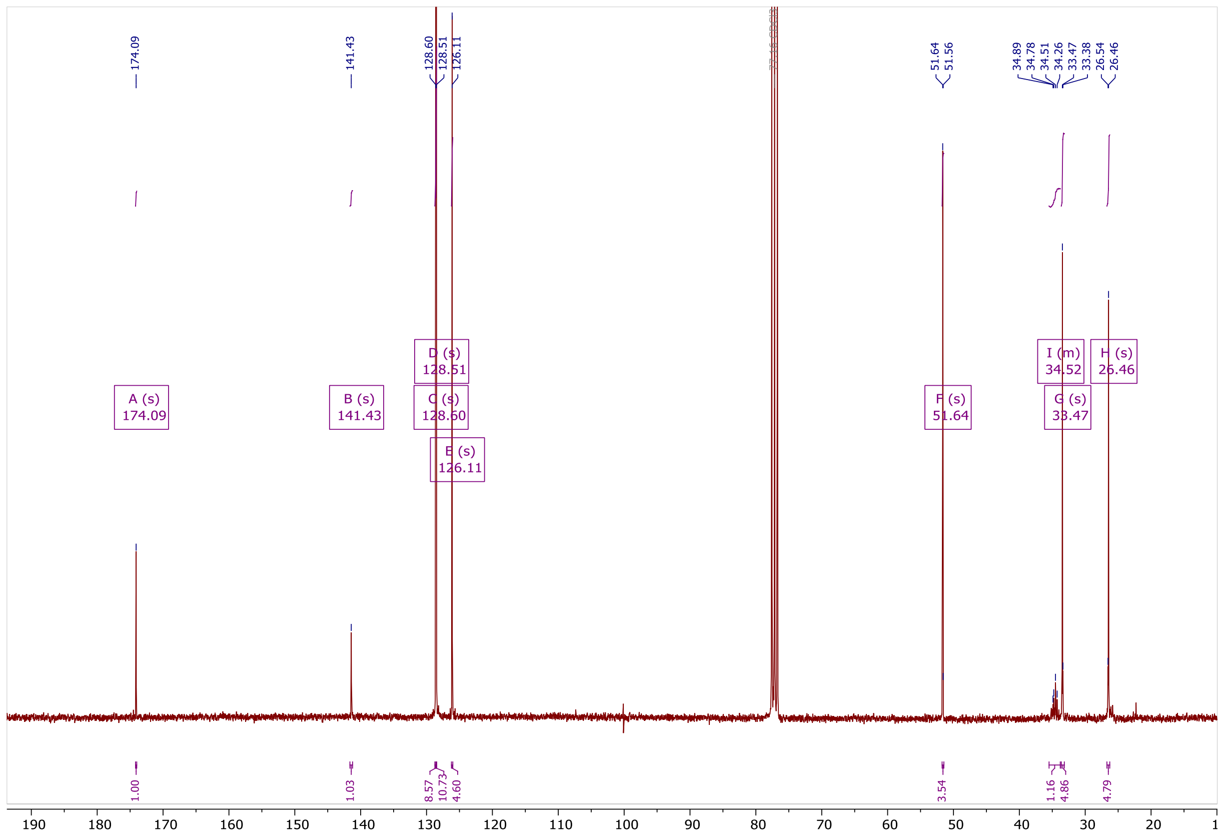

Figure S103: ^{13}C NMR spectrum of methyl 4-phenylbutanoate-4,4-d_2 in $CDCl_3$.

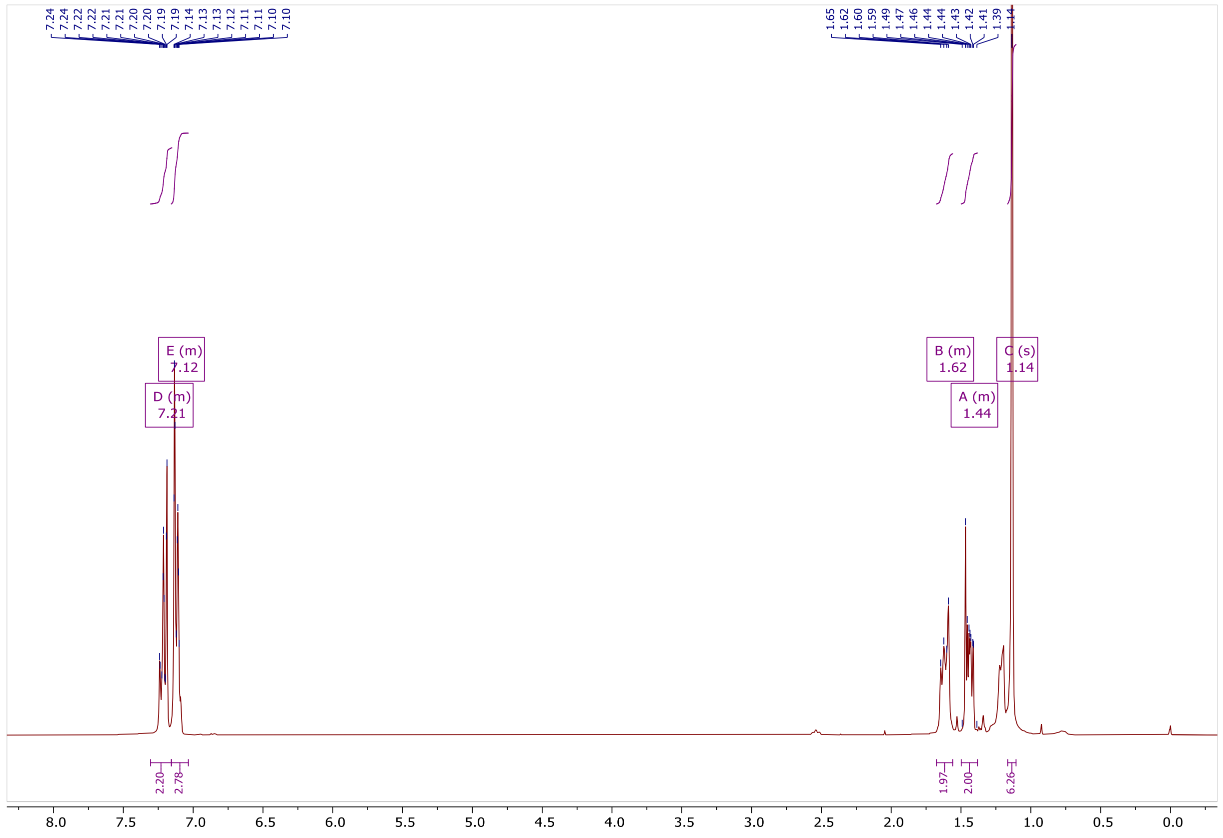

Figure S104: ^{1}H NMR spectrum of 2-methyl-5-phenylpentan-5,5-d_2-2-ol in $CDCl_3$.

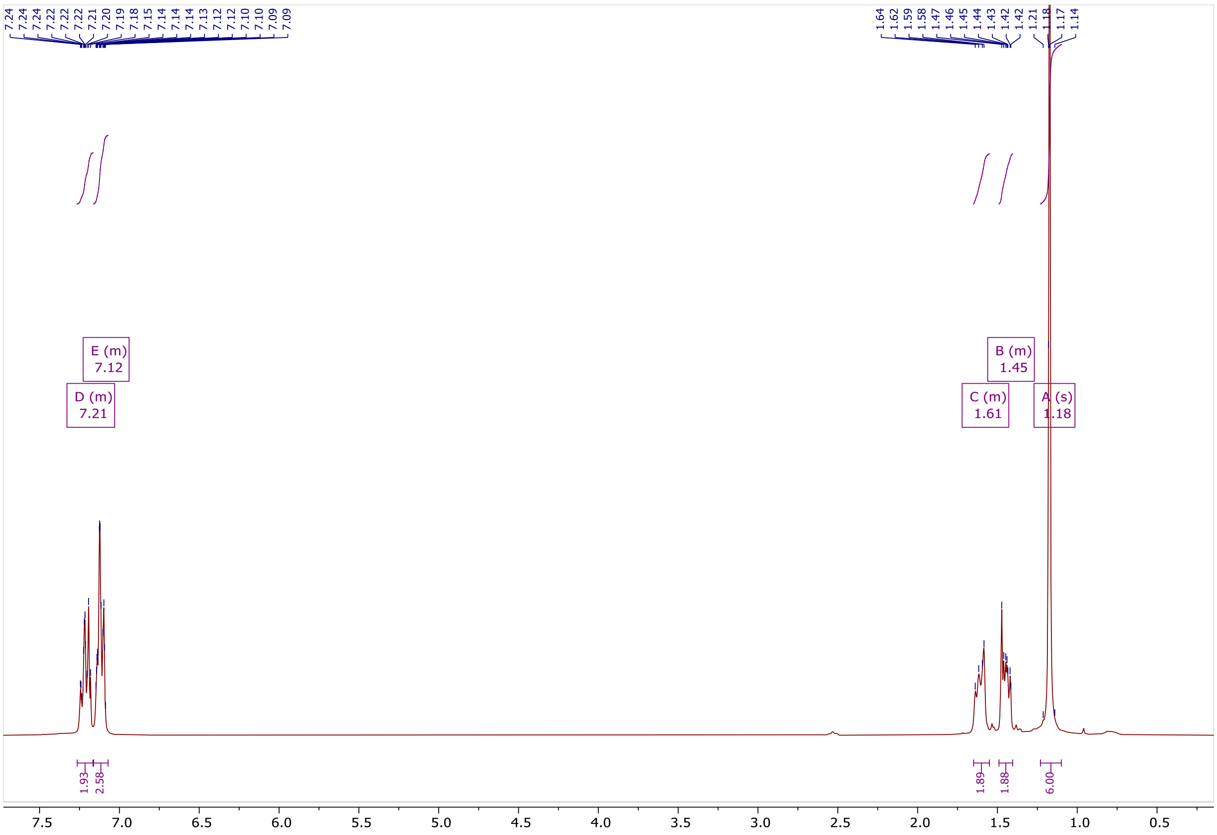

Figure S105: ^{1}H NMR spectrum of substrate **1a-d$_2$** in $CDCl_3$.

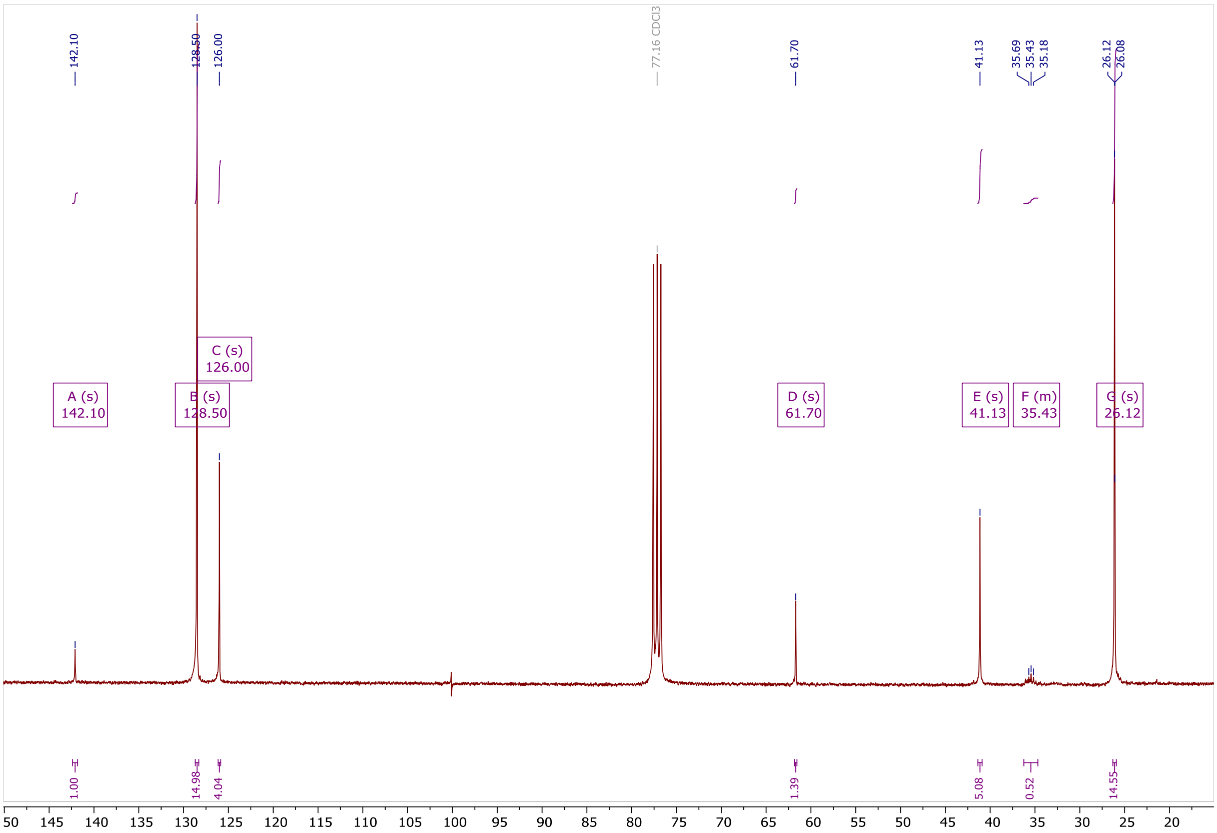

Figure S106: ^{13}C NMR spectrum of substrate **1a-d$_2$** in $CDCl_3$.

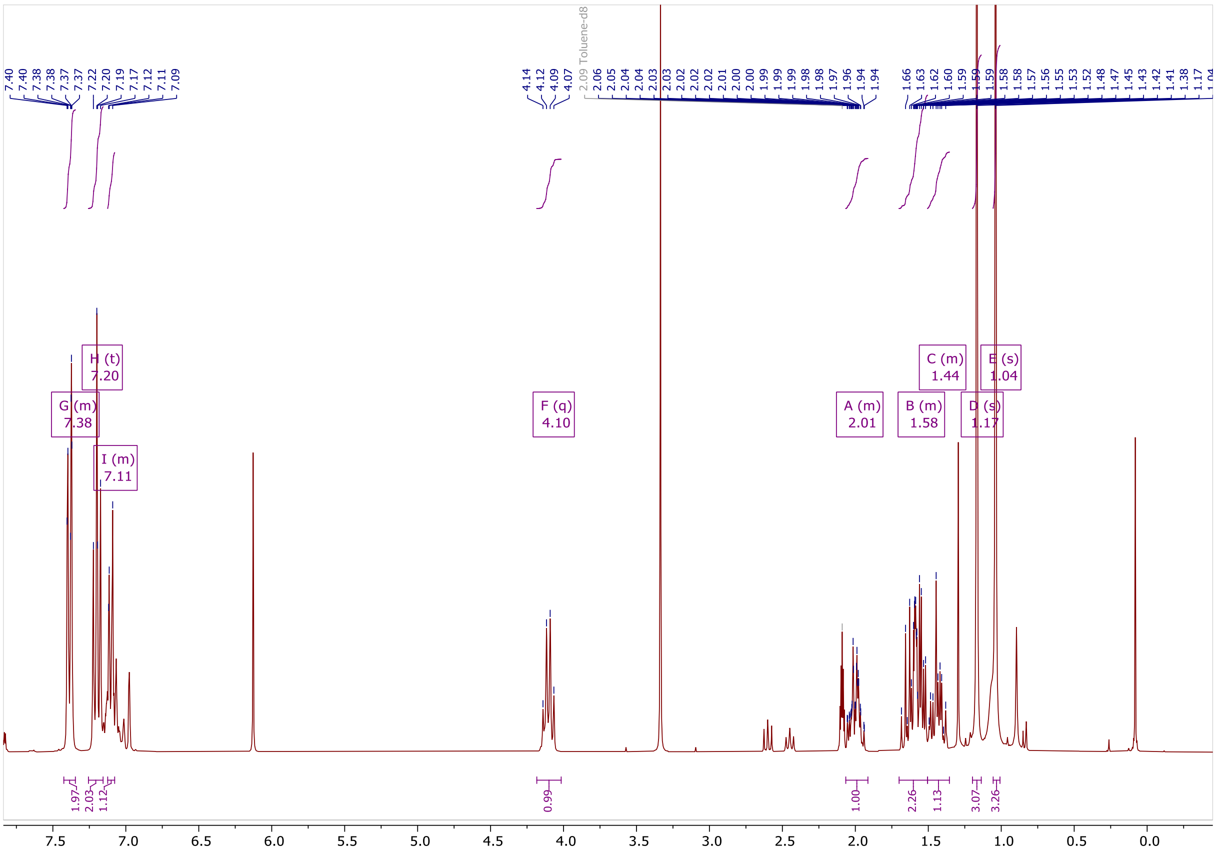

Figure S107: ^{1}H NMR spectrum of crude **1b** (t = 5 days) in toluene-d_8 with 1,3,5-Trimethoxybenzene as internal standard.

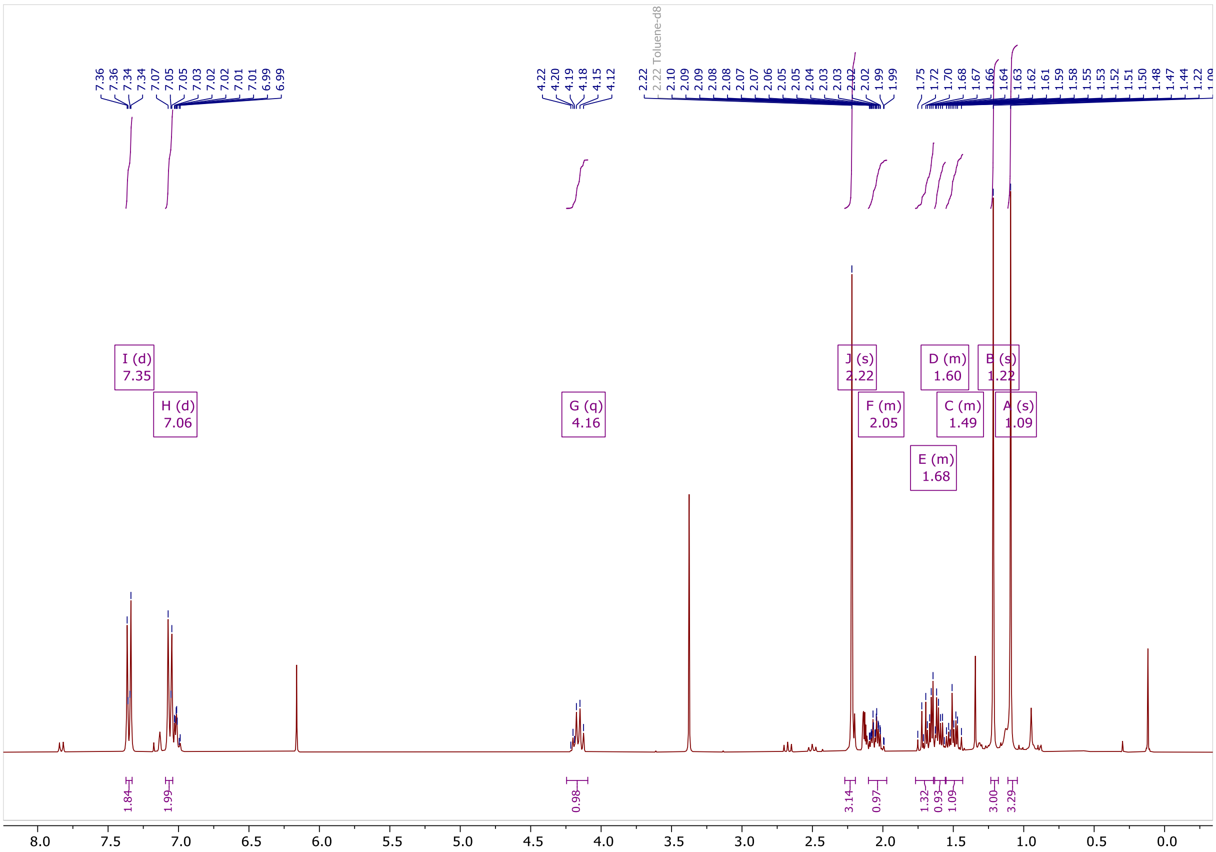

Figure 108: ^{1}H NMR spectrum of crude **5b** (t = 24 h) in toluene-d_8 with 1,3,5-Trimethoxybenzene as internal standard.

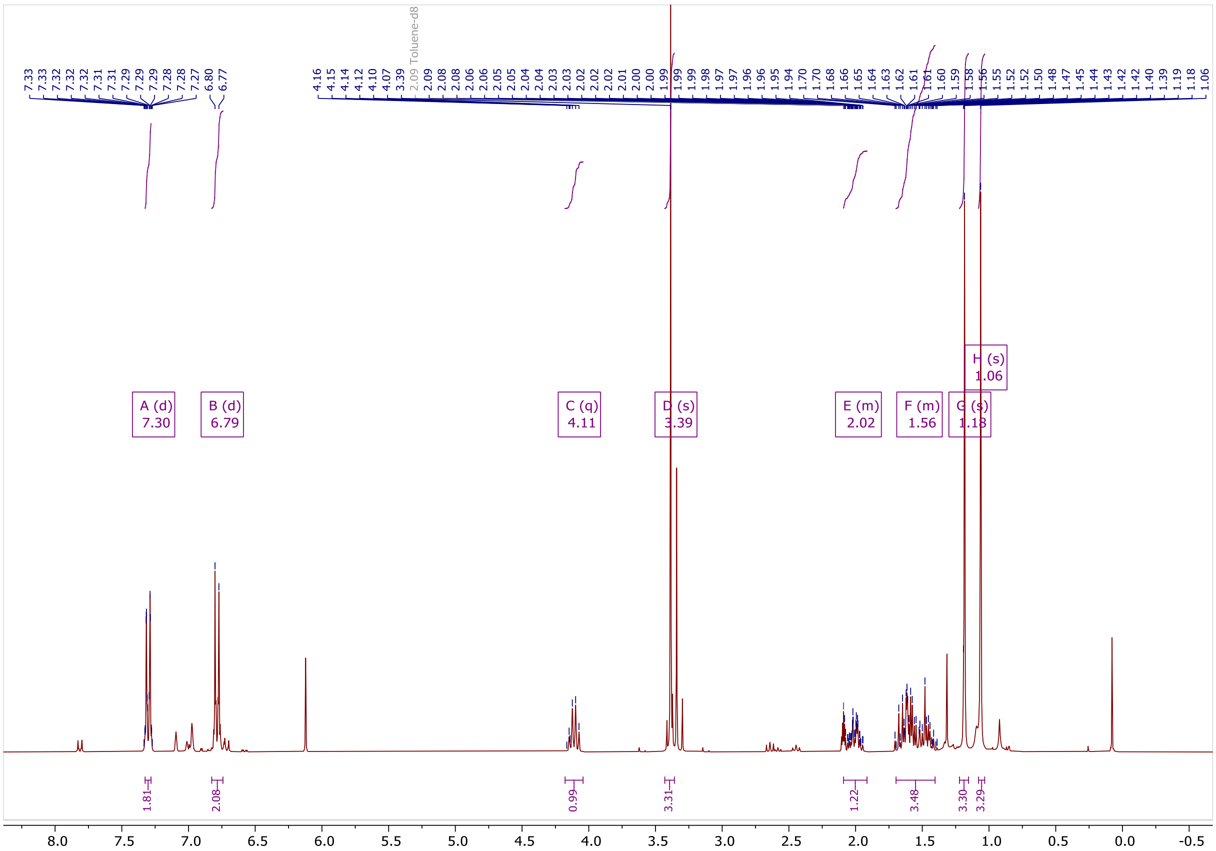

Figure S109: ^{1}H NMR spectrum of crude **6b** (t = 24 h) in toluene-d_8 with 1,3,5-Trimethoxybenzene as internal standard.

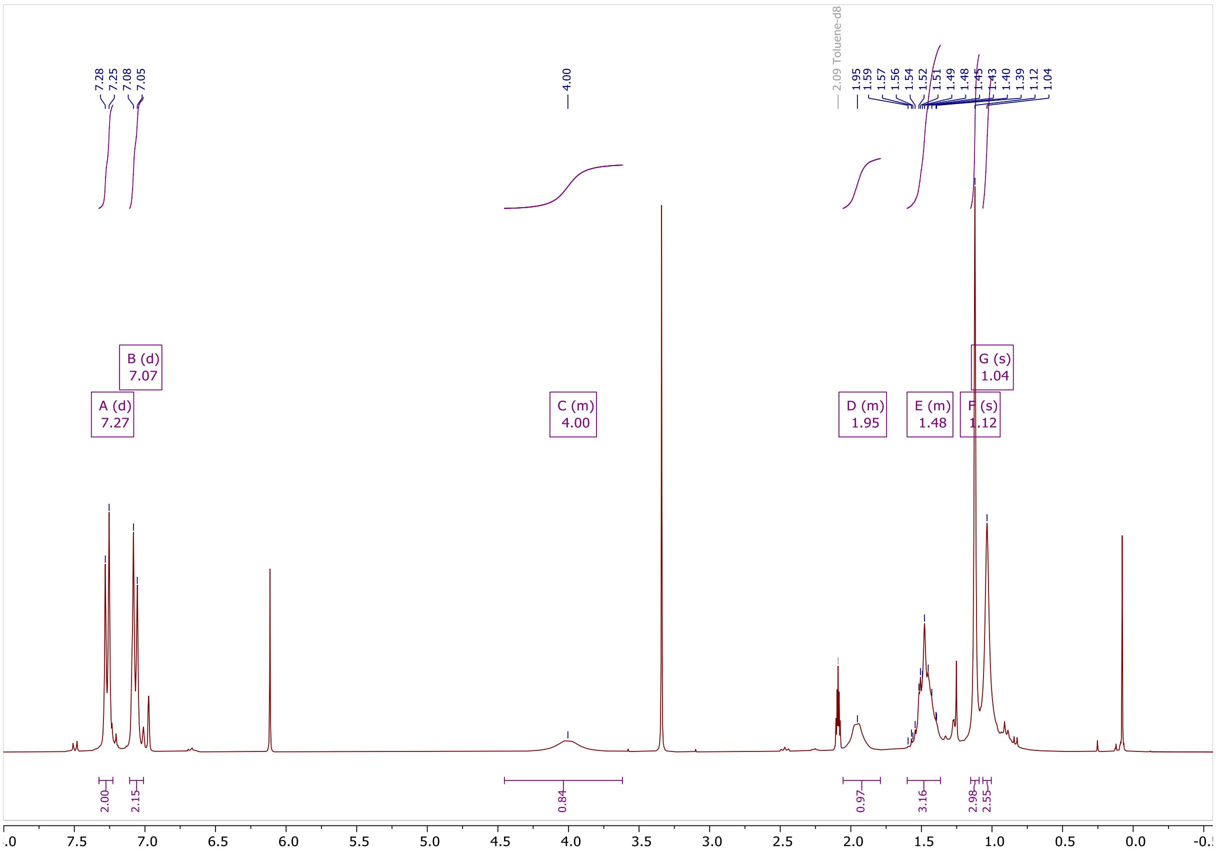

Figure S110: ^{1}H NMR spectrum of crude **7b** (t = 24 h) in toluene-d_8 with 1,3,5-Trimethoxybenzene as internal standard.

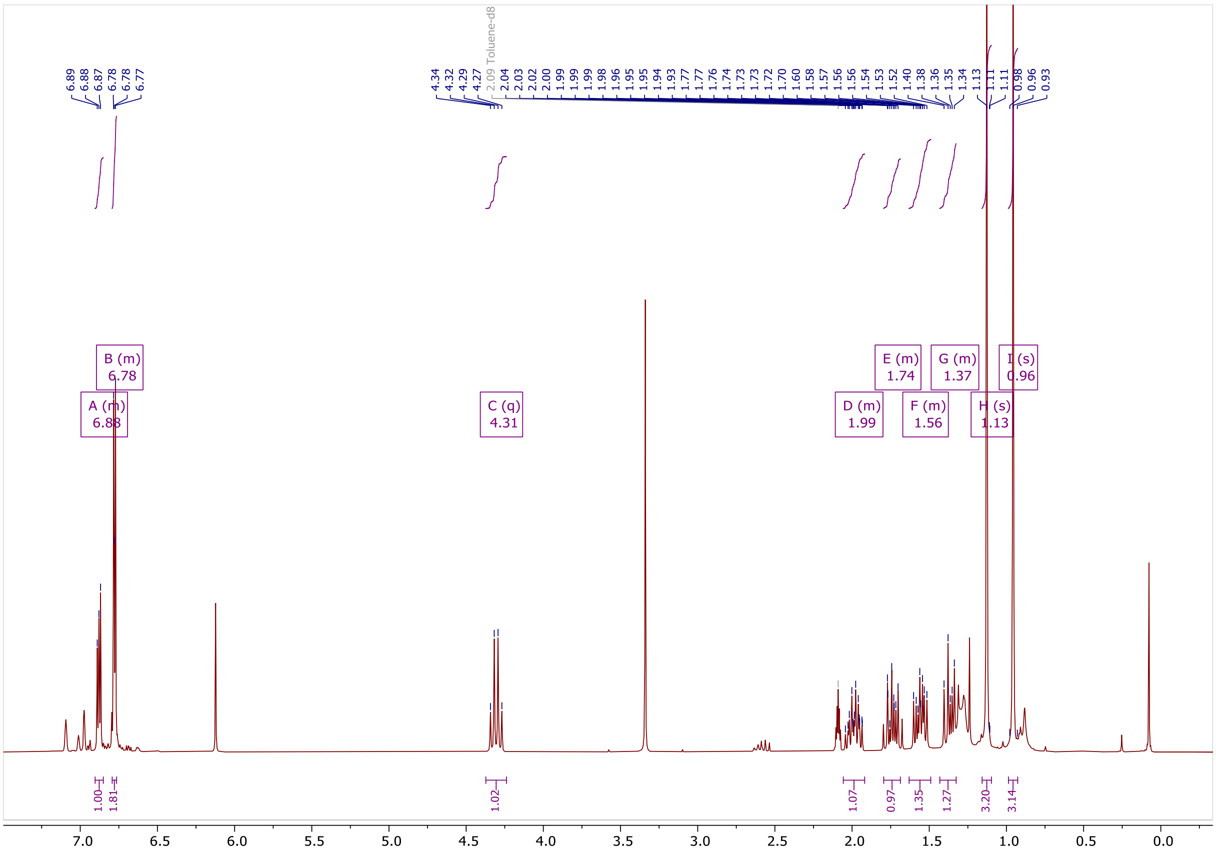

Figure S111: ^{1}H NMR spectrum of crude **8b** (t = 24 h) in toluene-d_8 with 1,3,5-Trimethoxybenzene as internal standard.

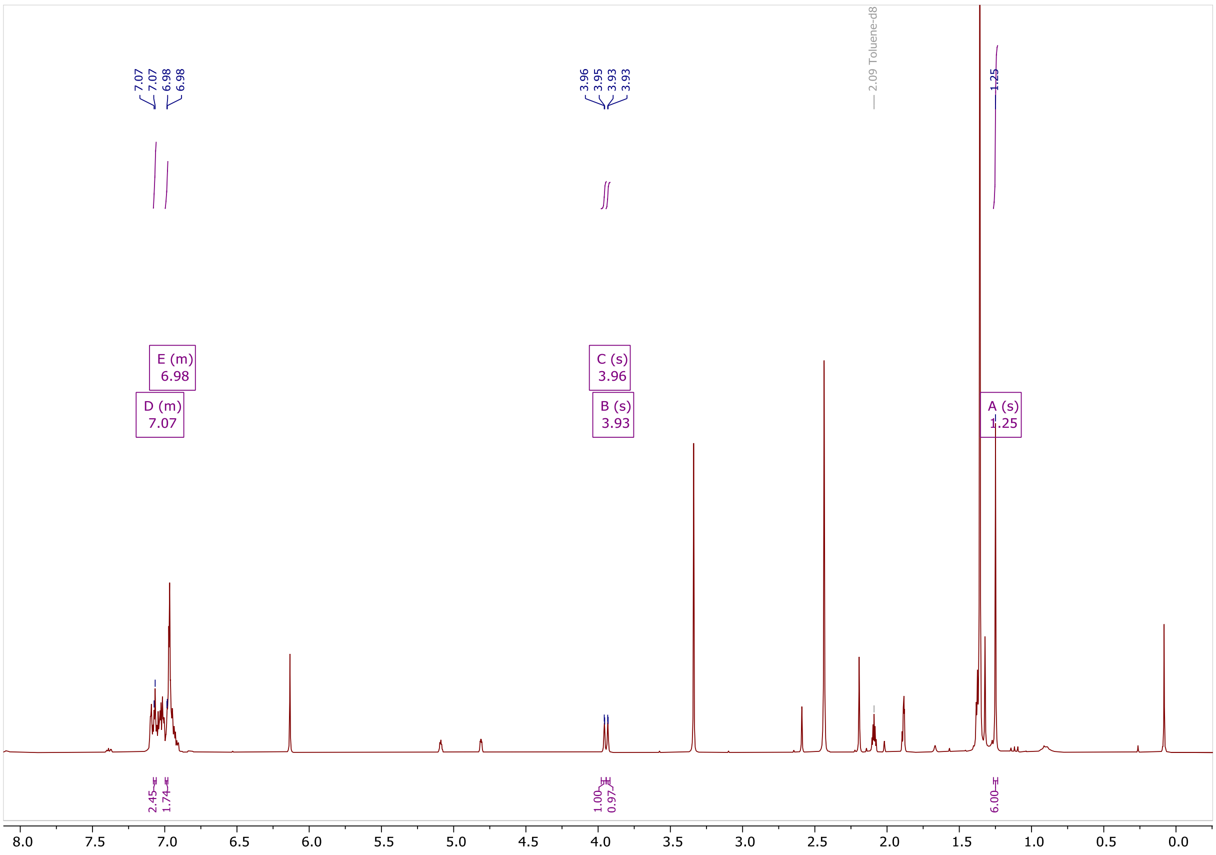

Figure S112: ^{1}H NMR spectrum of crude **9b** (t = 24 h) in toluene-d_8 with 1,3,5-Trimethoxybenzene as internal standard.

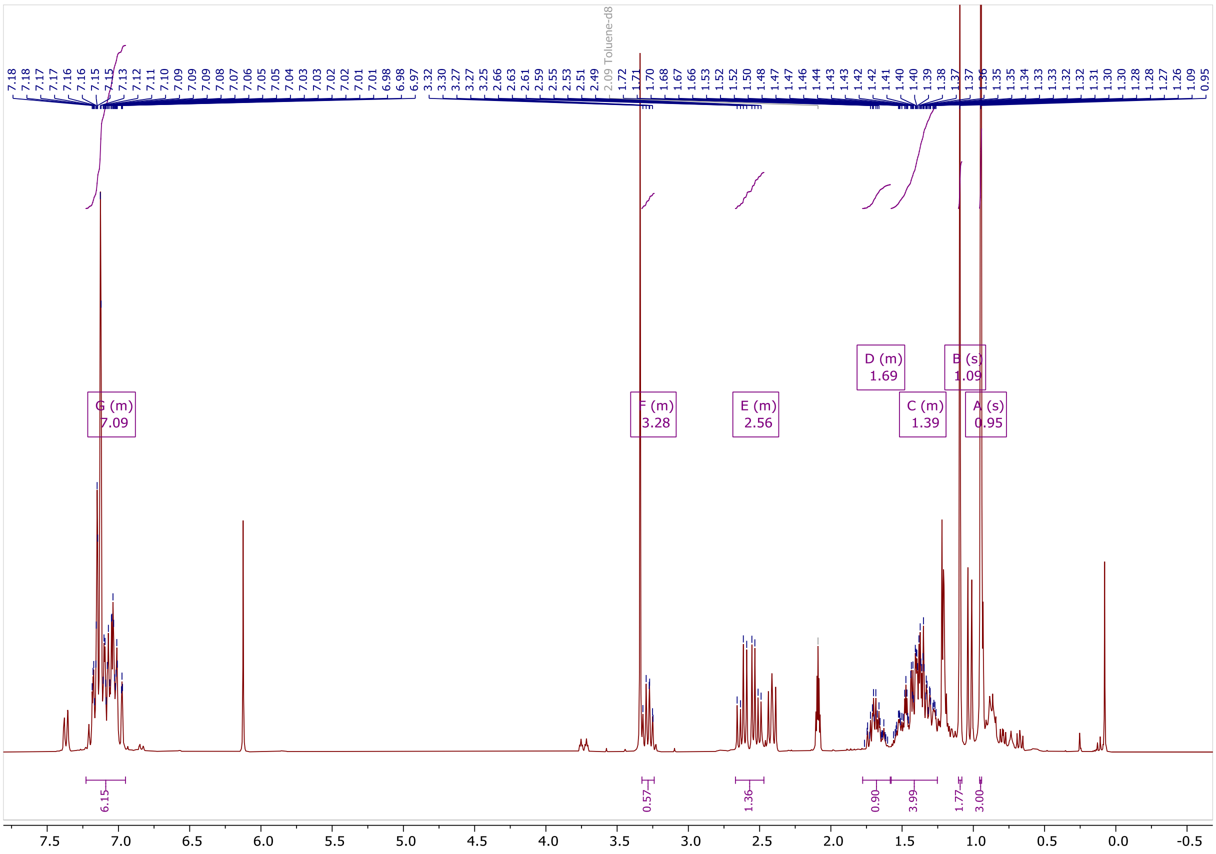

Figure S113: ^{1}H NMR spectrum of crude **10b** (t = 24 h) in toluene-d_8 with 1,3,5-Trimethoxybenzene as internal standard.

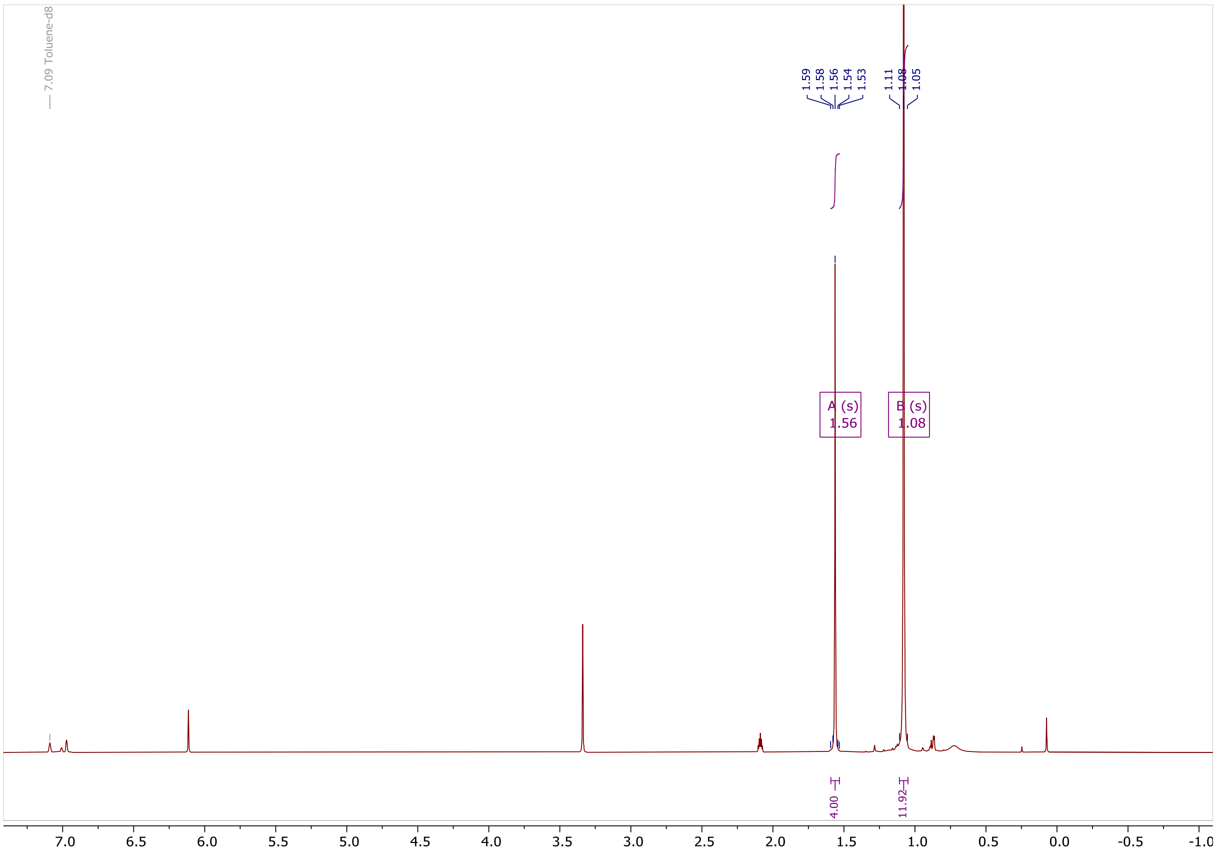

Figure S114: ^{1}H NMR spectrum of crude **11b** (t = 24 h) in toluene-d_8 with 1,3,5-Trimethoxybenzene as internal standard.

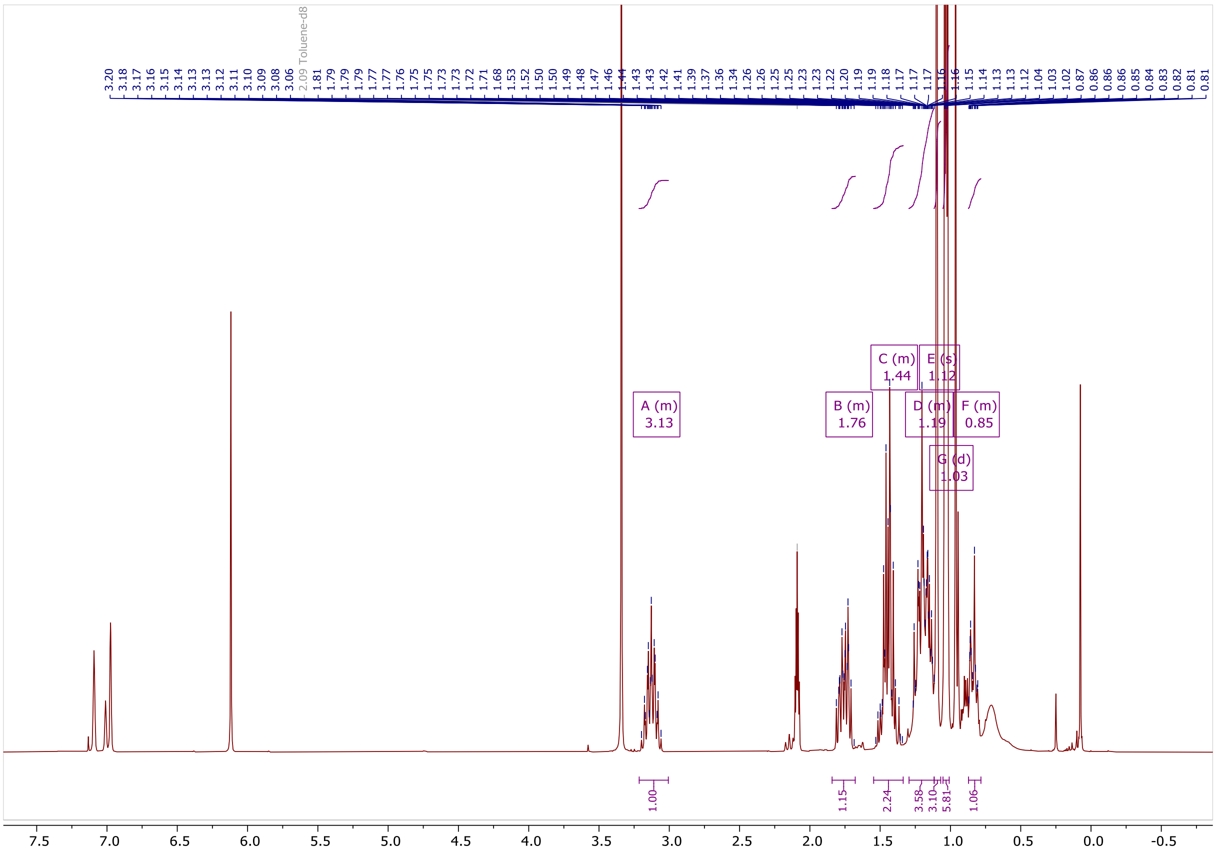

Figure S115: ^{1}H NMR spectrum of crude **12b** (t = 24 h) in toluene-d_8 with 1,3,5-Trimethoxybenzene as internal standard.

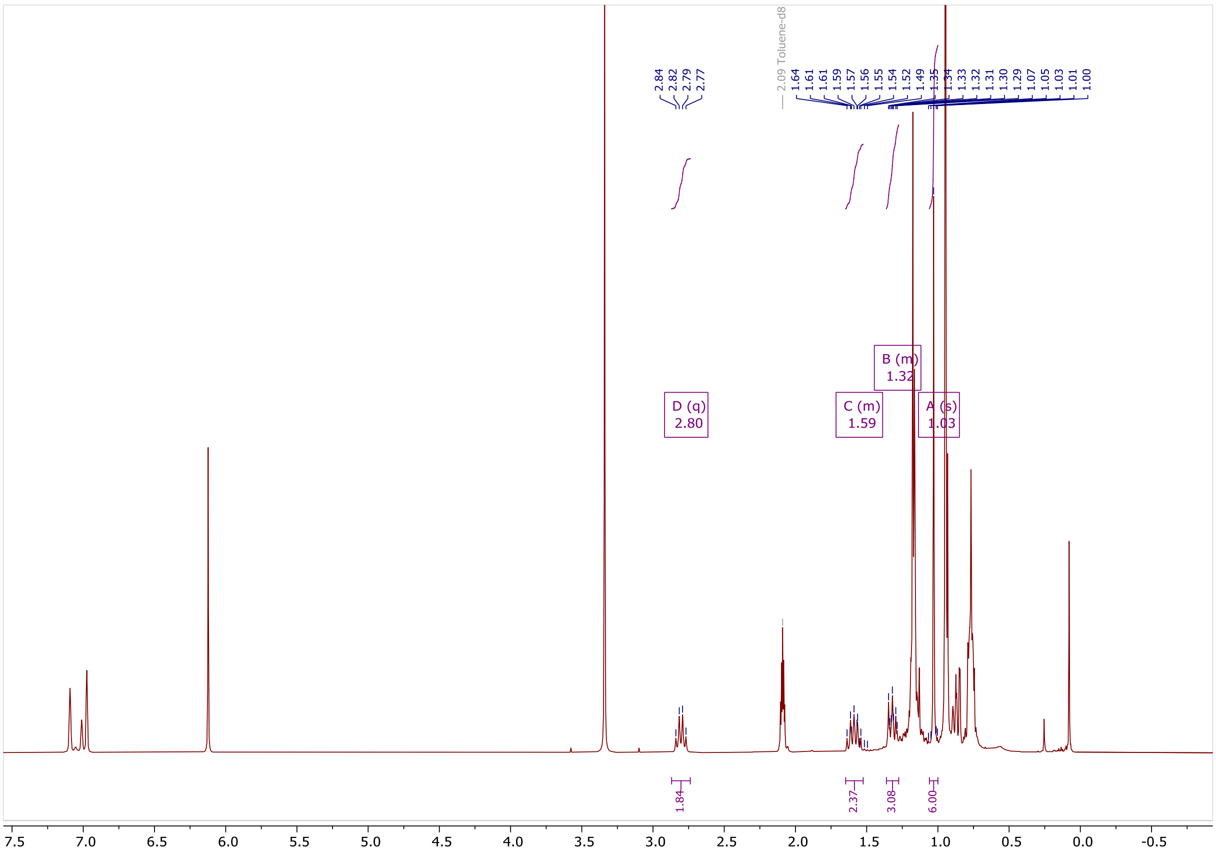

Figure S116: ^{1}H NMR spectrum of crude **13b** (t = 24 h) in toluene-d_8 with 1,3,5-Trimethoxybenzene as internal standard.

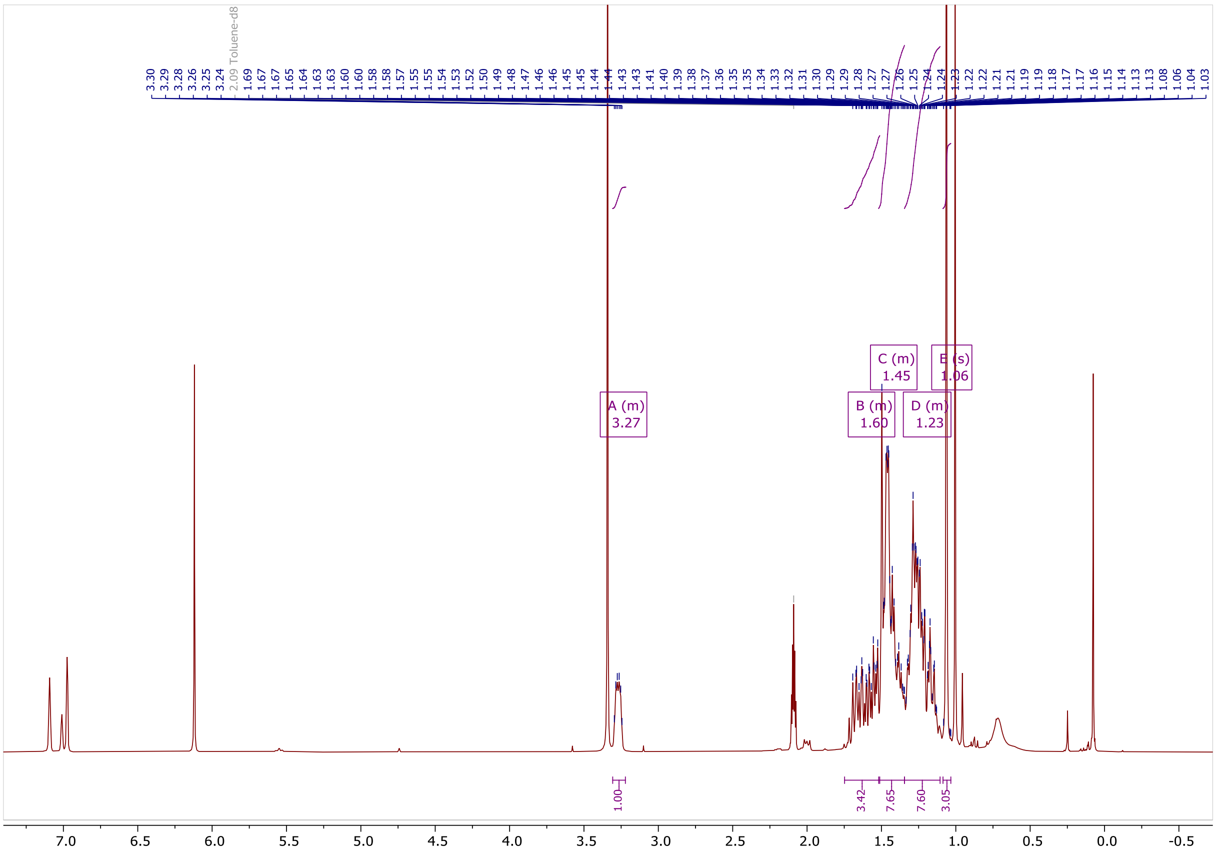

Figure S117: ^{1}H NMR spectrum of crude **14b** (t = 24 h) in toluene-d_8 with 1,3,5-Trimethoxybenzene as internal standard.

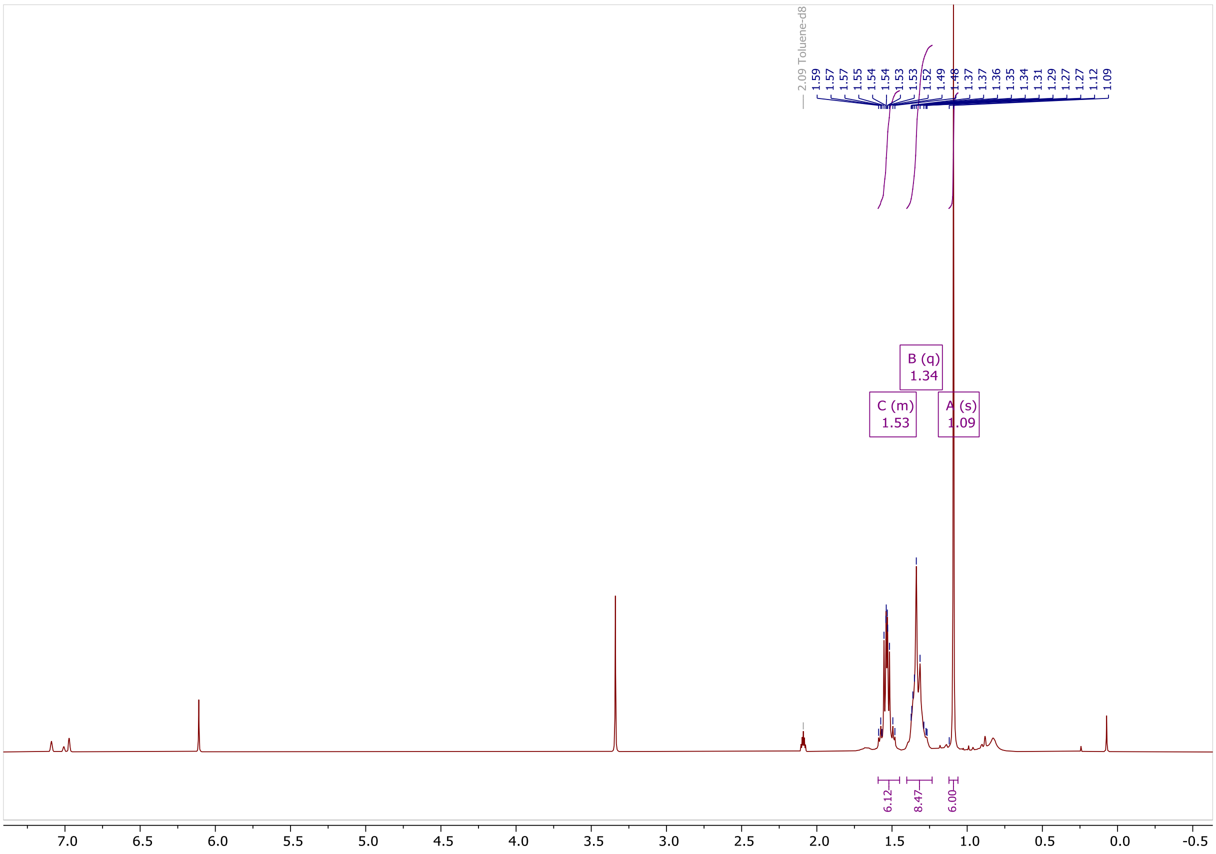

Figure S118: ^{1}H NMR spectrum of crude **15b** (t = 24 h) in toluene-d_8 with 1,3,5-Trimethoxybenzene as internal standard.

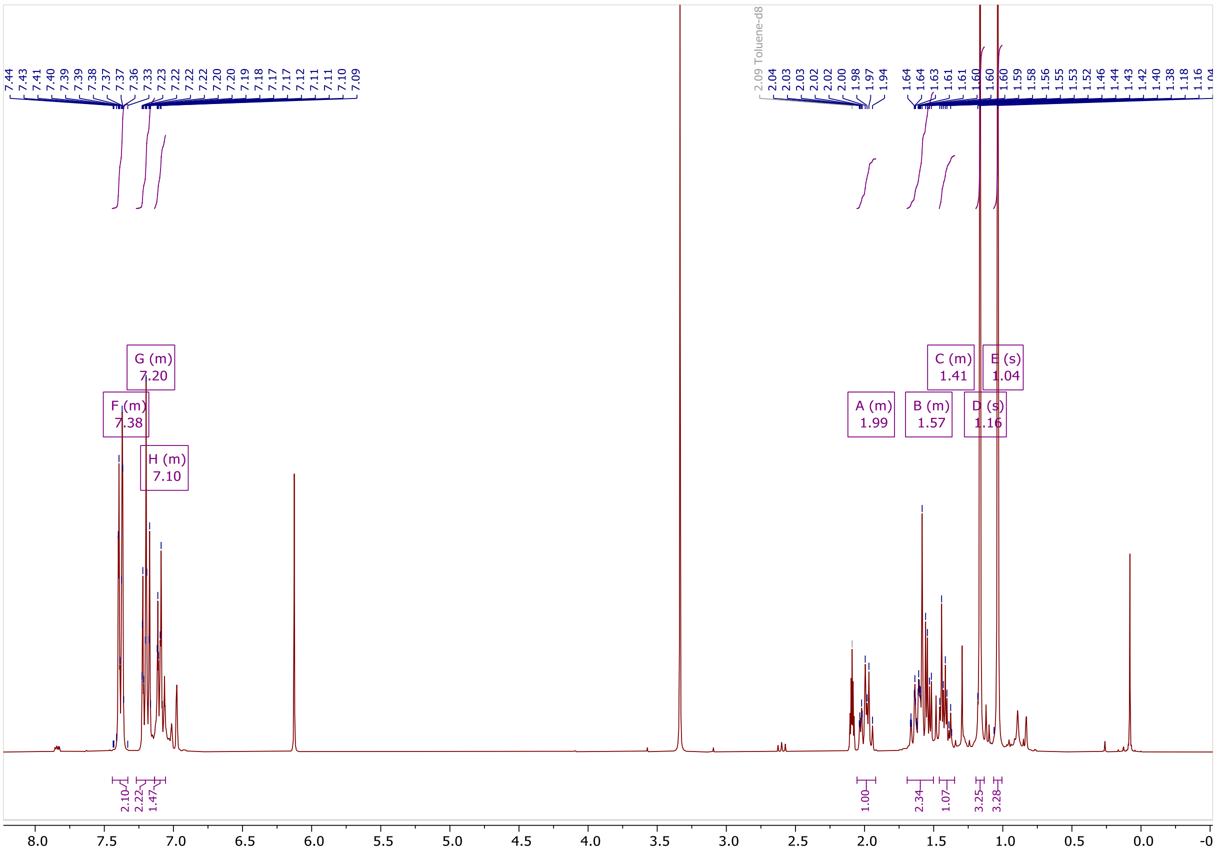

Figure S119: ^{1}H NMR spectrum of crude **1b-d$_2$** (t = 5 days) in toluene-d$_8$ with 1,3,5-Trimethoxybenzene as internal standard.

Crystallographic and refinement data

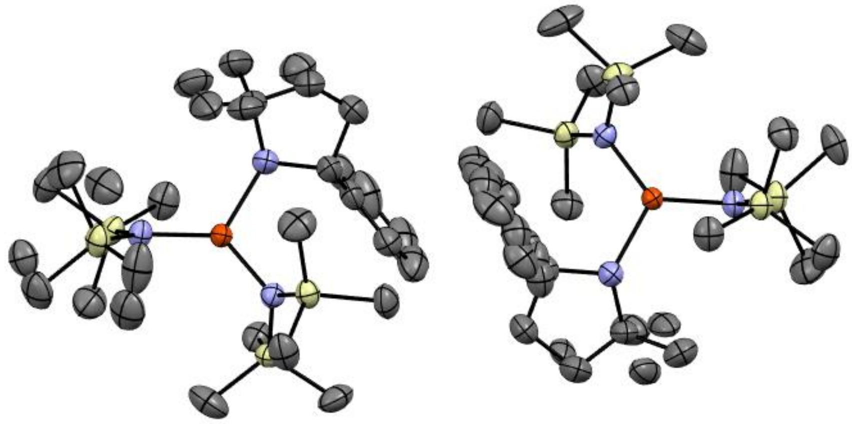

Figure S120: ORTEP representation of $Fe(HMDS)_2$ with a coordinated amine product (50% probability ellipsoids, H atoms omitted for clarity).

Table S6: Crystal data and structure refinement for WS381.

**Identification code**	21MA168_WS381
**CCDC deposit number**	2171951
**Empirical formula**	$C_{24}H_{53}FeN_3Si_4$
**Formula weight**	551.90
**Temperature/K**	173.01(10)
**Crystal system**	monoclinic
**Space group**	$P2_1/n$
**a/Å**	18.67067(17)
**b/Å**	18.94109(18)
**c/Å**	18.90816(17)
**α/°**	90
**β/°**	94.3084(8)
**γ/°**	90
**Volume/Å^3**	6667.84(11)
**Z**	8
**ρ_{calc}g/cm^3**	1.100
**μ/mm^{-1}**	5.110
**F(000)**	2400.0
**Crystal size/mm^3**	0.258 × 0.226 × 0.083
**Radiation**	Cu Kα (λ = 1.54184)
**2Θ range for data collection/°**	6.416 to 153.478
**Index ranges**	-23 ≤ h ≤ 23, -23 ≤ k ≤ 22, -23 ≤ l ≤ 23
**Reflections collected**	132519
**Independent reflections**	14043 [R_{int} = 0.0583, R_{sigma} = 0.0238]
**Data/restraints/parameters**	14043/90/742
**Goodness-of-fit on F^2**	1.076
**Final R indexes [I>=2σ (I)]**	R_1 = 0.0509, wR_2 = 0.1462
**Final R indexes [all data]**	R_1 = 0.0572, wR_2 = 0.1523
**Largest diff. peak/hole / e Å^{-3}**	0.97/-0.58

References

[S1] Fulmer, G. R.; Miller, A. J. M.; Sherden, N. H.; Gottlieb, H. E.; Nudelman, A.; Stoltz, B. M.; Bercaw, J. E.; Goldberg, K. I. NMR Chemical Shifts of Trace Impurities: Common Laboratory Solvents, Organics, and Gases in Deuterated Solvents Relevant to the Organometallic Chemist. *Organometallics* **2010**, *29*, 2176–2179.

[S2] Neese, F. The ORCA Program System. *Wiley Interdiscip. Rev. Comput. Mol. Sci* **2012**, *2*, 73–78.

[S3] Neese, F. Software Update: The ORCA Program System, Version 4.0. *Wiley Interdiscip. Rev. Comput. Mol. Sci* **2018**, *8*, 1–6.

[S4] Lee, C.; Yang, W.; Parr, R. G. Development of the Colle-Salvetti Correlation-Energy Formula into a Functional of the Electron Density. *Phys. Rev. B Condens. Matter Mater. Phys.* **1988**, *37*, 785–789.

[S5] Becke, A. D. Density-Functional Thermochemistry . III . The Role of Exact Exchange. *J. Chem. Phys.* **1993**, *98*, 5648–5652.

[S6] Weigend, F.; Häser, M.; Patzelt, H.; Ahlrichs, R. RI-MP2 : Optimized Auxiliary Basis Sets and Demonstration of Efficiency. *Chem. Phys. Lett.* **1998**, *294*, 143–152.

[S7] Weigend, F.; Ahlrichs, R. Balanced Basis Sets of Split Valence, Triple Zeta Valence and Quadruple Zeta Valence Quality for H to Rn: Design and Assessment of Accuracy. *Phys. Chem. Chem. Phys.* **2005**, *7*, 3297–3305.

[S8] Neese, F.; Izsák, R. An Overlap Fitted Chain of Spheres Exchange Method. *J. Chem. Phys.* **2011**, *135*, 144105–144111.

[S9] Weigend, F. Accurate Coulomb-Fitting Basis Sets for H to Rn. *Phys. Chem. Chem. Phys.* **2006**, *8*, 1057–1065.

[S10] Grimme, S.; Antony, J.; Ehrlich, S.; Krieg, H. A Consistent and Accurate Ab Initio Parametrization of Density Functional Dispersion Correction (DFT-D) for the 94 Elements H-Pu. *J. Chem. Phys.* **2010**, *132*.

[S11] Grimme, S. Supramolecular Binding Thermodynamics by Dispersion-Corrected Density Functional Theory. *Chem. Eur. J.* **2012**, *18*, 9955–9964.

[S12] Chemcraft - Graphical Software for Visualization of Quantum Chemistry Computations. Https://Www.Chemcraftprog.Com.

[S13] Perdew, J. P. Density-Functional Approximation for the Correlation Energy of the Inhomogeneous Electron Gas. *Phys. Rev. B* **1986**, *33*, 8822–8824.

[S14] Perdew, J. P. Erratum: Density-Functional Approximation for the Correlation Energy of the Inhomogeneous Electron Gas [Phys. Rev. B 33, 8822 (1986)]. *Phys. Rev. B* **1986**, *34*, 7406.

[S15] Eichkorn, K.; Treutler, O.; Öhm, H.; Häser, M.; Ahlrichs, R. Auxiliary Basis Sets to Approximate Coulomb Potentials. *Chem. Phys. Lett.* **1995**, *240*, 283–290.

[S16] Hellweg, A.; Hättig, C.; Höfener, S.; Klopper, W. Optimized Accurate Auxiliary Basis Sets for RI-MP2 and RI-CC2 Calculations for the Atoms Rb to Rn. *Theor. Chem. Acc.* **2007**, *117*, 587–597.

[S17] Hanwell, M. D.; Curtis, D. E.; Lonie, D. C.; Vandermeersch, T.; Zurek, E.; Hutchison, G. R. Avogadro: An Advanced Semantic Chemical Editor, Visualization, and Analysis Platform. *J. Cheminform.* **2012**, *4*, 1–17.

[S18] Angeli, C.; Cimiraglia, R.; Evangelisti, S.; Leininger, T.; Malrieu, J. Introduction of n -Electron

Valence States for Multireference Perturbation Theory. *J. Chem. Phys.* **2001**, *114*, 10252–10264.

[S19] Angeli, C.; Cimiraglia, R.; Malrieu, J.-P. N-Electron Valence State Perturbation Theory: A Fast Implementation of the Strongly Contracted Variant. *Chem. Phys. Lett.* **2001**, *350*, 297–305.

[S20] Angeli, C.; Cimiraglia, R.; Malrieu, J. N-Electron Valence State Perturbation Theory: A Spinless Formulation and an Efficient Implementation of the Strongly Contracted and of the Partially Contracted Variants. *J. Chem. Phys.* **2002**, *117*.

[S21] Knizia, G. Intrinsic Atomic Orbitals: An Unbiased Bridge between Quantum Theory and Chemical Concepts. *J. Chem. Theory Comput.* **2013**, *9*, 4834–4843.

[S22] Oxford Diffraction (2018). CrysAlisPro (Version 1.171.40.37a). Oxford Diffraction Ltd., Yarnton, Oxfordshire, UK.

[S23] Sheldrick, G. M. (2015). Acta Cryst. A71, 3-8.

[S24] Sheldrick, G. M. (2015). Acta Cryst. C71, 3-8.

[S25] Dolomanov, O. V.; Bourhis, L. J.; Gildea, R. J.; Howard, J. A. K.; Puschmann, H. OLEX2: A Complete Structure Solution, Refinement and Analysis Program. *J. Appl. Crystallogr.* **2009**, *42*, 339–341.

[S26] Maddock, L. C. H.; Cadenbach, T.; Kennedy, A. R.; Borilovic, I.; Aromí, G.; Hevia, E. Accessing Sodium Ferrate Complexes Containing Neutral and Anionic N-Heterocyclic Carbene Ligands: Structural, Synthetic, and Magnetic Insights. *Inorg. Chem.* **2015**, *54*, 9201–9210.

[S27] Andersen, R. A.; Faegri, K.; Green, J. C.; Haaland, A.; Lappert, M. F.; Leung, W.; Rypdal, K. Synthesis of Bis [Bis(Trimethylsilyl) Amido] Iron(II). Structure and Bonding in M[N(SiMe3)2]2 (M = Mn, Fe, Co): Two-Coordinate Transition-Metal Amides. *Inorg. Chem.* **1988**, *27*, 1782–1786.

[S28] Hennessy, E. T.; Betley, T. A. Complex N-Heterocycle Synthesis via Iron-Catalyzed, Direct C-H Bond Amination. *Science* **2013**, *340*, 591–595.

[S29] Baek, Y.; Betley, T. A. Catalytic C-H Amination Mediated by Dipyrrin Cobalt Imidos. *J. Am. Chem. Soc.* **2019**, *141*, 7797–7806.

[S30] Shimogaki, M.; Fujita, M.; Sugimura, T. Metal-Free Enantioselective Oxidative Arylation of Alkenes: Hypervalent-Iodine-Promoted Oxidative C−C Bond Formation. *Angew. Chem., Int. Ed.* **2016**, *55*, 15797–15801.

[S31] Álvarez, S.; Álvarez, R.; Khanwalkar, H.; Germain, P.; Lemaire, G.; Rodríguez-Barrios, F.; Gronemeyer, H.; de Lera, Á. R. Retinoid Receptor Subtype-Selective Modulators through Synthetic Modifications of RARγ Agonists. *Bioorganic Med. Chem.* **2009**, *17*, 4345–4359.

[S32] Dong, Y.; Clarke, R. M.; Porter, G. J.; Betley, T. A. Efficient C-H Amination Catalysis Using Nickel-Dipyrrin Complexes. *J. Am. Chem. Soc.* **2020**, *142*, 10996–11005.

[S33] Li, N. N.; Zhang, Y. L.; Mao, S.; Gao, Y. R.; Guo, D. D.; Wang, Y. Q. Palladium-Catalyzed C-H Homocoupling of Furans and Thiophenes Using Oxygen as the Oxidant. *Org. Lett.* **2014**, *16*, 2732–2735.

[S34] Estévez, M. C.; Galve, R.; Sánchez-Baeza, F.; Marco, M. P. Disulfide Symmetric Dimers as Stable Pre-Hapten Forms for Bioconjugation: A Strategy to Prepare Immunoreagents for the Detection of Sulfophenyl Carboxylate Residues in Environmental Samples. *Chem. Eur. J.* **2008**, *14*, 1906–1917.

[S35] Khalaf, A. A.; Roberts, R. M. Friedel-Crafts Cyclialkylations of Certain Mono- and Diphenylsubstituted Alcohols and Alkyl Chlorides. *J. Org. Chem.* **1972**, *37*, 4227–4235.

[S36] Stroek, W.; Keilwerth, M.; Pividori, D. M.; Meyer, K.; Albrecht, M. An Iron–Mesoionic Carbene Complex for Catalytic Intramolecular C–H Amination Utilizing Organic Azides. *J. Am. Chem. Soc.* **2021**, *143*, 20157–20165.

[S37] Zhao, J.; Zhao, X. J.; Cao, P.; Liu, J. K.; Wu, B. Polycyclic Azetidines and Pyrrolidines via Palladium-Catalyzed Intramolecular Amination of Unactivated C(Sp3)-H Bonds. *Org. Lett.* **2017**, *19*, 4880–4883.

[S38] Łażewska, D.; Mogilski, S.; Hagenow, S.; Kuder, K.; Głuch-Lutwin, M.; Siwek, A.; Więcek, M.; Kaleta, M.; Seibel, U.; Buschauer, A.; Filipek, B.; Stark, H.; Kieć-Kononowicz, K. Alkyl Derivatives of 1,3,5-Triazine as Histamine H 4 Receptor Ligands. *Bioorganic Med. Chem.* **2019**, *27*, 1254–1262.

[S39] Okazawa, N. E.; Sorensen, T. S. Solution Carbocation Stabilities Measured by Internal Competition for a Hydride Ion. *Can. J. Chem.* **1982**, *60*, 2180–2193.

[S40] Jeon, J. Y.; Park, S.; Han, J.; Maurya, S.; Mohanty, A. D.; Tian, D.; Saikia, N.; Hickner, M. A.; Ryu, C. Y.; Tuckerman, M. E.; Paddison, S. J.; Kim, Y. S.; Bae, C. Synthesis of Aromatic Anion Exchange Membranes by Friedel-Crafts Bromoalkylation and Cross-Linking of Polystyrene Block Copolymers. *Macromolecules* **2019**, *52*, 2139–2147.
